# Bayesian optimization-driven parallel-screening of multiple parameters for the flow synthesis of biaryl compounds

**DOI:** 10.1038/s42004-022-00764-7

**Published:** 2022-11-10

**Authors:** Masaru Kondo, H. D. P. Wathsala, Mohamed S. H. Salem, Kazunori Ishikawa, Satoshi Hara, Takayuki Takaai, Takashi Washio, Hiroaki Sasai, Shinobu Takizawa

**Affiliations:** 1 Department of Materials Science and Engineering, Graduate School of Science and Engineering, Ibaraki University, Naka-narusawa, Hitachi, Ibaraki 316-8511 Japan; 2 SANKEN, Osaka University, Mihogaoka, Ibaraki-shi, Osaka 567-0047 Japan; 3 Pharmaceutical Organic Chemistry Department, Faculty of Pharmacy, Suez Canal University, Ismailia, 41522 Egypt; 4 Artificial Intelligence Research Center, SANKEN, Osaka University, Suita, Japan; 5 Graduate School of Pharmaceutical Sciences, Osaka University, Yamada-oka, Suita-shi, Osaka 565-0871 Japan

**Keywords:** Synthetic chemistry methodology, Cheminformatics, Organocatalysis, Flow chemistry

## Abstract

Traditional optimization methods using one variable at a time approach waste time and chemicals and assume that different parameters are independent from one another. Hence, a simpler, more practical, and rapid process for predicting reaction conditions that can be applied to several manufacturing environmentally sustainable processes is highly desirable. In this study, biaryl compounds were synthesized efficiently using an organic Brønsted acid catalyst in a flow system. Bayesian optimization-assisted multi-parameter screening, which employs one-hot encoding and appropriate acquisition function, rapidly predicted the suitable conditions for the synthesis of 2-amino-2′-hydroxy-biaryls (maximum yield of 96%). The established protocol was also applied in an optimization process for the efficient synthesis of 2,2′-dihydroxy biaryls (up to 97% yield). The optimized reaction conditions were successfully applied to gram-scale synthesis. We believe our algorithm can be beneficial as it can screen a reactor design without complicated quantification and descriptors.

## Abstract (web summary)

Data-driven methodology plays an important role in the rapid identification of appropriate chemical conditions, however, optimization of multiple variables in the flow reaction remains challenging. Here, the authors report a Bayesian optimization-assisted multi-parameter screening to predict the suitable conditions to achieve the efficient synthesis of biaryl compounds in a flow system.

## Introduction

Data-driven methodology enables the rapid identification of appropriate conditions for eco-friendly and sustainable chemical processes^1–4^. Among these methods, computational and automated protocols identifying suitable reaction conditions in continuous-flow system have been extensively investigated owing to their reproducibility, rapid heating, mixing, short reaction periods, and ease of automation^5–14^. Furthermore, Gaussian process regression can efficiently predict the appropriate reaction parameters of a flow reaction by estimating yields from a small dataset^15^. Despite the significant advances in this field, it is still difficult to efficiently and simultaneously optimize multiple flow reaction variables (e.g., flow rate, pipe diameter, and length, micromixer (reactor) type, and also other conventional reaction parameters). In recent, Bayesian optimization (BO), which is a powerful probabilistic method of determining the global maximum (or minimum) of a black-box objective function, is useful for multi-parameter screening in flow platform as well as batch system^16–21^. Our group also applied the BO-assisted screening of numerical parameters for electrochemical oxidation of amines to ketimines and electrochemical reductive carboxylation in flow^22,23^.

**Fig. 1:**
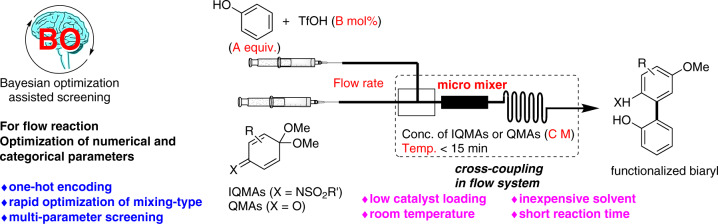
Bayesian optimization assisted screening for flow synthesis of functionalized biaryls under mild conditions. Bayesian optimization-driven parallel-screening on six numerical and categorical parameters of micromixer-types and organocatalytic conditions in the flow biaryl synthesis.

To utilize categorical chemical variables (e.g., solvents and reagents) for data-driven reaction optimization, the steric and electronic properties of a molecule were converted to the corresponding numeric values with descriptors^21,24–26^, which required precise structural representation, the quantum chemical properties and theoretical calculations for the construction of a practical model. It is difficult for even if experienced chemists and scientists, to attain the chemical reaction’s dataset with the selected categorical parameters and minimum features. It is also challenging to convert dominant, non-numerical parameters into numerical parameters through the selection of proper physical and engineering features^27–32^, although these categorical parameters are crucial to achieve good outcomes. To demonstrate a simpler and more practical BO-assisted method of identifying optimal reaction conditions, we focused on the direct optimization of categorical parameters with neither feature extraction nor model construction. In this study, we enhanced the BO algorithm by adopting a categorical variable as an integer value *via* one-hot encoding without employing one-hot encoding to avoid the effect of a relative magnitude between integer values^33,34^ (e.g., mixer A: ‘0’ represented by 1 0 0, mixer B: ‘1’ represented by 0 1 0, mixer C: ‘2’ represented by 0 0 1). A categorical variable can be rounded to the closest integer and induced to the appropriate value, along with the optimization of a larger number of continuous numerical factors. Using BO-assisted parallel screening of six numerical and categorical parameters, appropriate continuous flow synthetic conditions were determined for the production of functionalized biaryls *via* the redox-neutral cross-coupling reaction of iminoquinone monoacetals (IQMAs) or quinone monoacetals (QMAs) with arenols (Fig. 1).

Functionalized biaryl motifs are found in numerous natural products^35,36^, pharmaceuticals^37–39^, and chiral ligands^40–43^. These biaryl compounds are typically synthesized by transition-metal-catalyzed reactions such as cross-coupling and oxidative coupling of aryl compounds in a regio- and stereoselective manner^44–52^. More recently, an efficient, metal-free, and operationally simple organocatalysis process has been developed for synthesis of arenol-derived biaryls. In 2016, two independent studies reported readily available organic Brønsted acid-catalyzed cross-coupling in batch reactions of QMAs or IQMAs with arenols to afford phenol biaryls with the following organic acids: TFA or (PhO)_2_PO_2_H^51^; MeSO_3_H^52^. Although both studies reported good yields and a wide substrate scope, the reaction conditions had drawbacks, such as the high catalyst loading (20 mol%), long reaction period (16 h), and high reaction temperature (at 100 °C). We envisaged a flow system with higher mixing efficiency, good thermal conductivity, and easy reaction control could address more effective and green-sustainable synthetic process for the organocatalyzed cross-coupling without a formation of by-product.

## Results and discussion

### BO-assisted parallel screening of flow reaction conditions and evaluation of substrate scope

To determine a practical optimization methodology for the flow reaction conditions, we used IQMA **1a**, 2-naphthol **2a**, and a catalytic amount of TfOH in toluene to conduct six reactions to screen five continuous numerical parameters and one categorical parameter: amount of **2a** (1–3 equiv.), temperature (20–60 °C), concentration of **1a** in toluene (0.01–0.1 M), flow rate (0.05–0.2 mL/min), catalyst loading (0.5–2 mol%), and mixer type (Comet X, β-type, and T-shaped; for more information about micromixers, see Fig. S2 in Supplementary Note 3). We set a broader initial dataset using six, rather than three datapoints which can be inadequate to find suitable conditions, avoid expensive solvents and toxic reagent, and decrease the amount of chemicals (see Supplementary Data 2). The results are summarized in Table 1, entries 1–6.

**Table 1:** Screening of reaction conditions for cross-coupling of IQMA **1a** and 2-naphthol **2a**^a^.


Entry	micro mixer	2a (equiv.)	TfOH (mol%)	Temp. (˚C)	Conc. of 1a (M)	Flow rate (mL/min)	Residence time (min)	NMR yield^b^ (%)
1	Comet X^c^	2.0	1.0	60	0.05	0.05	24	68
2	Comet X	3.0	0.5	40	0.01	0.2	6	73
3	β-type^d^	1.0	2.0	60	0.01	0.1	13.5	42
4	β-type	3.0	0.5	20	0.1	0.1	13.5	28
5	T-shaped^e^	1.0	2.0	40	0.05	0.05	16	55
6	T-shaped	2.0	1.0	20	0.1	0.2	4	75
7	T-shaped	2.0	1.0	20	0.1	0.15	5.5	81
8	Comet X	2.3	1.0	55	0.039	0.04	30	77
9	β-type	1.1	2.4	85	0.15	0.1	13.5	40
10	T-shaped	1.3	1.2	15	0.11	0.15	5.5	78
11	T-shaped	2.1	1.1	30	0.061	0.15	5.5	76
12	Comet X	2.8	1.0	50	0.014	0.11	11	79
13	Comet X	3.4	1.3	55	0.01	0.032	37.5	88
14	Comet X	2.2	1.7	15	0.1	0.014	85.5	44
15	Comet X	3.0	1.5	25	0.015	0.08	15	96 (93)^f^

^a^Reaction conditions: **1a** (0.065 mmol), **2a**, and TfOH, in degassed dry toluene.

^b^1,3,5-Trimethoxybenzene was used as a standard.

^c^Volume of Comet X microreactor = 2.4 mL.

^d^Volume of β-type microreactor = 2.7 mL.

^e^Volume of T-shaped microreactor = 1.6 mL.

^f^Isolated yield.

On the other hand, using TfOH in a batch system did not afford the desired biaryls **3** due to the fast decomposition of IQMAs **1** under the strong acidic conditions of TfOH, and the generation of various side products. In the flow system, the rapid mixing of substrates and quenching of TfOH improved the yield by suppressing these undesired reaction pathways.

In our study, we employed BO using parallel lower confidence bounds (LCB) as an acquisition function^53^. Parallel BO efficiently evaluates an expensive objective function at several points, simultaneously. Optimization of the mixers was not efficiently achieved using other acquisition functions such as single EI (expected improvement), LCB, and parallel EI. With a batch size of three, each mixer was suggested along with next numerical parameters to examine (entries 7–9) based on the initial dataset in entries 1–6. The evaluation of these three estimated conditions by experiments reported a slightly improved yield (up to 81%) under the reaction conditions corresponding to entry 7. Further consideration of entries 1–9 with the BO protocol suggested the β-type mixer was not appropriate for the coupling of **1a** with **2a** as shown in entries 10–12. However, no improvement in the yield was observed with a use of Comet X and T-shaped mixers (maximum yield of 79%). Finally, we acquired three different experimental datapoints (entries 13–15) with Comet X through BO-assisted optimization based on the 12 conditions in entries 1–12. Gratifyingly, the desired product **3a** was obtained in 93% isolated yield using a microflow system (Comet X micro mixer, flow rate = 0.08 mL/min, and residence time = 15 min) as shown in entry 15. Screening of microreactors (T-shape, β-type, and Comet-X) showed Comet-X had superior performance under the same reaction conditions (see Scheme S1 in Supplementary Note 4).

**Fig. 2:**
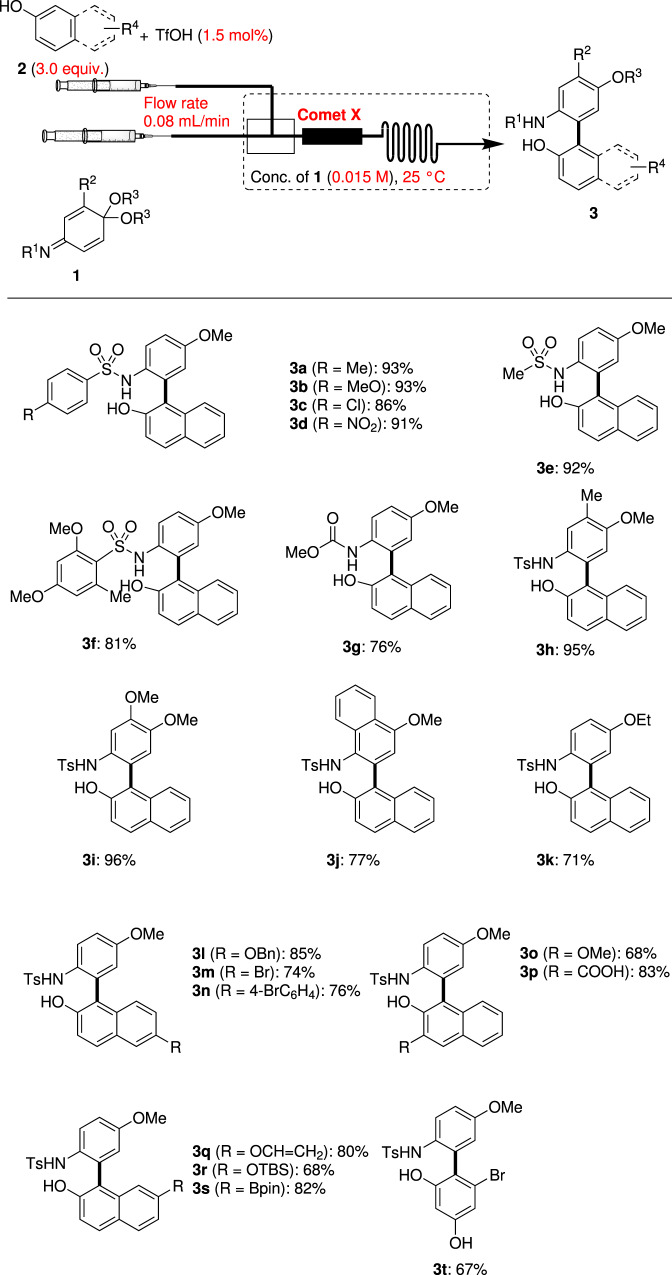
Substrate scope of cross-coupling using IQMAs **1** and arenols **2**^a^. Yields are those of isolated **3**. ^a^Reaction conditions: **1** (0.065 mmol), **2** (3.0 equiv.), and TfOH (1.5 mol%) in degassed dry toluene (0.015 M), micro mixer: Comet X, flow rate: 0.08 mL/min, at 25 °C.

Having BO-established suitable reaction conditions, we examined the substrate scope for the flow synthesis of biaryls using a variety of IQMAs **1** and arenols **2** in Fig. 2. IQMAs (**1b–d**) bearing an electron-donating or withdrawing substituent at the 4-position of the phenyl group; they were reacted with 2-naphthol (**2a**) to synthesize the corresponding products **3b–d** with good yields (86–93%). The sterically less hindered or bulky sulfonyl groups also produced biaryls **3e** and **3f** in 92 and 81% yields, respectively. The product **3g** was successfully obtained in 76% yield from methoxycarbonyl-protected IQMA **1g**. Similarly, mono-substituted IQMAs and iminonaphthoquinone monoacetals **1h**–**j** were converted into the corresponding coupling products **3h**–**j** (77–96% yields). The enantioselective coupling reaction of IQMA **1h** and 2-naphthol **2a** was tested using chiral phosphoric acid and the product **3h** was obtained in 78% yield with 23% ee, which is slightly higher than the reported values (see Supplementary Note 1)^51^. Moreover, diethyl acetal **1k** reacted smoothly with **2a** to give the biaryl product **3k** in 71% yield. IQMA **1a** was coupled with 2-naphthols bearing electron-withdrawing or donating groups **2b**–**d** at the 6-position to afford the corresponding 2-amino-2′-hydroxy-products **3l**–**n** with 74–85% yields. The reactions using 3-methoxy- and 3-carboxyl-2-naphthols **2e** and **2f** afforded the coupling products **3o** and **3p** in 68 and 83% yields, respectively. Vinyloxy- (**2g**), *tert*-butyl dimethyl siloxy- (**2h**), and pinacol boronic ester groups (**2i**) at the 7-position were also tested into the flow system, giving the desired products **3q**–**s** in 68–82% yields. Using 5-bromoresorcinol **2j** as a phenolic nucleophile, the coupling product **3t** was isolated in 67% yield.

Although a cross-coupling reaction using quinone monoacetal **4a** and **2j** for further extension of the substrate scope was performed under the optimized conditions, the isolated yield of the desired biarenol **5a** was only 38%. Thus, to determine the appropriate reaction conditions for QMAs, BO-assisted screening of **4a** and **2j** as model substrates was performed (Table 2).

**Table 2:** Screening of reaction conditions for cross-coupling QMA of **4a** and 5-bromoresorcinol **2j**^a^.


Entry	Micro mixer	2j (equiv.)	TfOH (mol%)	Temp. (˚C)	Conc. of 4a (M)	Flow rate (mL/min)	Residence time (min)	NMR yield^b^ (%)
1	Comet X^c^	2.0	1.0	60	0.05	0.05	24	14
2	Comet X	3.0	0.5	40	0.01	0.2	6	35
3	β-type^d^	1.0	2.0	60	0.01	0.1	13.5	11
4	β-type	3.0	0.5	20	0.1	0.1	13.5	52
5	T-shaped^e^	1.0	2.0	40	0.05	0.05	16	4
6	T-shaped	2.0	1.0	20	0.1	0.2	4	29
7	Comet X	3.0	0.29	25	0.14	0.048	25	20
8	β-type	3.5	0.25	15	0.035	0.087	15.5	58
9	T-shaped	3.5	0.98	30	0.15	0.022	36	19
10	Comet X	3.4	0.37	35	0.056	0.13	9	10
11	β-type	2.4	0.34	15	0.019	0.041	33	40
12	T-shaped	2.4	0.73	15	0.011	0.060	13’	19
13	β-type	3.2	0.25	15	0.068	0.062	22	20
14	β-type	3.5	0.53	15	0.067	0.097	14	43
15	β-type	3.2	0.35	30	0.044	0.068	20	69 (66)^c^

^a^Reaction conditions: **4a** (0.065 mmol), **2j**, and TfOH, in degassed dry toluene/EtOAc (10/1).

^b^1,3,5-Trimethoxybenzene was used as a standard.

^c^Volume of Comet X microreactor = 2.4 mL.

^d^Volume of β-type microreactor = 2.7 mL.

^e^Volume of T-shaped microreactor = 1.6 mL.

^f^Isolated yield.

**Fig. 3:**
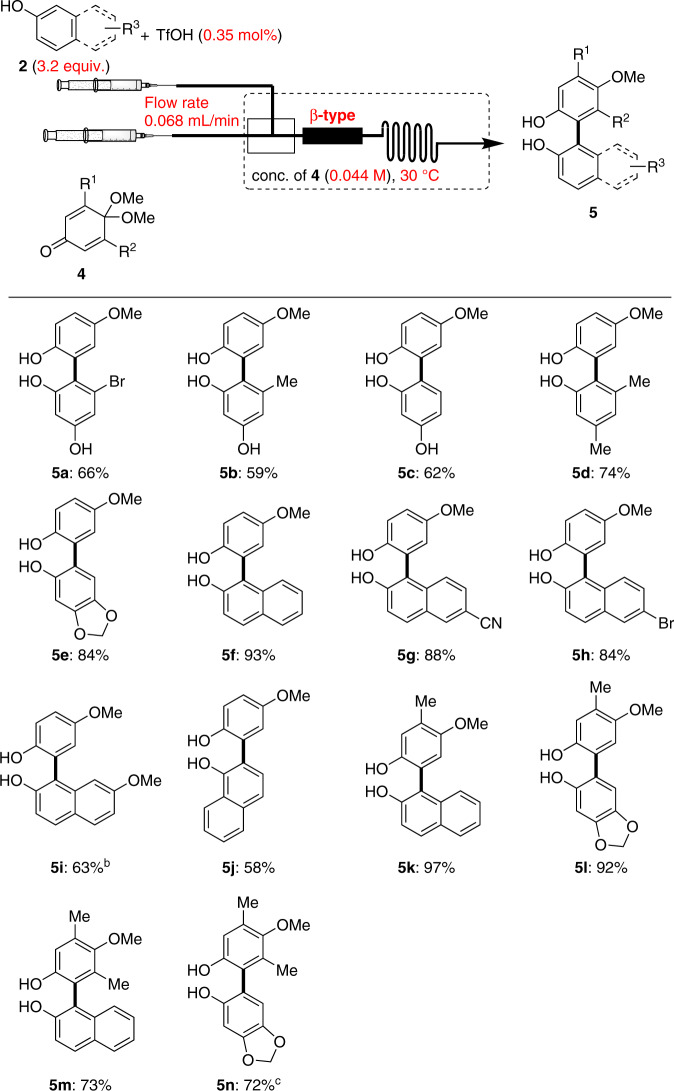
Substrate scope of cross-coupling using QMAs **4** and arenols **2**^a^. Yields are those of isolated **5**. ^a^Reaction conditions: **4** (0.065 mmol), **2** (3.2 equiv.), and TfOH (0.35 mol%) in degassed dry toluene/AcOEt (conc. of **4**, 0.044 M, 10/1), micro mixer: β-type, flow rate: 0.068 mL/min, at 30 °C. ^b^Flow rate: 0.055 mL/min, at 35 °C. ^c^TfOH (0.5 mol%), flow rate: 0.048 mL/min, at 35 °C.

The experimental dataset (Table 2, entries 1–6) was collected under the same reaction conditions listed in Table 1 (entries 1–6). Similarly, when BO with parallel LCB and experimental evaluation were repeatedly performed (entries 7–15), the yield of product **5a** was improved to 69% (isolated yield: 66%) with a use of β-type mixer conditions in entry 15. In the previous reaction (Table 1), different mixer (Comet-X), and lower concentration of IQMAs **1** was required compared to the optimized conditions (Table 2). When we tested this lower concentration using QMAs **4**, low conversion of **4** was observed, while testing IQMAs **1** under these new conditions generated many side products. Hence the difference in the mixer suitable for each reaction can be rationalized to be due to the difference in the respective stirring methods. Under the revised reaction conditions (Table 2, entry 15), we evaluated the scope of the cross-coupling reaction using QMAs **4** and arenols **2**, according to Fig. 3. Using acceptor **4a** with 5-methylresorcinol (**2k**) and non-substituted resorcinol (**2l**), the corresponding products **5b** and **5c**, were obtained in moderate yields (59 and 62%, respectively). Substrates such as 3,5-dimethylphenol (**2m**) and sesamol (**2n**) were successfully converted to **5d** and **5e** in 74 and 84% yields, respectively. When a variety of naphthols (**2a**: 2-naphthol; **2o**: 6-cyano-2-naphthol; **2p**: 6-bromo-2-naphthol; **2q**: 7-methoxy-2-naphtol; **2r**: 1-naphthol) reacted, the corresponding biarenols **5f**–**j** were produced with 58–93% yields. The structure of **5j** was further confirmed by X-ray crystallographic analysis (see Supplementary Note 6). In addition, the reaction of 3-methylated QMA **4b** with **2a** and **2n** afforded the corresponding products in high yields (**5k**: 97% and **5l**: 92%). Finally, we found that sterically hindered 3,5-dimethylated **4c** was tolerated in this transformation to give biarenols **5m** and **5n** in good yields (73 and 72%, respectively).

### Gram-scale synthesis and transformation of product

**Fig. 4:**
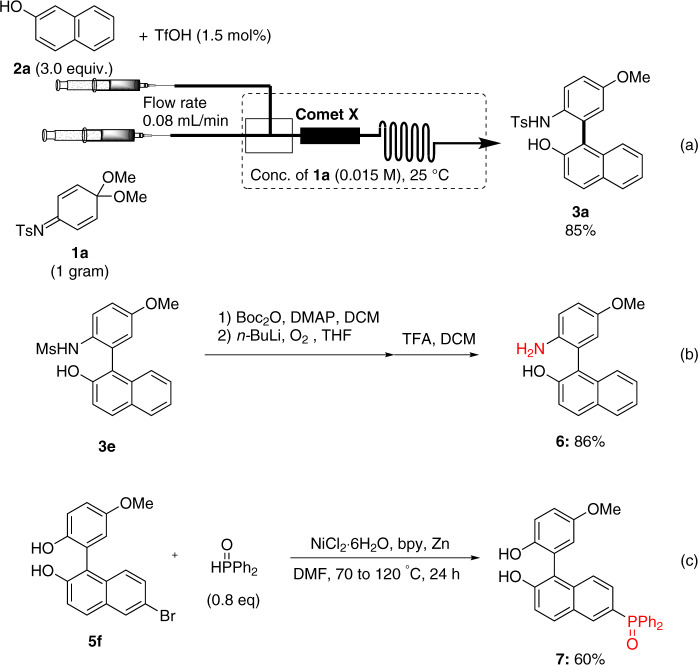
Gram-scale synthesis of **3a** and synthetic applications of **3e** and **5f**. **a** Gram-scale synthesis of **3a** under the optimized conditions. **b** Removal of Ms group. **c** Reductive coupling of **5f** with diphenyl phosphine oxide.

To demonstrate the utility of our reaction, we investigated the scalability of the reaction system. Using 1 gram of **1a** under BO-established suitable conditions, the desired biaryl **3a** was obtained with a yield of 85% (Fig. 4a). According to a previously reported procedure^51^, the methanesulfonyl group in **3e** was successfully removed, and the corresponding product **6** was obtained with 86% yield (Fig. 4b) (see Supplementary Note 2). Moreover, the Ni(II)/Zn-catalyzed reductive coupling of **5f** with diphenyl phosphine oxide provided the P-arylated product **7** with 60% yield (Fig. 4c)^54^.

### Plausible reaction mechanism

**Fig. 5:**
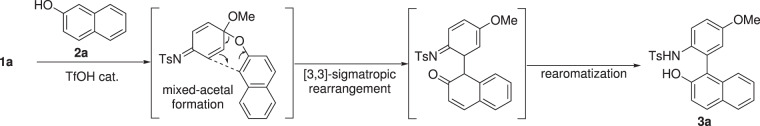
Plausible mechanism. TfOH promotes mixed-acetal formation from **1a** and **2a**. Then, [3,3]-sigmatropic rearrangement and rearomatization provide compound **3a**.

A plausible reaction mechanism for the Brønsted acid-catalyzed cross-coupling reaction may involves the formation of a mixed acetal/[3,3]-sigmatropic rearrangement (Fig. 5)^51^. Initially, the strong Brønsted acid, TfOH, is thought to promote the formation of a mixed acetal from **1a** and **2a**. Subsequently, the [3,3]-sigmatropic rearrangement of the mixed acetal and re-aromatization would provide the biarenol **3a**. According to Kürti’s report, we performed the coupling reaction of 2-methoxynaphthalene with **1a** (see Fig. S3 in Supplementary Note 5) and the desired coupling product was not obtained because the methyl-capped substrate could not form the mixed acetal intermediate under our optimized reaction conditions. This suggests that our flow reaction proceeded by a similar reaction intermediate with that of Kürti’s reaction^51^.

In conclusion, we demonstrated a highly efficient, rapid, and regioselective synthetic method for various biaryls utilizing the redox-neutral cross-coupling of iminoquinone monoacetals or quinone monoacetals with arenols in a flow system. The reaction proceeded smoothly at room temperature in an inexpensive and low toxicity toluene medium with 1 mol% TfOH. BO-assisted parallel screening with one-hot encoding successfully estimated suitable reaction conditions, including both numerical and categorical parameters. Our algorithm can screen for engineering variables such as the type of micromixer, providing a method for chemists that does not require complicated quantification or descriptors. The optimized conditions were successfully applied to gram-scale synthesis. To the best of our knowledge, this is the first report on the redox-free flow process for the synthesis of highly functionalized biaryls. Our group is currently investigating BO-assisted screening of multiple categorical parameters and the highly enantioselective synthesis of biaryls using an immobilized chiral catalyst in flow.

## Methods

### General methods

For synthetic details and analytical data of all reaction starting materials **1** and **4**, see Supplementary Methods 1 and 2. For synthetic and analytical details of all atropisomeric biaryls **3** and **5**, see Supplementary Methods 3 and 4. For NMR spectra see Supplementary Data 1.

### General procedure for the biaryl synthesis of IQMAs 1 and arenol 2 using TfOH in flow system

**Fig. 6:**
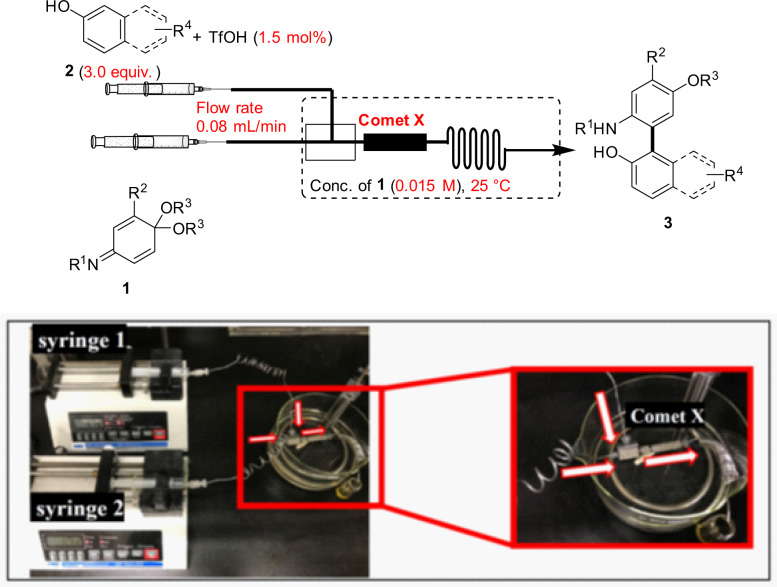
The flow system of the synthesis of atropisomeric biaryls **3**. The flow reactor used to synthesize atropisomeric biaryls **3** using a Comet-X mixer dipped in an oil bath at 25 °C and under 0.08 mL/min flow rate.

As shown in Fig. 6, a flow microreactor system was dipped in oil bath to heat at 25 °C. A solution of **1a** (0.065 mmol, 0.015 M) in toluene (2.2 mL, syringe 1), and a solution of **2a** (0.195 mmol, 0.045 M) and TfOH (1.5 mol%) in toluene (2.2 mL, syringe 2) were introduced to the flow microreactor system by syringe pumps at a flow rate: 0.08 mL/min (see Fig. S1 in Supplementary Method 3). The resulting solution was passed through Comet X mixer (total volume = 2.4 mL, residence time = 15 min) and directly forwarded to the quenching saturated aq. NaHCO_3_ solution. After all the amount of toluene solutions were pumped, we pumped a fresh air to the flow microreactor at the same flow rate (0.08 mL/min) to avoid losing 1 reactor volume inside. Finally, the organic layer was extracted with EtOAc (15 mL × 3), dried over Na_2_SO_4_, concentrated *in vacuo*. The residue was purified by silica column chromatography (*n*-hexane/EtOAc) to afford **3a** (93%, 25.4 mg).

## Supplementary information

Takizawa_PR File

Supplementary Information

Description of Additional Supplementary Files

Supplementary Data 1

Supplementary Data 2

Supplementary Data 3

## Data Availability

Additional data supporting the findings described in this manuscript are available in the Supplementary information and supplementary Data 1–3. The X-ray crystallographic coordinate for structures reported in this study have been deposited at the Cambridge Crystallographic Data Center (CCDC) under deposition numbers CCDC-2142538 (**5j**). These data can be obtained free of charge from The Cambridge Crystallographic Data Center via www.ccdc.cam.ac.uk/data_request/cif. The authors declare that all other data supporting the findings of this study are available within the article and Supplementary information and Data 1–3, and also are available from the corresponding author upon reasonable request.

## Code availability

Codes were deposited in a DOI-minting repository (Zenodo) under the title of ‘Scripts for categorical Bayesian optimization-assisted screening of reaction conditions in the flow biaryl synthesis’ 10.5281/zenodo.7151503.
