# Supplementary material for: Bayesian optimization-driven parallel-screening of multiple parameters for the flow synthesis of biaryl compounds
Source: Commun Chem. 2022 Nov 10;5:148. doi: 10.1038/s42004-022-00764-7 (PMC9814103; doi:10.1038/s42004-022-00764-7)
Supplement: Supplementary file 2 — Supplementary Information [file 42004_2022_764_MOESM2_ESM.pdf]

Supporting Information

Bayesian optimization-driven parallel-screening of multiple parameters for the flow synthesis of biaryl compounds

Masaru Kondo[1,2], H. D. P. Wathsala[2], Mohamed S. H. Salem[2,3], Kazunori Ishikawa[2], Satoshi Hara[2], Takayuki Takaai[2], Takashi Washio[2,4]*, Hiroaki Sasai[2,5] and Shinobu Takizawa[1,4]*

[1] *Department of Materials Science and Engineering, Graduate School of Science and Engineering, Ibaraki University, Naka-narusawa, Hitachi, Ibaraki 316-8511, Japan*

[2] *SANKEN, Osaka University, Mihogaoka, Ibaraki-shi, Osaka 567-0047, Japan*

[3] *Pharmaceutical Organic Chemistry Department, Faculty of Pharmacy, Suez Canal University, Ismailia 41522, Egypt*

[4] *Artificial Intelligence Research Center, SANKEN, Osaka University, Japan*

[5] *Graduate School of Pharmaceutical Sciences, Osaka University, Yamada-oka, Suita-shi, Osaka 565-0871, Japan*

Correspondence: washio@ar.sanken.osaka-u.ac.jp, taki@sanken.osaka-u.ac.jp

---

Table of Contents

1. **Supplementary Method 1:** ***general information*** **S2**
2. **Supplementary Method 2:** ***general procedure for the synthesis of starting materials 1 and 4*** **S2**
3. **Supplementary Method 3:** ***general procedure for the synthesis of atropoisomeric biaryls 3 in a flow system*** **S3**
4. **Supplementary Method 4:** ***general procedure for the synthesis of atropoisomeric biaryls 5 in a flow system*** **S10**
5. **Supplementary Note 1:** ***preliminary result for the asymmetric synthesis of atropoisomeric biaryl 3h*** **S14**
6. **Supplementary Note 2:** ***transformations of 3e and 5f*** **S15**
7. **Supplementary Note 3:** ***microreactor information*** **S16**
8. **Supplementary Note 4:** ***screening of microreactors*** **S18**
9. **Supplementary Note 5:** ***mechanistic study*** **S18**
10. **Supplementary Note 6:** ***X-Ray crystallographic analysis*** **S19**
11. **Supplementary References** **S22**

1. Supplementary Method 1: ***general information***

^{1}H-, and ^{13}C-NMR were recorded with JEOL JMN ECS 400 FT NMR, JEOL JMN ECS 600 FT NMR, or Bruker AVANCE II (^{1}H-NMR 400, or 600 MHz, ^{13}C-NMR 100, 150, or 175 MHz) ^{1}H-NMR spectra are reported as follows: a chemical shift in ppm downfield of tetramethylsilane (TMS) and referenced to residual solvent peak ($CDCl_3$) at 7.26 ppm, (CD_3OD) at 3.31, or ($(CD_3)_2CO$) at 2.05 ppm, integration, multiplicities (s = singlet, d = doublet, dd = doublet of doublets, t = triplet, q = quartet, m = multiplet), and coupling constants (Hz). ^{13}C-NMR spectra reported in ppm relative to the central line of triplet for $CDCl_3$ at 77.16 ppm, or the central line of septet for ($(CD_3)_2CO$) at 29.84 ppm. ESI-MS spectra were obtained with JMS-T100LC (JEOL). FT-IR spectra were recorded on JASCO FT-IR system (FT/IR4100). Thin-layer chromatography (TLC) analysis of reaction mixtures was performed using Merck silica gel 60 F254 TLC plates and visualized under UV. Column chromatography on SiO_2 was performed with Kanto Silica Gel 60 (63-210 μm). Comet-01-X[1] micromixer with inner diameter of 100 μm were manufactured by Techno Application. T-shaped and β-type[2] micromixers were manufactured by MiChS. These mixers are made of stainless steel. The flow microreactor system was dipped in a cooling water bath to control the temperature. Solutions were introduced to the flow microreactor system using syringe pumps, Harvard Model 11, equipped with gastight syringes purchased from YMC. Naphthalen-2-ol (**2a**) was purchased from KANTO CHEMICAL CO., INC. 6-Bromonaphthalen-2-ol (**2m**), 3,5-xylenol (**2d**), and sesamol (**2e**) were purchased from WAKO PURE CHEMICAL INDUSTRIES CO., LTD. 3-Hydroxy-2-naphthoic acid (**2p**), 5-methylbenzene-1,3-diol (**2b**), and 6-hydroxy-2-naphthonitrile (**2g**) were purchased from TOKYO CHEMICAL INDUSTRY CO., LTD. Resorcinol (**2c**), and naphthalen-1-ol (**2j**) were purchased from KISHIDA CHEMISTRY. Other starting materials were synthesized according to reported procedures: 6-(Benzyloxy)naphthalen-2-ol (**2l**)[3], 6-(4-bromophenyl)naphthalen-2-ol (**2n**)[4], 3-methoxynaphthalen-2-ol (**2o**)[5], 7-(allyloxy)naphthalen-2-ol (**2q**)[6], 7-((tert-butyldimethylsilyl)oxy)naphthalen-2-ol (**2r**)[7], 7-(4,4,5,5-tetramethyl-1,3,2-dioxaborolan-2-yl)naphthalen-2-ol (**2s**)[8], 5-bromobenzene-1,3-diol (**2t**)[9], and 7-methoxynaphthalen-2-ol (**2i**)[3]. The other simple chemicals and solvents were purchased from commercial suppliers and used without further purification.

2. Supplementary Method 2: ***general procedure for the synthesis of starting materials 1 and 4***

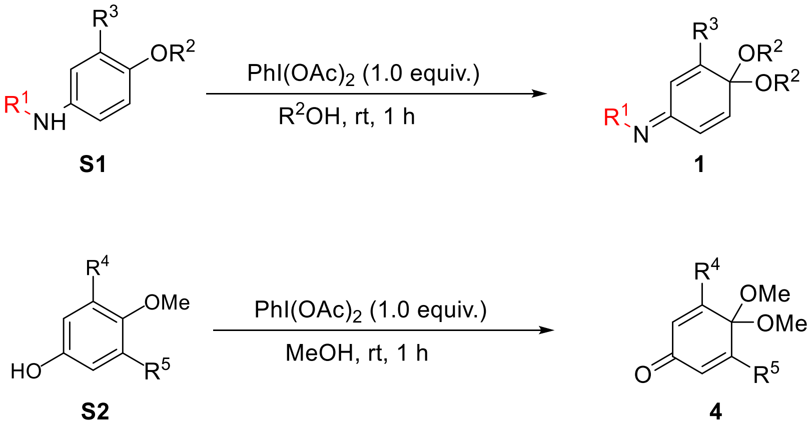

Starting materials **1** and **4** were synthesized according to the reported procedures.[10,11] **1a**[12], **1b**[11], **1c**[13], **1d**[13], **1e**[14], **1h**[13], **1i**[14], **1j**[13], **1k**[13], **4a**[15], **4k**[16], and **4m**[17] were known compounds: $PhI(OAc)_2$ (3.08 mmol) was added to a solution of **S1** or **S2** (3.08 mmol) in MeOH (15 mL) at 0 °C. After stirring at room temperature until its completion as monitored by TLC, saturated aq. $NaHCO_3$ solution was added to the reaction mixture. After extraction with EtOAc (50 mL × 3), the combined organic layers were collected, washed with brine (30 mL × 2), dried over Na_2SO_4, and evaporated *in vacuo*. The residue was purified by

silica column chromatography to afford corresponding **1** or **4**.

N-(4,4-Dimethoxycyclohexa-2,5-dien-1-ylidene)-2,4-dimethoxy-6-methylbenzenesulfonamide (**1f**)

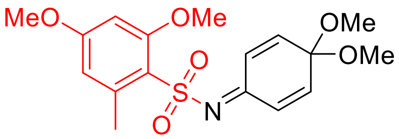

89% yield as a yellow solid.

^{1}H-NMR (400 MHz, $CDCl_3$) δ 7.72-7.75 (m, 2H), 6.66-6.73 (m, 2H), 6.44 (s, 1H), 6.38 (dd, J = 9.8, 2.1 Hz, 1H), 3.89 (s, 3H), 3.89 (s, 3H), 3.36 (s, 6H), 2.16 (s, 3H).

^{13}C-NMR (100 MHz, $CDCl_3$) δ 163.23, 157.60, 142.28, 141.54, 131.42, 131.14, 123.56, 118.90, 95.43, 92.45, 56.47, 55.87, 50.50, 15.39.

HRMS (ESI) calcd for $C_{17}H_{21}NO_6SNa$: *m/z* ([M+Na$^+$]) 390.0982, found 390.0983.

IR (KBr) 2943, 2839, 1611, 1550, 1464, 1290, 1133, 1030, 657, 557 cm^{-1}.

Methyl (4,4-dimethoxycyclohexa-2,5-dien-1-ylidene)carbamate (**1g**)

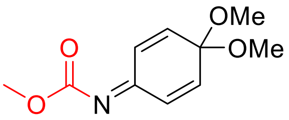

76% yield as a brown solid.

^{1}H-NMR (400 MHz, $CDCl_3$) δ 6.54 (d, J = 8.2 Hz, 2H), 6.40 (d, J = 8.2 Hz, 2H), 3.83 (s, 3H), 3.30 (s, 6H).

^{13}C-NMR (100 MHz, $CDCl_3$) δ 162.83, 157.87, 140.24, 139.65, 130.44, 123.34, 93.06, 53.67, 50.31.

HRMS (ESI) calcd for $C_{10}H_{13}NO_4Na$: *m/z* ([M+Na$^+$]) 234.0737, found 234.0736.

IR (KBr) 2943, 2833, 1720, 1605, 1433, 1238, 1113, 1036, 964, 752 cm^{-1}.

3. Supplementary Method 3: *general procedure for the synthesis of atropoisomeric biaryls 3 in a flow system*

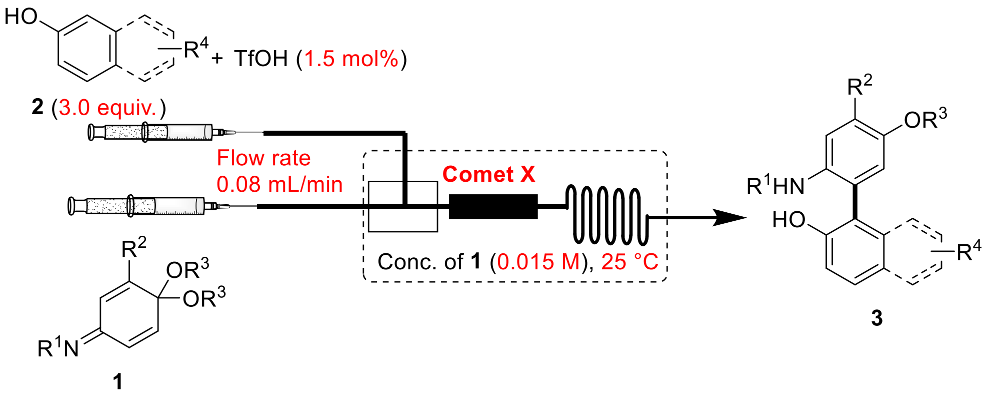

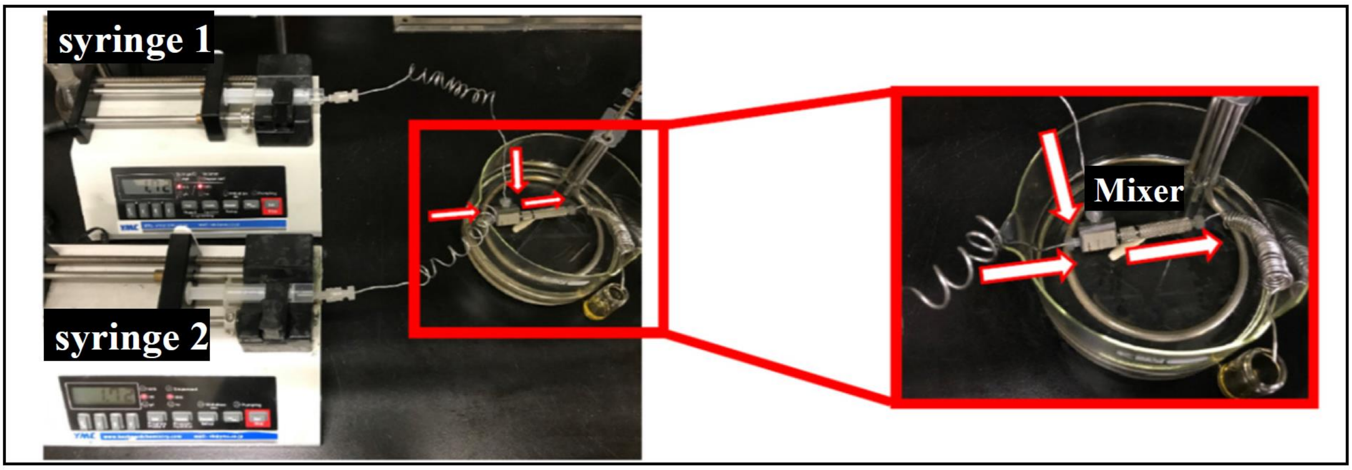

Fig. S1. The flow system of the synthesis of atropoisomeric biaryls **3**.

As shown in Fig. S1, a flow microreactor system was dipped in oil bath to heat at 25 ˚C. A solution of **1** (0.065 mmol) in toluene (2.2 mL, syringe 1), and a solution of **2** (0.195 mmol, 3.0 equiv.) and TfOH (1.5 mol%) in toluene (2.2 mL, syringe 2) were introduced to the flow microreactor system with Comet X mixer by syringe pumps (flow rate: 0.08 mL/min). After the continuous-flow was kept within residence time, the reaction mixture was collected and quenched with aq. $NaHCO_3$. The organic layer was extracted with EtOAc (15 mL × 3), dried over Na_2SO_4, concentrated *in vacuo*. The residue was purified by silica column chromatography (*n*-hexane/EtOAc) to afford **3**.

N-(2-(2-Hydroxynaphthalen-1-yl)-4-methoxyphenyl)-4-methylbenzenesulfonamide (**3a**)

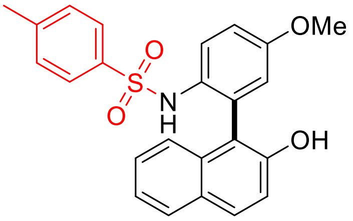

93% yield (25.4 mg) as a white solid.

^{1}H-NMR (400 MHz, $CDCl_3$) δ 7.77-7.81 (m, 3H), 7.32 (ddd, J = 7.8, 7.3, 0.9 Hz, 1H), 7.15-7.23 (m, 4H), 7.02 (dd, J = 8.9, 3.2 Hz, 1H), 6.88-6.91 (m, 3H), 6.72 (d, J = 3.2 Hz, 1H), 6.41 (s, 1H), 5.24 (s, 1H), 3.76 (s, 3H), 2.26 (s, 3H).

^{13}C-NMR (100 MHz, $CDCl_3$) δ 157.72, 150.50, 143.64, 135.90, 132.79, 130.86, 129.48, 129.15, 128.74, 128.39, 128.35, 127.32, 127.01, 125.75, 123.93, 123.77, 117.73, 117.06, 115.87, 115.57, 55.67, 21.64.

HRMS (ESI) calcd for $C_{24}H_{21}NO_4SNa$: *m/z* ([M+Na$^+$]) 442.1084, found 442.1079.

IR (KBr) 3495, 3266, 3014, 2975, 2363, 1609, 1491, 1384, 1288, 810 cm^{-1}.

N-(2-(2-Hydroxynaphthalen-1-yl)-4-methoxyphenyl)-4-methoxybenzenesulfonamide **(3b)**

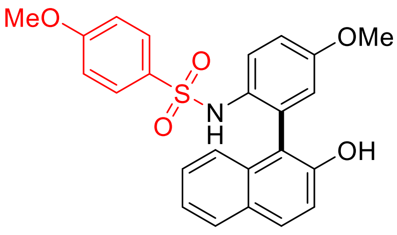

93% yield (25.0 mg) as a white solid.

^{1}H-NMR (400 MHz, $CDCl_3$) δ 7.78-7.83 (m, 3H), 7.28-7.35 (m, 3H), 7.21 (ddd, J = 7.8, 7.6, 1.2 Hz, 1H), 7.16 (d, J = 9.2 Hz, 1H), 7.03 (dd, J = 9.2, 3.2 Hz, 1H), 6.90 (d, J = 8.2 Hz, 1H), 6.72 (d, J = 3.2 Hz, 1H), 6.62 (dd, J = 6.9, 1.8 Hz, 2H), 6.23 (s, 1H), 4.85 (s, 1H), 3.79 (s, 3H), 3.77 (s, 3H).

^{13}C-NMR (100 MHz, $CDCl_3$) δ 163.03, 157.72, 150.54, 132.79, 130.90, 130.49, 129.15, 128.91, 128.33, 128.11, 127.42, 125.61, 123.86, 117.74, 117.01, 115.79, 115.67, 114.06, 55.68, 55.56 (Two carbons overlapped).

HRMS (ESI) calcd for $C_{24}H_{21}NO_5SNa$: *m/z* ([M+Na$^+$]) 458.1033, found 458.1029.

IR (KBr) 3474, 3354, 3249, 2964, 2833, 1594, 1496, 1330, 1153, 817 cm^{-1}.

4-Chloro-*N*-(2-(2-hydroxynaphthalen-1-yl)-4-methoxyphenyl)benzenesulfonamide (**3c**)

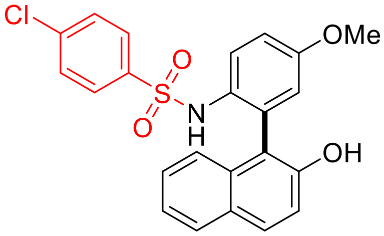

86% yield (23.0 mg) as a white solid.

^{1}H-NMR (400 MHz, $CDCl_3$) δ 7.82 (d, J = 6.9 Hz, 1H), 7.78-7.81 (m, 2H), 7.36 (ddd, J = 7.6, 7.3, 1.2 Hz, 1H), 7.17-7.24 (m, 3H), 7.15 (d, J = 8.7 Hz, 1H), 7.05 (dd, J = 8.9, 3.0 Hz, 1H), 7.00-7.02 (m, 2H), 6.85 (d, J = 8.2 Hz, 1H), 6.74 (d, J = 2.7 Hz, 1H), 6.37 (s, 1H), 4.90 (s, 1H), 3.78 (s, 3H).

^{13}C-NMR (100 MHz, $CDCl_3$) δ 158.15, 150.17, 139.33, 137.40, 132.72, 130.91, 129.21, 129.13, 129.06, 128.39, 128.26, 128.02, 127.60, 126.85, 124.08, 123.67, 117.57, 117.18, 116.00, 115.60, 55.70.

HRMS (ESI) calcd for $C_{23}H_{18}ClNO_4SNa$: *m/z* ([M+Na$^+$]) 462.0537, found 462.0541.

IR (KBr) 3491, 3249, 3085, 2941, 2837, 1613, 1490, 1331, 1163, 814 cm$^{-1.}$

N-(2-(2-Hydroxynaphthalen-1-yl)-4-methoxyphenyl)-4-nitrobenzenesulfonamide (**3d**)

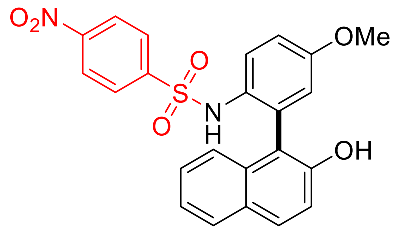

91% yield (24.3 mg) as a yellow solid.

^{1}H-NMR (400 MHz, CDCl$_3$) δ 7.79 (d, J = 8.7 Hz, 2H), 7.70 (d, J = 8.2 Hz, 1H), 7.65 (d, J = 8.7 Hz, 2H), 7.29 (d, J = 8.7 Hz, 2H), 7.23 (t, J = 7.6 Hz, 1H), 7.11-7.14 (m, 2H), 7.08 (dd, J = 8.9, 3.0 Hz, 1H), 6.90 (d, J = 8.7 Hz, 1H), 6.74-6.76 (m, 2H), 5.10 (s, 1H), 3.79 (s, 3H).

^{13}C-NMR (100 MHz, CDCl$_3$) δ 158.64, 149.25, 149.08, 144.49, 132.60, 131.43, 130.83, 129.88, 129.12, 128.27, 127.37, 127.16, 126.68, 124.12, 124.03, 123.64, 117.53, 117.42, 116.89, 115.26, 55.72.

HRMS (ESI) calcd for $C_{23}H_{18}N_2O_6SNa$: *m/z* ([M+Na$^+$]) 473.0778, found 473.0079.

IR (KBr) 3545, 3392, 3321, 3101, 1606, 1525, 1343, 1162, 856, 740 cm^{-1}.

N-(2-(2-Hydroxynaphthalen-1-yl)-4-methoxyphenyl)methanesulfonamide (**3e**)

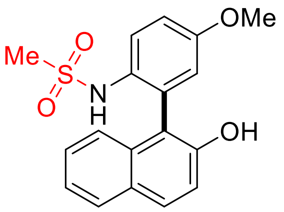

92% yield (30.0 mg) as a white solid.

^{1}H-NMR (400 MHz, CDCl$_3$) δ 7.85-7.90 (m, 2H), 7.75 (d, J = 9.2 Hz, 1H), 7.37-7.43 (m, 2H), 7.25-7.28 (m, 2H), 7.09 (dd, J = 9.2, 3.2 Hz, 1H), 6.89 (d, J = 3.2 Hz, 1H), 5.92 (s, 1H), 5.24 (s, 1H), 3.83 (s, 3H), 2.59 (s, 3H).

^{13}C-NMR (100 MHz, CDCl$_3$) δ 158.25, 150.68, 132.84, 131.15, 129.29, 129.09, 128.78, 127.81, 126.25, 124.26, 123.65, 117.94, 117.21, 116.09, 115.69, 55.76, 39.49 (One carbon overlapped).

HRMS (ESI) calcd for $C_{18}H_{17}NO_4SNa$: *m/z* ([M+Na$^+$]) 366.0771, found 366.0770.

IR (KBr) 3386, 3307, 3006, 2925, 2838, 1615, 1494, 1305, 1150, 821 cm^{-1}.

N-(2-(2-Hydroxynaphthalen-1-yl)-4-methoxyphenyl)-2,4-dimethoxy-6-methylbenzenesulfonamide (**3f**)

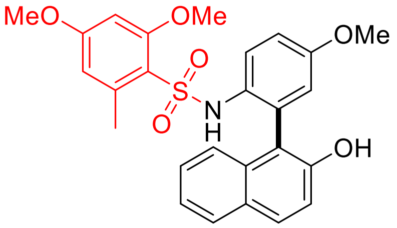

81% yield (25.0 mg) as a white solid.

^{1}H-NMR (400 MHz, CDCl$_3$) δ 7.82 (d, J = 3.7 Hz, 1H), 7.80 (d, J = 3.7 Hz, 1H), 7.76 (d, J = 8.2 Hz, 1H), 7.35 (d, J = 8.5 Hz, 1H), 7.31 (s, 1H), 7.27 (ddd, J = 7.8, 7.3, 0.9 Hz, 1H), 7.14 (ddd, J = 7.8, 7.6, 1.15 Hz, 1H), 6.99-7.02 (m, 2H), 6.94 (d, J = 8.7 Hz, 1H), 6.62 (d, J = 2.7 Hz, 1H), 6.01 (s, 1H), 5.60 (s, 1H), 3.73 (s, 3H), 3.67 (s, 3H), 3.18 (s, 3H), 1.99 (s, 3H).

^{13}C-NMR (100 MHz, CDCl$_3$) δ 162.33, 157.51, 156.11, 150.68, 133.46, 131.07, 130.23, 129.38, 128.97, 128.61, 127.61, 127.09, 126.64, 124.59, 123.58, 117.89, 117.72, 116.87, 116.51, 115.46, 94.00, 55.62, 55.60, 55.38, 15.31 (One carbon overlapped).

HRMS (ESI) calcd for $C_{26}H_{25}NO_6SNa$: *m/z* ([M+Na$^+$]) 502.1295, found 502.1290.

IR (KBr) 3413, 3304, 2948, 1606, 1495, 1433, 1320, 1287, 1138, 817 cm^{-1}.

Methyl (2-(2-hydroxynaphthalen-1-yl)-4-methoxyphenyl)carbamate (**3g**)

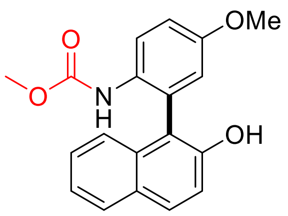

76% yield (16.0 mg) as a white solid.

^{1}H-NMR (400 MHz, $CDCl_3$) *δ* 8.13 (s, 1H), 7.82-7.88 (m, 2H), 7.36-7.39 (m, 2H), 7.29 (d, *J* = 8.7 Hz, 1H), 7.23-7.26 (m, 1H), 7.09 (dd, *J* = 9.2, 3.2 Hz, 1H), 6.81 (d, *J* = 3.2 Hz, 1H), 6.10 (s, 1H), 5.21 (s, 1H), 3.81 (s, 3H), 3.59 (s, 3H).

^{13}C-NMR (150 MHz, $CDCl_3$) *δ* 154.54, 151.18, 132.89, 130.86, 130.60, 129.20, 128.44, 127.42, 124.13, 123.98, 117.93, 116.57, 115.86, 115.81, 115.78, 55.73, 52.37 (Two carbons overlapped).

HRMS (ESI) calcd for $C_{19}H_{17}NO_4Na$: *m/z* ([M+Na$^+$]) 346.1050, found 346.1051.

IR (KBr) 3412, 3308, 3060, 2958, 2844, 2375, 1713, 1517, 1211, 820 cm^{-1}.

N-(2-(2-Hydroxynaphthalen-1-yl)-4-methoxy-5-methylphenyl)-4-methylbenzenesulfonamide (**3h**)

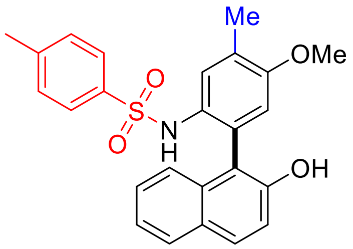

95% yield (26.9 mg) as a white solid.

^{1}H-NMR (400 MHz, $CDCl_3$) *δ* 7.81 (t, *J* = 8.2 Hz, 2H), 7.66 (s, 1H), 7.33 (t, *J* = 7.3 Hz, 1H), 7.25-7.27 (m, 2H), 7.21 (t, *J* = 7.8 Hz, 1H), 7.15 (d, *J* = 8.7 Hz, 1H), 6.97 (d, *J* = 7.8 Hz, 2H), 6.91 (d, *J* = 8.7 Hz, 1H), 6.58 (s, 1H), 6.19 (s, 1H), 4.76 (s, 1H), 3.72 (s, 3H), 2.31 (s, 6H).

^{13}C-NMR (100 MHz, $CDCl_3$) *δ* 156.00, 150.60, 143.57, 135.89, 132.93, 130.71, 129.46, 129.11, 128.79, 128.34, 128.18, 127.25, 127.00, 126.72, 124.84, 123.98, 123.71, 117.63, 116.05, 112.91, 55.69, 21.66, 16.50.

HRMS (ESI) calcd for $C_{25}H_{23}NO_4SNa$: *m/z* ([M+Na$^+$]) 456.1240, found 456.1237.

IR (KBr) 3401, 3290, 3066, 2980, 1627, 1504, 1376, 1318, 1155, 807 cm^{-1}.

N-(2-(2-Hydroxynaphthalen-1-yl)-4,5-dimethoxyphenyl)-4-methylbenzenesulfonamide (**3i**)

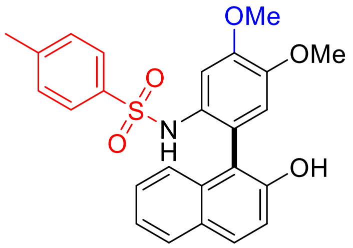

96% yield (28.1 mg) as a white solid.

^{1}H-NMR (400 MHz, $CDCl_3$) *δ* 7.81 (t, *J* = 8.7 Hz, 2H), 7.47 (s, 1H), 7.33 (ddd, *J* = 7.6, 7.3, 1.2 Hz, 1H), 7.28 (d, *J* = 8.2 Hz, 2H), 7.20 (ddd, *J* = 7.8, 7.3, 1.2 Hz, 1H), 7.16 (d, *J* = 9.2, 1H), 6.98 (d, *J* = 7.8 Hz, 2H), 6.88 (d, *J* = 8.2 Hz, 1H), 6.62 (s, 1H), 6.27 (s, 1H), 4.77 (s, 1H), 4.00 (s, 3H), 3.77 (s, 3H), 2.32 (s, 3H).

^{13}C-NMR (100 MHz, $CDCl_3$) *δ* 150.89, 149.84, 147.17, 143.88, 135.68, 133.11, 130.84, 129.56, 129.19, 129.15, 128.39, 127.33, 127.08, 123.88, 123.77, 117.59, 117.45, 115.38, 113.97, 107.27, 56.32, 56.19, 21.68.

HRMS (ESI) calcd for $C_{25}H_{23}NO_5SNa$: *m/z* ([M+Na$^+$]) 472.1189, found 472.1188.

IR (KBr) 3403, 3348, 2969, 2844, 1512, 1344, 1202, 1158, 1091, 812 cm^{-1}.

N-(2-Hydroxy-4'-methoxy-[1,2'-binaphthalen]-1'-yl)-4-methylbenzenesulfonamide (**3j**)

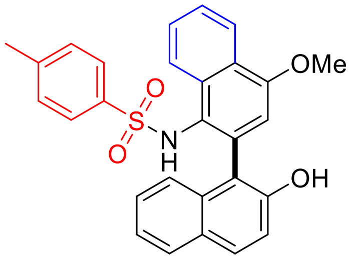

77% yield (23.5 mg) as a white solid.

^{1}H-NMR (400 MHz, $CDCl_3$) *δ* 8.41 (d, *J* = 7.8 Hz, 1H), 8.32 (dd, *J* = 6.9, 1.4 Hz, 1H), 7.70-7.74 (m, 2H), 7.57-7.64 (m, 2H), 7.31 (ddd, *J* = 7.6, 7.3, 1.2 Hz, 1H), 7.22-7.25 (m, 1H), 7.17 (d, *J* = 8.7 Hz, 1H), 7.11 (d, *J* = 9.2 Hz, 1H), 7.04 (d, *J* = 8.2 Hz, 2H), 6.68 (s, 1H), 6.65 (s, 2H), 6.63 (s, 1H), 5.63 (s, 1H), 3.92 (s, 3H), 2.15 (s, 3H).

^{13}C-NMR (150 MHz, $CDCl_3$) *δ* 155.46, 149.78, 142.58, 136.89, 133.88, 132.74, 131.45, 130.19, 129.42, 129.06, 128.27, 127.64, 126.97, 126.48, 126.37, 125.51, 124.68, 124.11, 123.65, 122.08, 119.49, 118.09,

107.20, 55.95, 21.60 (One carbon overlapped).

HRMS (ESI) calcd for $C_{28}H_{23}NO_4SNa$: *m/z* ([M+Na$^+$]) 492.1240, found 492.1248.

IR (KBr) 3436, 3276, 3003, 2928, 1627, 1596, 1510, 1311, 1145, 817 cm^{-1}.

N-(4-Ethoxy-2-(2-hydroxynaphthalen-1-yl)phenyl)-4-methylbenzenesulfonamide (**3k**)

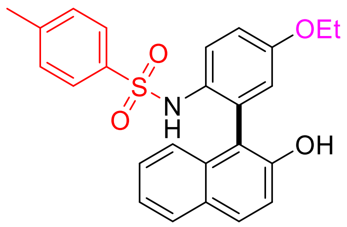

71% yield (20.0 mg) as a white solid.

^{1}H-NMR (400 MHz, $CDCl_3$) δ 7.77-7.83 (m, 3H), 7.33 (ddd, J = 8.2, 7.3, 1.2 Hz, 1H), 7.24-7.26 (m, 2H), 7.21 (ddd, J = 8.0, 7.3, 1.2 Hz, 1H), 7.15 (d, J = 8.7 Hz, 1H), 7.02 (dd, J = 9.2, 3.0 Hz, 1H), 6.97 (d, J = 8.2 Hz, 2H), 6.89 (d, J = 8.7 Hz, 1H), 6.70 (d, J = 3.0 Hz, 1H), 6.20 (s, 1H), 4.69 (s, 1H), 3.97 (qd, J = 6.9, 1.1 Hz, 2H), 2.31 (s, 3H), 1.38 (t, J = 6.9 Hz, 3H).

^{13}C-NMR (100 MHz, $CDCl_3$) δ 157.14, 150.58, 143.68, 135.93, 132.73, 130.89, 129.52, 129.14, 128.68, 128.40, 127.86, 127.41, 127.07, 125.46, 123.85, 123.82, 117.72, 117.45, 116.23, 115.74, 63.95, 21.67, 14.90.

HRMS (ESI) calcd for $C_{25}H_{23}NO_4SNa$: *m/z* ([M+Na$^+$]) 456.1240, found 456.1239.

IR (KBr) 3469, 3257, 2980, 2925, 1608, 1494, 1386, 1328, 1162, 935 cm^{-1}.

N-(2-(6-(Benzyloxy)-2-hydroxynaphthalen-1-yl)-4-methoxyphenyl)-4-methylbenzenesulfonamide (**3l**)

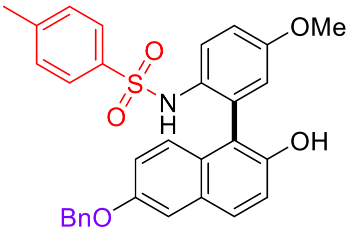

85% yield (29.0 mg) as a yellow solid.

^{1}H-NMR (400 MHz, $CDCl_3$) δ 7.76 (d, J = 9.2 Hz, 1H), 7.67-7.73 (m, 2H), 7.29-7.37 (m, 5H), 7.21 (d, J = 8.7 Hz, 2H), 7.02-7.08 (m, 2H), 6.98 (d, J = 8.7 Hz, 1H), 6.90 (d, J = 7.8 Hz, 2H), 6.66 (d, J = 3.2 Hz, 1H), 6.26 (d, J = 2.3 Hz, 1H), 6.24 (s, 1H), 4.82 (d, J = 4.1 Hz, 2H), 4.79 (s, 1H), 3.74 (s, 3H), 2.26 (s, 3H).

^{13}C-NMR (100 MHz, $CDCl_3$) δ 157.91, 157.87, 151.08, 143.69, 136.71, 136.05, 134.15, 130.51, 129.99, 129.41, 128.76, 128.65, 128.57, 128.20, 127.68, 127.04, 126.14, 124.56, 116.65, 116.53, 115.74, 115.15, 104.25, 69.90, 55.67, 21.58 (One carbon overlapped).

HRMS (ESI) calcd for $C_{31}H_{27}NO_5SNa$: *m/z* ([M+Na$^+$]) 548.1502, found 548.1501.

IR (KBr) 3413, 3304, 3035, 2917, 2362, 1623, 1377, 1320, 1151, 838 cm^{-1}.

N-(2-(6-Bromo-2-hydroxynaphthalen-1-yl)-4-methoxyphenyl)-4-methylbenzenesulfonamide (**3m**)

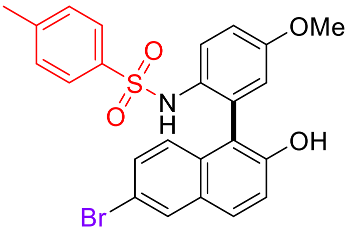

74% yield (24.0 mg) as a white solid.

^{1}H-NMR (400 MHz, $CDCl_3$) δ 7.92 (d, J = 2.3 Hz, 1H), 7.79 (d, J = 9.2 Hz, 1H), 7.72 (d, J = 9.2 Hz, 1H), 7.16-7.20 (m, 4H), 7.04 (dd, J = 8.9, 3.2 Hz, 1H), 6.88 (d, J = 8.2 Hz, 2H), 6.70 (d, J = 9.2 Hz, 1H), 6.67 (d, J = 3.2 Hz, 1H), 6.30 (s, 1H), 5.15 (s, 1H), 3.77 (s, 3H), 2.30 (s, 3H).

^{13}C-NMR (100 MHz, $CDCl_3$) δ 157.87, 150.60, 143.84, 136.00, 131.52, 130.36, 130.25, 130.11, 129.85, 129.41, 128.58, 128.11, 126.91, 126.44, 125.86, 118.85, 117.59, 117.06, 116.44, 115.77, 55.71, 21.71.

HRMS (ESI) calcd for $C_{24}H_{20}BrNO_4SNa$: *m/z* ([M+Na$^+$]) 520.0189, found 520.0187.

IR (KBr) 3382, 3312, 3080, 2938, 2840, 1601, 1492, 1335, 1156, 807 cm^{-1}.

N-(2-(6-(4-Bromophenyl)-2-hydroxynaphthalen-1-yl)-4-methoxyphenyl)-4-methylbenzenesulfonamide (**3n**)

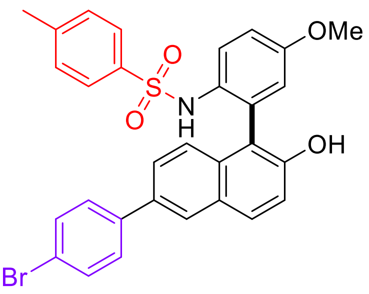

76% yield (29.0 mg) as a white solid.

¹H-NMR (400 MHz, $CDCl_3$) δ 7.89 (d, J = 8.2 Hz, 1H), 7.86 (d, J = 9.2 Hz, 1H), 7.75 (d, J = 9.2 Hz, 1H), 7.57 (dd, J = 8.2, 1.8 Hz, 1H), 7.54 (d, J = 8.7 Hz, 2H), 7.33 (d, J = 8.7 Hz, 2H), 7.26 (d, J = 8.2 Hz, 2H), 7.19 (d, J = 9.2 Hz, 1H), 7.12 (s, 1H), 7.03 (dd, J = 8.9, 3.2 Hz, 1H), 6.81 (d, J = 8.2 Hz, 2H), 6.74 (d, J = 3.2 Hz, 1H), 6.34 (s, 1H), 4.94 (s, 1H), 3.77 (s, 3H), 2.15 (s, 3H).

¹³C-NMR (100 MHz, $CDCl_3$) δ 157.68, 151.25, 143.76, 139.79, 138.74, 135.86, 132.98, 132.11, 130.74, 129.45, 129.32, 129.05, 128.93, 128.54, 127.29, 127.08, 125.08, 123.28, 122.04, 121.51, 118.08, 116.98, 115.98, 115.88, 55.72, 21.57.

HRMS (ESI) calcd for $C_{30}H_{24}BrNO_4SNa$: *m/z* ([M+Na$^+$]) 596.0502, found 596.0502.

IR (KBr) 3407, 3326, 3050, 1622, 1489, 1328, 1206, 1159, 1091, 823 cm^{-1}.

N-(2-(2-Hydroxy-3-methoxynaphthalen-1-yl)-4-methoxyphenyl)-4-methylbenzenesulfonamide (**3o**)

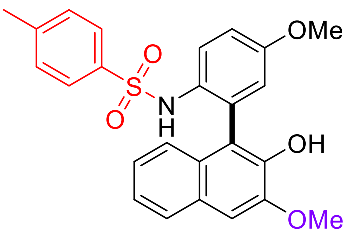

68% yield (19.8 mg) as a white solid.

¹H-NMR (400 MHz, $CDCl_3$) δ 7.77 (d, J = 8.7 Hz, 1H), 7.65 (d, J = 8.2 Hz, 1H), 7.27-7.30 (m, 1H), 7.16 (s, 1H), 7.05 (d, J = 8.2 Hz, 2H), 6.98-7.02 (m, 2H), 6.83 (d, J = 8.2 Hz, 1H), 6.69 (d, J = 2.7 Hz, 1H), 6.67 (s, 1H), 6.62 (d, J = 7.8 Hz, 2H), 6.11 (s, 1H), 4.10 (s, 3H), 3.75 (s, 3H), 2.12 (s, 3H).

¹³C-NMR (150 MHz, $CDCl_3$) δ 157.47, 146.28, 142.82, 141.80, 136.05, 130.58, 129.14, 129.06, 128.30, 127.87, 127.54, 126.81, 126.75, 124.71, 124.63, 124.14, 117.05, 116.91, 114.72, 106.30, 56.23, 55.60, 21.57.

HRMS (ESI) calcd for $C_{25}H_{23}NO_5SNa$: *m/z* ([M+Na$^+$]) 472.1189, found 472.1183.

IR (KBr) 3423, 3358, 3072, 2363, 2310, 1608, 1492, 1387, 1151, 868 cm^{-1}.

3-Hydroxy-4-(5-methoxy-2-((4-methylphenyl)sulfonamido)phenyl)-2-naphthoic acid (**3p**)

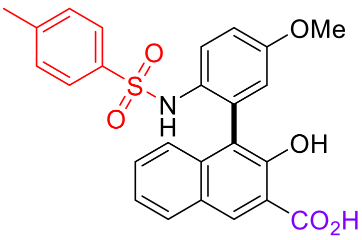

83% yield (25.0 mg) as a pale yellow solid.

¹H-NMR (400 MHz, $CDCl_3$) δ 11.02 (s, 1H), 8.61 (s, 1H), 7.87 (dd, J = 7.3, 1.4 Hz, 1H), 7.62 (d, J = 8.2 Hz, 2H), 7.37-7.45 (m, 2H), 7.22-7.27 (m, 3H), 7.12 (dd, J = 8.7, 2.3 Hz, 1H), 7.01 (d, J = 8.7 Hz, 1H), 6.90 (d, J = 2.3 Hz, 1H), 6.26 (s, 1H), 5.13 (s, 1H), 4.07 (s, 3H), 2.39 (s, 3H).

¹³C-NMR (150 MHz, $CDCl_3$) δ 170.52, 153.38, 152.68, 143.76, 136.74, 136.08, 133.76, 130.06, 129.99, 129.74, 129.04, 128.06, 127.58, 127.52, 126.00, 124.58, 122.62, 117.66, 116.80, 113.97, 53.11, 21.72 (One carbon overlapped).

HRMS (ESI) calcd for $C_{25}H_{21}NO_6SNa$: *m/z* ([M+Na$^+$]) 486.0982, found 486.0980.

IR (KBr) 3518, 3419, 3249, 2948, 2373, 1683, 1449, 1322, 1158, 809 cm^{-1}.

N-(2-(7-(Allyloxy)-2-hydroxynaphthalen-1-yl)-4-methoxyphenyl)-4-methylbenzenesulfonamide (**3q**)

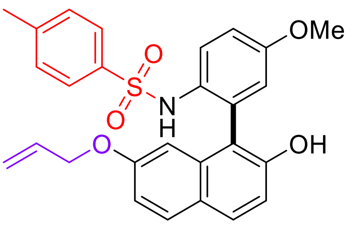

80% yield (24.7 mg) as a white solid.

^{1}H-NMR (400 MHz, $CDCl_3$) δ 7.79 (d, *J* = 8.7 Hz, 1H), 7.73 (d, *J* = 8.7 Hz, 1H), 7.70 (d, *J* = 8.7 Hz, 1H), 7.21 (d, *J* = 8.2 Hz, 2H), 6.98-7.04 (m, 3H), 6.91 (d, *J* = 8.2 Hz, 2H), 6.71 (d, *J* = 2.7 Hz, 1H), 6.31 (s, 1H), 6.20 (d, *J* = 2.7 Hz, 1H), 5.96 (dq, *J* = 17.3, 5.5 Hz, 1H), 5.24-5.34 (m, 2H), 4.70 (s, 1H), 4.28 (d, *J* = 5.5 Hz, 2H), 3.77 (s, 3H), 2.29 (s, 3H).

^{13}C-NMR (100 MHz, $CDCl_3$) δ 157.81, 151.01, 143.73, 135.93, 134.19, 133.07, 130.55, 129.95, 129.38, 128.60, 128.48, 127.04, 126.10, 126.02, 124.55, 118.21, 116.73, 116.39, 115.71, 115.07, 103.95, 68.67, 55.67, 21.58 (One carbon overlapped).

HRMS (ESI) calcd for $C_{27}H_{25}NO_5SNa$: *m/z* ([M+Na$^+$]) 498.1346, found 498.1344.

IR (KBr) 3567, 3485, 3276, 3151, 1622, 1513, 1291, 1215, 1093, 838 cm^{-1}.

N-(2-(7-((tert-Butyldimethylsilyl)oxy)-2-hydroxynaphthalen-1-yl)-4-methoxyphenyl)-4-methylbenzenesulfonamide (**3r**)

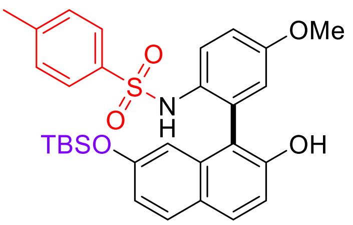

68% yield (24.3 mg) as an orange solid.

^{1}H-NMR (400 MHz, $CDCl_3$) δ 7.77 (d, *J* = 9.2 Hz, 1H), 7.72 (d, *J* = 8.7 Hz, 1H), 7.65 (d, *J* = 8.7 Hz, 1H), 7.20 (d, *J* = 8.2 Hz, 2H), 7.02 (dd, *J* = 8.9, 3.0 Hz, 1H), 6.98 (d, *J* = 9.2 Hz, 1H), 6.87-6.91 (m, 3H), 6.70 (d, *J* = 2.7 Hz, 1H), 6.37 (s, 1H), 6.24 (d, *J* = 2.3 Hz, 1H), 4.92 (s, 1H), 3.76 (s, 3H), 2.27 (s, 3H), 0.93 (s, 9H), 0.094 (s, 6H).

^{13}C-NMR (150 MHz, $CDCl_3$) δ 157.79, 155.02, 150.73, 143.75, 135.82, 134.31, 130.50, 129.83, 129.42, 128.63, 126.93, 126.13, 124.76, 119.87, 116.69, 115.76, 115.28, 114.77, 111.38, 55.64, 25.82, 21.62, 18.39, -4.17, -4.37 (One carbon overlapped).

HRMS (ESI) calcd for $C_{30}H_{35}NO_5SSiNa$: *m/z* ([M+Na$^+$]) 572.1897, found 572.1893.

IR (KBr) 3413, 3348, 2955, 2929, 2857, 1624, 1509, 1331, 1160, 838 cm^{-1}.

N-(2-(2-Hydroxy-7-(4,4,5,5-tetramethyl-1,3,2-dioxaborolan-2-yl)naphthalen-1-yl)-4-methoxyphenyl)-4-methylbenzene sulfonamide (**3s**)

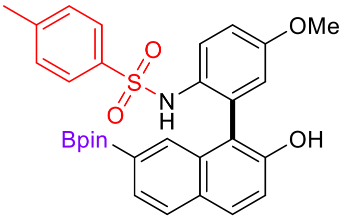

82% yield (29.2 mg) as a white solid.

^{1}H-NMR (400 MHz, $CDCl_3$) δ 7.73-7.80 (m, 3H), 7.71 (dd, *J* = 8.2, 0.9 Hz, 1H), 7.52 (s, 1H), 7.19 (d, *J* = 8.2 Hz, 2H), 7.15 (d, *J* = 8.7 Hz, 1H), 7.05 (dd, *J* = 8.9, 3.2 Hz, 1H), 6.86 (d, *J* = 8.2 Hz, 2H), 6.73 (d, *J* = 3.2 Hz, 1H), 6.38 (s, 1H), 4.92 (s, 1H), 3.78 (s, 3H), 2.22 (s, 3H), 1.34 (s, 6H), 1.33 (s, 6H).

^{13}C-NMR (100 MHz, $CDCl_3$) δ 158.00, 150.07, 143.35, 135.91, 132.04, 131.72, 131.63, 131.19, 130.79, 130.54, 129.37, 128.79, 128.43, 128.07, 127.43, 126.77, 118.67, 116.99, 115.98, 84.02, 55.74, 25.02, 21.64 (One carbon overlapped).

HRMS (ESI) calcd for $C_{30}H_{32}BNO_6SNa$: *m/z* ([M+Na$^+$]) 568.1936, found 568.1931.

IR (KBr) 3419, 3337, 2982, 2925, 1604, 1500, 1453, 1345, 1158, 733 cm^{-1}.

N-(2'-Bromo-4',6'-dihydroxy-5-methoxy-[1,1'-biphenyl]-2-yl)-4-methylbenzenesulfonamide (**3t**)

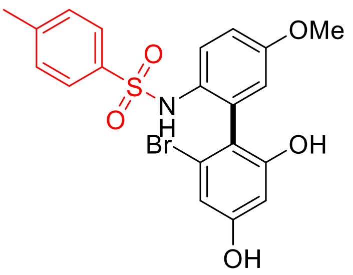

67% yield (20.2 mg) as a white solid.

^{1}H-NMR (600 MHz, $CDCl_3$) (mixture of two rotamers) δ 7.74 (d, J = 8.2 Hz, 1H), 7.52 (d, J = 8.2 Hz, 1H), 7.47 (d, J = 9.6 Hz, 0.5H), 7.42 (d, J = 9.6 Hz, 1H), 7.28 (d, J = 8.2 Hz, 1H), 7.19 (d, J = 8.2 Hz, 1H), 6.91 (dd, J = 8.9, 2.7 Hz, 0.5H), 6.80 (d, J = 8.9 Hz, 1H), 6.73 (d, J = 2.1 Hz, 0.5H), 6.64 (d, J = 2.7 Hz, 0.5H), 6.57 (d, J = 2.7 Hz, 0.5H), 6.54 (d, J = 2.7 Hz, 0.5H), 6.40 (d, J = 2.1 Hz, 0.5H), 6.35 (br s, 0.5H), 3.76 (s, 3H), 2.44 (s, 1.5H), 2.39 (s, 1.5H).

^{13}C-NMR (150 MHz, $CDCl_3$) (mixture of two rotamers) δ 158.79, 157.76, 157.57, 157.33, 156.77, 154.50, 144.57, 144.22, 136.09, 135.71, 132.99, 131.69, 129.92, 129.56, 128.63, 127.55, 127.20, 126.74, 126.27, 124.77, 121.04, 117.46, 117.35, 115.32, 114.21, 113.48, 113.13, 104.61, 102.89, 55.65, 55.53, 21.81, 21.70 (Three carbons overlapped).

HRMS (ESI) calcd for $C_{20}H_{18}BrNO_5SNa$: *m/z* ([M+Na$^+$]) 485.9981, found 485.9978.

IR (KBr) 3376, 2926, 2839, 1704, 1607, 1505, 1454, 1323, 1157, 812 cm^{-1}.

4. Supplementary Method 4: ***general procedure for the synthesis of atropoisomeric biaryls 5 in a flow system***

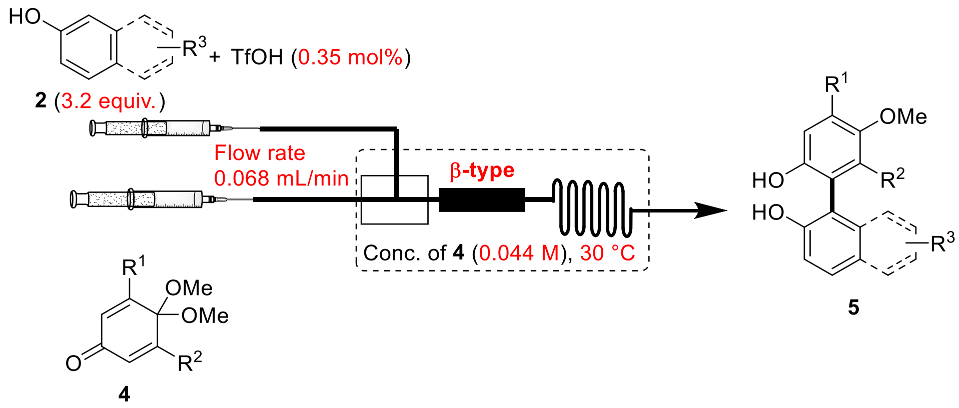

As shown in Fig. S1, a flow microreactor system was dipped in oil bath to heat at 30 ˚C. A solution of **4** (0.065 mmol) in toluene/EtOAc (10/1) (0.74 mL), and a solution of **2** (0.21 mmol, 3.2 equiv.) and TfOH (0.35 mol%) in toluene (0.74 mL) were introduced to the flow microreactor system with β-type mixer by syringe pumps (flow rate: 0.068 mL/min). After the continuous-flow was kept within residence time, the reaction mixture was collected and quenched with aq. $NaHCO_3$. The organic layer was extracted with EtOAc (15 mL × 3), dried over Na_2SO_4, concentrated *in vacuo*. The residue was purified by silica column chromatography (*n*-hexane/EtOAc) to afford **5**.

6-Bromo-5'-methoxy-[1,1'-biphenyl]-2,2',4-triol (known compound **5a**) [18]

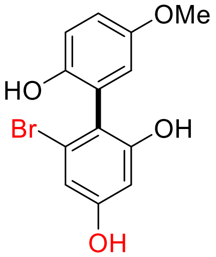

69% yield (26.7 mg) as a colorless solid.

^{1}H-NMR (400 MHz, $(CD_3)_2CO$) δ 6.83 (d, J = 8.7 Hz, 1H), 6.78 (dd, J = 8.7, 2.7 Hz, 1H), 6.70 (d, J = 2.3 Hz, 1H), 6.61 (d, J = 2.7 Hz, 1H), 6.46 (d, J = 2.3 Hz, 1H), 3.73 (s, 3H).

5'-Methoxy-6-methyl-[1,1'-biphenyl]-2,2',4-triol (**5b**)

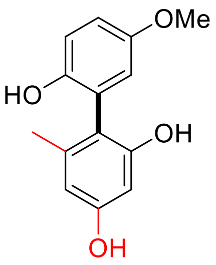

59% yield (18.9 mg) as a yellowish-white solid.

^{1}H-NMR (400 MHz, $CDCl_3$) δ 7.00 (d, J = 8.7 Hz, 1H), 6.91 (dd, J = 8.7, 2.7 Hz, 1H), 6.66 (d, J = 2.7 Hz, 1H), 6.41 (d, J = 2.3 Hz, 1H), 6.39 (d, J = 2.3 Hz, 1H), 4.89 (s, 1H), 4.88 (s, 1H), 4.59 (s, 1H), 3.77 (s, 3H), 2.04 (s, 3H).

^{13}C-NMR (100 MHz, $CDCl_3$) δ 157.02, 155.11, 154.09, 148.09, 140.32, 120.48, 117.05, 116.48, 115.89, 113.72, 109.94, 100.58, 55.92, 20.11.

HRMS (ESI) calcd for $C_{14}H_{14}O_4Na$: *m/z* ([M+Na$^+$]) 269.0784, found 269.0777.

IR (KBr) 3304, 3292, 3276, 2954, 2833, 1605, 1462, 1152, 1039, 803 cm^{-1}.

5'-Methoxy-[1,1'-biphenyl]-2,2',4-triol (**5c**)

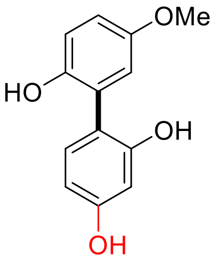

62% yield (18.7 mg) as a yellowish-white solid.

^{1}H-NMR (400 MHz, $(CD_3)_2CO$) δ 8.40 (br. s, 2H), 7.94 (br. s, 1H), 7.13 (d, J = 8.2 Hz, 1H), 6.88 (dd, J = 7.6, 1.6 Hz, 1H), 6.76-6.79 (m, 2H), 6.48-6.51 (m, 2H), 3.76 (s, 3H).

^{13}C-NMR (100 MHz, $(CD_3)_2CO$) δ 159.15, 155.58, 154.49, 148.27, 133.07, 127.93, 118.55, 117.99, 117.03, 114.42, 108.99, 104.31, 55.78.

HRMS (ESI) calcd for $C_{13}H_{12}O_4Na$: *m/z* ([M+Na$^+$]) 255.0628, found 255.0630.

IR (KBr) 3420, 3397, 3337, 3082, 2968, 2840, 1878, 1613, 1112, 834 cm^{-1}.

5'-Methoxy-4,6-dimethyl-[1,1'-biphenyl]-2,2'-diol (**5d**)

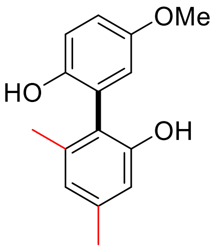

74% yield (23.5 mg) as a colorless sticky solid.

^{1}H-NMR (400 MHz, $CDCl_3$) δ 6.98 (d, J = 8.7 Hz, 1H), 6.90 (dd, J = 8.7, 3.2 Hz, 1H), 6.73 (s, 1H), 6.71 (s, 1H), 6.66 (d, J = 3.2 Hz, 1H), 4.88 (s, 1H), 4.65 (s, 1H), 3.76 (s, 3H), 2.32 (s, 3H), 2.05 (s, 3H).

^{13}C-NMR (100 MHz, $CDCl_3$) δ 154.02, 153.72, 147.80, 140.32, 138.61, 123.60, 121.04, 118.37, 117.07, 116.27, 115.60, 113.96, 55.87, 21.42, 19.93.

HRMS (ESI) calcd for $C_{15}H_{16}O_3Na$: *m/z* ([M+Na$^+$]) 267.0992, found 267.0989.

IR (KBr) 3493, 3426, 2921, 2835, 1624, 1487, 1269, 1152, 1038, 840 cm^{-1}.

6-(2-Hydroxy-5-methoxyphenyl)benzo[d][1,3]dioxol-5-ol (**5e**)

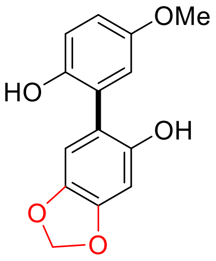

84% yield (28.4 mg) as a pale violet solid.

^{1}H-NMR (400 MHz, $CDCl_3$) δ 6.95 (d, J = 9.2 Hz, 1H), 6.87 (dd, J = 9.2, 3.2 Hz, 1H), 6.76 (d, J = 3.2 Hz, 1H), 6.71 (s, 1H), 6.60 (s, 1H), 5.97 (s, 2H), 5.32 (s, 1H), 5.02 (s, 1H), 3.78 (s, 3H).

^{13}C-NMR (100 MHz, $CDCl_3$) δ 154.29, 148.85, 148.05, 146.68, 142.41, 124.43, 117.62, 116.07, 115.59, 115.38, 109.71, 101.63, 99.18, 55.96.

HRMS (ESI) calcd for $C_{14}H_{12}O_5Na$: *m/z* ([M+Na$^+$]) 283.0577, found 283.0572.

IR (KBr) 3435, 3230, 2961, 2908, 1626, 1488, 1432, 1282, 1042, 865 cm^{-1}.

1-(2-Hydroxy-5-methoxyphenyl)naphthalen-2-ol (known compound **5f**)[19]

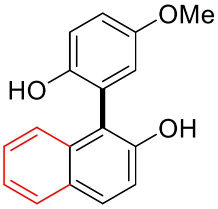

93% yield (32.2 mg) as a yellow liquid.

^{1}H-NMR (400 MHz, $CDCl_3$) δ 7.86 (d, J = 8.7 Hz, 1H), 7.84 (dd, J = 7.6, 1.6 Hz, 1H), 7.35-7.41 (m, 3H), 7.29 (d, J = 9.2 Hz, 1H), 7.07 (d, J = 9.2 Hz, 1H), 6.99 (dd, J = 9.2, 3.2 Hz, 1H), 6.79 (d, J = 3.2 Hz, 1H), 5.38 (s, 1H), 4.61 (s, 1H), 3.77 (s, 3H).

6-Hydroxy-5-(2-hydroxy-5-methoxyphenyl)-2-naphthonitrile (known compound **5g**)[20]

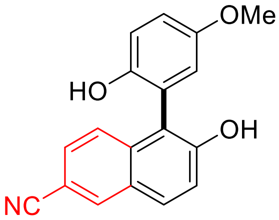

88% yield (33.3 mg) as a white solid.

^{1}H-NMR (400 MHz, $(CD_3)_2CO$) δ 8.36 (d, J = 1.4 Hz, 1H), 7.98 (d, J = 8.7 Hz, 1H), 7.54 (dd, J = 8.7, 1.4 Hz, 1H), 7.50 (d, J = 9.2 Hz, 1H), 7.42 (d, J = 8.7 Hz, 1H), 6.98 (d, J = 8.7 Hz, 1H), 6.91 (dd, J = 9.2, 3.2 Hz, 1H), 6.75 (d, J = 3.2 Hz, 1H), 3.76 (s, 3H).

6-Bromo-1-(2-hydroxy-5-methoxyphenyl)naphthalen-2-ol (known compound **5h**)[21]

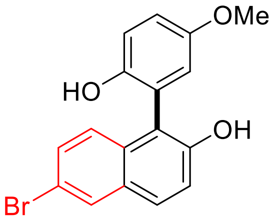

88% yield (37.7 mg) as a brown solid.

^{1}H-NMR (400 MHz, $CDCl_3$) δ 7.99 (d, J = 2.1 Hz, 1H), 7.77 (d, J = 9.1 Hz, 1H), 7.46 (dd, J = 2.1, 8.8 Hz, 1H), 7.33–7.27 (m, 2H), 7.08 (d, J = 9.1 Hz, 1H), 7.01 (dd, J = 2.8, 8.8 Hz, 1H), 6.77 (d, J = 3.2 Hz, 1H), 5.37 (s, 1H), 4.52 (s, 1H), 3.79 (s, 3H).

1-(2-hydroxy-5-methoxyphenyl)-7-methoxynaphthalen-2-ol (known compound **5i**)[20]

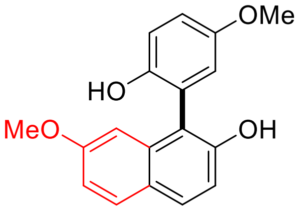

63% yield (24.3 mg) as a yellow liquid (the reaction carried out at 35 °C with 0.055 mL/min flow rate).

^{1}H-NMR (400 MHz, $CDCl_3$) δ 7.79 (d, J = 8.7 Hz, 1H), 7.74 (d, J = 9.2 Hz, 1H), 7.14 (d, J = 9.2 Hz, 1H), 7.10 (d, J = 9.2 Hz, 1H), 7.02 (m, 2H), 6.81 (d, J = 3.2 Hz, 1H), 6.67 (d, J = 2.3 Hz, 1H), 5.25 (s, 1H), 4.55 (s, 1H), 3.79 (s, 3H), 3.73 (s, 3H).

2-(2-Hydroxy-5-methoxyphenyl)naphthalen-1-ol (**5j**)

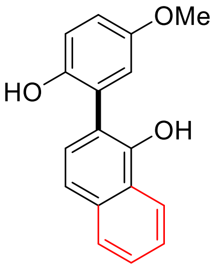

58% yield (20.1 mg) as a white solid.

^{1}H-NMR (400 MHz, $(CD_3)_2CO$) δ 8.37-8.39 (m, 1H), 7.85-7.88 (m, 1H), 7.50-7.53 (m, 3H), 7.46 (d, J = 8.7 Hz, 1H), 7.04 (d, J = 8.7 Hz, 1H), 6.94 (d, J = 3.2 Hz, 1H), 6.88 (dd, J = 8.7, 3.2 Hz, 1H), 3.80 (s, 3H).

^{13}C-NMR (100 MHz, $(CD_3)_2CO$) δ 154.80, 150.48, 147.85, 135.20, 129.86, 128.09, 127.34, 127.05, 127.01, 125.97, 123.63, 120.84, 120.55, 117.68, 117.53, 115.39, 55.91.

HRMS (ESI) calcd for $C_{17}H_{14}O_3Na$: m/z ([M+Na$^+$]) 289.0835, found 289.0830.

IR (KBr) 3151, 3057, 2999, 2956, 1869, 1509, 1415, 1368, 1038, 819 cm^{-1}.

1-(2-Hydroxy-5-methoxy-4-methylphenyl)naphthalen-2-ol (known compound **5k**)[18]

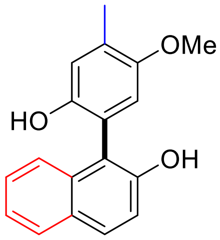

97% yield (35.3 mg) as a yellow liquid.

^{1}H-NMR (400 MHz, CD_3OD) δ 7.64-7.67 (m, 2H), 7.32 (dd, J = 8.3, 0.9 Hz, 1H), 7.09-7.19 (m, 3H), 6.73 (s, 1H), 6.56 (s, 1H), 3.60 (s, 3H), 2.15 (s, 3H).

6-(2-Hydroxy-5-methoxy-4-methylphenyl)benzo[d][1,3]dioxol-5-ol (**5l**)

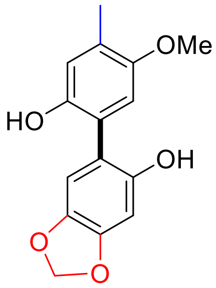

92% yield (32.8 mg) as a violet solid.

^{1}H-NMR (400 MHz, $(CD_3)_2CO$) δ 8.15 (br. s, 2H), 6.80 (s, 1H), 6.78 (s, 1H), 6.76 (s, 1H), 6.54 (s, 1H), 5.96 (s, 2H), 3.81 (s, 3H), 2.15 (s, 3H).

^{13}C-NMR (100 MHz, $(CD_3)_2CO$) δ 152.66, 149.61, 148.65, 147.54, 142.43, 127.27, 124.34, 119.63, 119.23, 113.89, 110.90, 102.08, 99.54, 56.04, 16.02.

HRMS (ESI) calcd for $C_{15}H_{14}O_5Na$: m/z ([M+Na$^+$]) 297.0733, found 297.0728.

IR (KBr) 3482, 3273, 2955, 2881, 2323, 1626, 1479, 1408, 1172, 875 cm^{-1}.

1-(6-Hydroxy-3-methoxy-2,4-dimethylphenyl)naphthalen-2-ol (known compound **5m**)[18]

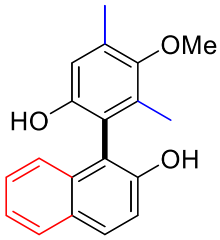

73% yield (27.9 mg) as an orange solid.

^{1}H-NMR (400 MHz, CD_3OD) δ 7.66-7.69 (m, 2H), 7.06-7.17 (m, 4H), 6.59 (s, 1H), 3.61 (s, 3H), 2.23 (s, 3H), 1.73 (s, 3H).

6-(6-Hydroxy-3-methoxy-2,4-dimethylphenyl)benzo[d][1,3]dioxol-5-ol (**5n**)

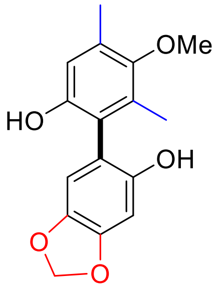

72% yield (27.0 mg) as a yellow solid (the reaction carried out at 35 °C with 0.048 mL/min flow rate using 0.50 mol% TfOH).

^{1}H-NMR (400 MHz, $(CD_3)_2CO$) δ 7.42 (s, 1H), 7.16 (s, 1H), 6.58 (s, 1H), 6.52 (s, 1H), 6.49 (s, 1H), 5.94 (q, J = 1.4 Hz, 2H), 3.63 (s, 3H), 2.21 (s, 3H), 1.98 (s, 3H).

^{13}C-NMR (100 MHz, $(CD_3)_2CO$) δ 151.78, 150.94, 150.59, 148.58, 141.66, 131.96, 131.14, 123.86, 115.74, 111.34, 101.86, 98.86, 98.78, 59.95, 16.23, 13.72.

HRMS (ESI) calcd for $C_{16}H_{16}O_5Na$: m/z ([M+Na$^+$]) 311.0890, found 311.0887.

IR (KBr) 3480, 3398, 3044, 2888, 2524, 1703, 1624, 1294, 1082, 844 cm^{-1}.

5. Supplementary Note 1: *preliminary result for the asymmetric synthesis of atropoisomeric biaryl 3h*

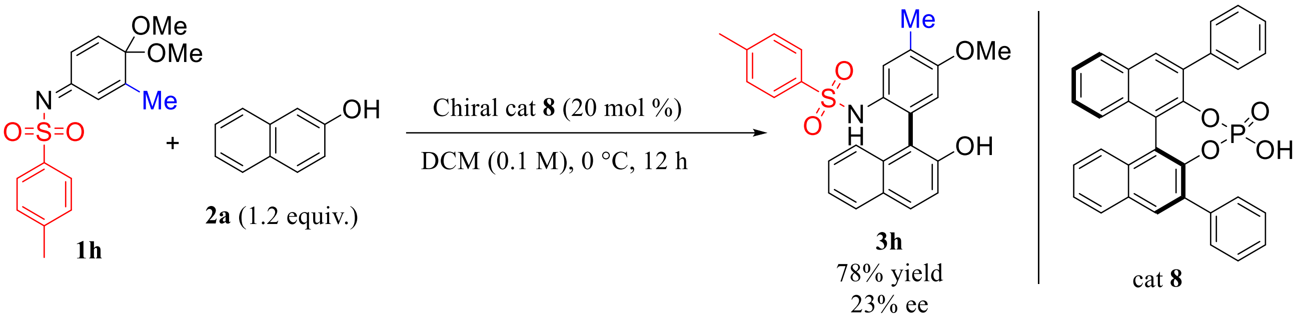

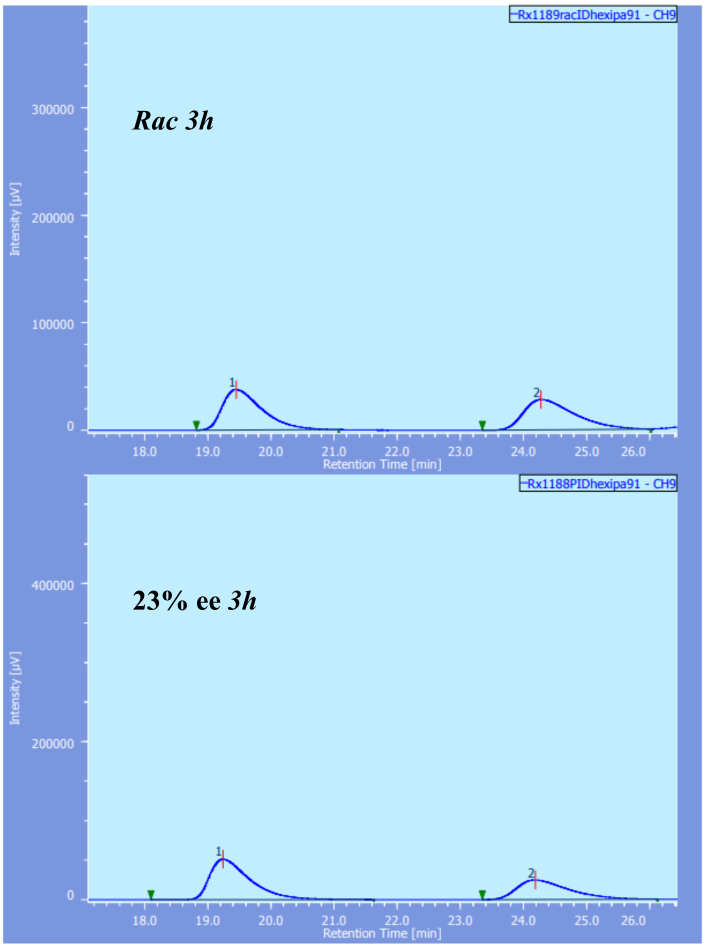

Channel & Peak Information Table

Chromatogram Name Rx1189racIDhexipa91−CH9

Sample Name

Channel Name 254.0nm

#	Peak Name	CH	tR [min]	Area [µV·sec]	Height [µV]	Area%	Height%	Quantity	NTP	Resolution	Symmetry Factor	Warning
1	Unknown	9	19.447	1645370	37626	51.217	57.291	N/A	4806	3.750	1.725	
2	Unknown	9	24.272	1567172	28049	48.783	42.709	N/A	4432	N/A	1.672	

Chromatogram Name Rx1188PIDhexipa91−CH9

Sample Name

Channel Name 254.0nm

#	Peak Name	CH	tR [min]	Area [µV·sec]	Height [µV]	Area%	Height%	Quantity	NTP	Resolution	Symmetry Factor	Warning
1	Unknown	9	19.238	2263066	50978	61.648	67.522	N/A	4742	3.806	1.879	
2	Unknown	9	24.183	1407903	24521	38.352	32.478	N/A	4226	N/A	1.677	

HPLC conditions: Daicel Chiralpak ID column, 2-propanol/*n*-hexane = 1/9, flow rate 1.0 mL/min, 254 nm; 19.2 min. (major isomer) and 24.1 min. (minor isomer).

6. Supplementary Note 2: *transformations of 3e and 5f*

6.1 Removal of Ms group

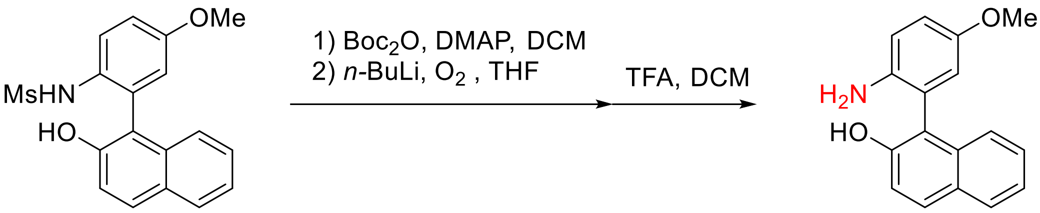

3e **6** (86% yield)

According to the reported procedure[23], the methanesulfonyl group in **3e** was successfully removed to afford the corresponding product **6**: To a stirring solution of **3e** (50 mg, 0.145 mmol) and Boc_2O (2.4 equiv, 0.34 mmol) in DCM (10 mL), DMAP (0.4 equiv) was added at room temperature under air atmosphere. After stirring for 1 h, DCM was removed under reduced pressure and the obtained residue was used without further purification. Under N_2 gas atmosphere, dry THF (0.04M) was added and, *n*-BuLi (2.6 M in hexane, 3.0 equiv., 0.168 mL) was added dropwise to the mixture at 0 ˚C. After stirring for 20 min, N_2 gas balloon was replaced by a dry O_2 balloon and kept for stirring at room temperature. After stirring for 1h, water was added, and the aqueous phase was extracted with EtOAc three times. The combined organic layers were dried over anhydrous Na_2SO_4 and evaporated *in vacuo*. Finally, TFA (0.3 mL) was added dropwise to the mixture in DCM (5 mL). After stirring for 1 h, the mixture was evaporated *in vacuo*, and directly purified by column chromatography on SiO_2 (acetone/*n*-hexane = 1:1) to afford compound **6** (33.1 mg, 86% yield) as a brown solid.

1-(2-Amino-5-methoxyphenyl)naphthalen-2-ol (**6**)

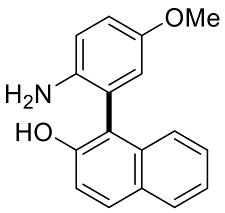

^{1}H-NMR (600 MHz, $CDCl_3$) δ 7.83 (d, J = 8.2 Hz, 2H), 7.53 (d, J = 8.2 Hz, 1H), 7.39 (ddd, J = 7.6, 7.3, 1.4 Hz, 1H), 7.35 (ddd, J = 7.6, 6.9, 1.4 Hz, 1H), 7.30 (d, J = 8.2 Hz, 1H), 6.90-6.94 (m, 2H), 6.80 (d, J = 2.7 Hz, 1H), 3.76 (s, 3H).

^{13}C-NMR (175 MHz, $CDCl_3$) δ 153.44, 151.34, 138.16, 133.10, 130.16, 129.48, 128.41, 126.88, 124.79, 123.80, 120.94, 118.66, 118.29, 117.64, 116.99, 116.40, 55.90.

HRMS (ESI) calcd for $C_{17}H_{15}NO_2Na$: *m/z* ([M+Na$^+$]) 288.0995, found 288.0993.

IR (KBr) 3389, 3322, 3075, 1617, 1506, 1387, 1213, 1173, 1039, 755 cm^{-1}

6.2 Reductive coupling of 5 with diphenyl phosphine oxide

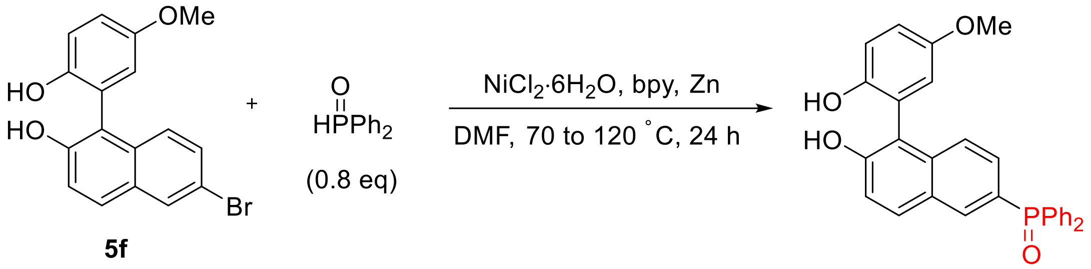

7 (60% yield)

The Ni(II)/Zn catalyzed reductive coupling of **5f** with diphenyl phosphine oxide was carried out following the literature procedure[24]: To a reaction vessel containing $NiCl_2.6H_2O$ (0.024 mmol), zinc (0.23 mmol), 2,2'-bipyridine (bpy) (0.048 mmol),

and **5f** (50 mg, 0.14 mmol) dissolved in DMF (0.6 M), diphenylphosphine oxide (0.8 equiv., 0.12 mmol) was added portionwise and the reaction mixture was stirred at 120 °C. After stirring for 24 h, it was allowed to cool to room temperature, quenched with water, and extracted with EtOAc three times. The organic layers were combined, dried over anhydrous Na_2SO_4, evaporated *in vacuo*, and purified by column chromatography on SiO_2 using dichloromethane-methanol as eluent to afford **7** (20.2 mg, 60% yield) as a white solid.

(6-Hydroxy-5-(2-hydroxy-5-methoxyphenyl)naphthalen-2-yl)diphenylphosphine oxide (**7**)

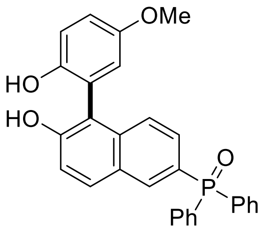

^{1}H-NMR (600 MHz, CD_3OD) δ 8.08 (d, J = 13.7 Hz, 1H), 7.81 (d, J = 8.9 Hz, 1H), 7.63-7.70 (m, 6H), 7.54-7.57 (m, 4H), 7.50 (dd, J = 8.9, 2.7 Hz, 1H), 7.43 (t, J = 9.3 Hz, 1H), 7.30 (d, J = 8.9 Hz, 1H), 6.91 (d, J = 8.9 Hz, 1H), 6.88 (dd, J = 8.9, 2.7 Hz, 1H), 6.71 (d, J = 2.7 Hz, 1H), 3.74 (s, 3H).
^{13}C-NMR (175 MHz, CD_3OD) δ 156.26, 154.54, 150.47, 137.34, 135.20 (d, $J_{C\text{-}P}$ = 10.4 Hz), 133.70, 133.15 (d, $J_{C\text{-}P}$ = 10.4 Hz), 132.98 (d, $^{1}J_{C\text{-}P}$ = 105.33 Hz), 131.29, 129.97 (d, $J_{C\text{-}P}$ = 11.7 Hz), 128.86 (d, $J_{C\text{-}P}$ = 14.3 Hz), 127.58 (d, $J_{C\text{-}P}$ = 10.4 Hz), 126.79 (d, $J_{C\text{-}P}$ = 13.00 Hz), 125.33 (d, $^{1}J_{C\text{-}P}$ = 107.93 Hz), 124.17, 120.83, 119.91, 118.46, 117.79, 115.90, 56.18.
^{31}P NMR (243 MHz, CD_3OD): δ 33.49.
HRMS (ESI) calcd for $C_{29}H_{23}O_4PNa$: m/z ([M+Na$^+$]) 489.1226, found 489.1240.
IR (KBr) 3386, 3068, 1611, 1512, 1438, 1208, 1168, 1119, 726, 700 cm^{-1}

7. Supplementary Note 3: *microreactor information*

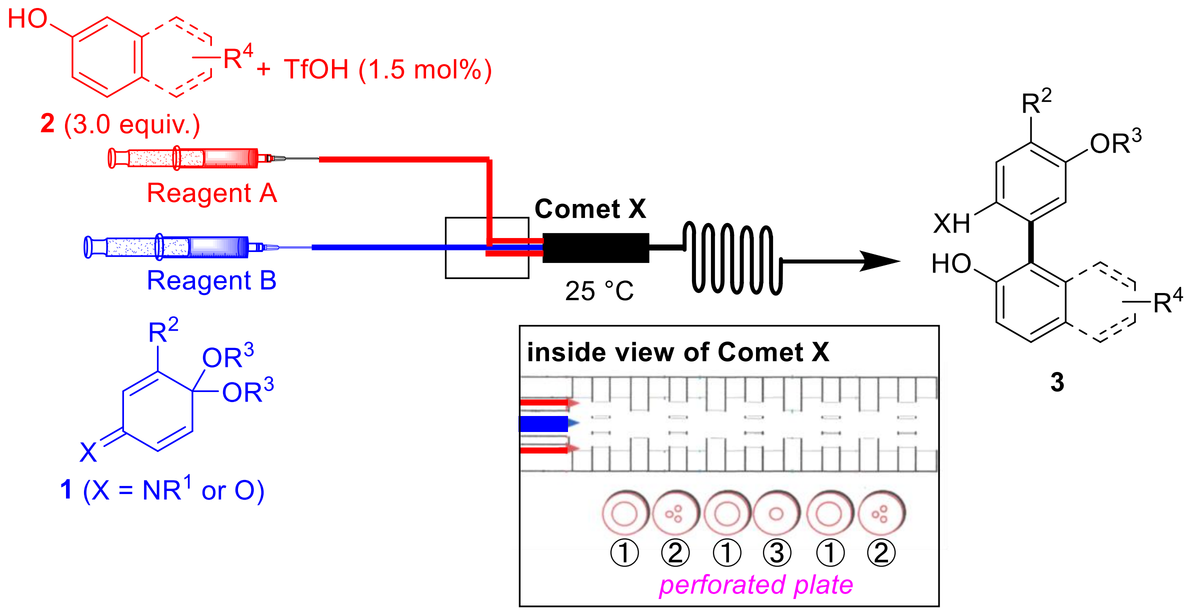

Comet X (Total solution holding capacity: 29.4 μL) equips 19 mixing spaces made by three different types of perforated-plates ①②③ in the stainless steel cylinder. Reagent B (substrate **1** in toluene) introduced to the start of the micro flow reactor from a 0.96 mm inner tube is surrounded by Reagent A (substrate **2** and TfOH in toluene), which has moved through a gap of 2.0 mm outer tube inner diameter and 1.56 mm outer tube diameter, over the entire surface area of the cylindrical release. After the first mixing of Reagent A/Reagent B, the mixture is pushed into three of ϕ0.5 hole on the plate immediately. In the micro spaces, Reagent B enters from the circumference side along the centre of the entire micro flow reactor, and the mixture enters from the circumference outside the centre. In the three-hole spaces, Reagent B enters from the circumference

side along the centre of the entire microflow reactor, and Reagent A enters from the circumference outside the centre. The mass transfer is extremely accelerated due to concentration gradient. And then the reaction takes place at the hall. The mixture divided into 3 parts are again integrated into one channel of ϕ 1.0 from the hall of ϕ 2.0. These results in a more concentrated mixing of the reagents and further reaction. Then, it passes through the ϕ 2.0 hall again and is dispersed into three ϕ 0.5 micro spaces.

The Micro Flow Reactor [Comet X] repeats the above process totally five times.

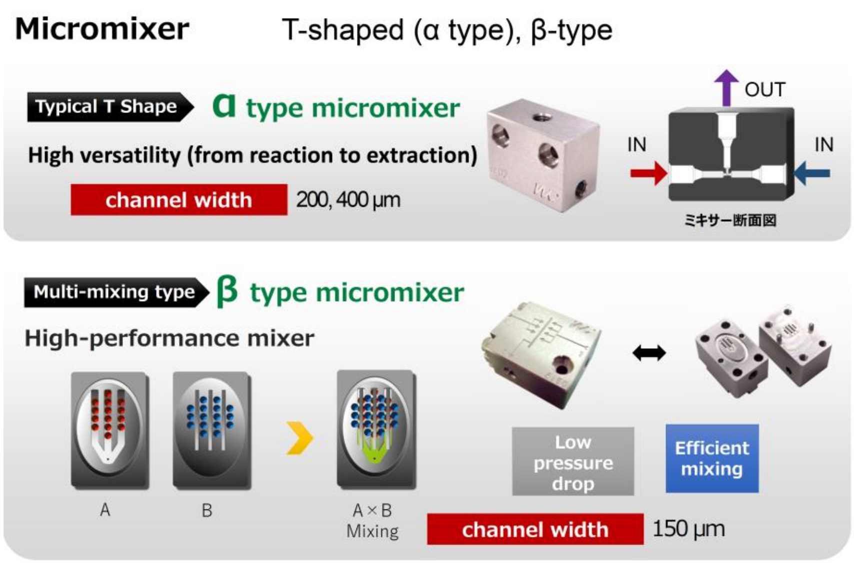

In β-type micromixer, substrate solution flows through multiple holes in A and B and is mixed. The red hole in A is blocked in the center by a protrusion on the B side, and the substrate solution emerges from the half-moon-shaped part. The blue hole in B is similarly blocked in the center by a protrusion on the A side, and the substrate solution emerges from the half-moon-shaped part. The substrate solution flows out of the half-moon-shaped part in B. The substrate solution mixes with the substrate solution and flows through the channel formed by the protrusions in A and B, and then flows out of the hole in the lower part of A side.

Fig. S2 Information for flow microreactors: Comet-X, T-shaped, and β-type

8. Supplementary Note 4: *screening of microreactors under same reaction conditions*

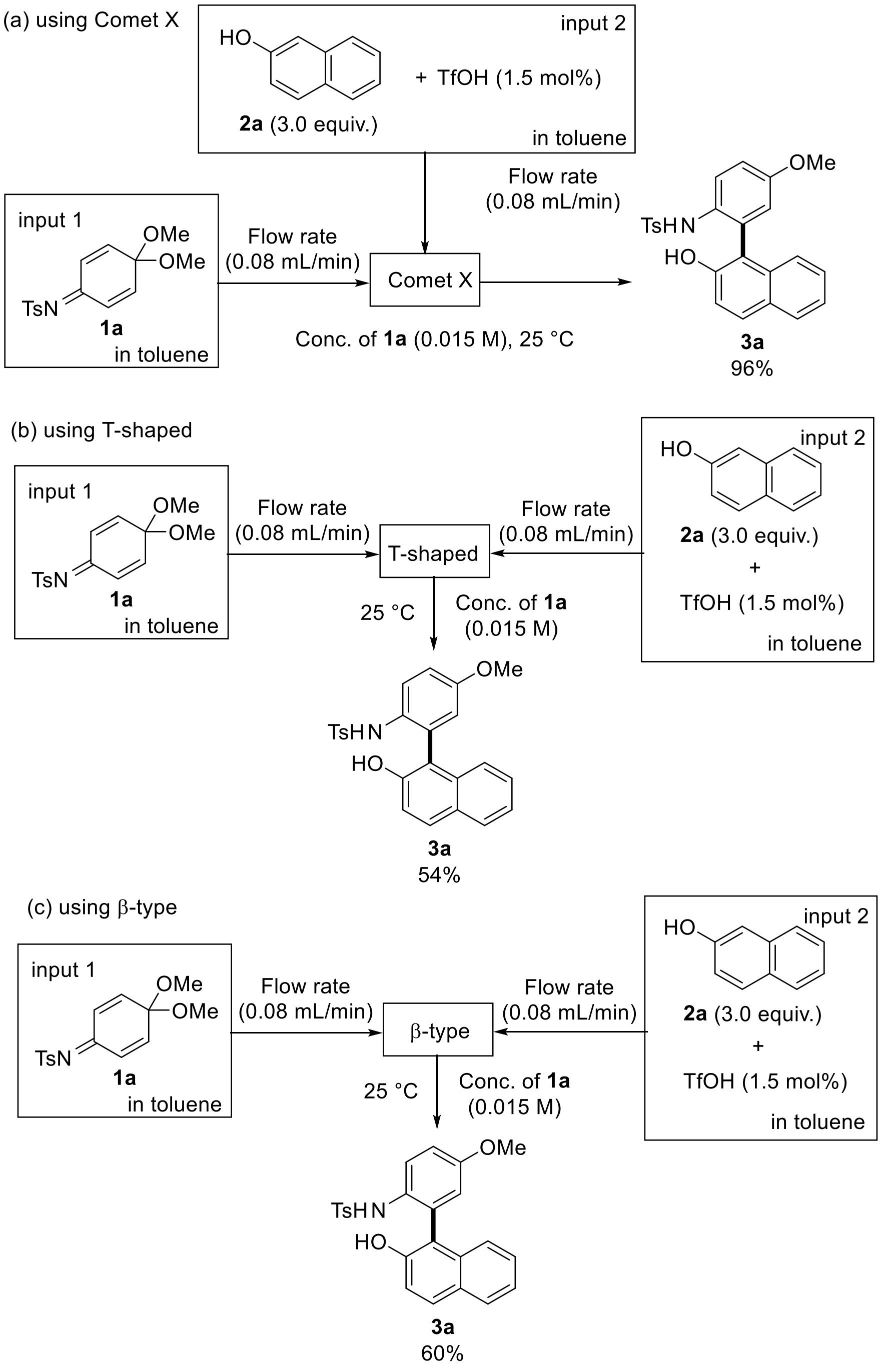

Scheme S1 A head-to-head comparison of the microreactors

9. Supplementary Note 5: *mechanistic study*

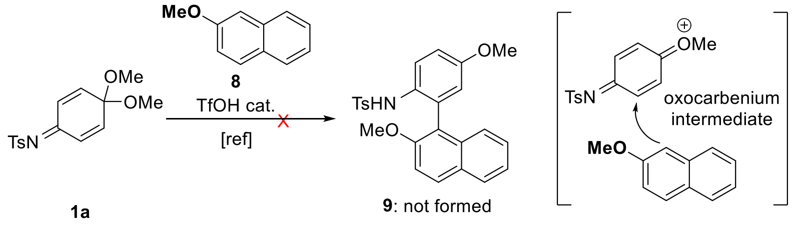

Fig. S3 Control experiment

10. Supplementary Note 6: ***X-ray crystallographic analysis***

X-ray crystallographic analysis of 5j (CCDC 2142538)

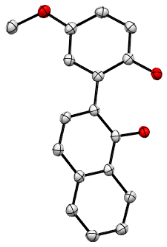

Identification code	Req158-1
Empirical formula	C17H14O3
Formula weight	266.28
Temperature/K	100
Crystal system	monoclinic
Space group	P21/c
a/Å	13.6920(2)
b/Å	11.2743(2)
c/Å	8.57540(10)
α/°	90
β/°	98.6050(10)
γ/°	90
Volume/Å3	1308.86(3)
Z	4
ρcalcg/cm3	1.351
μ/mm-1	0.749
F(000)	560.0
Crystal size/mm3	0.14 × 0.11 × 0.09
Radiation	CuKα (λ = 1.54184)
2Θ range for data collection/°	6.528 to 151.918
Index ranges	-17 ≤ h ≤ 17, -13 ≤ k ≤ 13, -10 ≤ l ≤ 10
Reflections collected	13180
Independent reflections	2658 [Rint = 0.0290, Rsigma = 0.0225]
Data/restraints/parameters	2658/0/184
Goodness-of-fit on F2	1.042
Final R indexes [I>=2σ (I)]	R1 = 0.0335, wR2 = 0.0947
Final R indexes [all data]	R1 = 0.0360, wR2 = 0.0969
Largest diff. peak/hole / e Å-3	0.19/-0.22

Datablock: req158-1

Bond precision:	C-C = 0.0015 A		Wavelength=1.54184
Cell:	a=13.6920(2)	b=11.2743(2)	c=8.5754(1)
	alpha=90	beta=98.605(1)	gamma=90
Temperature: 100 K			

	Calculated	Reported
Volume	1308.86(3)	1308.86(3)
Space group	P 21/c	P 1 21/c 1
Hall group	-P 2ybc	-P 2ybc
Moiety formula	C17 H14 O3	C17 H14 O3
Sum formula	C17 H14 O3	C17 H14 O3
Mr	266.28	266.28
Dx,g cm-3	1.351	1.351
Z	4	4
Mu (mm-1)	0.749	0.749
F000	560.0	560.0
F000'	561.76	
h,k,lmax	17,14,10	17,13,10
Nref	2732	2658
Tmin,Tmax	0.906,0.935	0.958,1.000
Tmin'	0.900	

Correction method= # Reported T Limits: Tmin=0.958 Tmax=1.000
AbsCorr = MULTI-SCAN

Data completeness= 0.973 Theta(max)= 75.959

R(reflections)= 0.0335(2425) wR2(reflections)= 0.0969(2658)

S = 1.042 Npar= 184

The following ALERTS were generated. Each ALERT has the format
test-name_ALERT_alert-type_alert-level.
Click on the hyperlinks for more details of the test.

Alert level G

PLAT005_ALERT_5_G No Embedded Refinement Details Found in the CIF Please Do !
PLAT007_ALERT_5_G Number of Unrefined Donor-H Atoms .... 2 Report
PLAT720_ALERT_4_G Number of Unusual/Non-Standard Labels .... 34 Note

0 ALERT level A = Most likely a serious problem - resolve or explain
0 ALERT level B = A potentially serious problem, consider carefully
0 ALERT level C = Check. Ensure it is not caused by an omission or oversight
3 ALERT level G = General information/check it is not something unexpected

0 ALERT type 1 CIF construction/syntax error, inconsistent or missing data
0 ALERT type 2 Indicator that the structure model may be wrong or deficient
0 ALERT type 3 Indicator that the structure quality may be low
1 ALERT type 4 Improvement, methodology, query or suggestion
2 ALERT type 5 Informative message, check

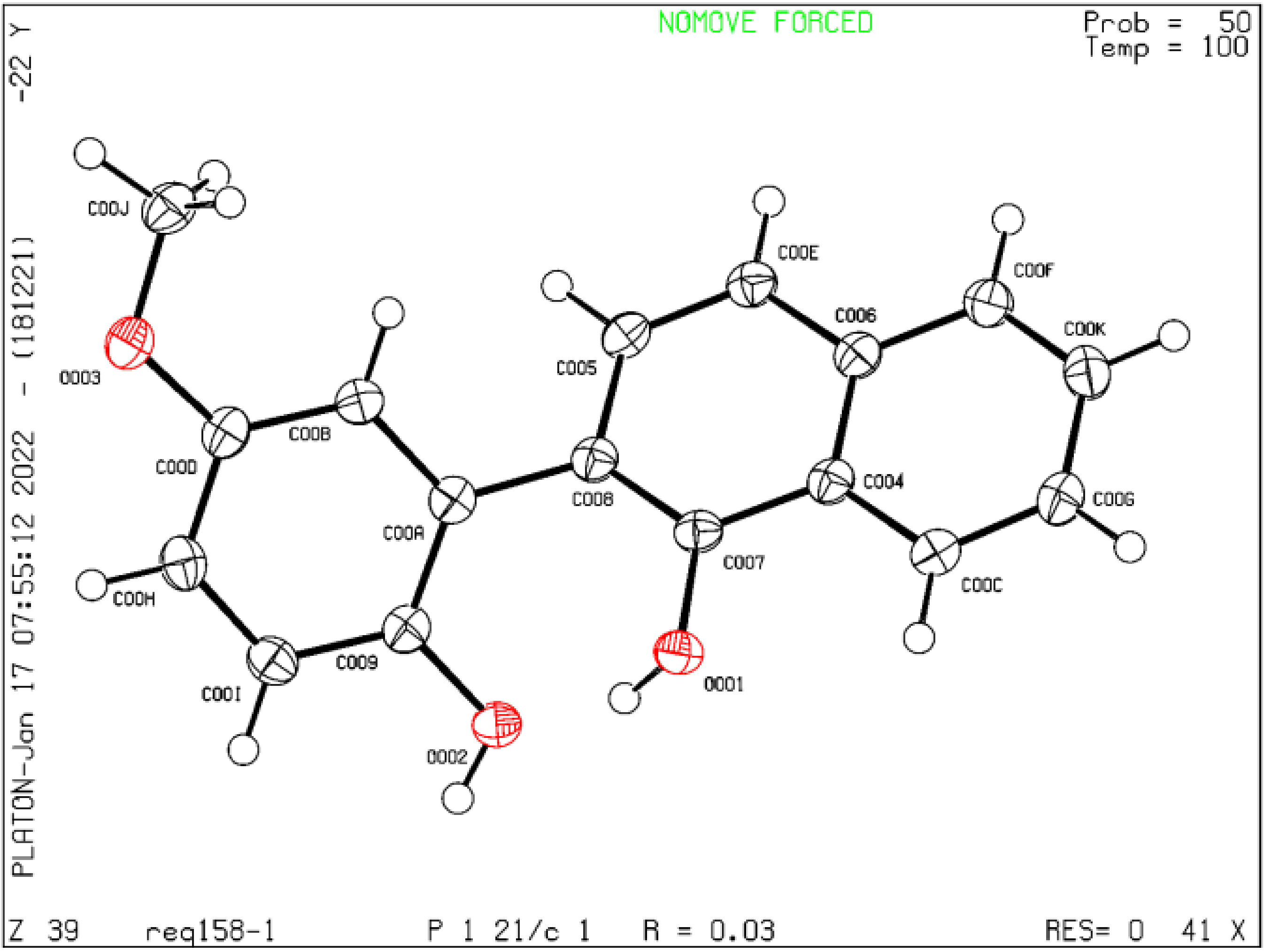
NOMOVE FORCED
Prob = 50
Temp = 100
-22 Y
PLATON-Jan 17 07:55:12 2022 - (181221)
C00J
O003
C00B
C00D
C00A
C00H
C009
C00I
O002
C005
C00E
C006
C00F
C00K
C008
C004
C00G
C007
C00C
O001
Z 39
req158-1
P 1 21/c 1
R = 0.03
RES= 0 41 X

11. Supplementary references

1. Selected publications on the flow reaction with Comet X mixer, see: (a) Koo, H., Kim, H. Y. & Oh, K. (E)-Selective Friedel–Crafts acylation of alkynes to β-chlorovinyl ketones: Defying isomerizations in batch reactions by flow chemistry approaches. *Org. Chem. Front.* **6**, 1868-1872 (2019); (b) Doi, T., Otaka, H., Umeda, K. & Yoshida, M. Study for diastereoselective aldol reaction in flow: Synthesis of (*E*)-(*S*)-3-hydroxy-7-tritylthio-4-heptenoic acid, a key component of cyclodepsipeptide HDAC inhibitors. *Tetrahedron* **71**, 6463-6470 (2015); (c) Pradipta, A. R., Tsutsui, A., Ogura, A., Hanashima, S., Yamaguchi, Y., Kurbangalieva, A. & Tanaka, K. Microfluidic mixing of polyamine with acrolein enables the detection of the [4+4] polymerization of intermediary unsaturated imines: The properties of a cytotoxic 1,5-diazacyclooctane hydrogel. *Synlett* **25**, 2442-2446 (2014); (d) Uchinashi, Y., Tanaka, K., Manabe, Y., Fujimoto, Y. & Fukase, K. Practical and efficient method for α-sialylation with an azide sialyl donor using a microreactor. *J. Carbohydr. Chem.* **33**, 55-67 (2014).
2. Furura, A., Okada, K., Fukuyama, T., Efficient anionic ring opening polymerization of ethylene oxide under microfluidic conditions. *Bull. Chem. Soc. Jpn.* **90**, 838-842 (2017)
3. Cabrera-Afonso, M. J., Carreño, M. C. & Urbano, A. Site-selective oxidative dearomatization of phenols and naphthols into *ortho*-quinols or epoxy *ortho*-quinols using oxone as the source of dimethyldioxirane. *Adv. Synth. Catal.* **361**, 4468-4473 (2019).
4. Zhang, W., Li, T., Wang, Q. & Zhao, W. Lewis acid-mediated cyanation of phenols using *N*-cyano-*N*-phenyl-*p*-toluene sulfonamide. *Adv. Synth. Catal.* **361**, 4914-4918 (2019).
5. Xia, Y., Chang, F., Lin, L., Xu, Y., Liu, X. & Feng, X. Asymmetric ring-opening of cyclopropyl ketones with *β*-naphthols catalyzed by a chiral *N*, *N'*-dioxide–scandium (iii) complex. *Org. Chem. Front.* **5**, 1293-1296 (2018).
6. Kamble, S. B., Maliekal, P. J., Dharpure, P. D., Badani, P. M. & Karnik, A. V. Synthesis of concave and vaulted *2H*-pyran-fused BINOLs and corresponding [5] and [7]-oxa-helicenoids: Regioselective cascade-concerted route and DFT studies. *J. Org. Chem.* **85**, 7739-7747 (2020).
7. Kim, D., Xuan, Q. P., Moon, H., Jun, Y. W., Ahn, K. H. Synthesis of benzocoumarins and characterization of their photophysical properties. *Asian J. Org. Chem.* **3**, 1089-1096 (2014).
8. Niculescu-Duvaz, D., Niculescu-Duvaz, I., Suijkerbuijk, B. M., Ménard, D., Zambon, A., Davies, L., Pons, J. F., Whittaker, S., Marais, R. & Springer, C. J. Potent BRAF kinase inhibitors based on 2,4,5-trisubstituted imidazole with naphthyl and benzothiophene 4-substituents. *Bioorg. Med. Chem.* **21**, 1284-1304 (2013).
9. Mandai, H., Hironaka, T., Mitsudo, K. & Suga, S. Acylative desymmetrization of cyclic meso-1, 3-diols by chiral DMAP derivatives. *Chem. Lett.* **50**, 471-474 (2021).
10. Kondo, M., Wathsala, H. D. P., Sako, M., Hanatani, Y., Ishikawa, K., Hara, S., Takaai, T., Washio, T., Takizawa, S. & Sasai, H. Exploration of flow reaction conditions using machine-learning for enantioselective organocatalyzed Rauhut–Currier and [3+ 2] annulation sequence. *Chem. Commun.* **56**, 12256-12256 (2020).
11. Kishi, K., Takizawa, S. & Sasai, H. Phosphine-catalyzed dual umpolung domino Michael reaction: Facile synthesis of hydroindole-and hydrobenzofuran-2-carboxylates. *ACS Catal.* **8**, 5228-5232 (2018).
12. Carreno, M. C. & Ribagorda, M. Anodic oxidation of *N*-protected 4-methoxy anilines: Improved synthesis of quinone imine acetals. *J. Org. Chem*. **65**, 1231-1234 (2000).
13. Liu, T., Li, Y., Cheng, F., Shen, X., Liu, J. & Lin, J. Highly chemo-and regioselective C–P cross-coupling reaction of quinone imine ketals with Ar_2P (O) H to construct *ortho*-amino triarylphosphine derivatives. *Green Chem.* **21**, 3536-3541 (2019).

14. More, S. G. & Kamble, R. B. Suryavanshi, G. Oxidative radical-mediated addition of ethers to quinone imine ketals: An access to hemiaminals. *J. Org. Chem.* **86**, 2107-2116 (2021).
15. McKillop, A., Perry, D. H., Edwards, M., Antus, S., Farkas, L., Nogradi, M. & Taylor, E. C. Thallium in organic synthesis. XLII. Direct oxidation of 4-substituted phenols to 4, 4-disubstituted cyclohexa-2, 5-dienones using thallium (III) nitrate. *J. Org. Chem.*, **41**, 282-287 (1976).
16. Yin, Z., Zhang, J., Wu, J., Liu, C., Sioson, K., Devany, M., Hu, C. & Zheng, S. Double hetero-Michael addition of *N*-substituted hydroxylamines to quinone monoketals: synthesis of bridged isoxazolidines. *Org. Lett.*, **15**, 3534-3537 (2013).
17. Jacob, A., Roy, T., Kaicharla, T. & Biju, A. T. Metal-free, Brønsted acid-catalyzed formal [3+ 2] annulation of quinone monoacetals with 2-naphthols. *J. Org. Chem.* **82**, 11269-11274 (2017).
18. Kamitanaka, T., Morimoto, K., Tsuboshima, K., Koseki, D., Takamuro, H., Dohi, T. & Kita, Y. Efficient coupling reaction of quinone monoacetal with phenols leading to phenol biaryls. *Angew. Chem. Int. Ed.* 55, 15535-15538 (2016).
19. More, N. Y. & Jeganmohan, M. Oxidative cross-coupling of two different phenols: an efficient route to unsymmetrical biphenols. *Org. Lett.* **17**, 3042-3045 (2015).
20. Wang, J., Zhao, Y., Gao, H., Gao, G. L., Yang, C. & Xia, W. Visible-light-mediated ddehydrogenative cross-coupling: synthesis of nonsymmetrical atropisomeric biaryls. *Asian J. Org. Chem.* **6**, 1402-1407 (2017).
21. Sharma, S., Parumala, S. K. R. & Peddinti, R. K. Lewis acid-mediated site-selective synthesis of oxygenated biaryls from methoxyphenols and electron-rich arenes. *J. Org. Chem.* **82**, 9367-9383 (2017).
22. Akiyama, T., Itoh, J., Yokota, K., Fuchibe, K. Enantioselective Mannich-type reaction catalyzed by a chiral Brønsted acid. *Angew. Chem. Int. Ed.* **43**, 1566-1568 (2004).
23. Gao, H., Xu, Q. L., Keene, C., Yousufuddin, M., Ess, D. H. & Kürti, L. Practical organocatalytic synthesis of functionalized non-*C2*-Symmetrical atropisomeric biaryls. *Angew. Chem. Int. Ed.* **55**, 566-571 (2016).
24. Zhang, X., Liu, H., Hu, X., Tang, G., Zhu, J., Zhao & Y. Ni (II)/Zn catalyzed reductive coupling of aryl halides with diphenylphosphine oxide in water. *Org. Lett.* **13**, 3478-3481 (2011).
