# Supplementary material for: Bayesian optimization-driven parallel-screening of multiple parameters for the flow synthesis of biaryl compounds
Source: Commun Chem. 2022 Nov 10;5:148. doi: 10.1038/s42004-022-00764-7 (PMC9814103; doi:10.1038/s42004-022-00764-7)
Supplement: Supplementary file 3 — Description of Additional Supplementary Files [file 42004_2022_764_MOESM3_ESM.pdf]

Description of Additional Supplementary Files

File name: Supplementary Data 1

Description: NMR spectra

File name: Supplementary Data 2

Description: BO scripts

File name: Supplementary Data 3

Description: Cif file of compound 5j
