# Supplementary material for: Bayesian optimization-driven parallel-screening of multiple parameters for the flow synthesis of biaryl compounds
Source: Commun Chem. 2022 Nov 10;5:148. doi: 10.1038/s42004-022-00764-7 (PMC9814103; doi:10.1038/s42004-022-00764-7)
Supplement: Supplementary file 4 — Supplementary Data 1 [file 42004_2022_764_MOESM4_ESM.pdf]

NMR Spectra

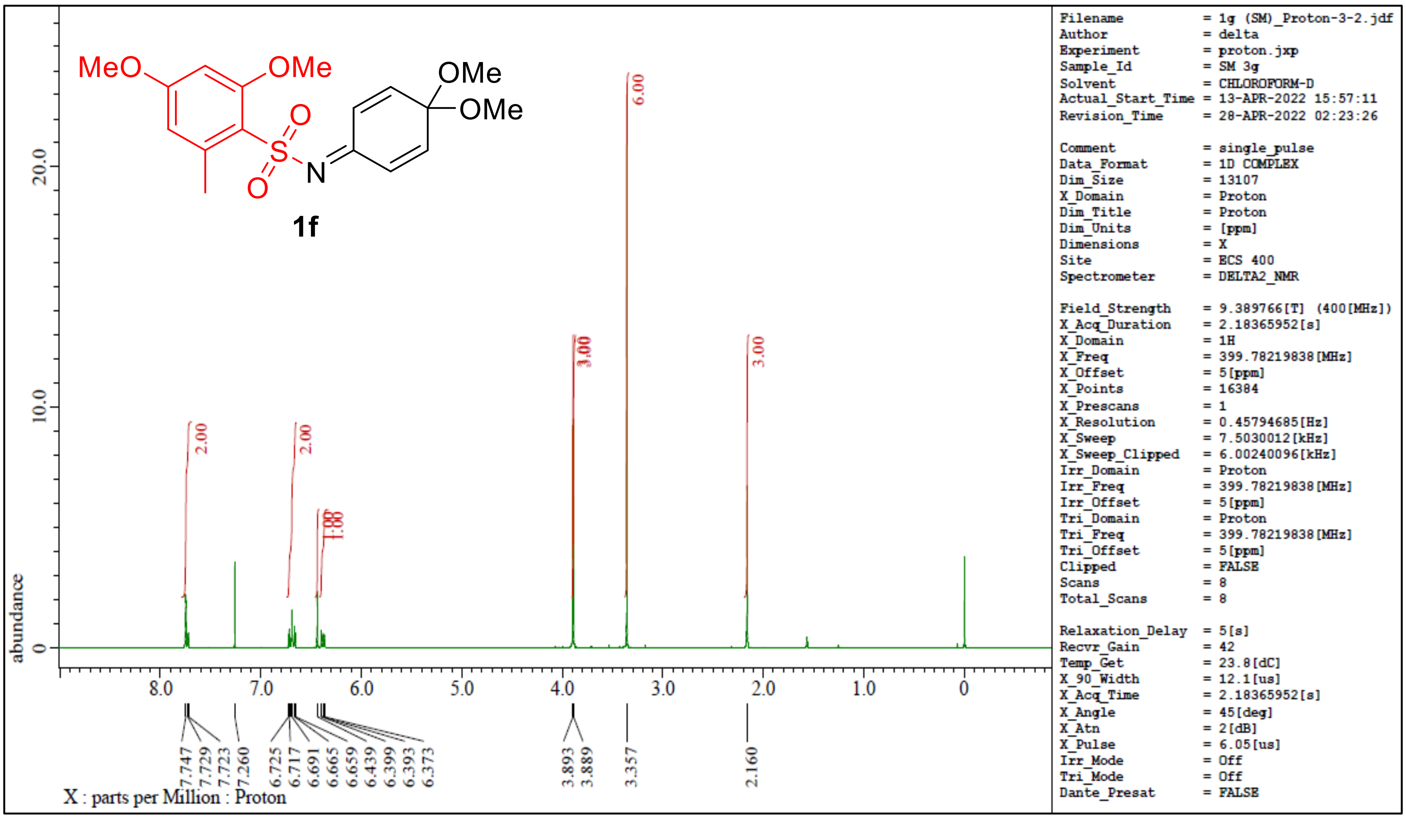

Compound **1f** (^{1}H NMR, 400 MHz, $CDCl_3$).

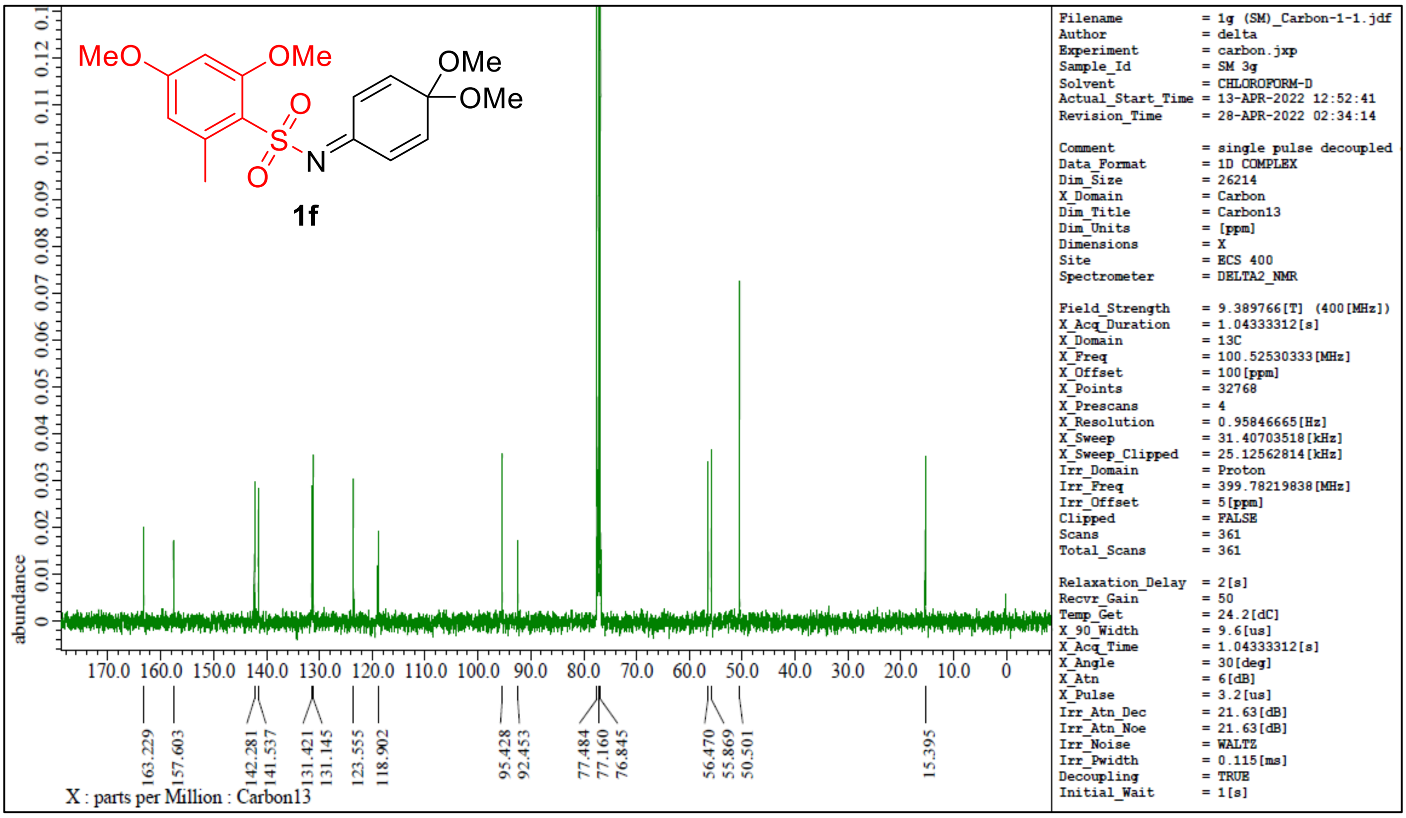

Compound **1f** (^{13}C NMR, 100 MHz, $CDCl_3$).

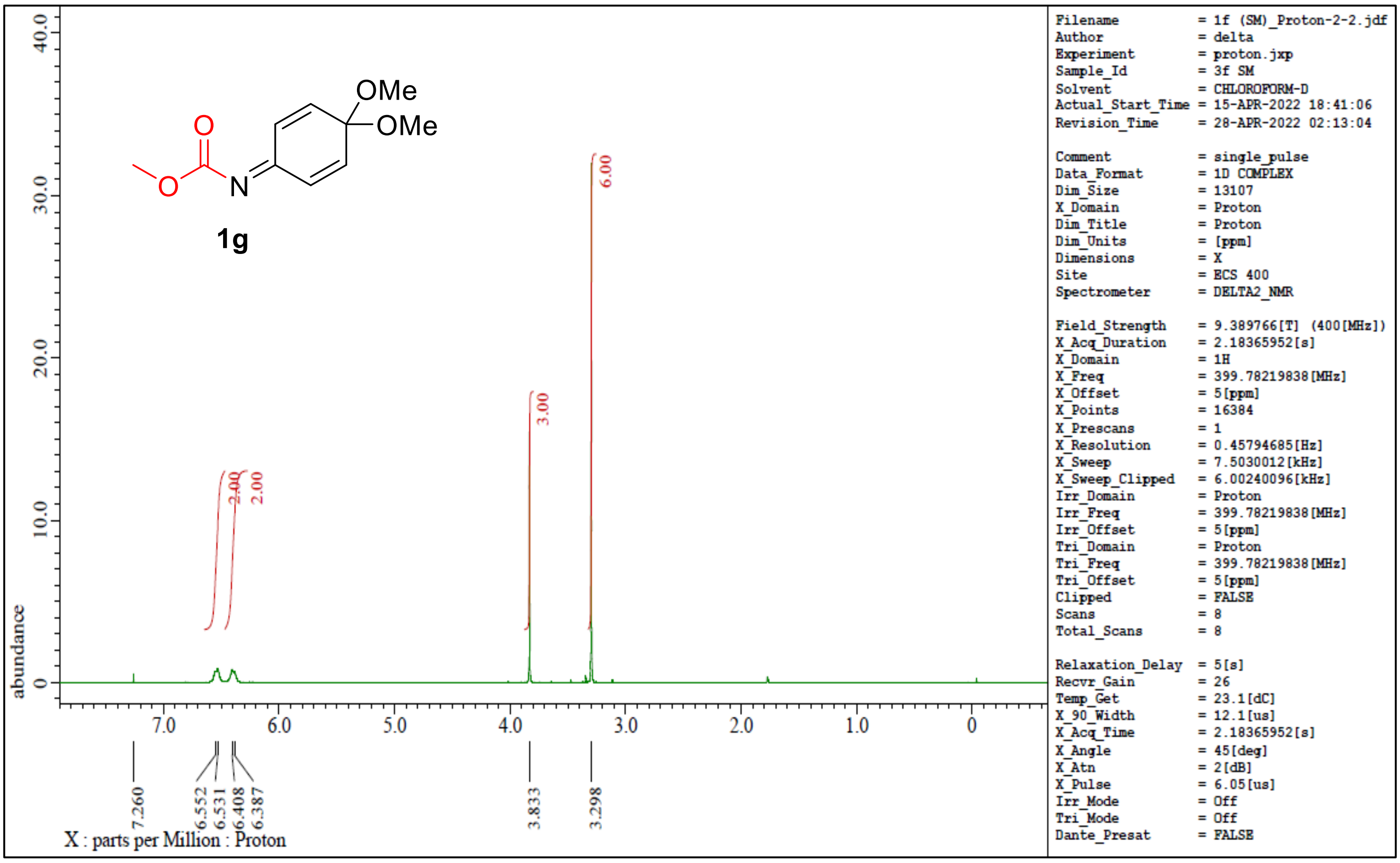

Compound **1g** (^{1}H NMR, 400 MHz, $CDCl_3$).

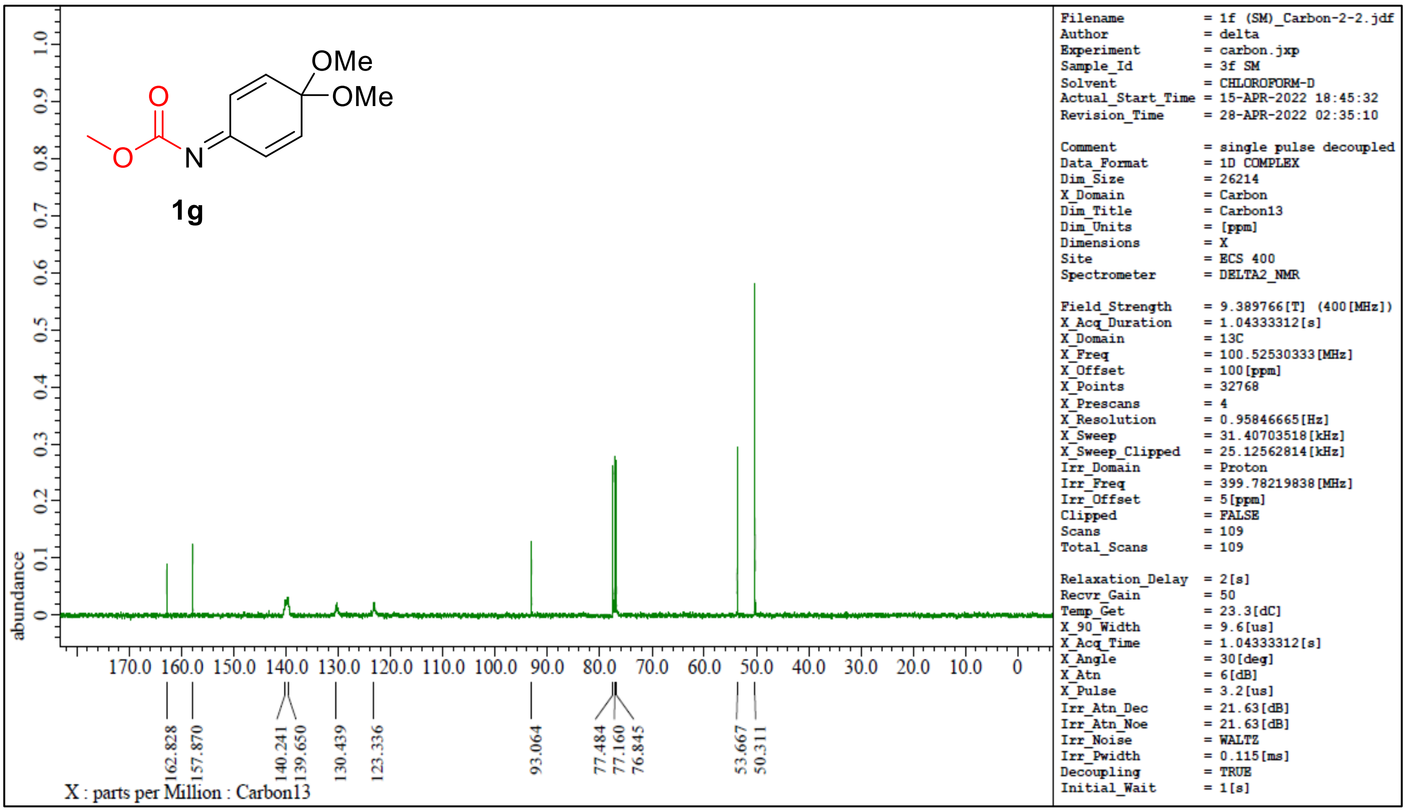

Compound **1g** (^{13}C NMR, 100 MHz, $CDCl_3$).

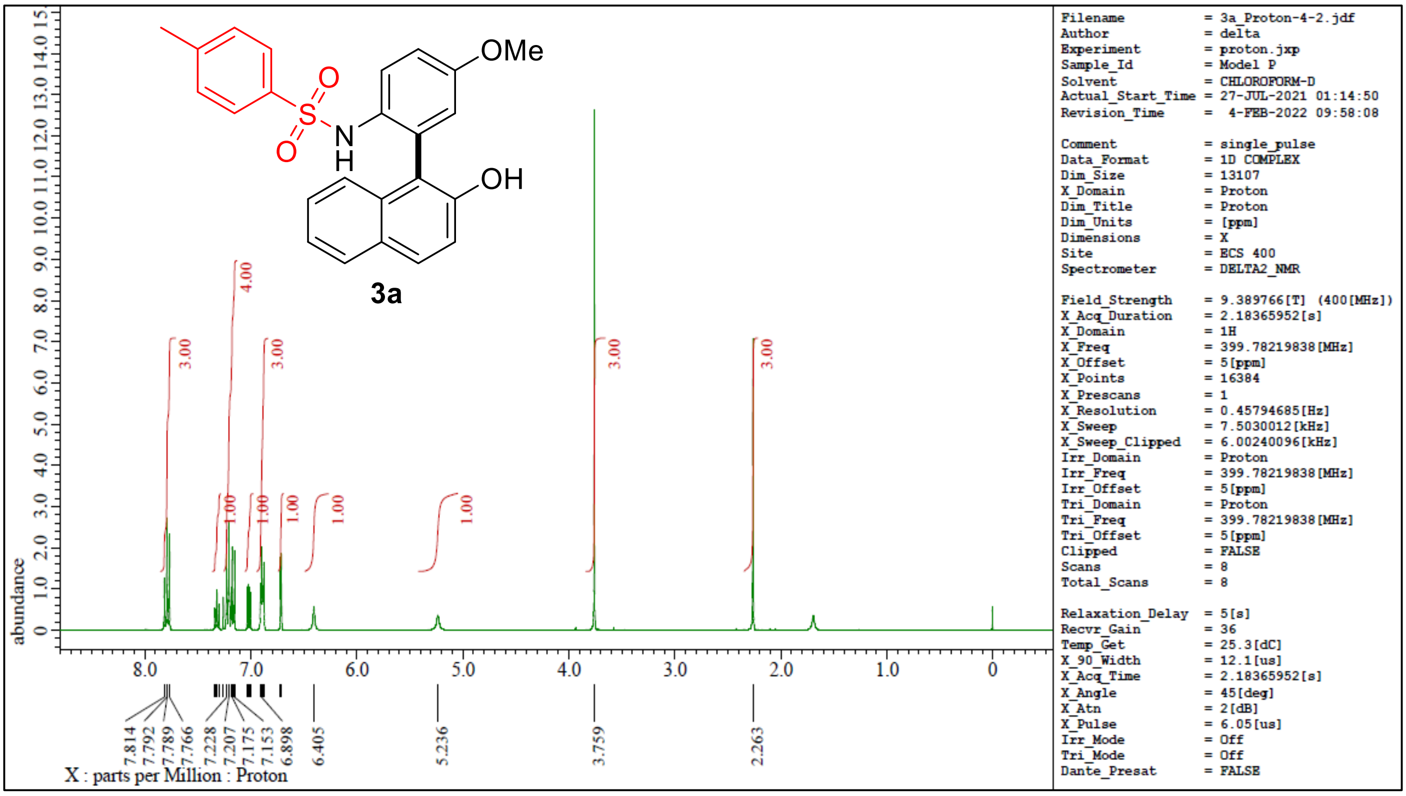

Compound **3a** (^{1}H NMR, 400 MHz, $CDCl_3$).

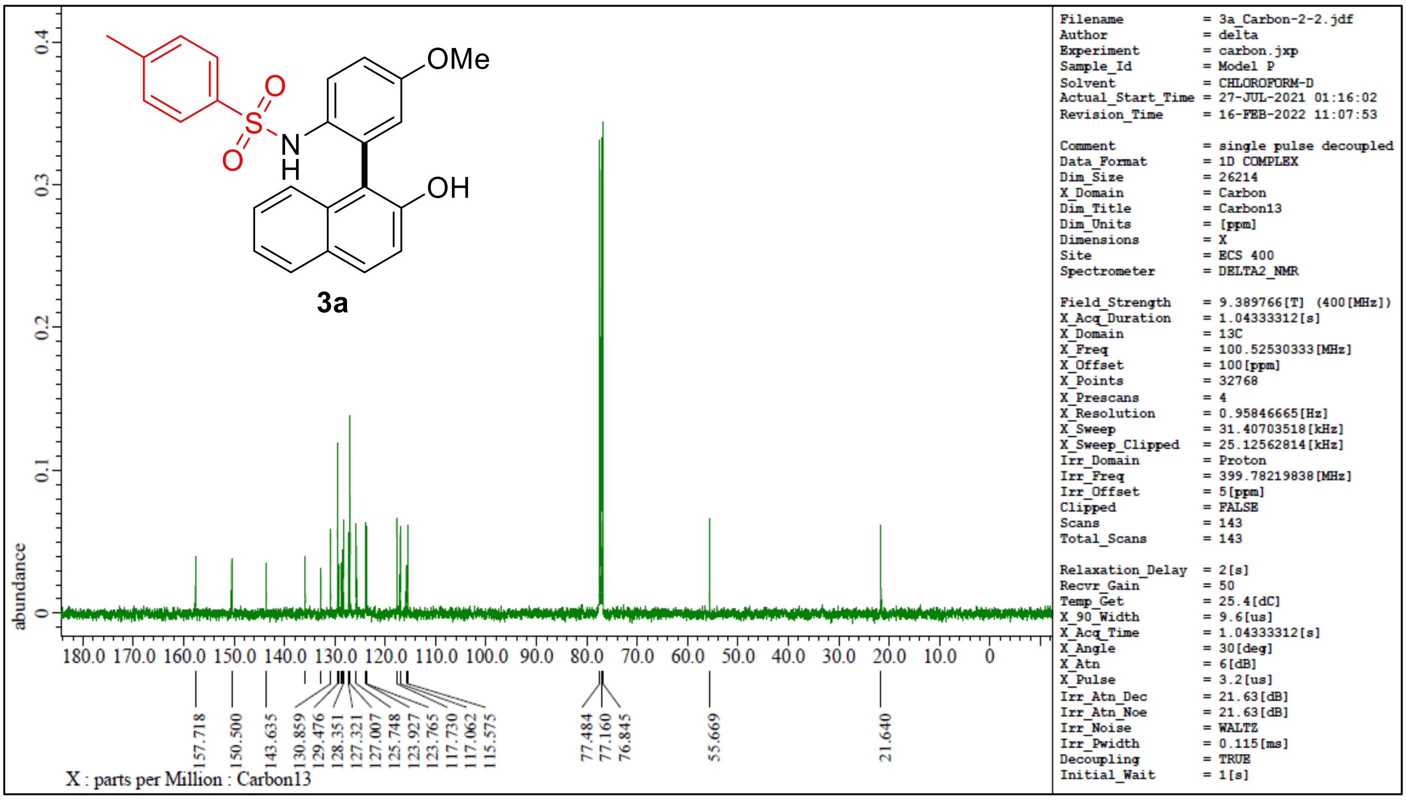

Compound **3a** (^{13}C NMR, 100 MHz, $CDCl_3$).

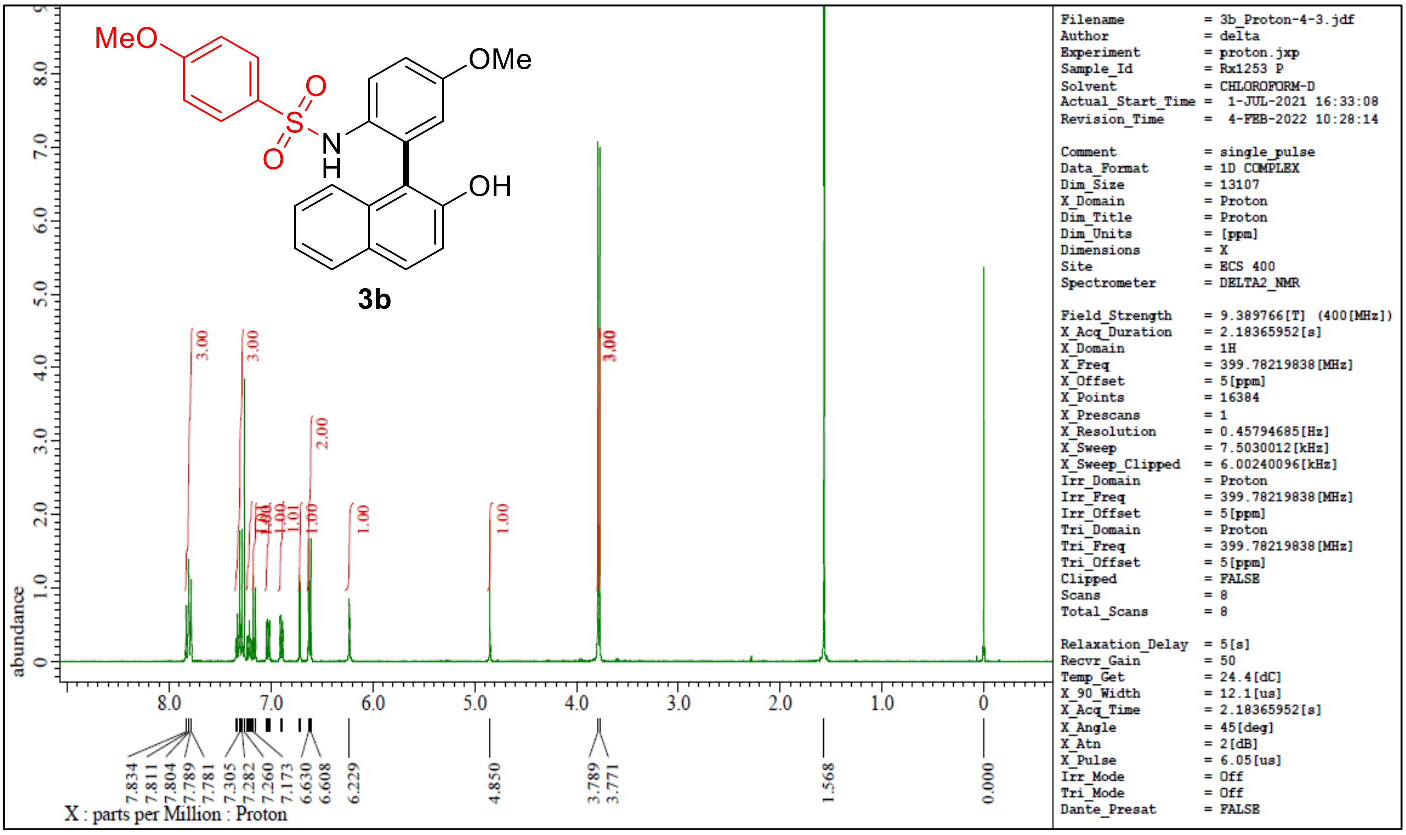

Compound **3b** (^{1}H NMR, 400 MHz, $CDCl_3$).

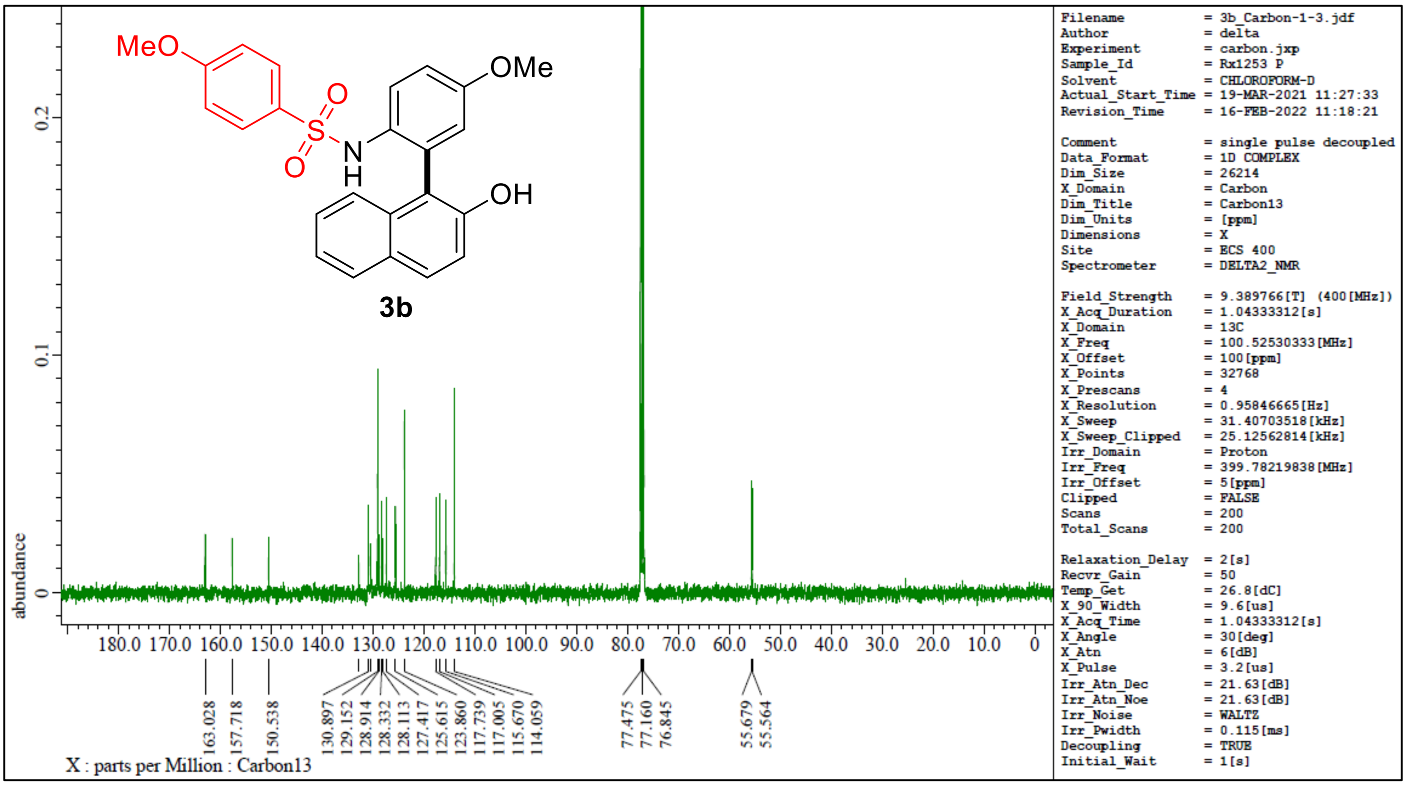

Compound **3b** (^{13}C NMR, 100 MHz, $CDCl_3$).

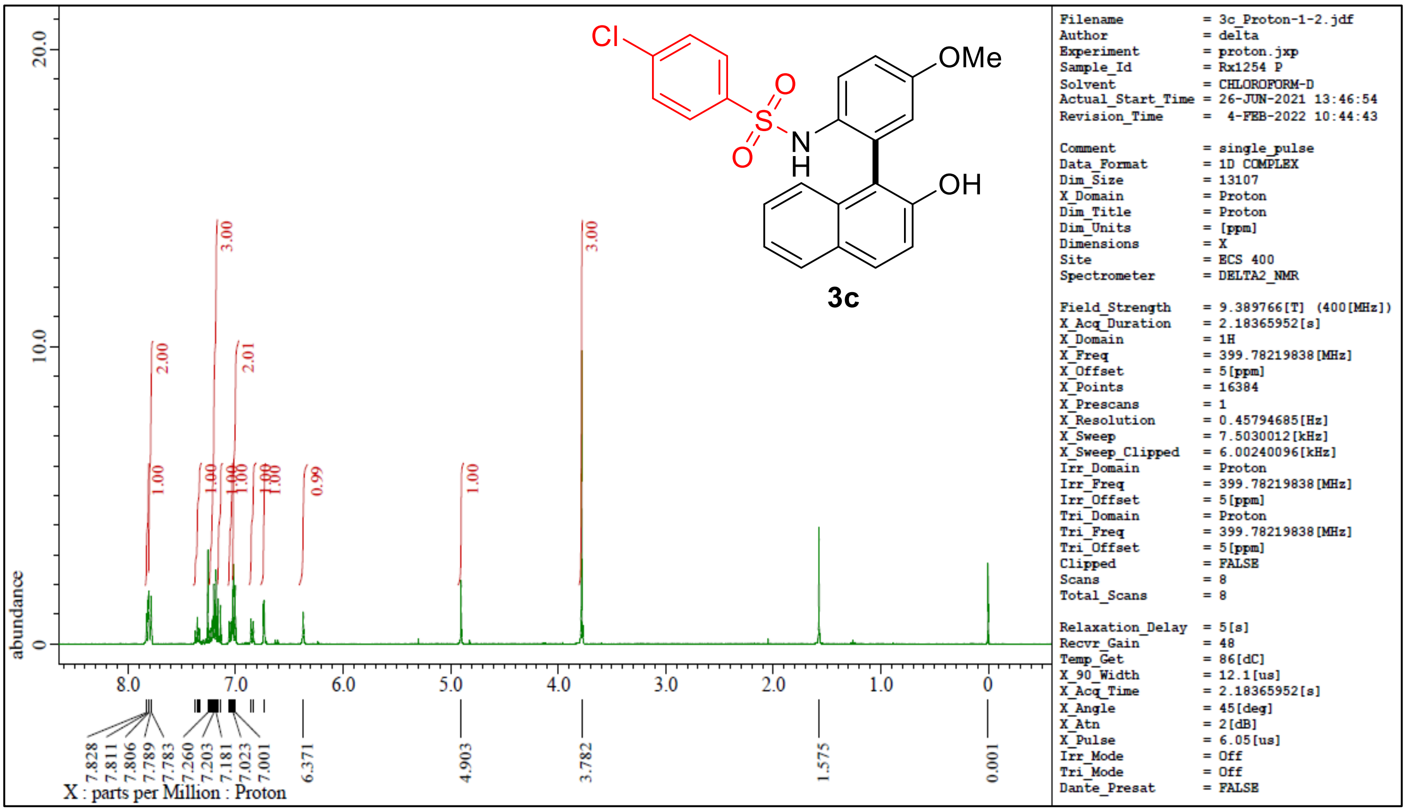

Compound **3c** (^{1}H NMR, 400 MHz, $CDCl_3$).

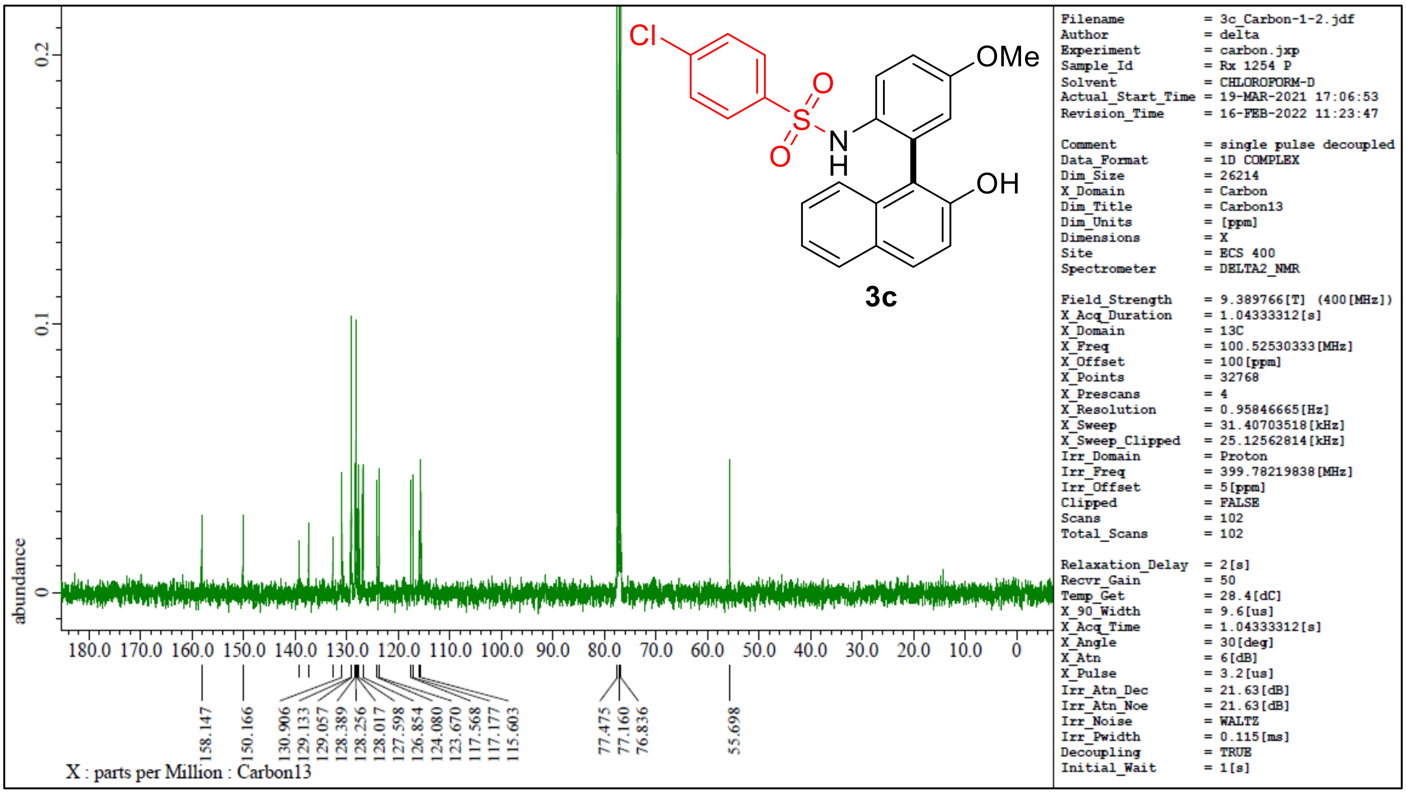

Compound **3c** (^{13}C NMR, 100 MHz, $CDCl_3$).

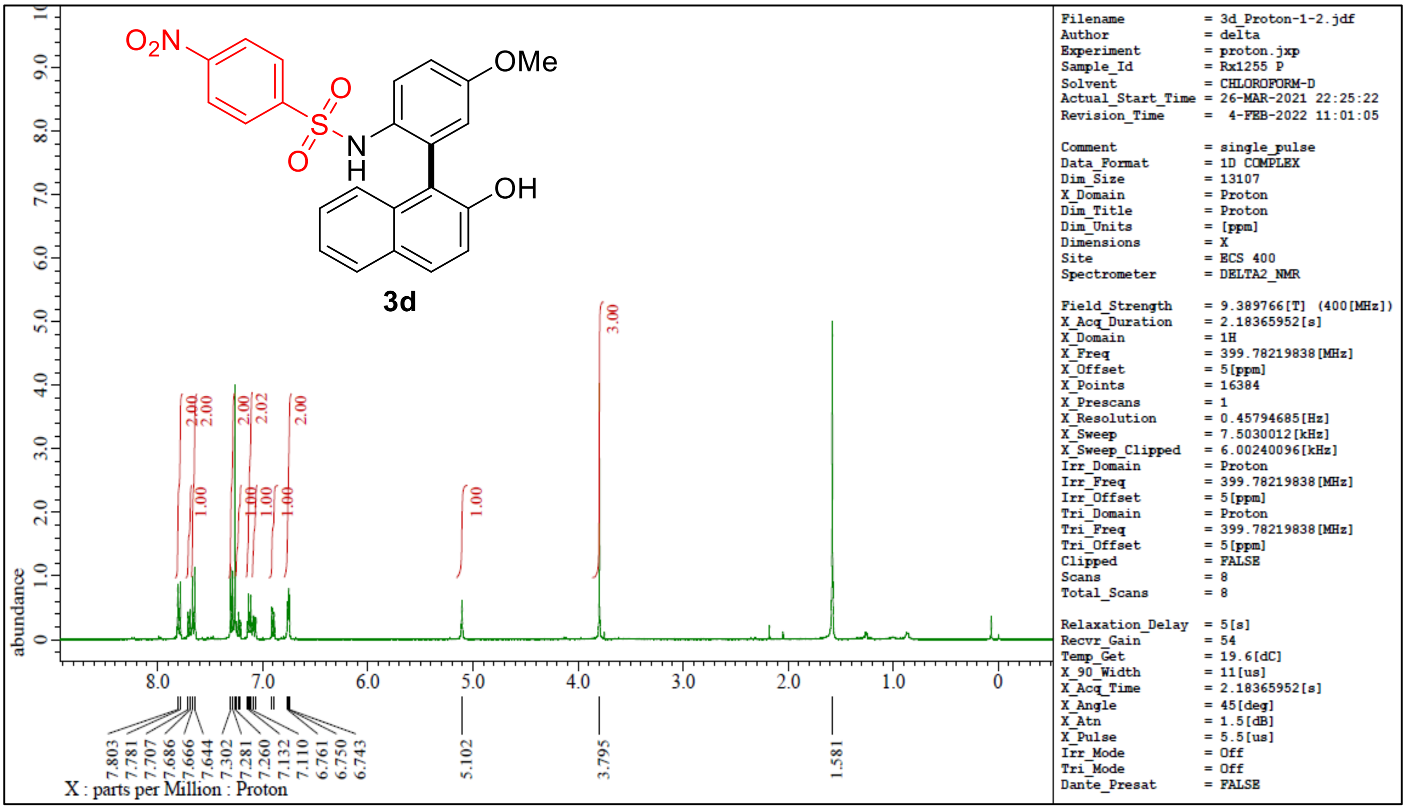

Compound **3d** (^{1}H NMR, 400 MHz, $CDCl_3$).

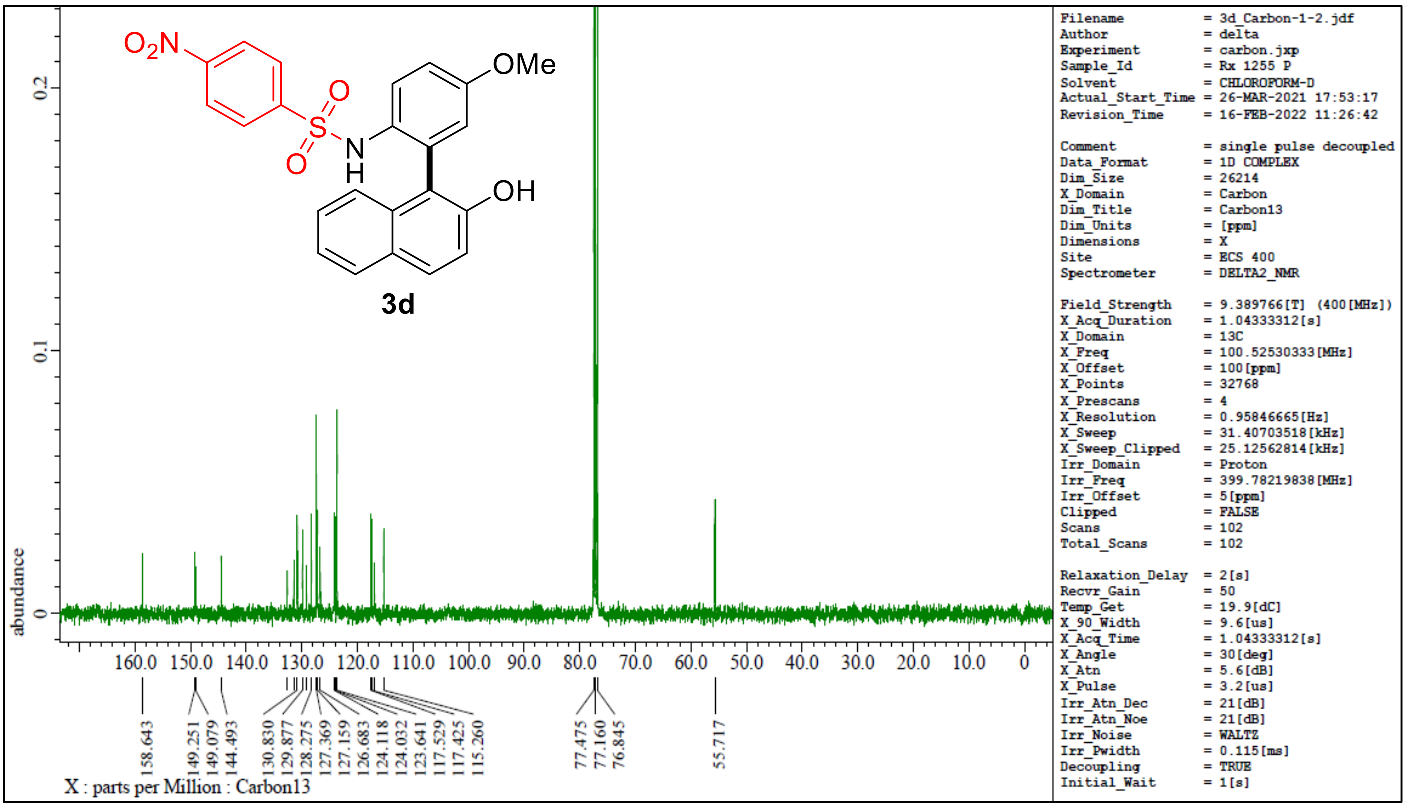

Compound **3d** (^{13}C NMR, 100 MHz, $CDCl_3$).

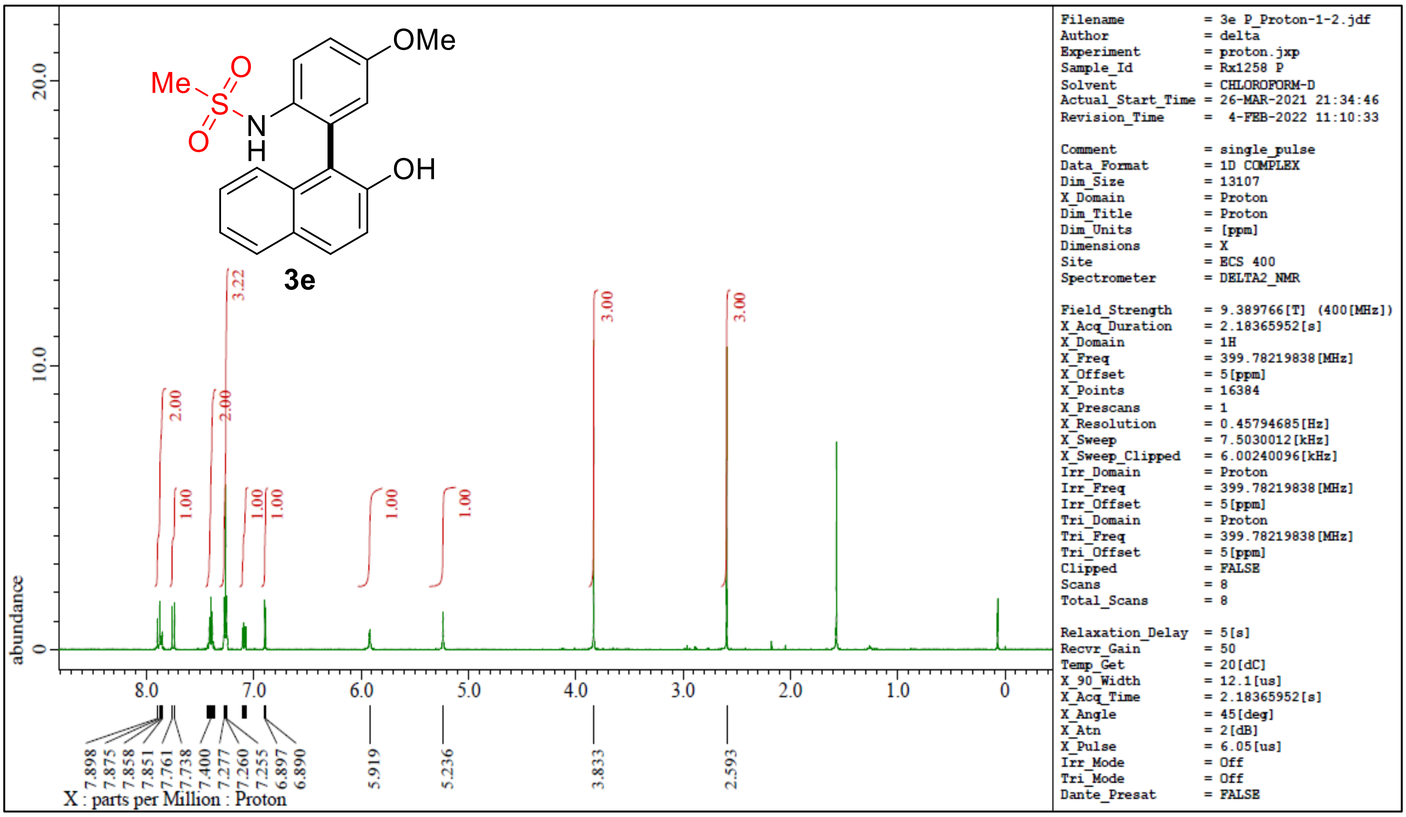

Compound **3e** (^{1}H NMR, 400 MHz, $CDCl_3$).

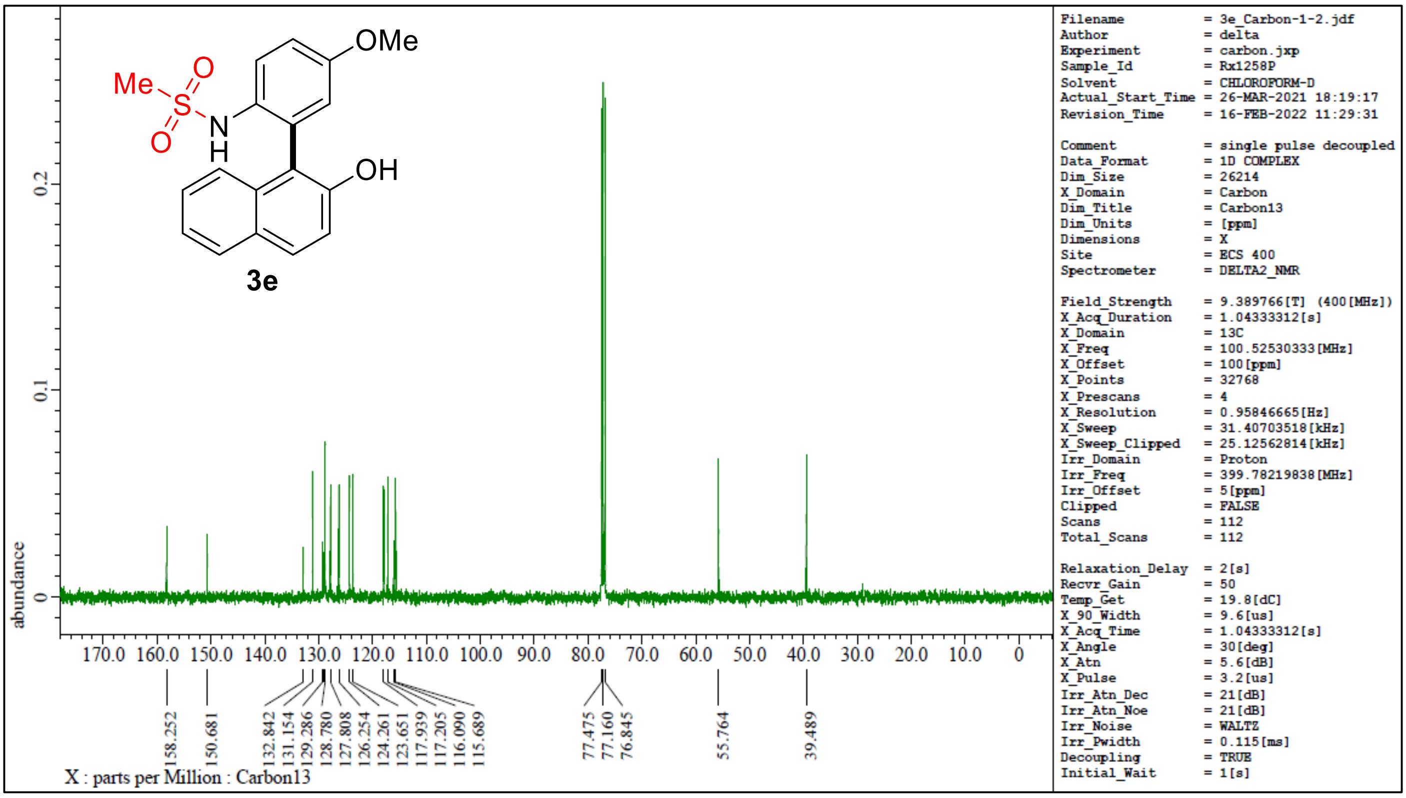

Compound **3e** (^{13}C NMR, 100 MHz, $CDCl_3$).

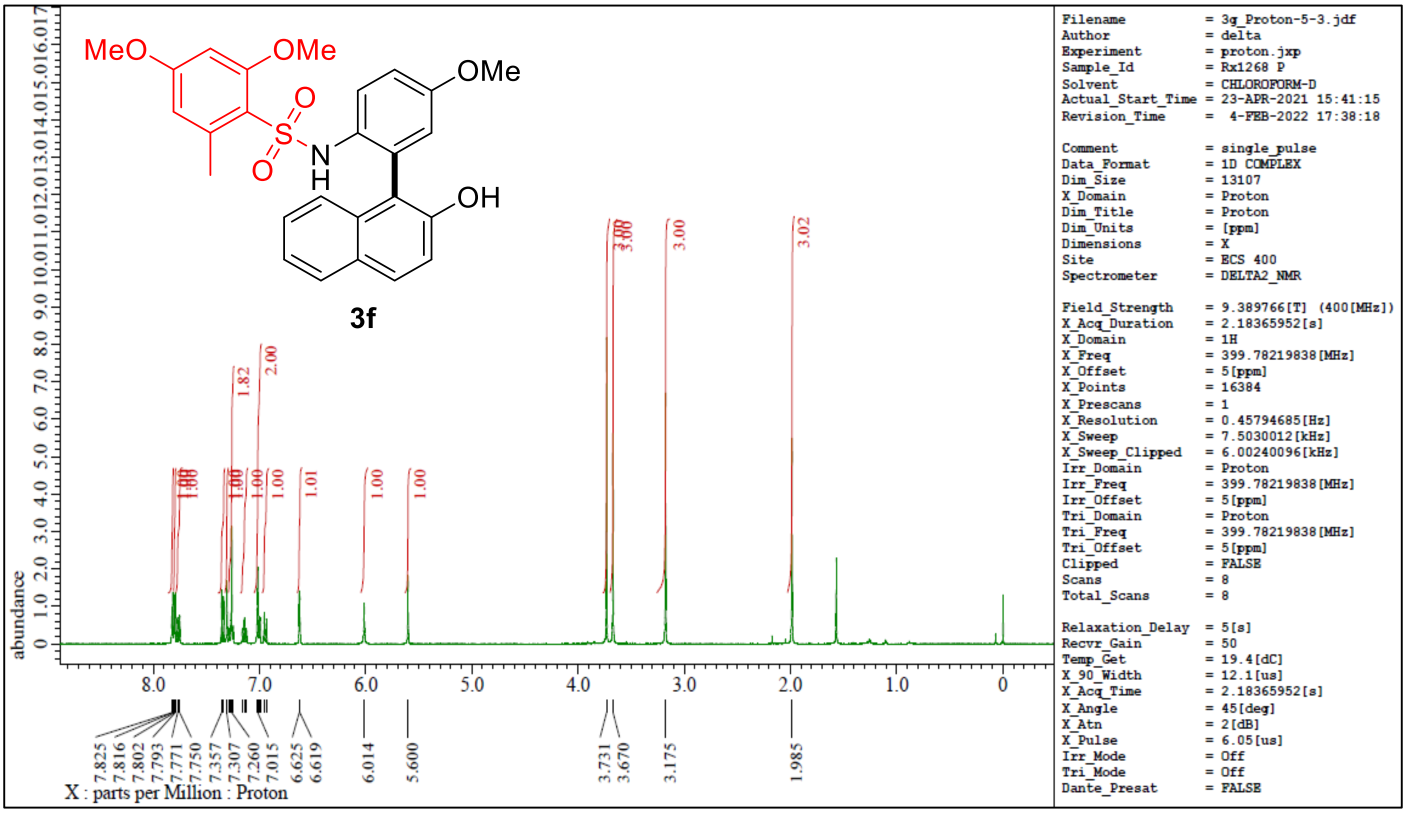

Compound **3f** (^{1}H NMR, 400 MHz, $CDCl_3$).

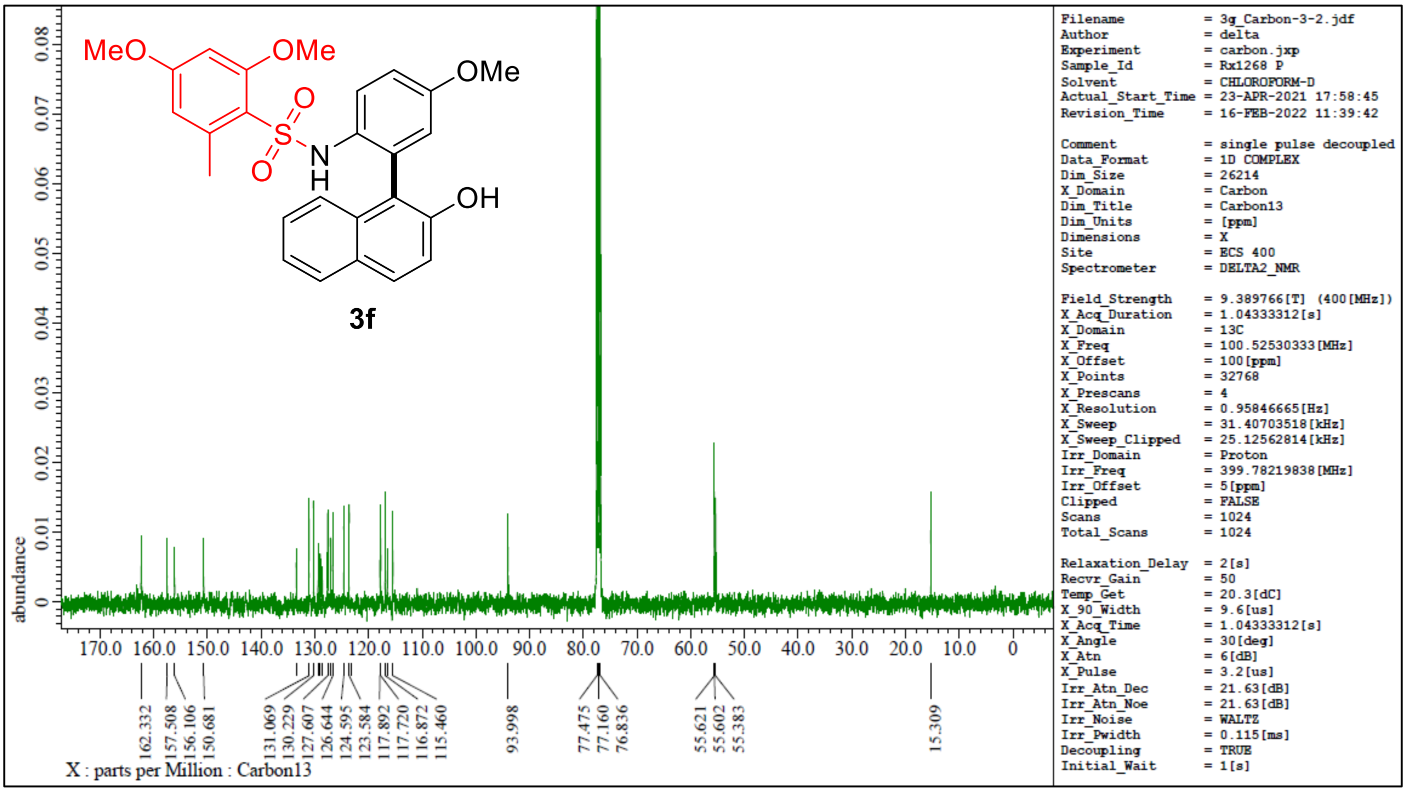

Compound **3f** (^{13}C NMR, 100 MHz, $CDCl_3$).

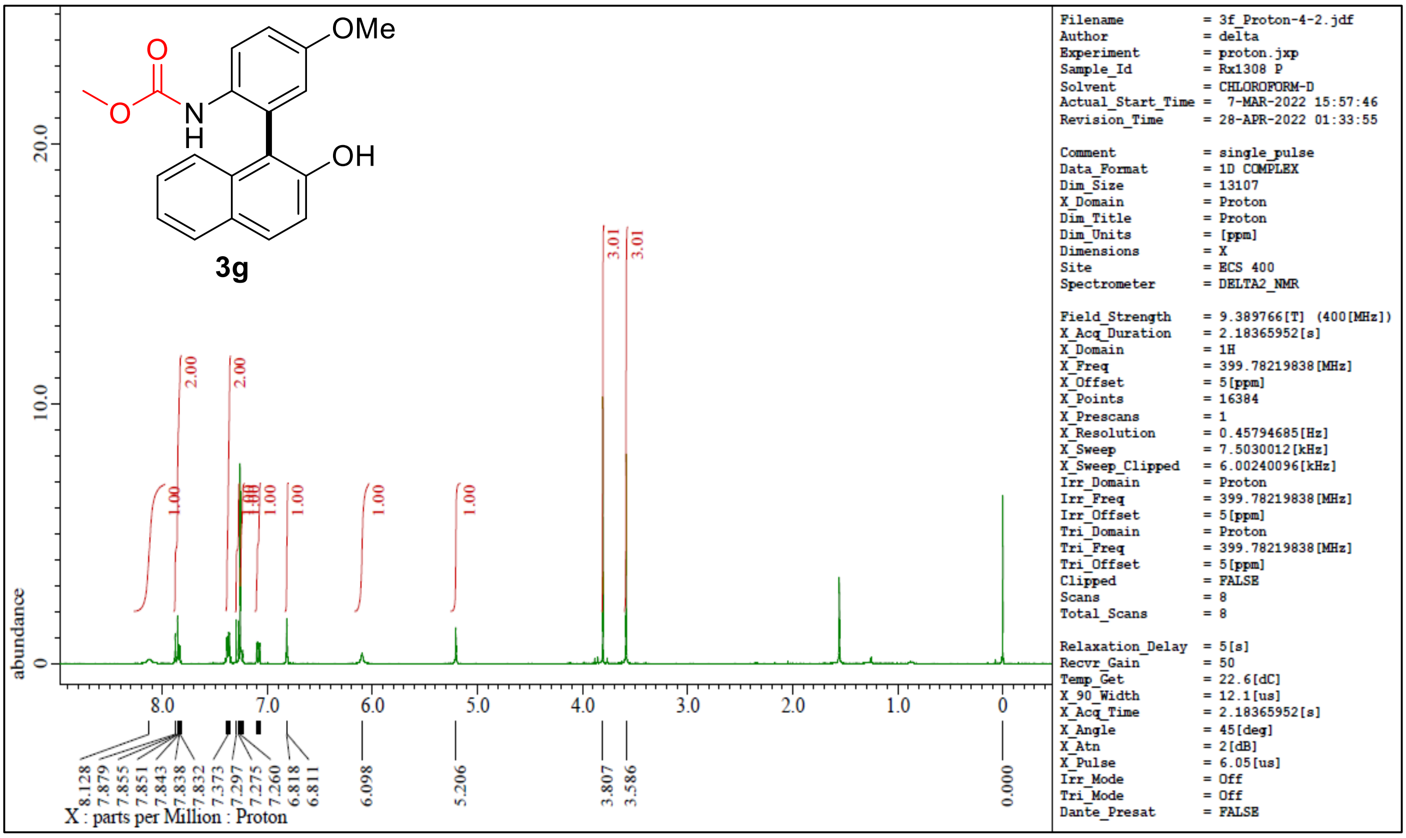

Compound **3g** (^{1}H NMR, 400 MHz, $CDCl_3$).

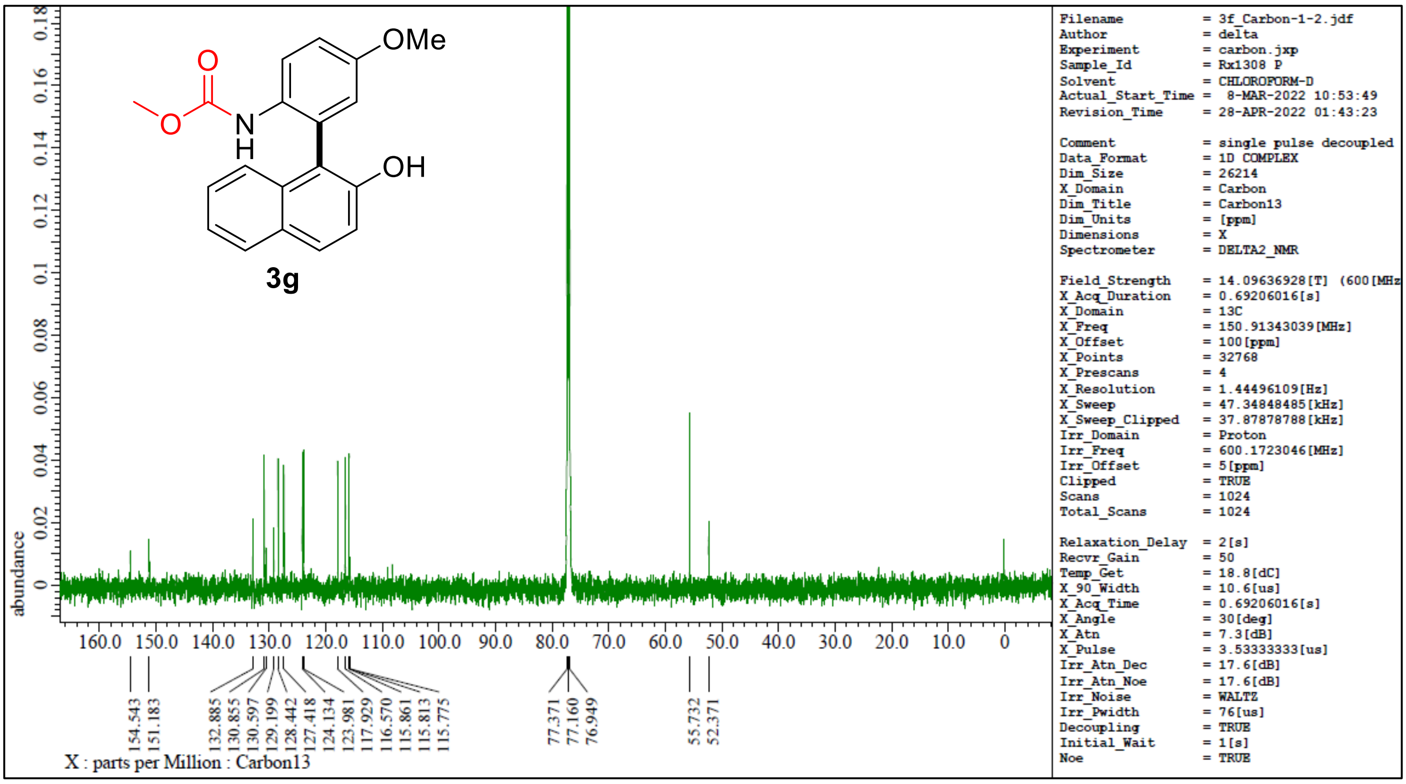

Compound **3g** (^{13}C NMR, 150 MHz, $CDCl_3$).

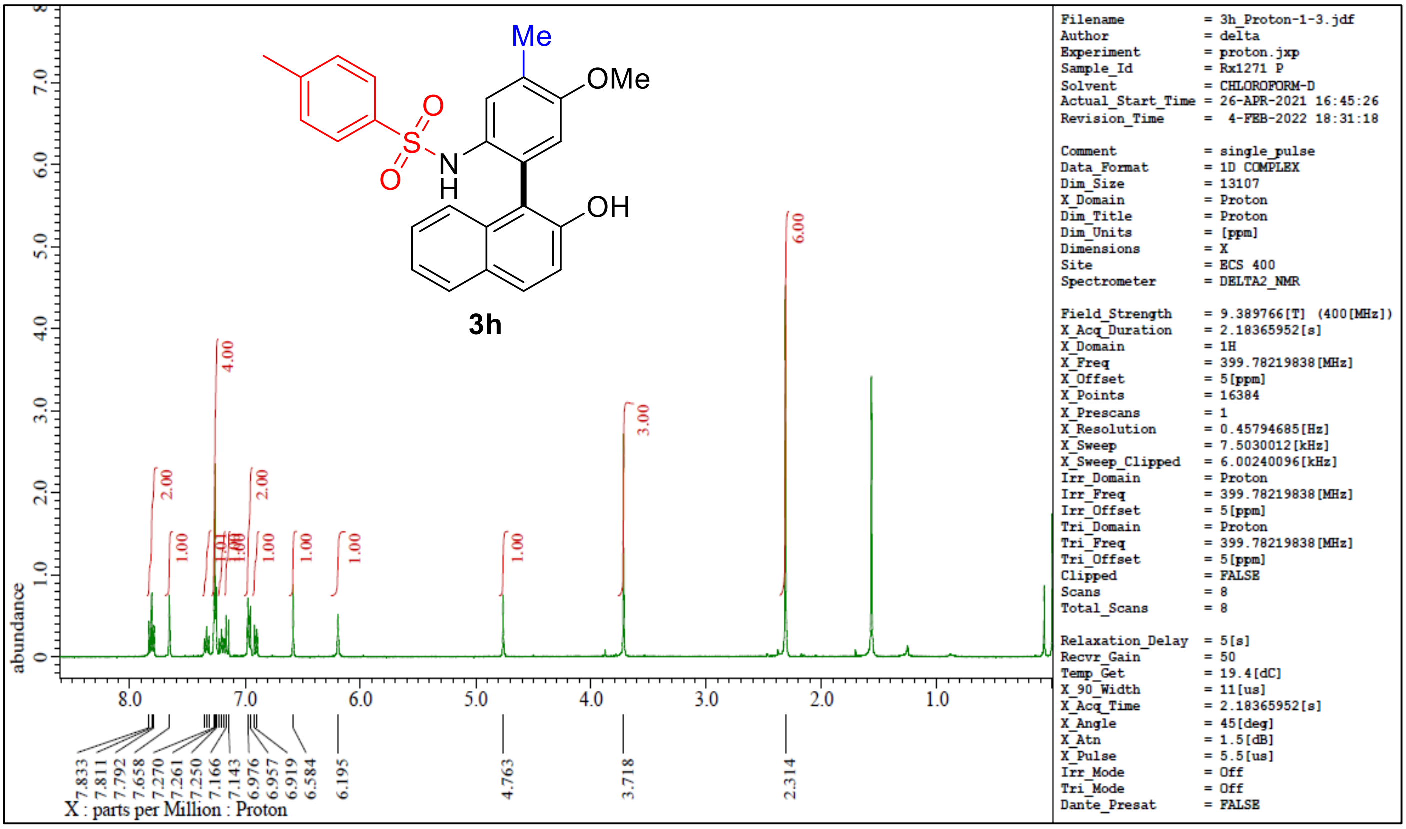

Compound **3h** (^{1}H NMR, 400 MHz, $CDCl_3$).

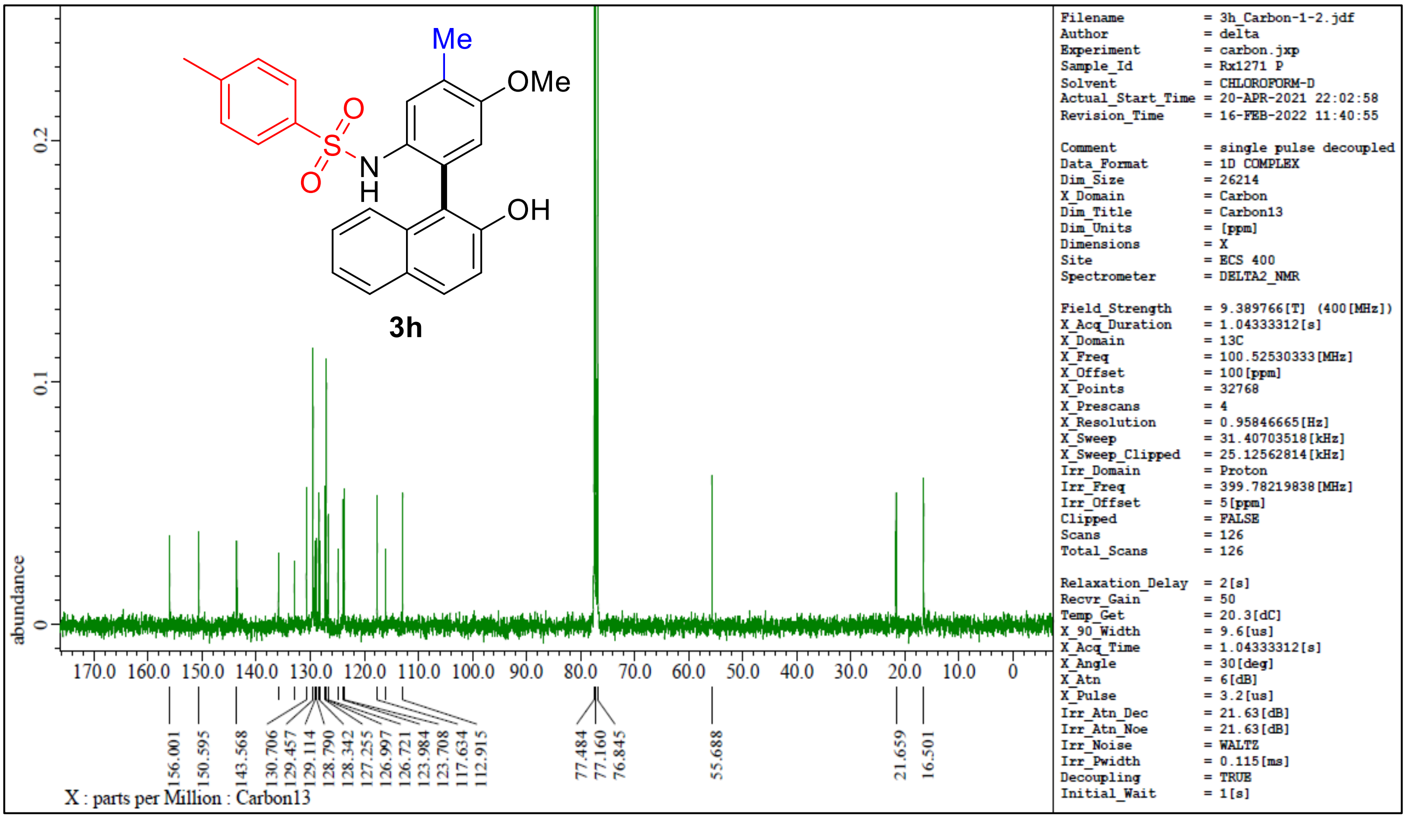

Compound **3h** (^{13}C NMR, 100 MHz, $CDCl_3$).

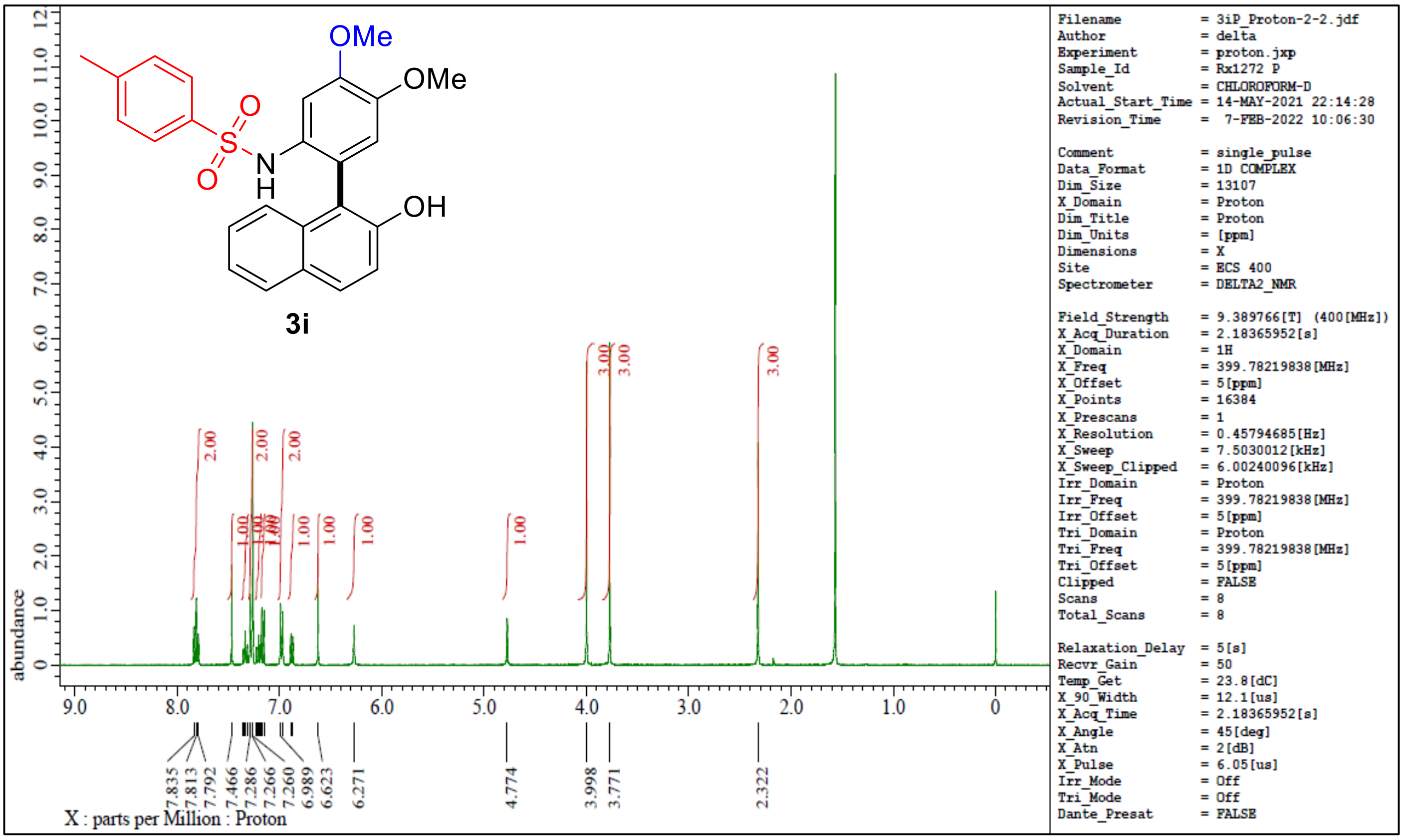

Compound **3i** (^{1}H NMR, 400 MHz, $CDCl_3$).

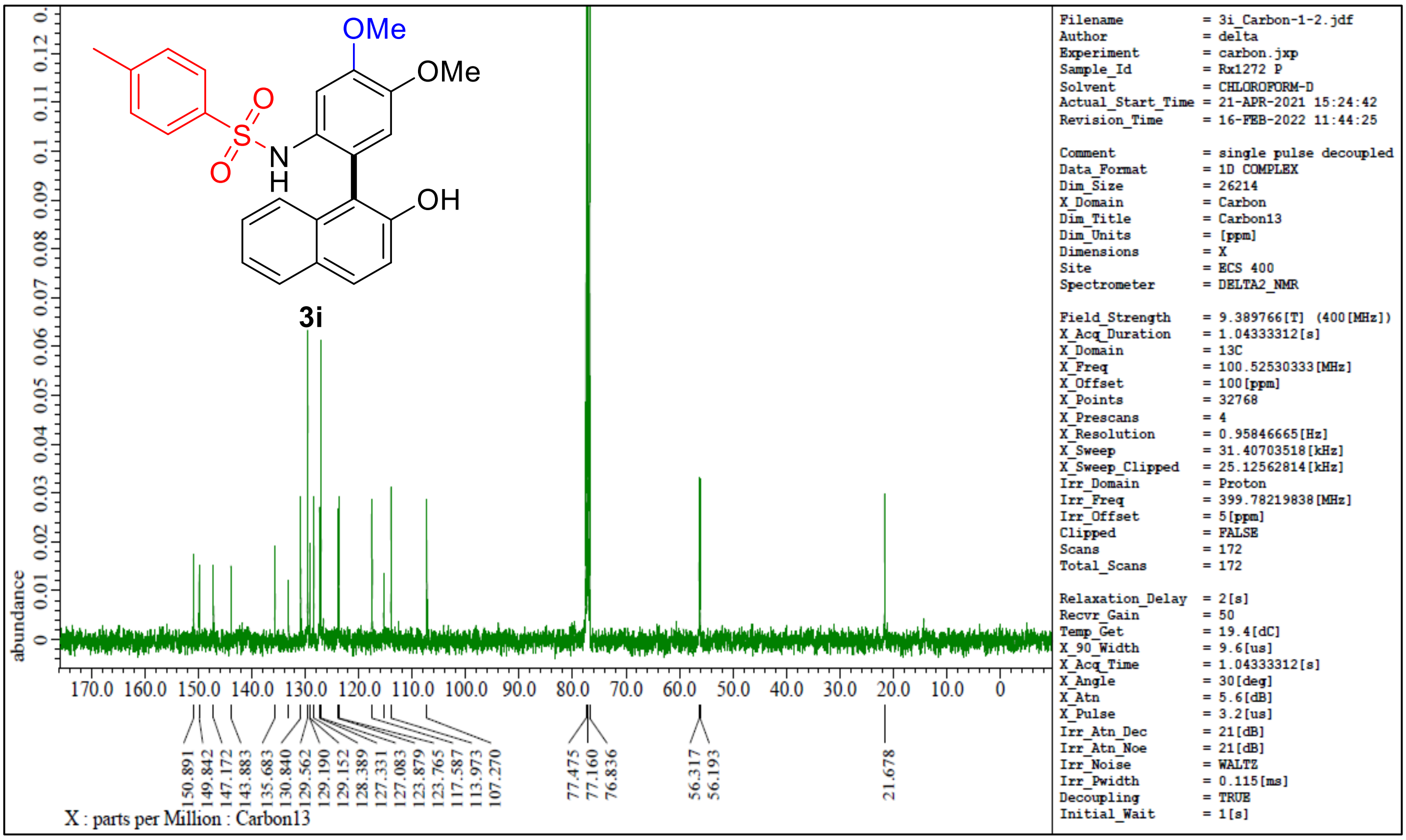

Compound **3i** (^{13}C NMR, 100 MHz, $CDCl_3$).

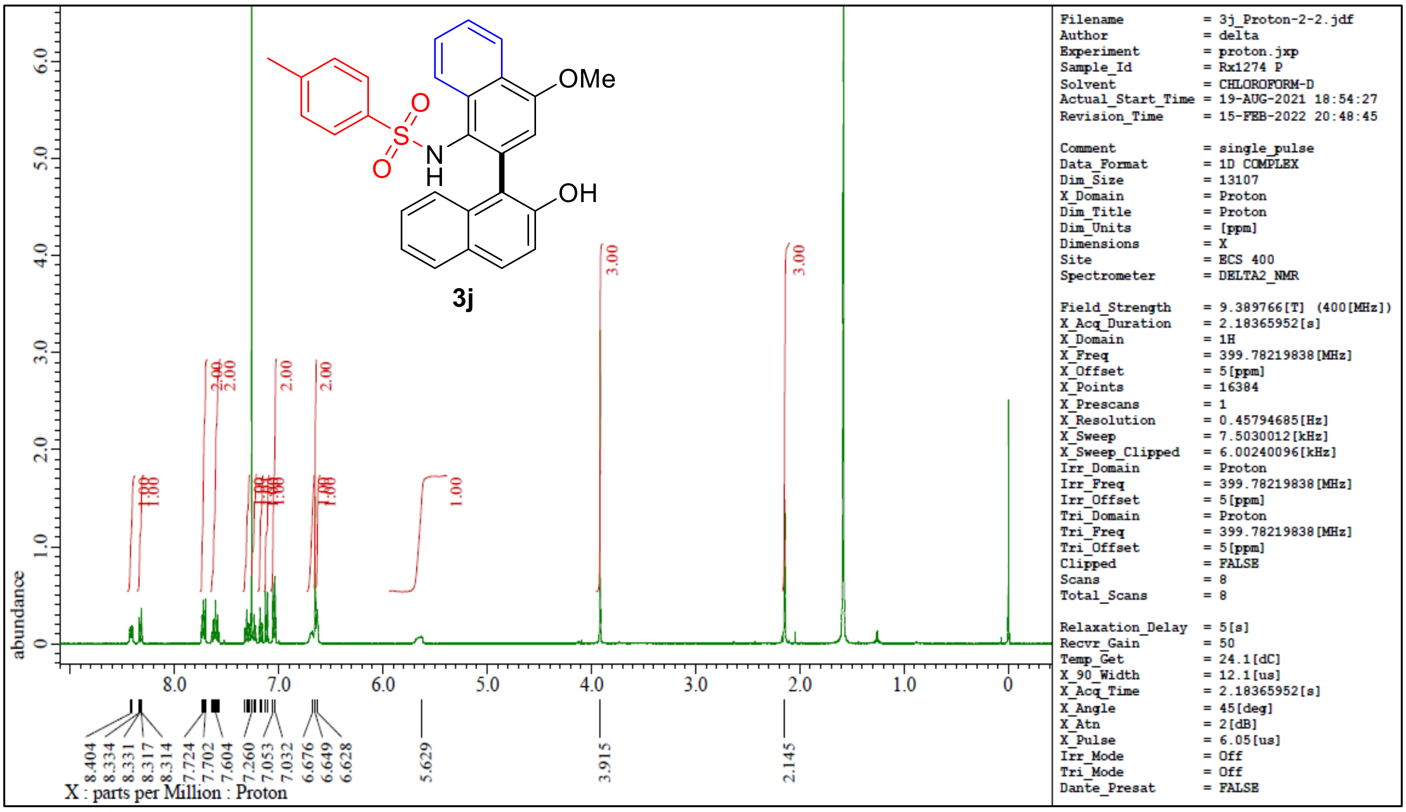

Compound **3j** (^{1}H NMR, 400 MHz, $CDCl_3$).

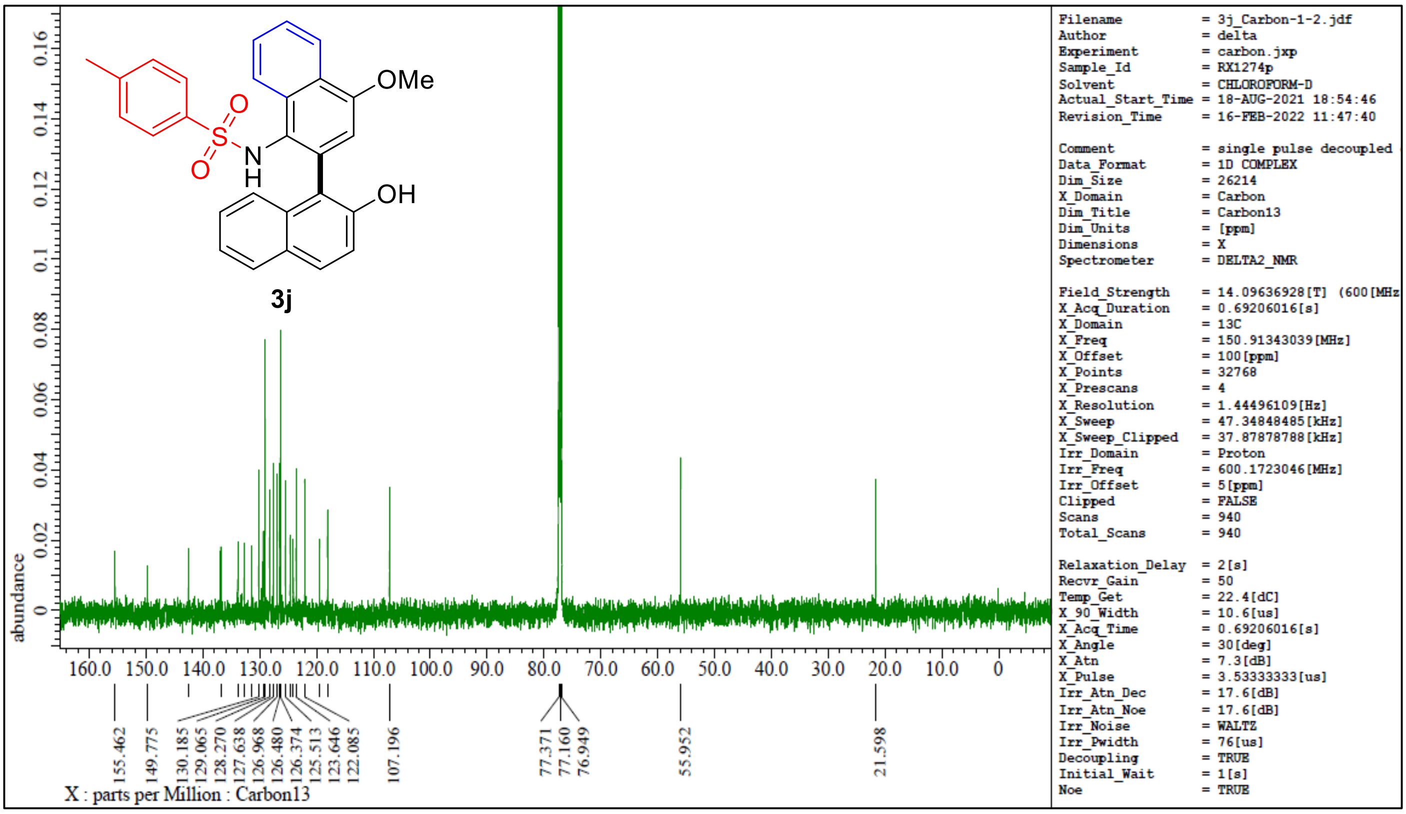

Compound **3j** (^{13}C NMR, 150 MHz, $CDCl_3$).

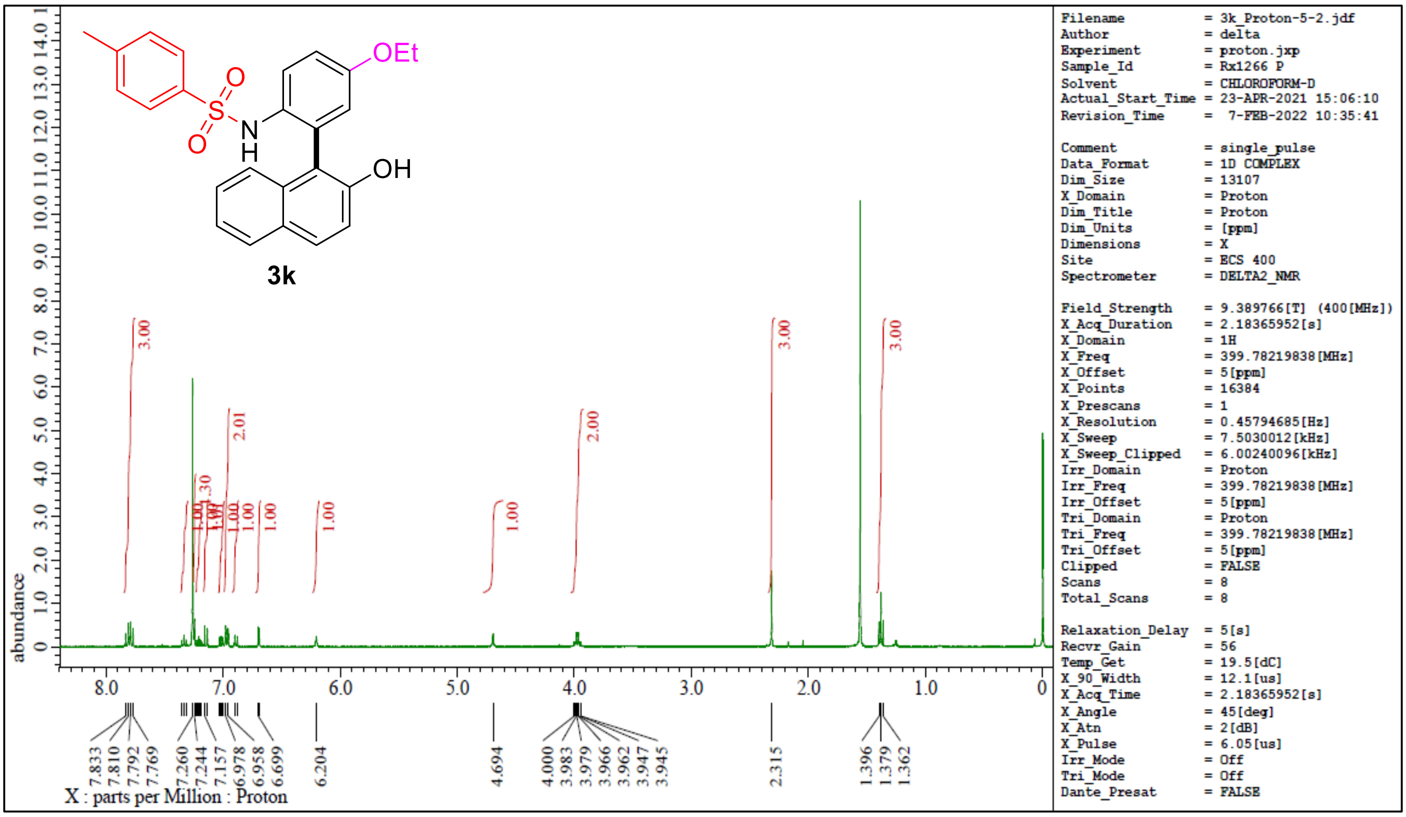

Compound **3k** (^{1}H NMR, 400 MHz, $CDCl_3$).

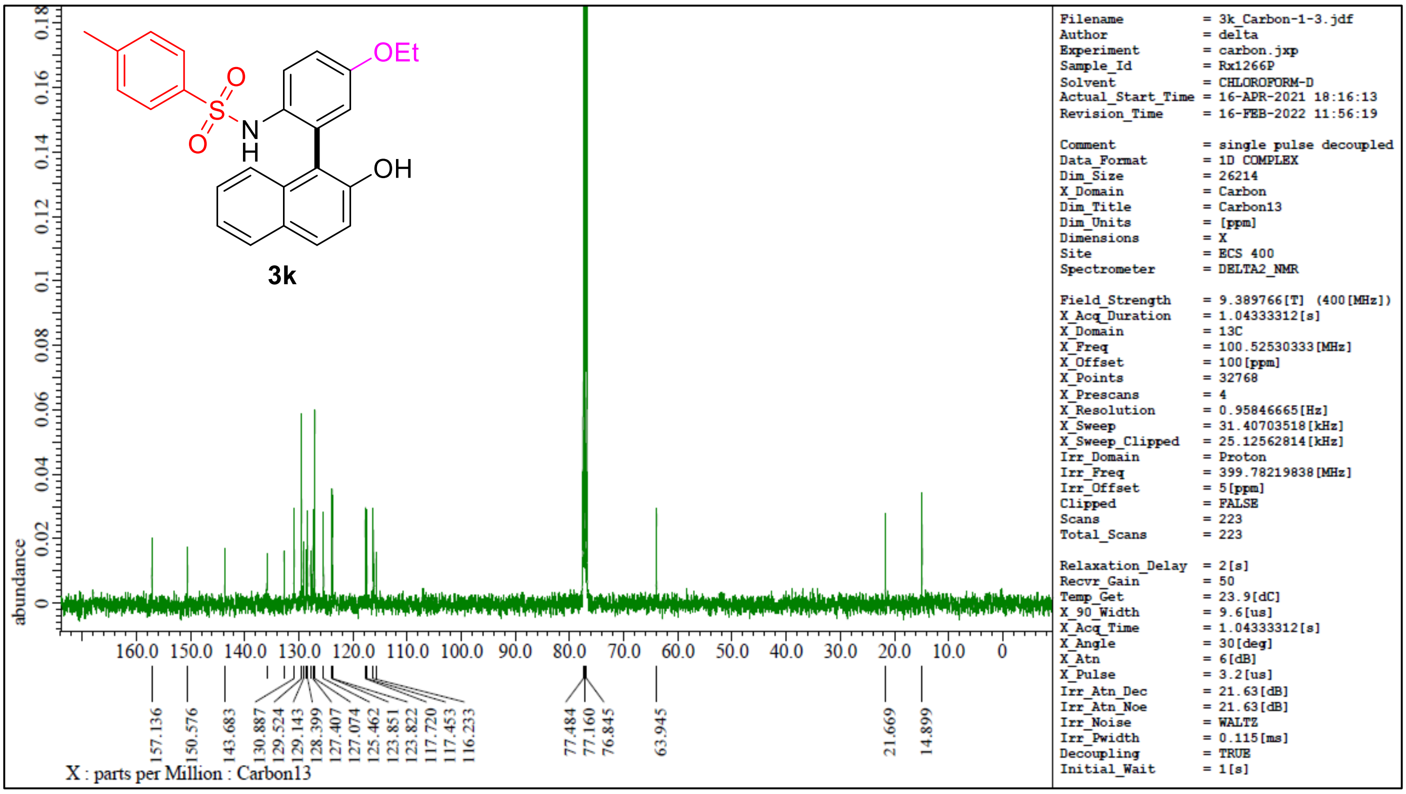

Compound **3k** (^{13}C NMR, 100 MHz, $CDCl_3$).

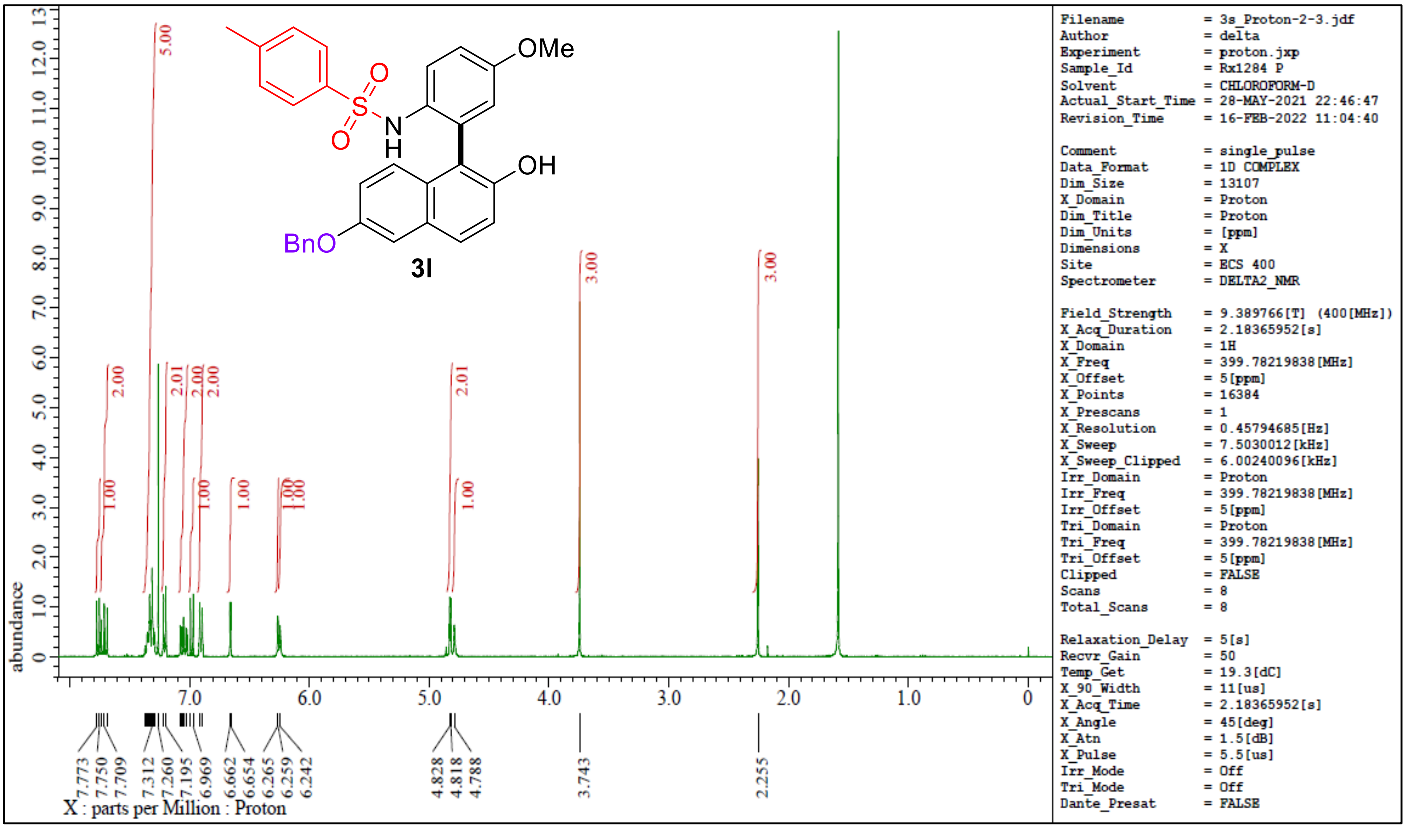

Compound **3l** (^{1}H NMR, 400 MHz, $CDCl_3$).

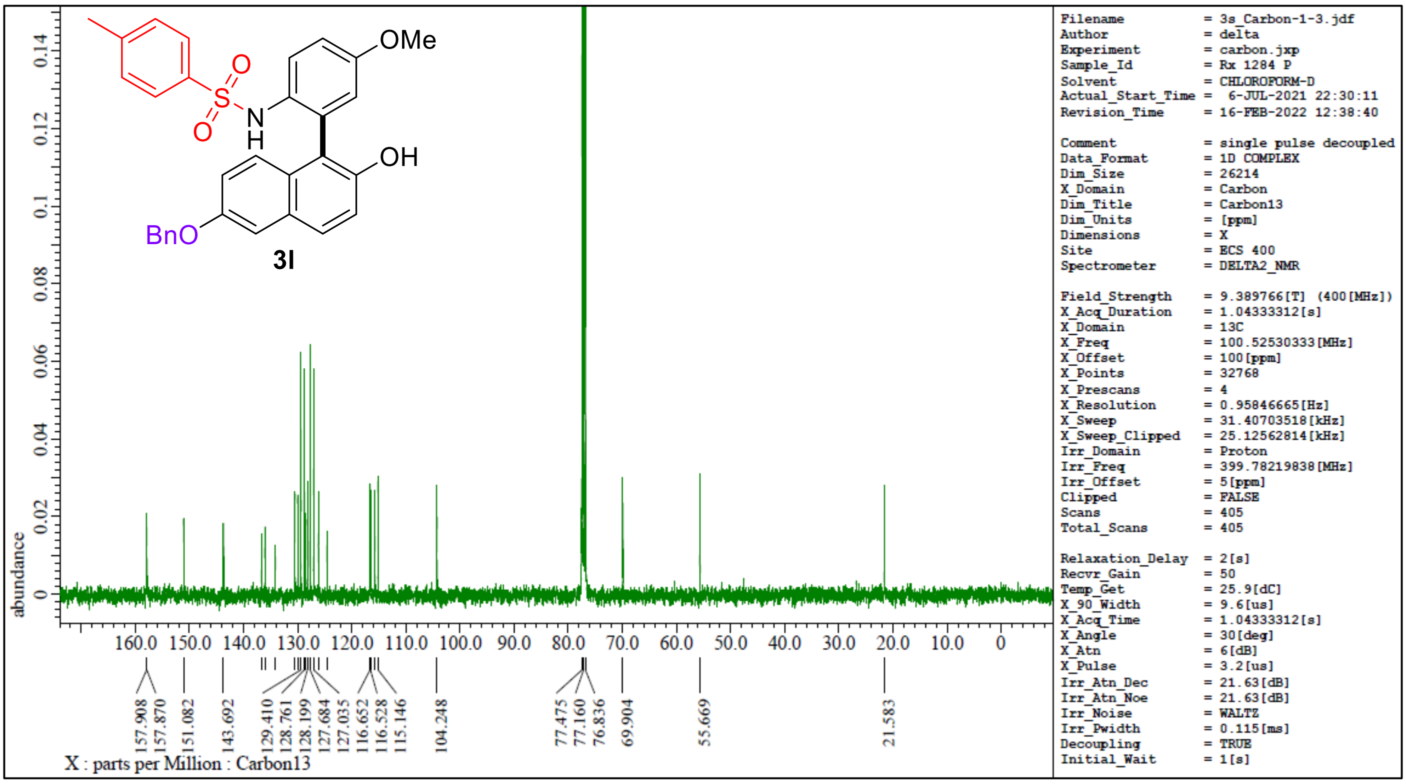

Compound **3l** (^{13}C NMR, 100 MHz, $CDCl_3$).

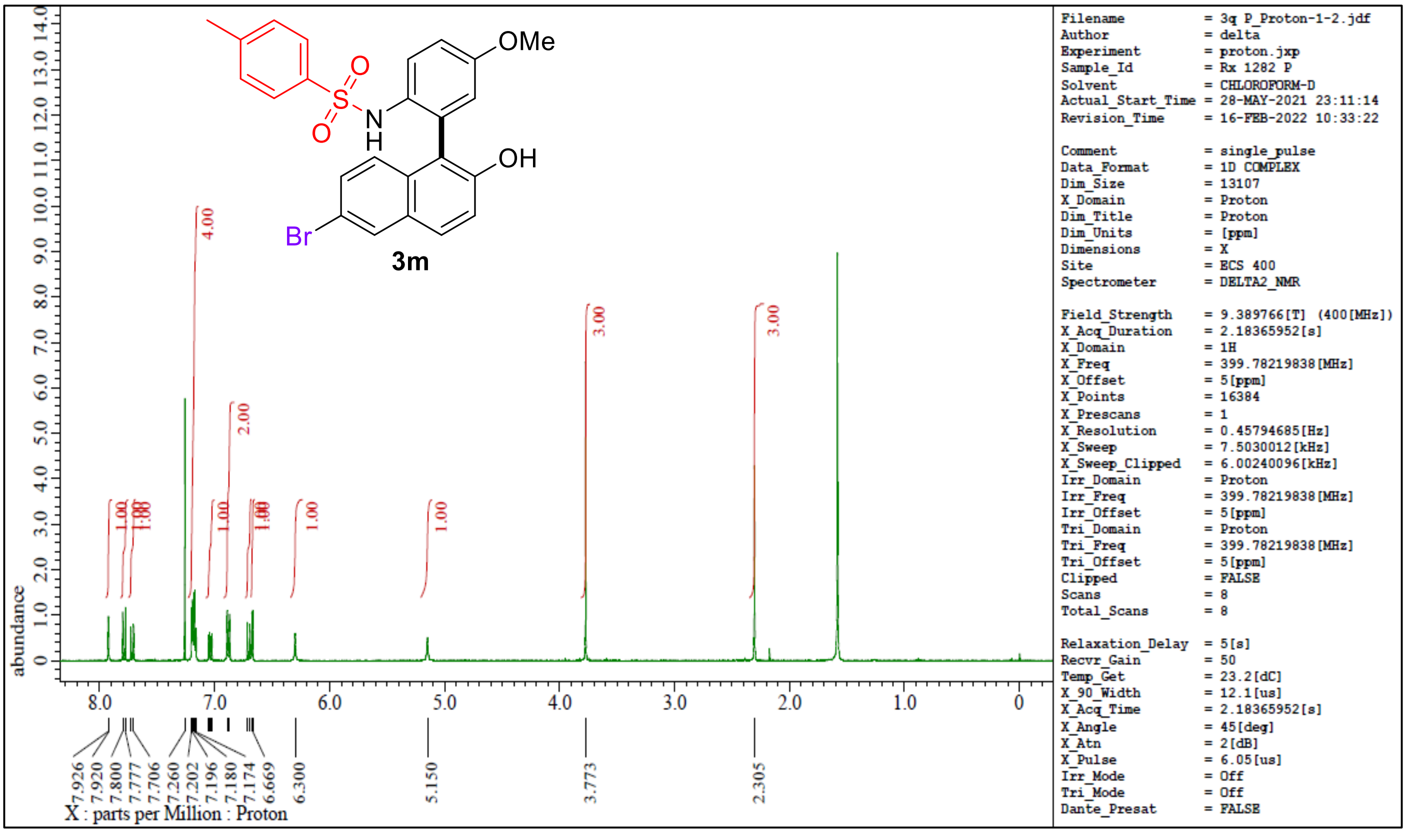

Compound **3m** (^{1}H NMR, 400 MHz, $CDCl_3$).

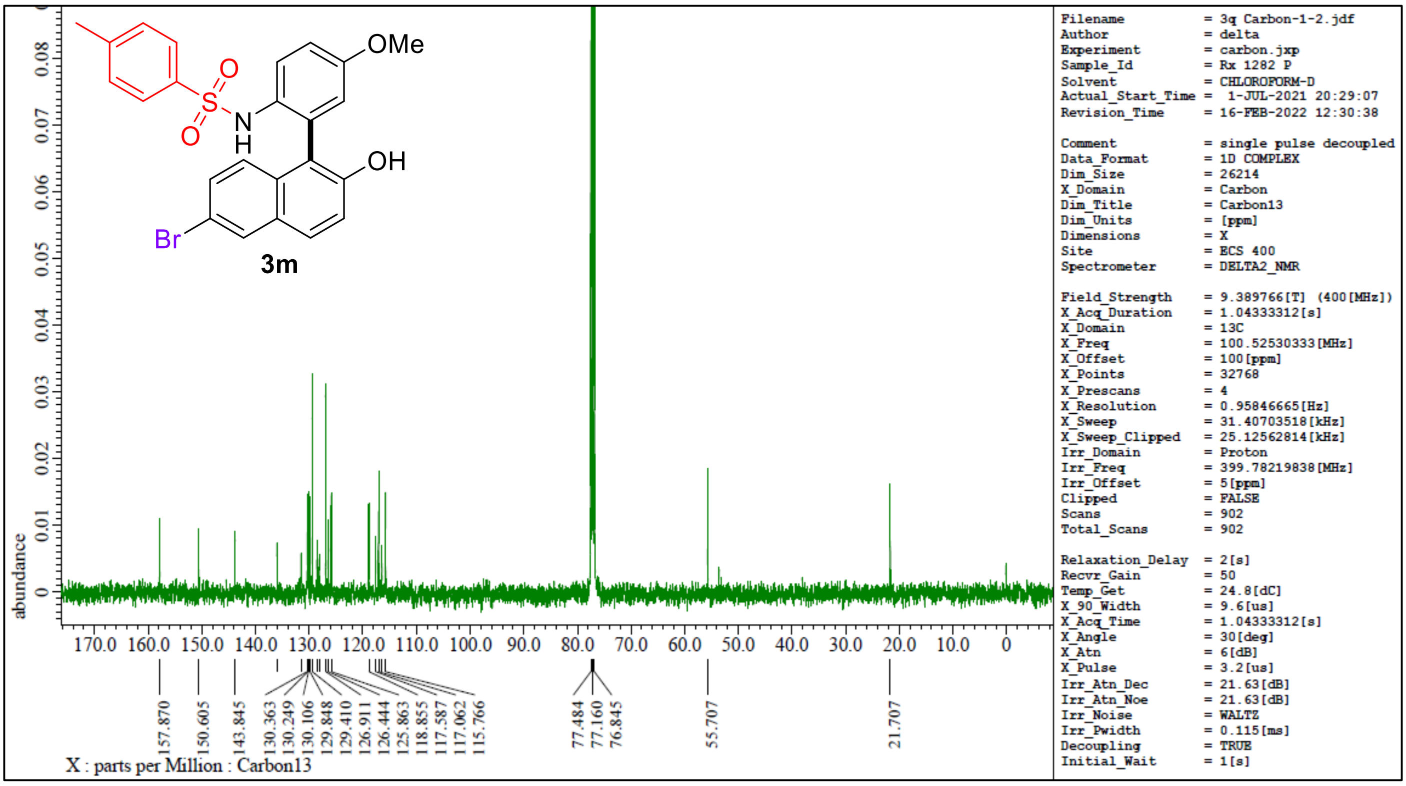

Compound **3m** (^{13}C NMR, 100 MHz, $CDCl_3$).

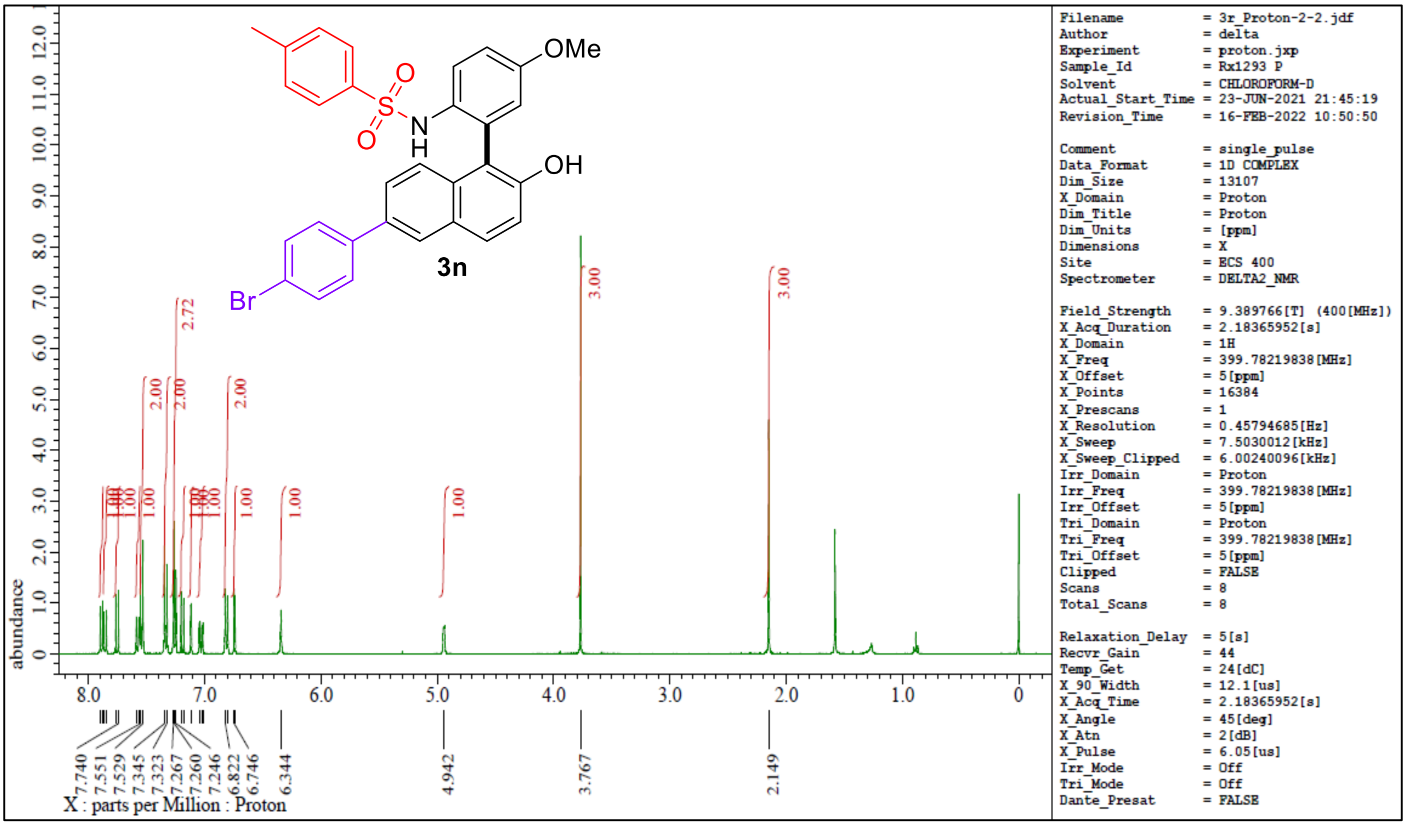

Compound **3n** (^{1}H NMR, 400 MHz, $CDCl_3$).

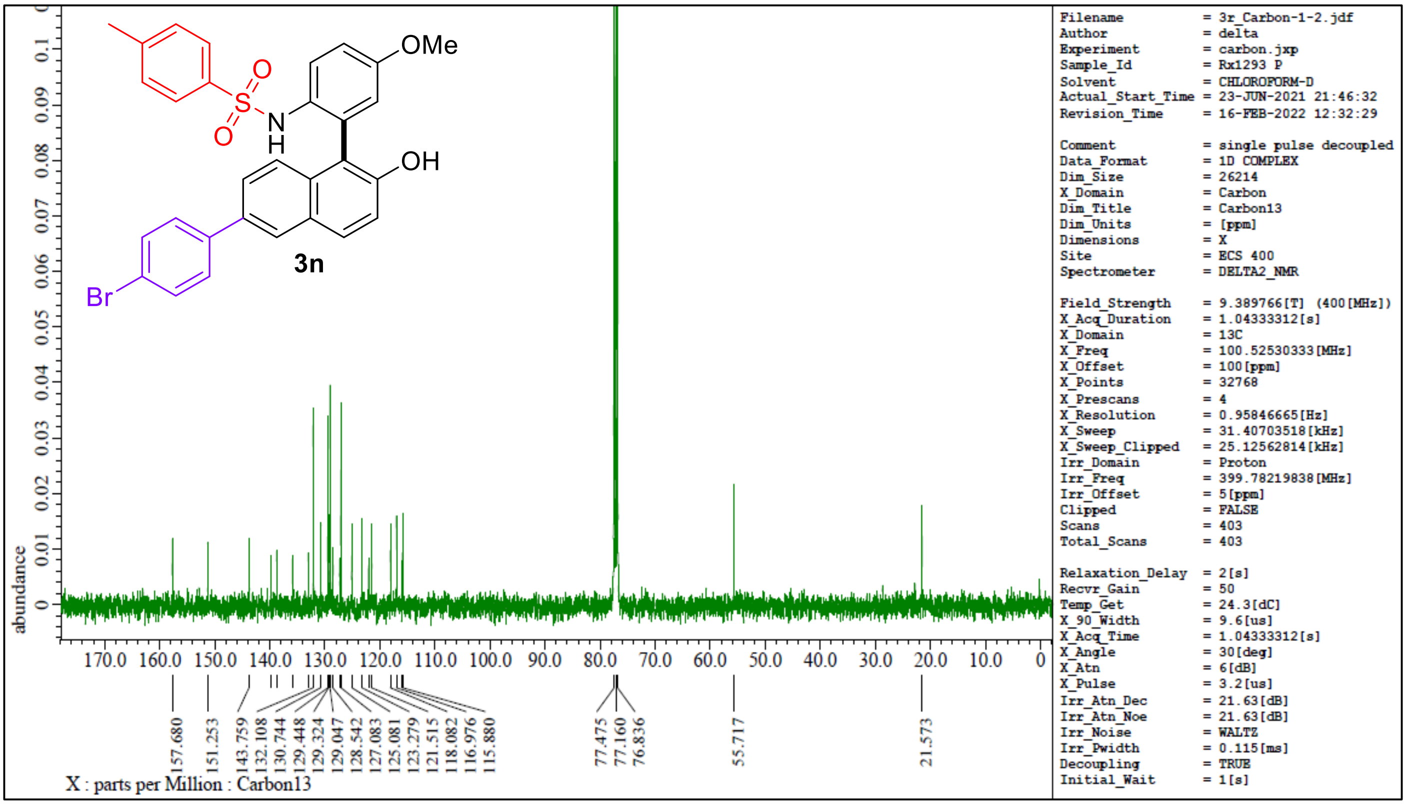

Compound **3n** (^{13}C NMR, 100 MHz, $CDCl_3$).

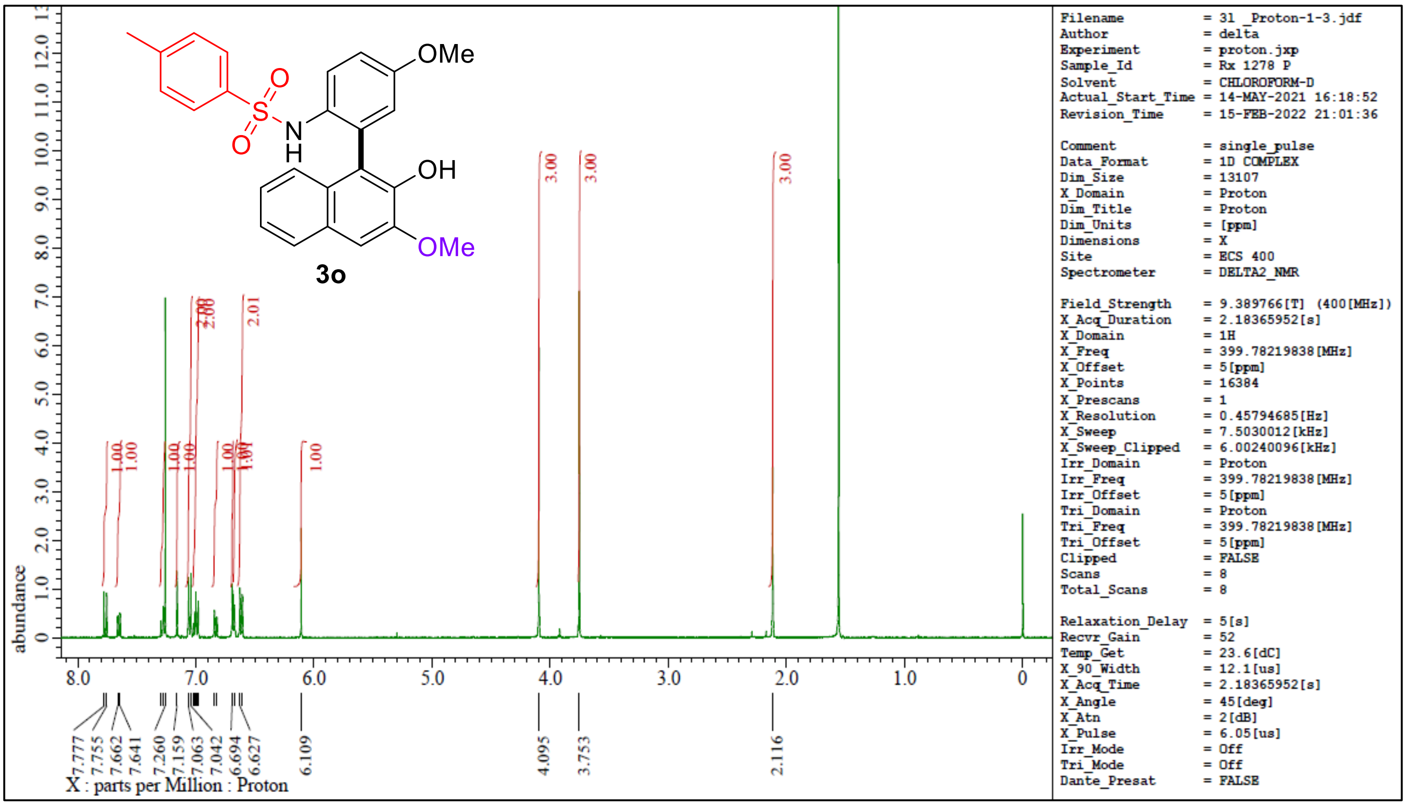

Compound **3o** (^{1}H NMR, 400 MHz, $CDCl_3$).

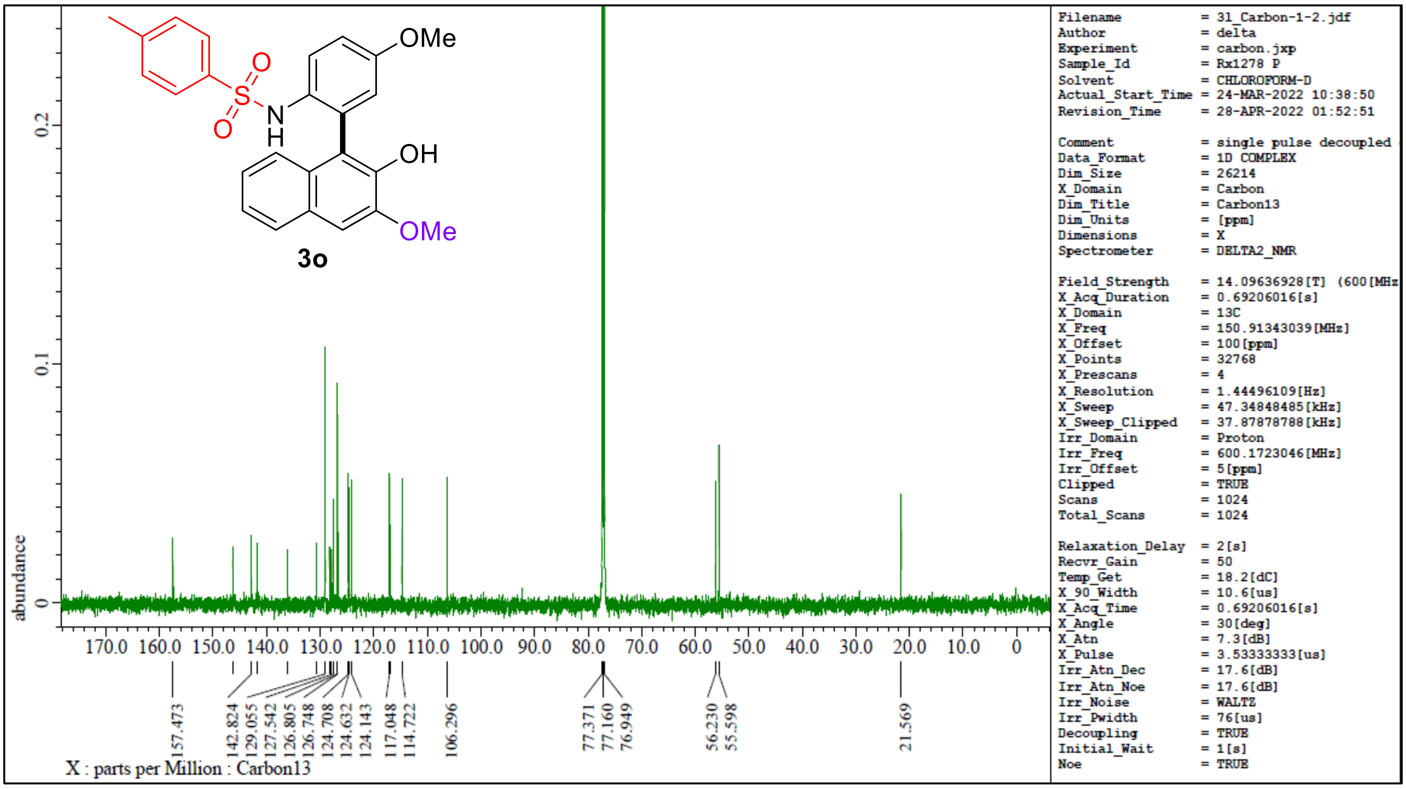

Compound **3o** (^{13}C NMR, 150 MHz, $CDCl_3$).

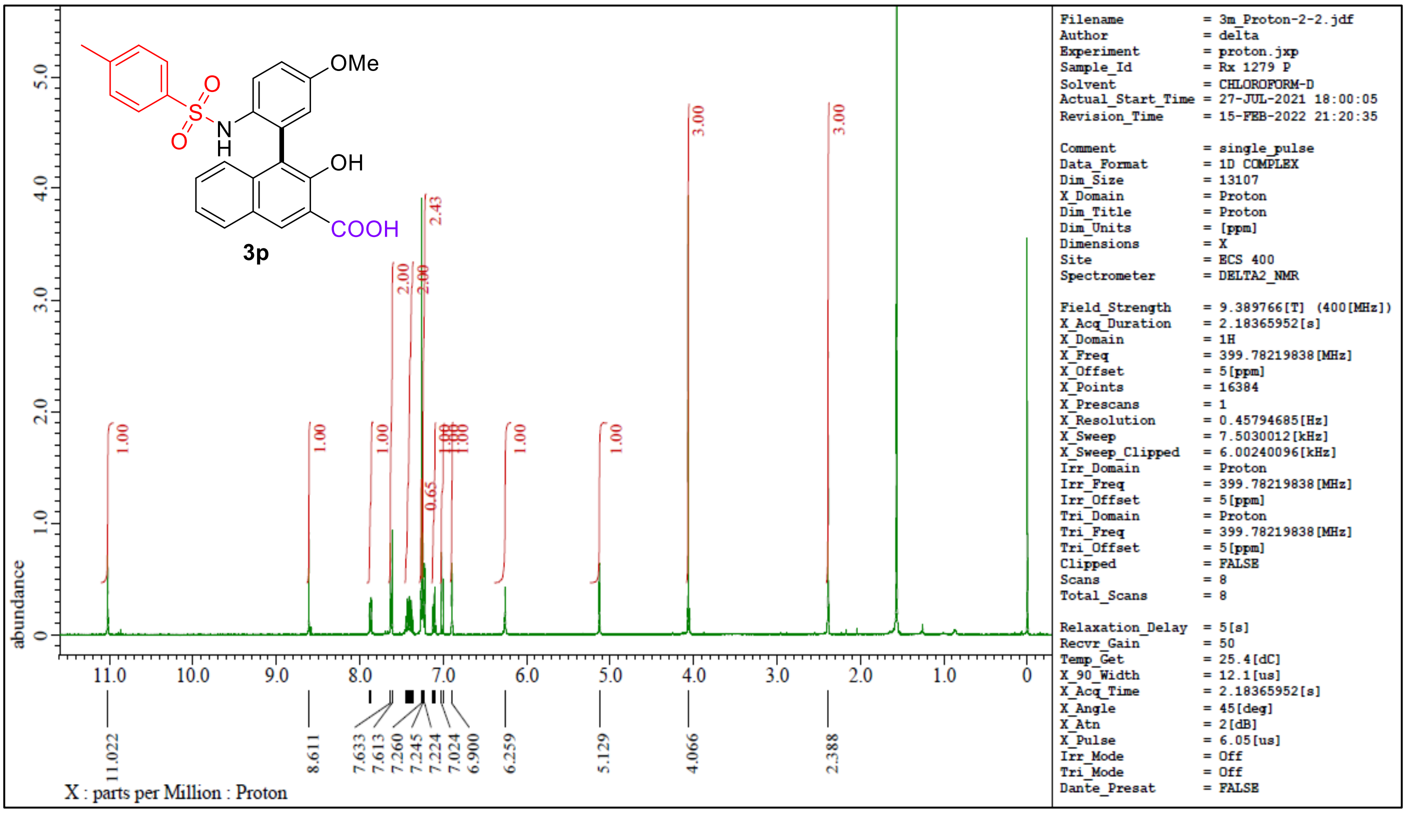

Compound **3p** (^{1}H NMR, 400 MHz, $CDCl_3$).

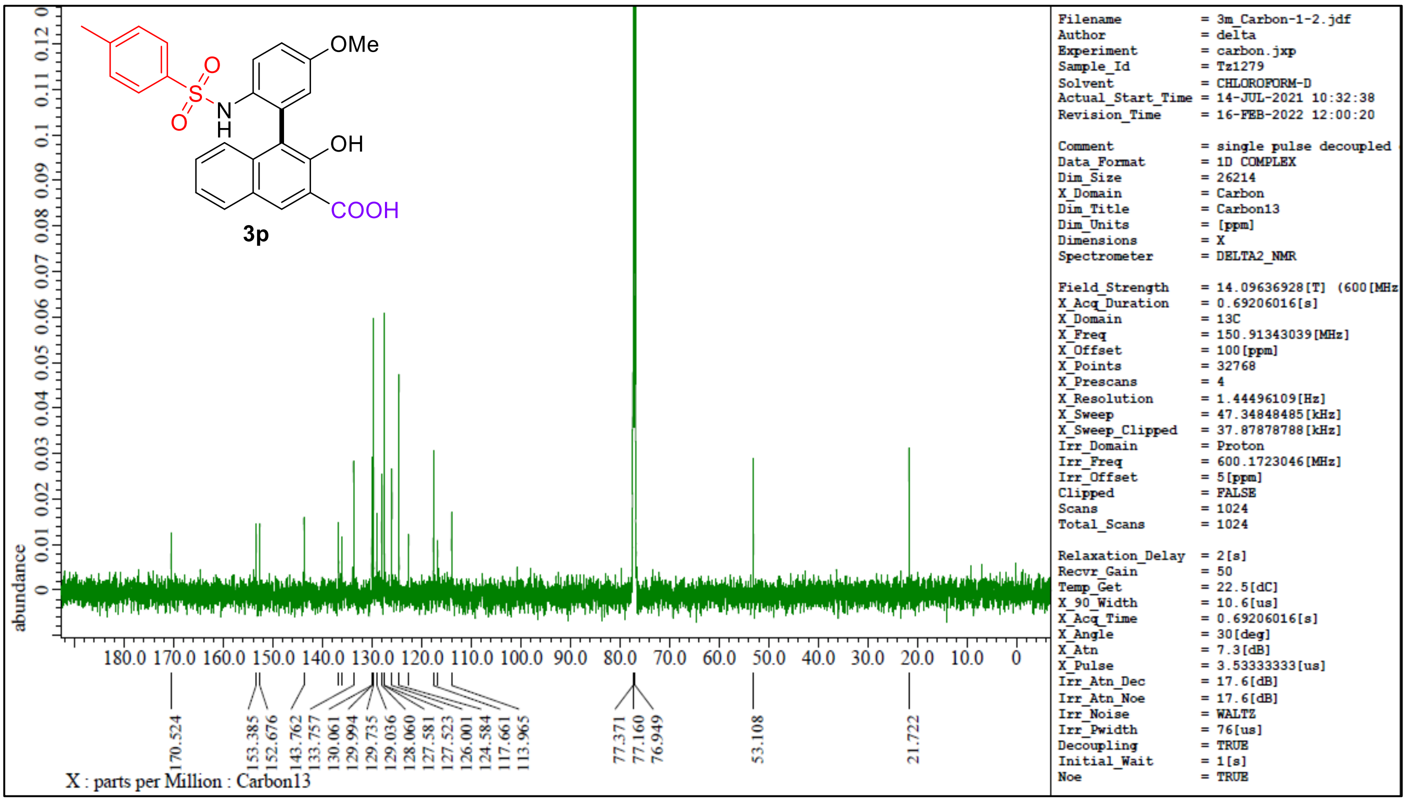

Compound **3p** (^{13}C NMR, 150 MHz, $CDCl_3$).

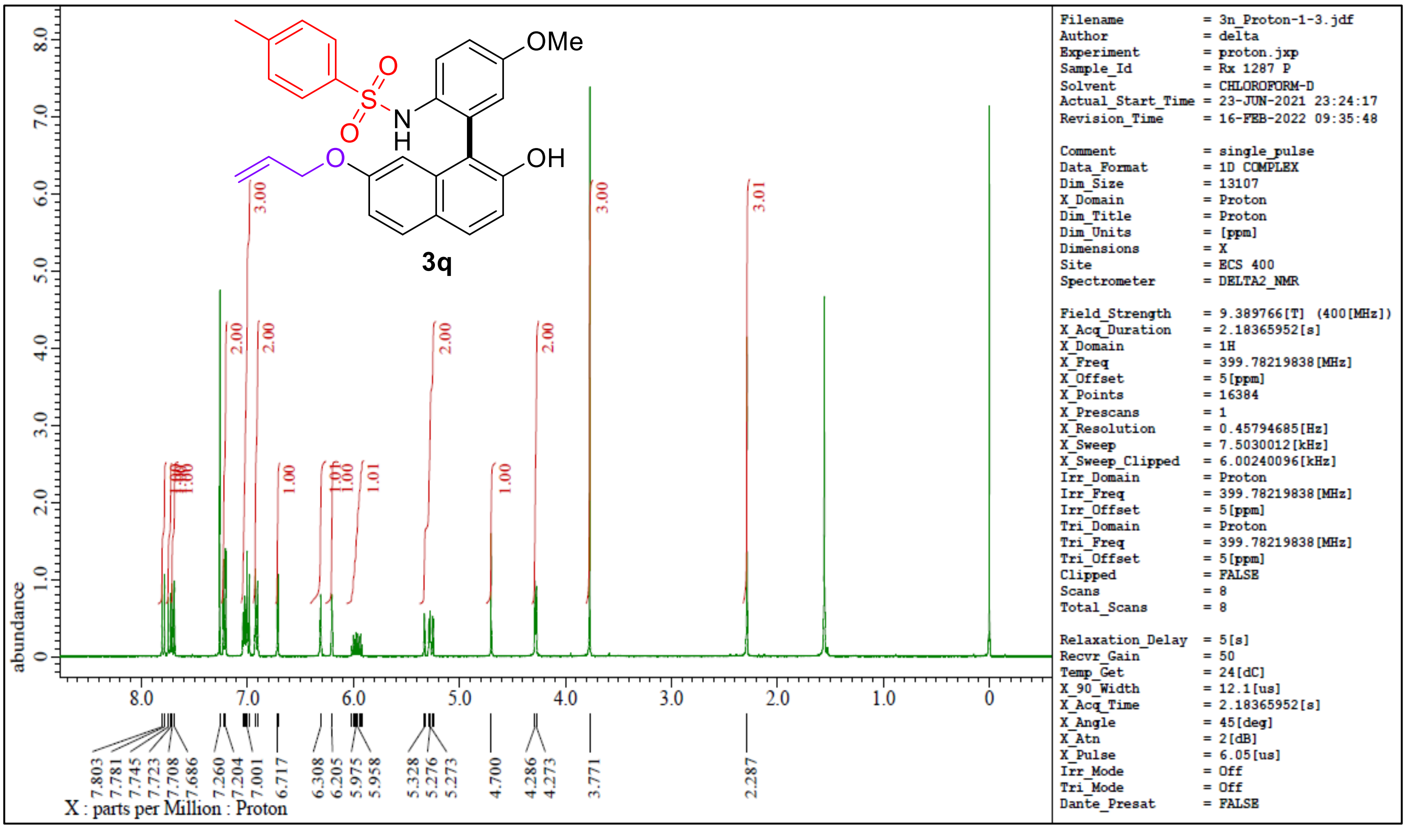

Compound **3q** (^{1}H NMR, 400 MHz, $CDCl_3$).

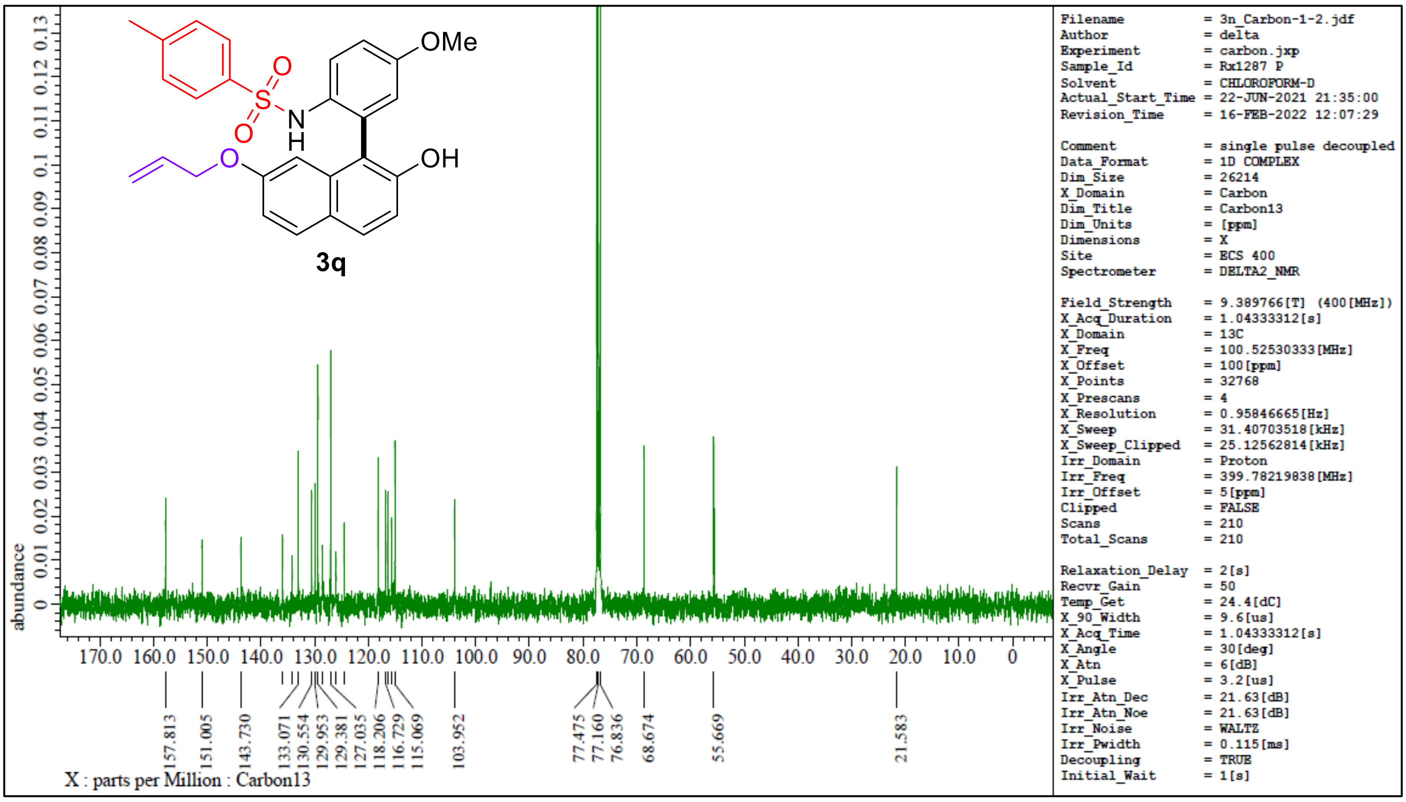

Compound **3q** (^{13}C NMR, 100 MHz, $CDCl_3$).

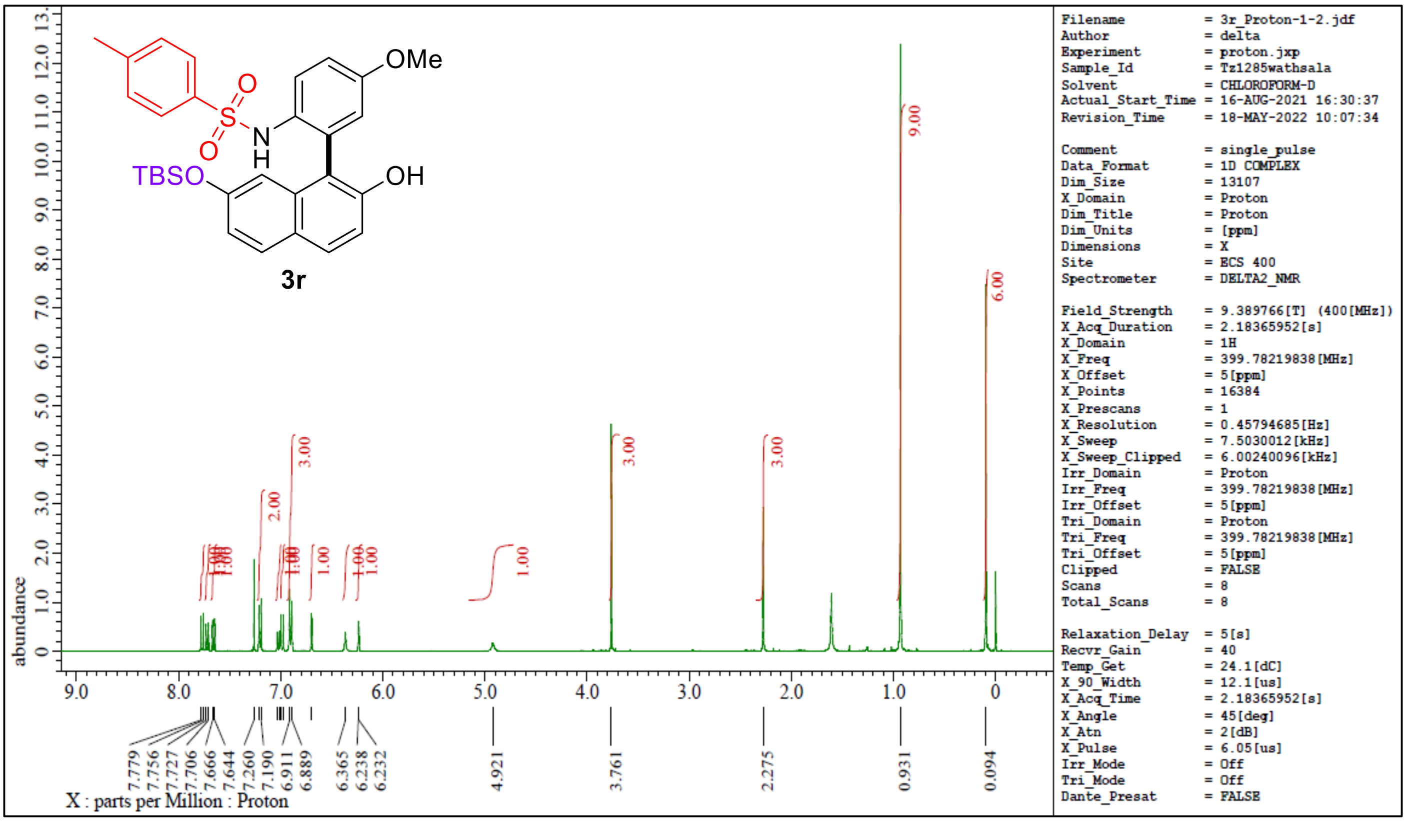

Compound **3r** (^{1}H NMR, 400 MHz, $CDCl_3$).

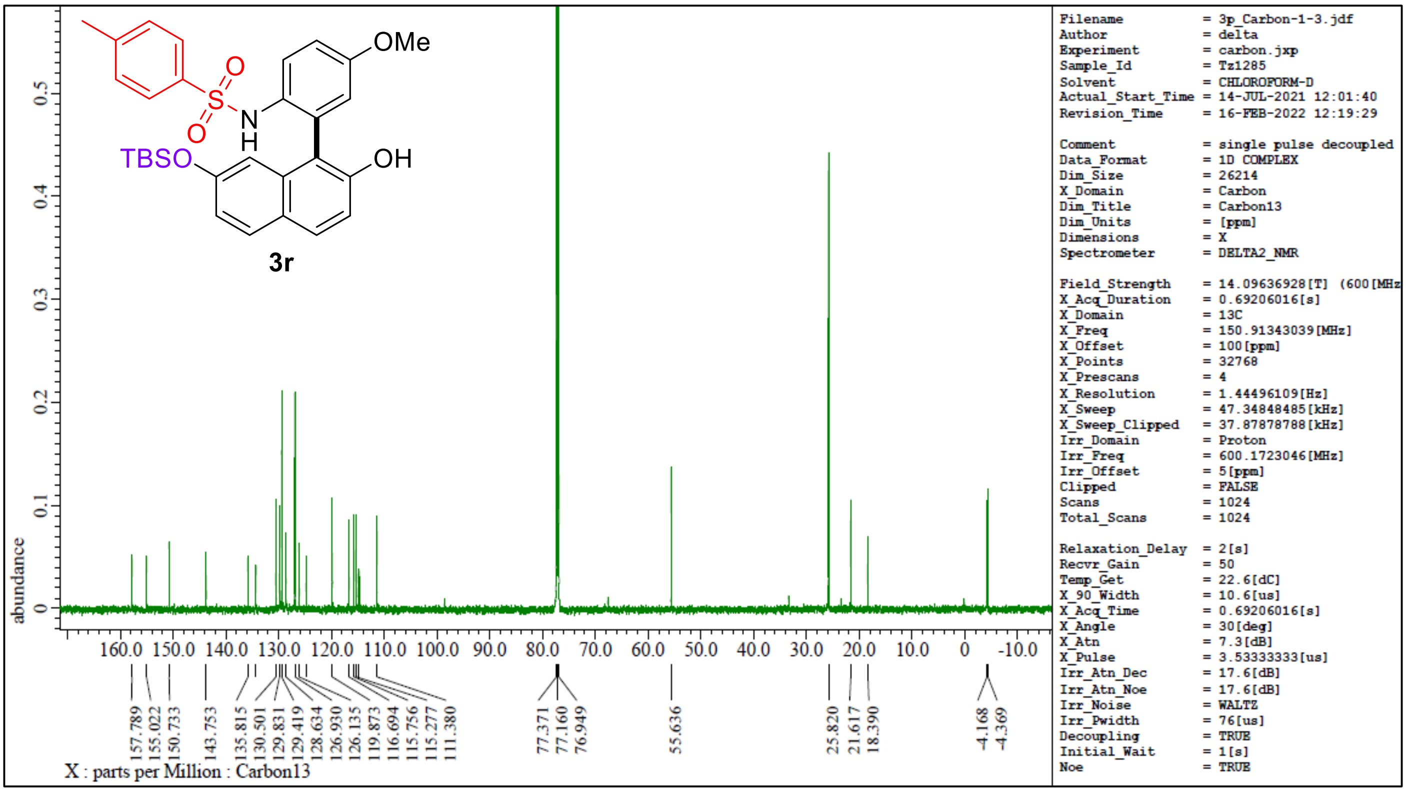

Compound **3r** (^{13}C NMR, 150 MHz, $CDCl_3$).

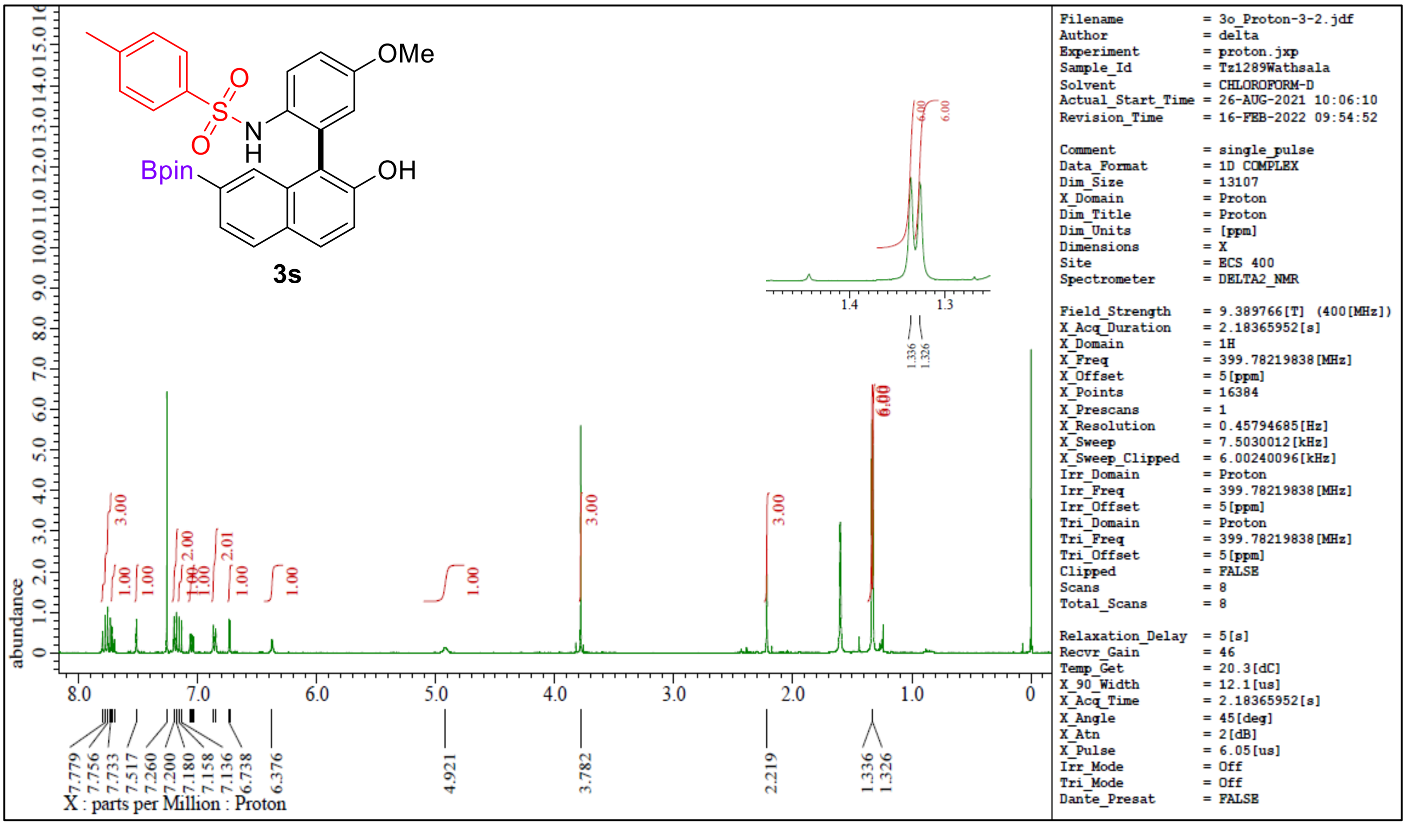

Compound **3s** (^{1}H NMR, 400 MHz, $CDCl_3$).

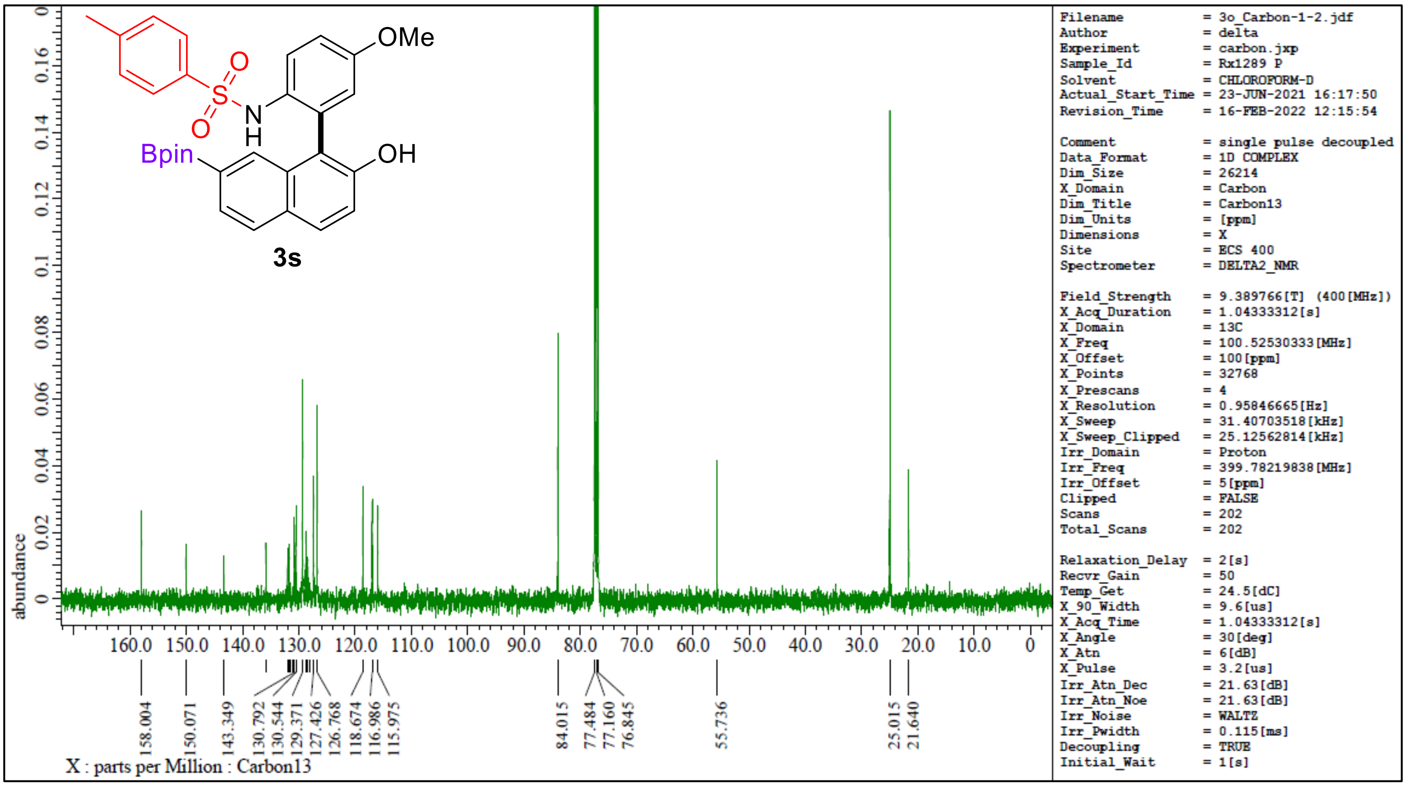

Compound **3s** (^{13}C NMR, 100 MHz, $CDCl_3$).

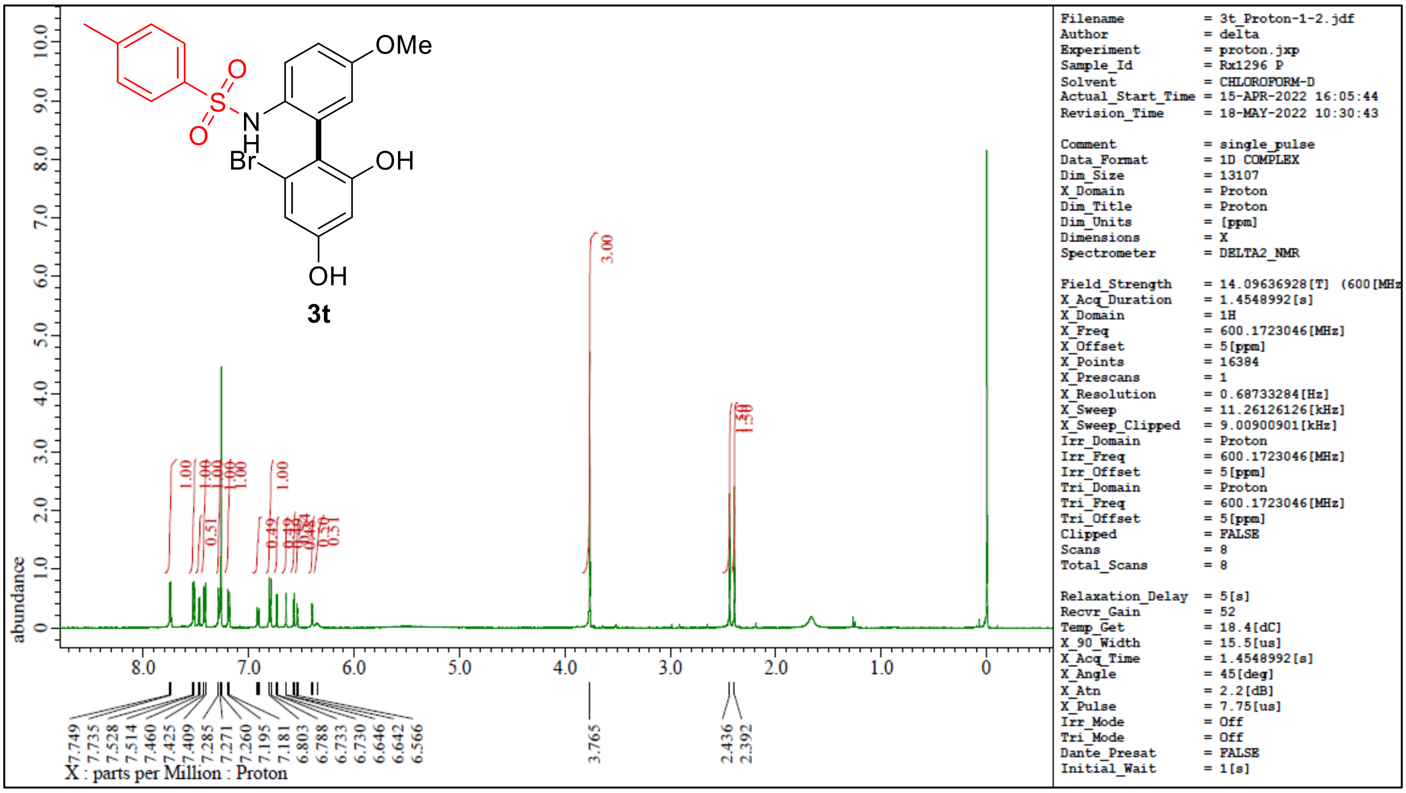

Compound **3t** (^{1}H NMR, 600 MHz, $CDCl_3$).

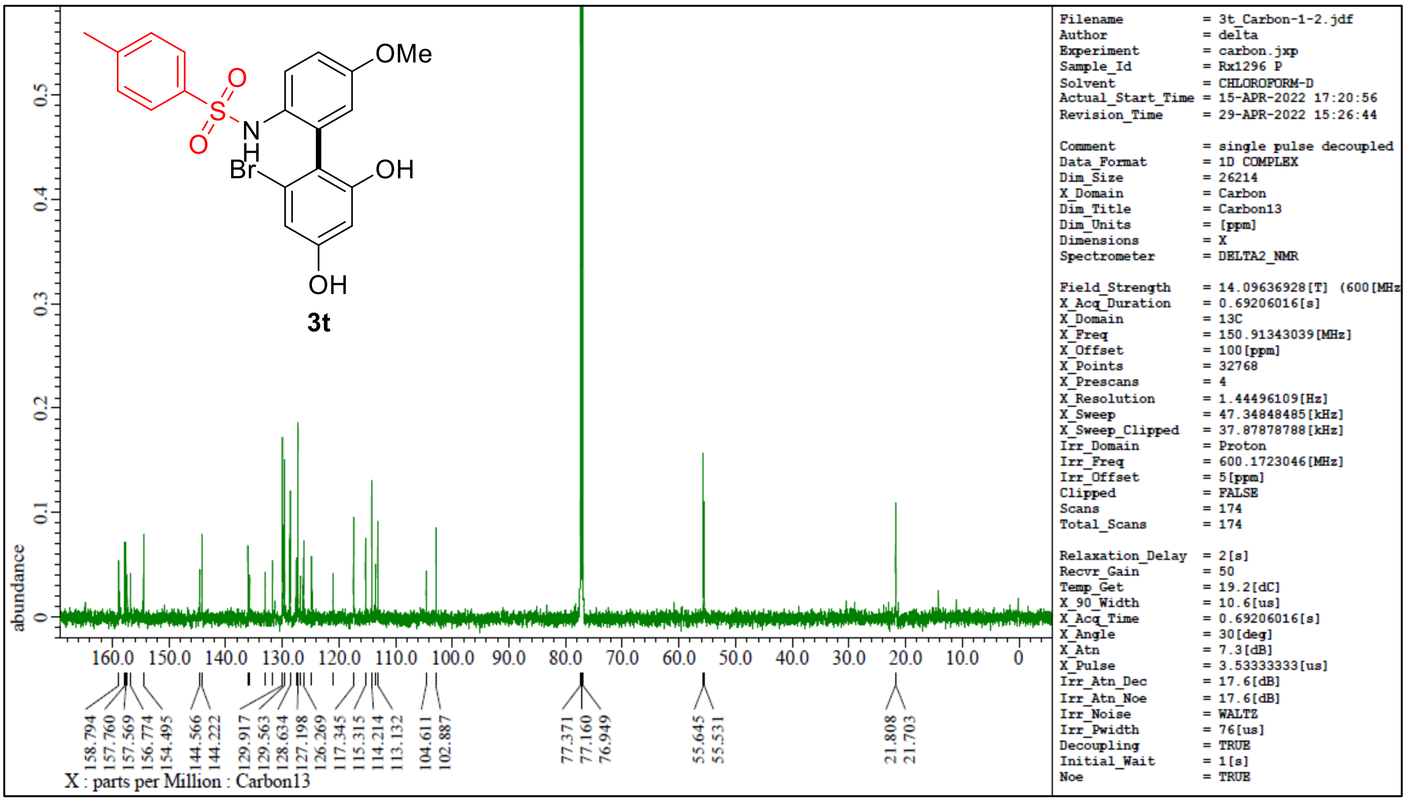

Compound **3t** (^{13}C NMR, 150 MHz, $CDCl_3$).

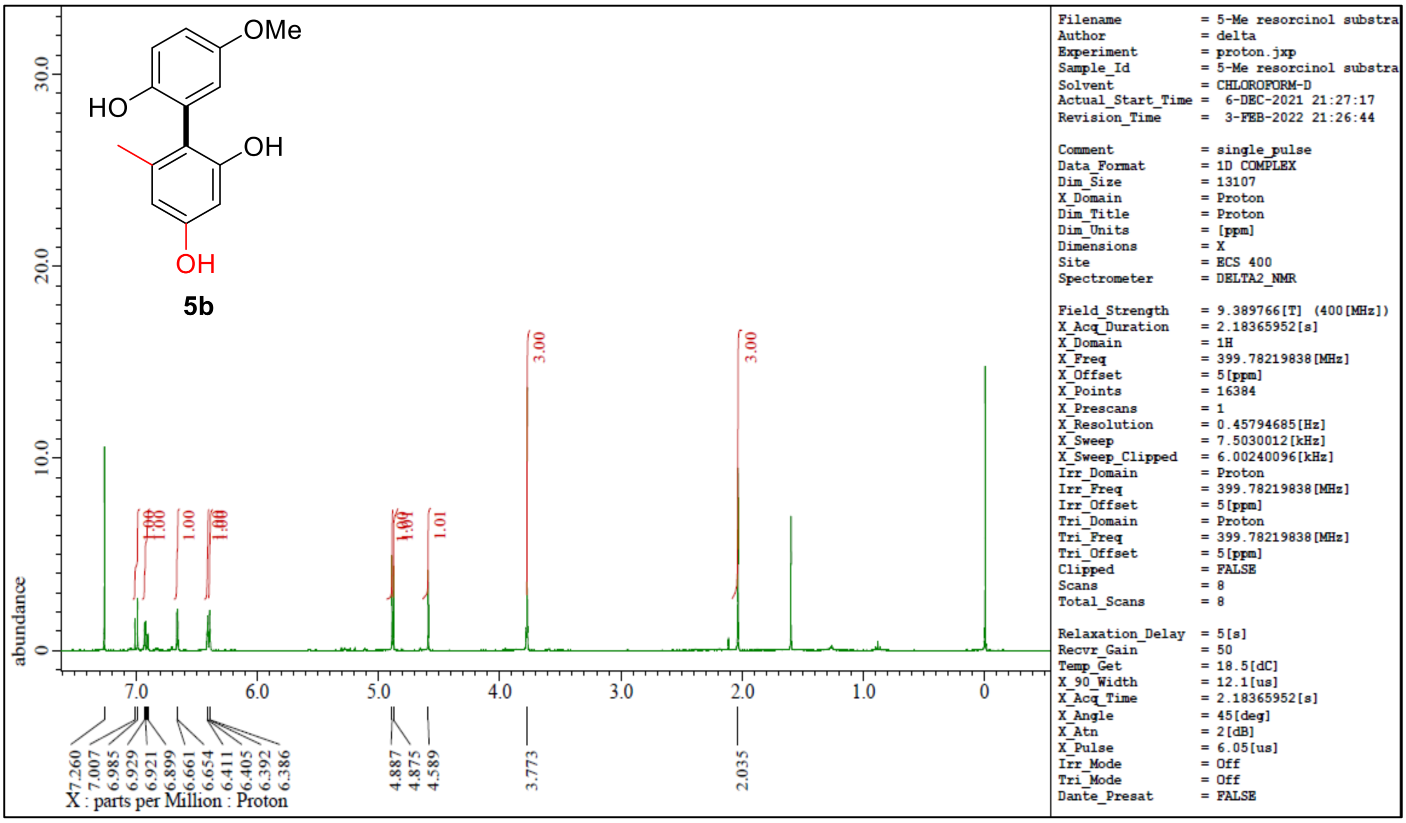

Compound **5b** (^{1}H NMR, 400 MHz, $CDCl_3$).

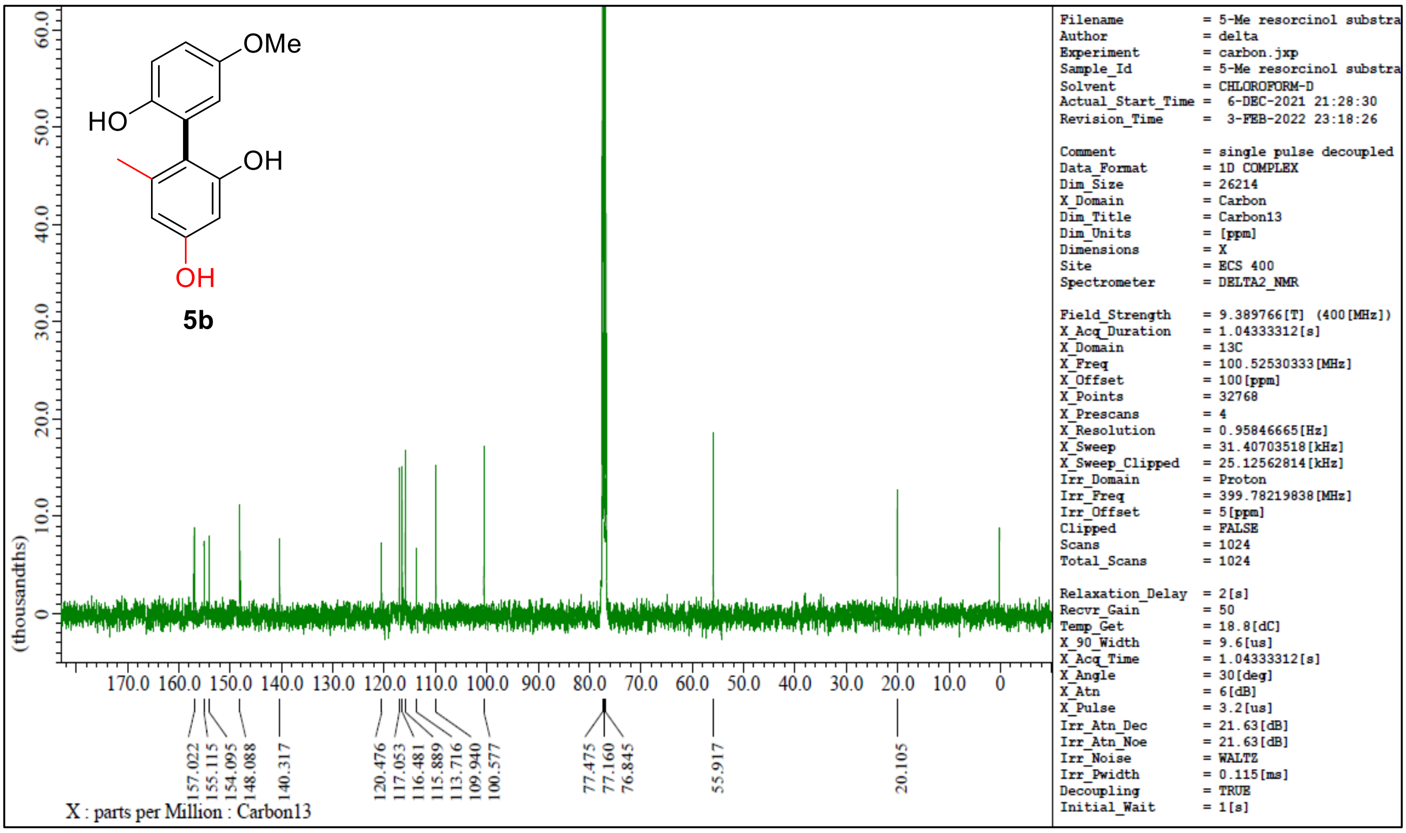

Compound **5b** (^{13}C NMR, 100 MHz, $CDCl_3$).

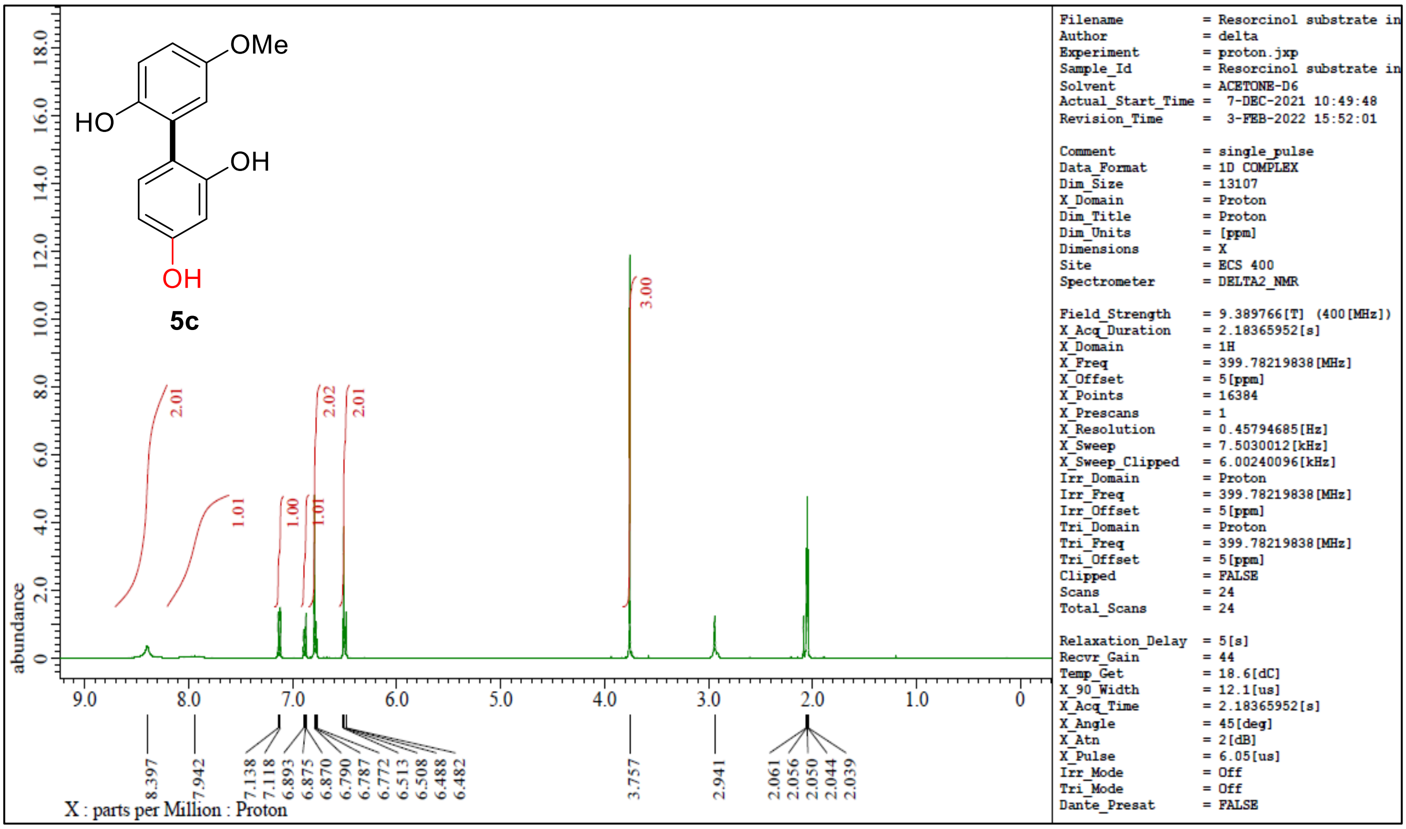

Compound **5c** (^{1}H NMR, 400 MHz, $(CD_3)_2CO$).

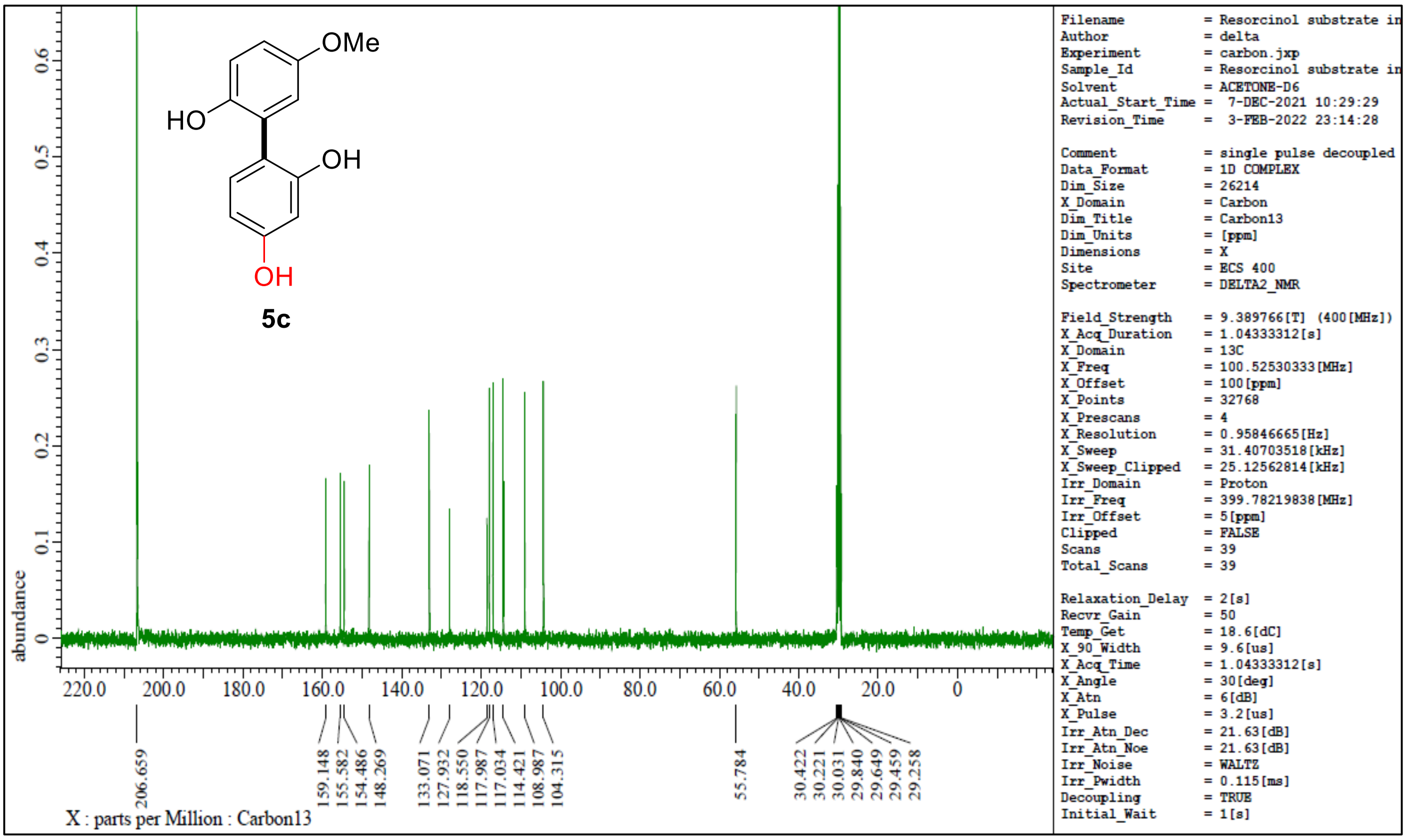

Compound **5c** (^{13}C NMR, 100 MHz, $(CD_3)_2CO$).

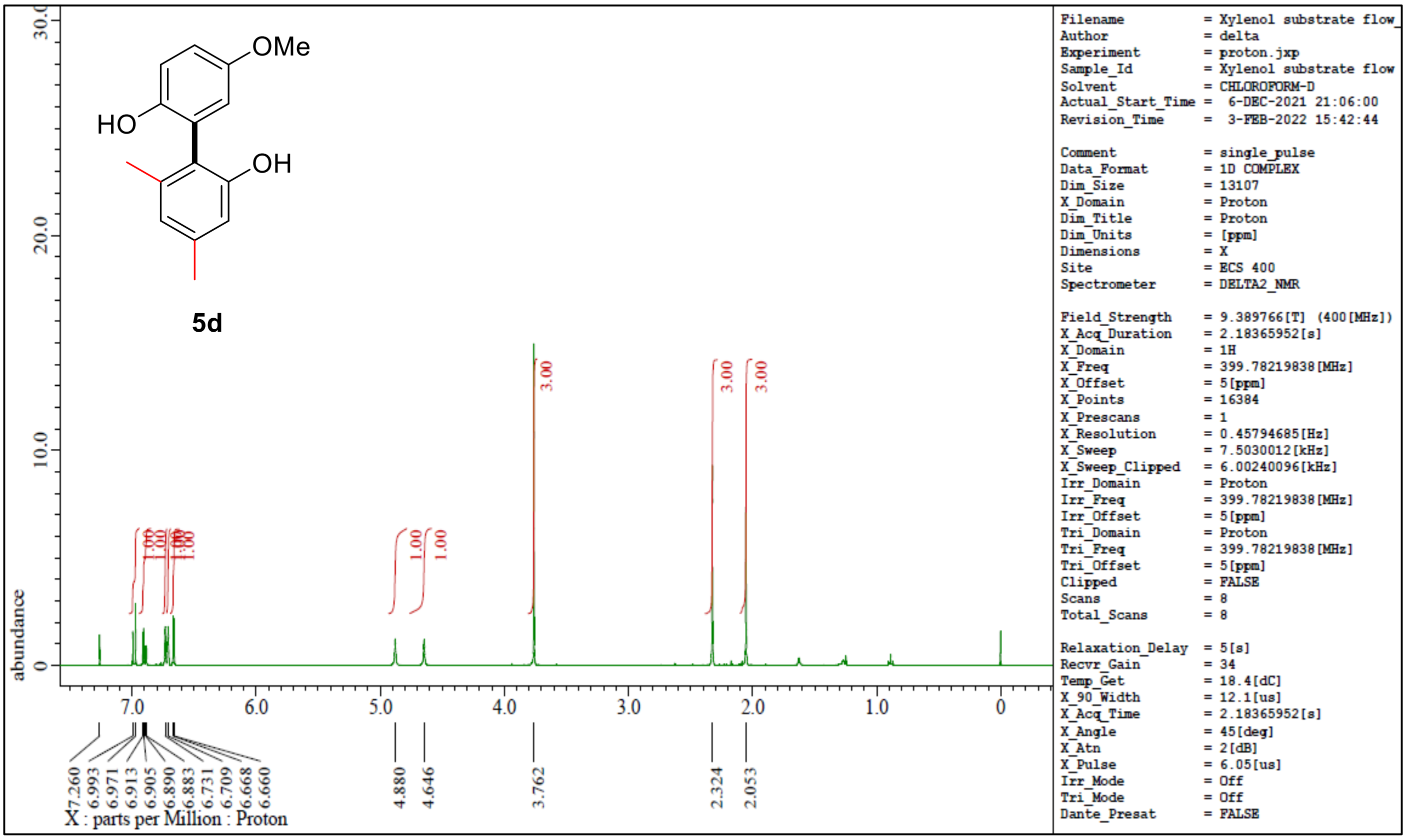

Compound **5d** (^{1}H NMR, 400 MHz, $CDCl_3$).

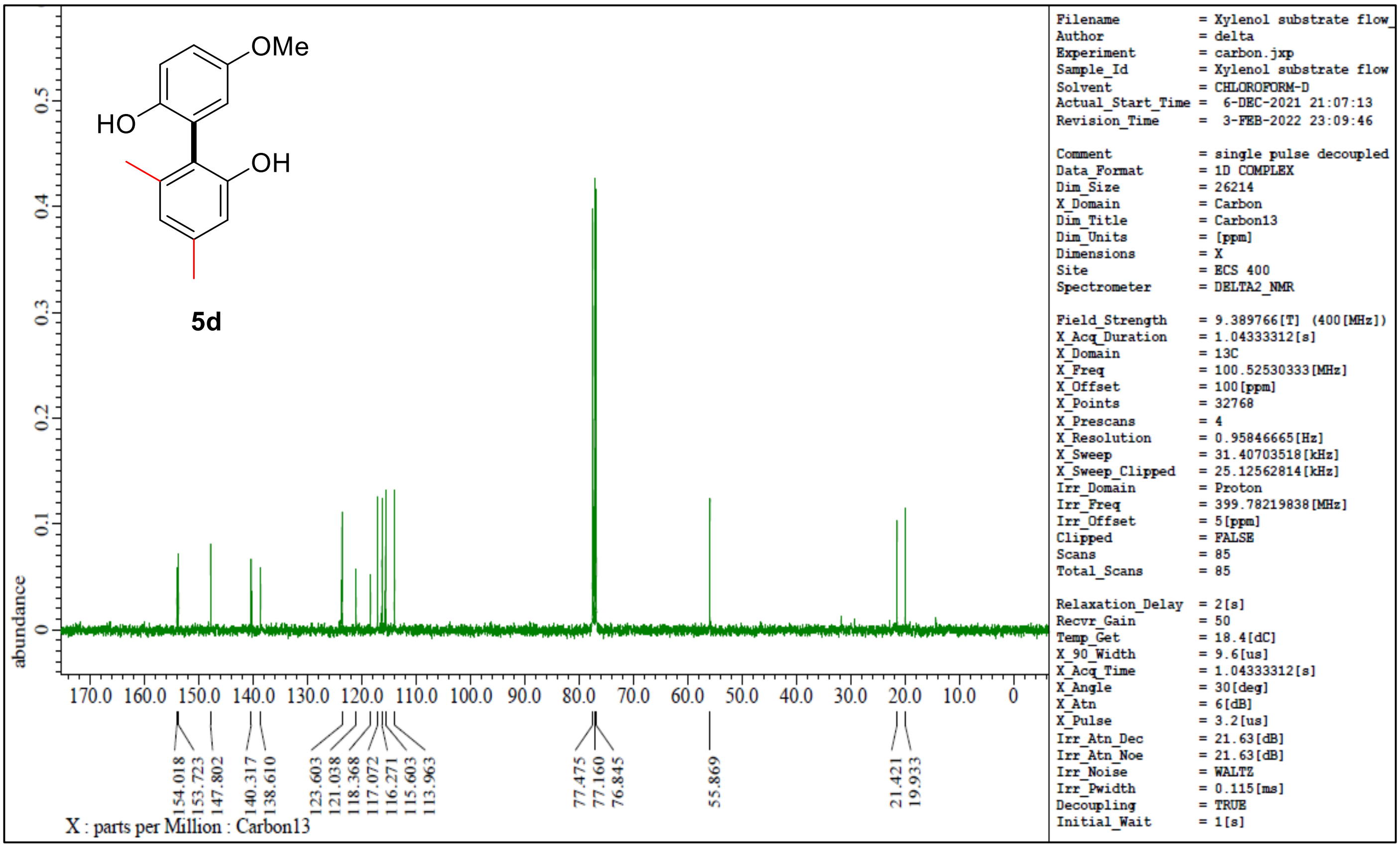

Compound **5d** (^{13}C NMR, 100 MHz, $CDCl_3$).

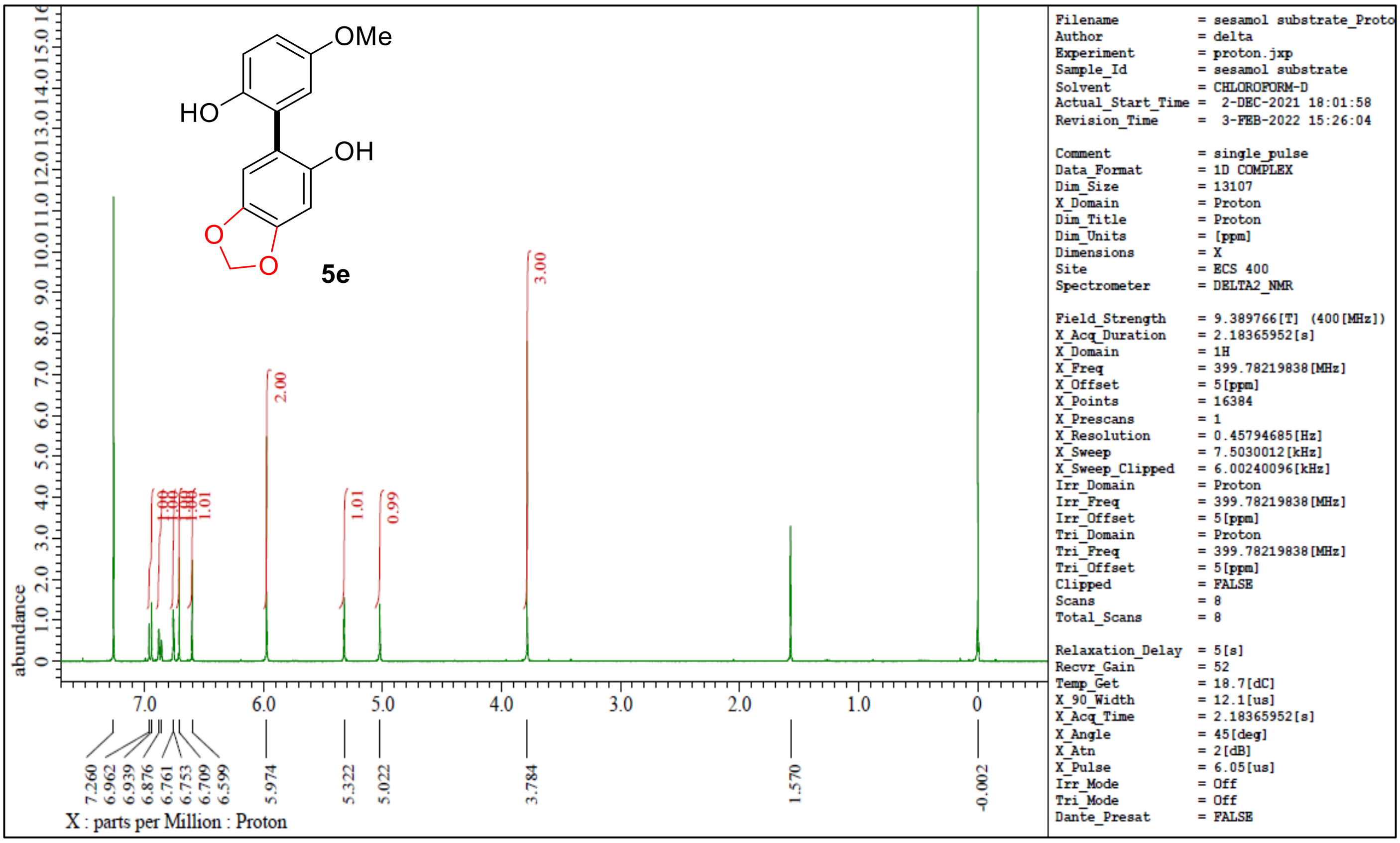

Compound **5e** (^{1}H NMR, 400 MHz, $CDCl_3$).

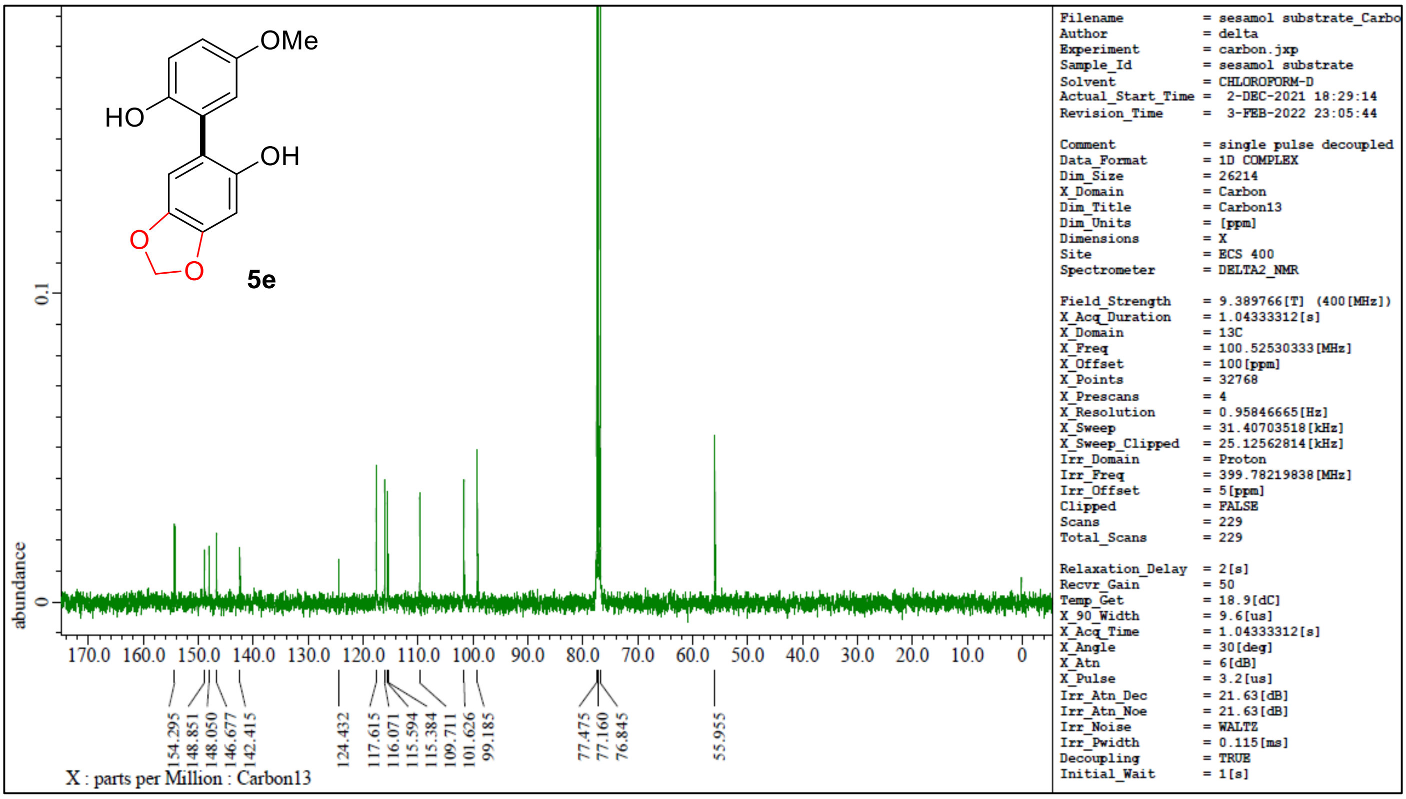

Compound **5e** (^{13}C NMR, 100 MHz, $CDCl_3$).

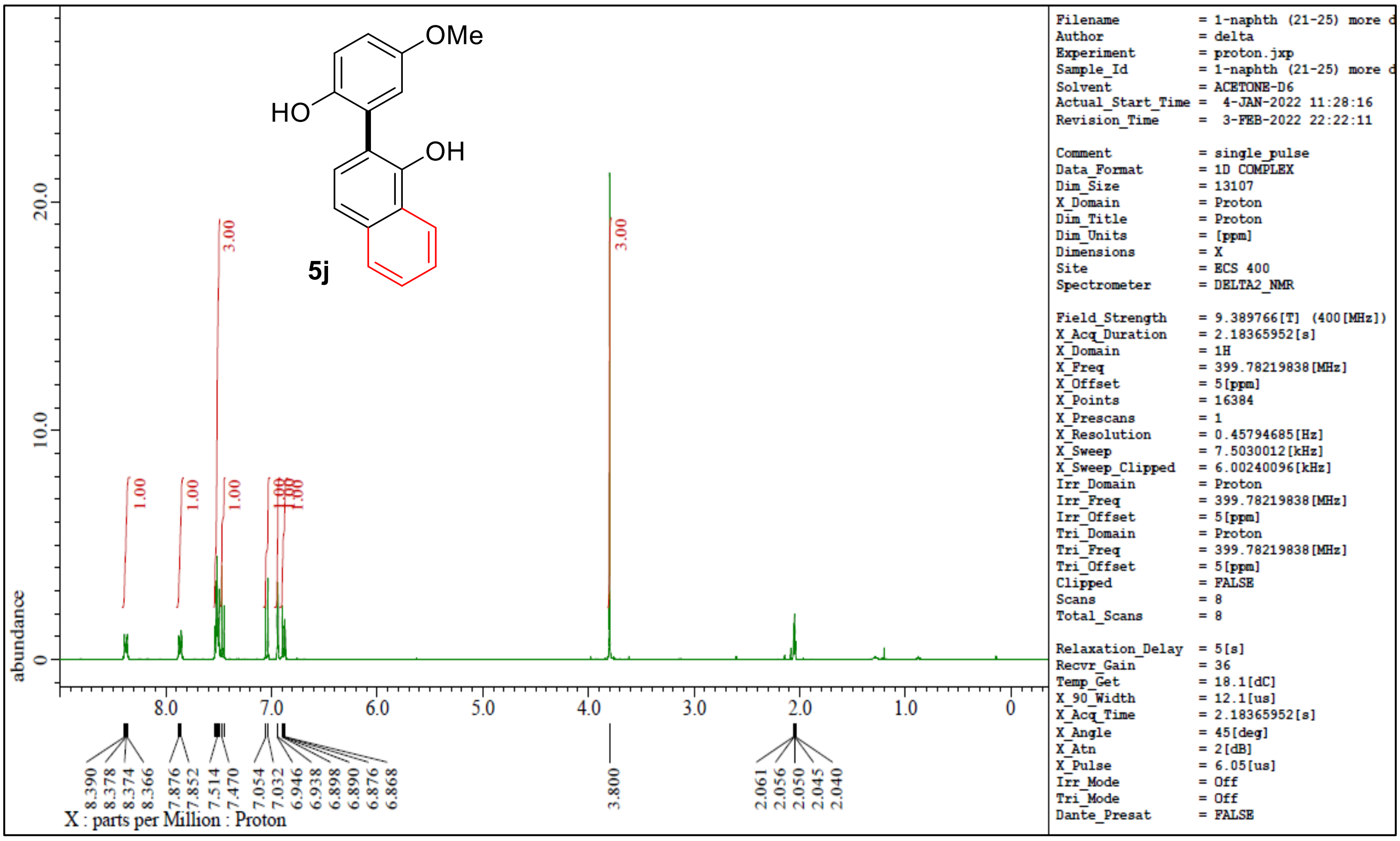

Compound **5j** (^{1}H NMR, 400 MHz, $(CD_3)_2CO$).

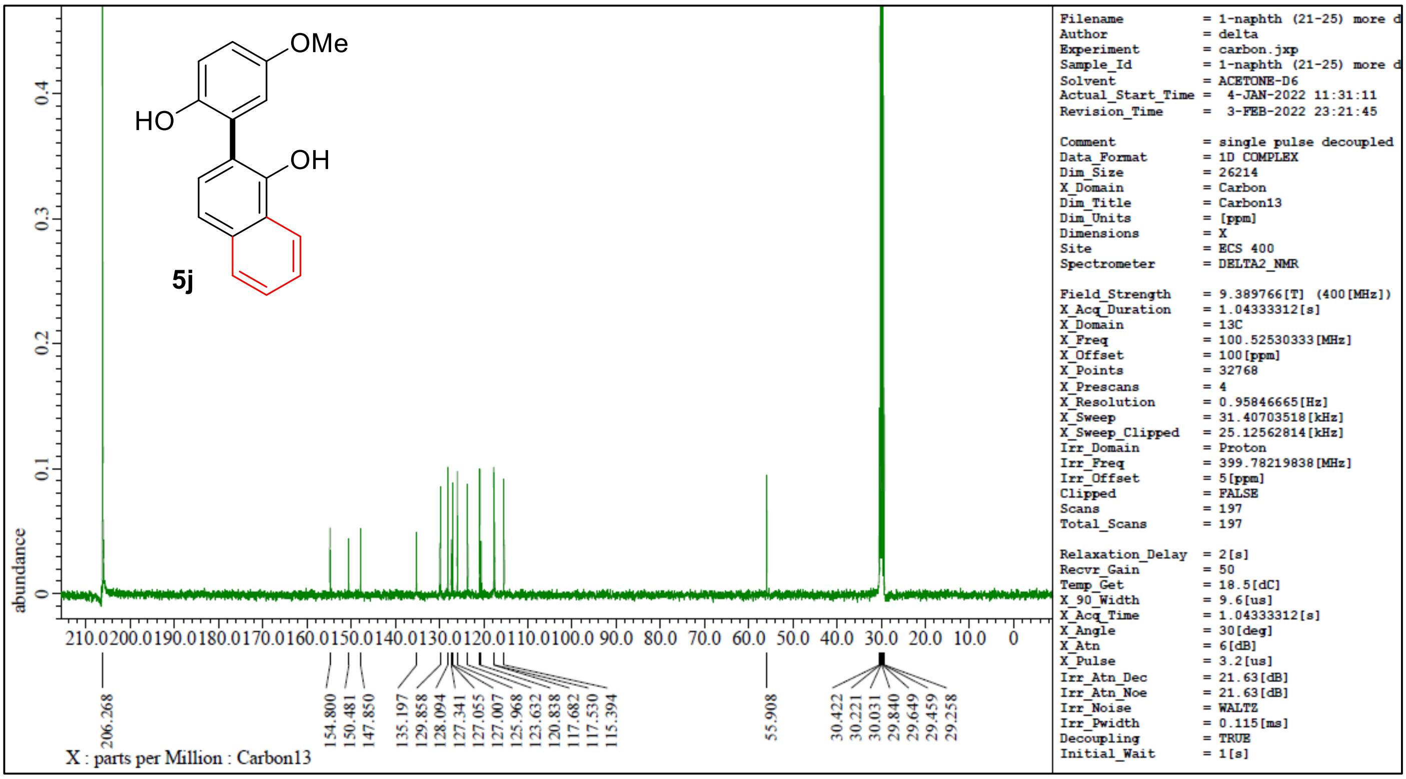

Compound **5j** (^{13}C NMR, 100 MHz, $(CD_3)_2CO$).

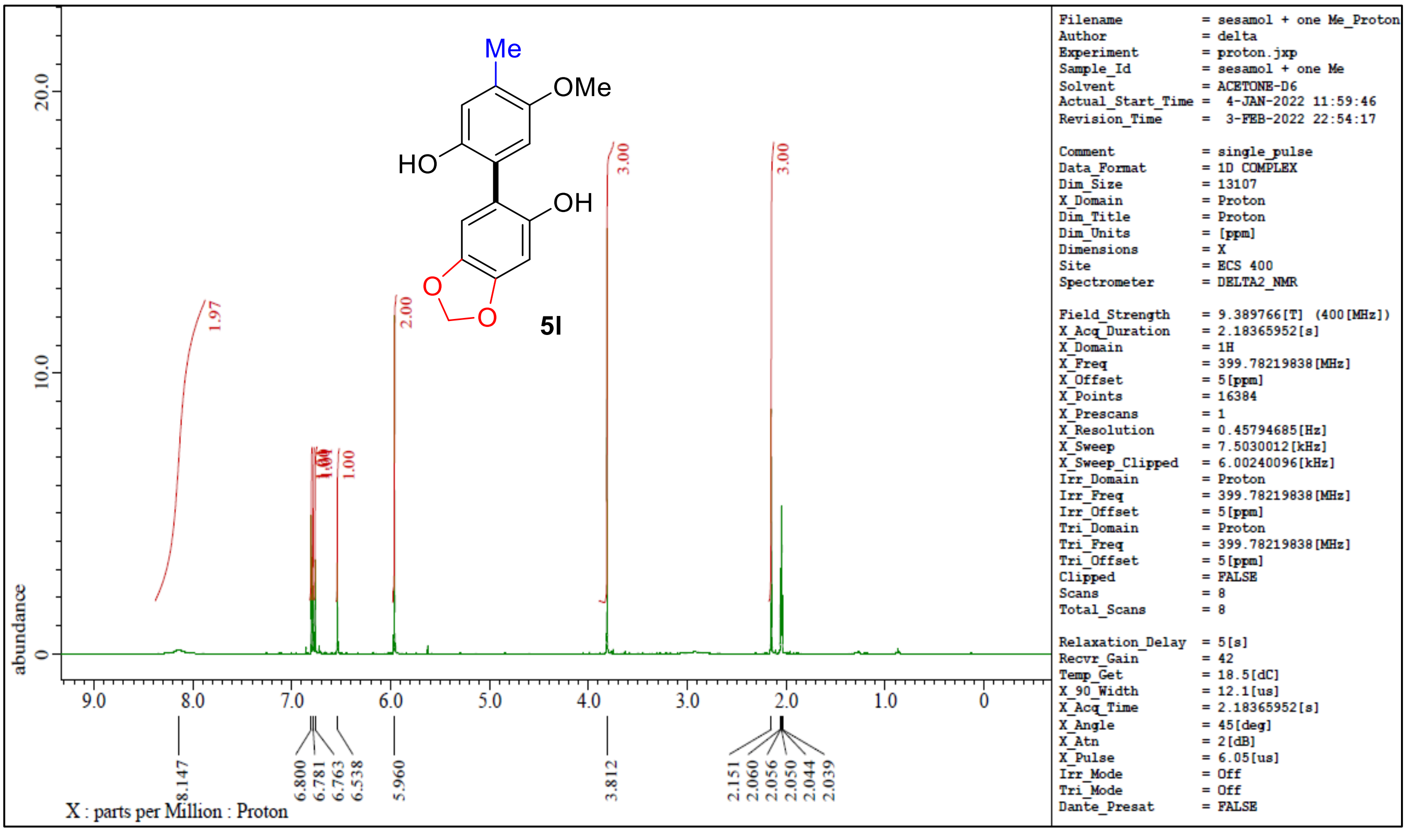

Compound **5l** (^{1}H NMR, 400 MHz, $(CD_3)_2CO$).

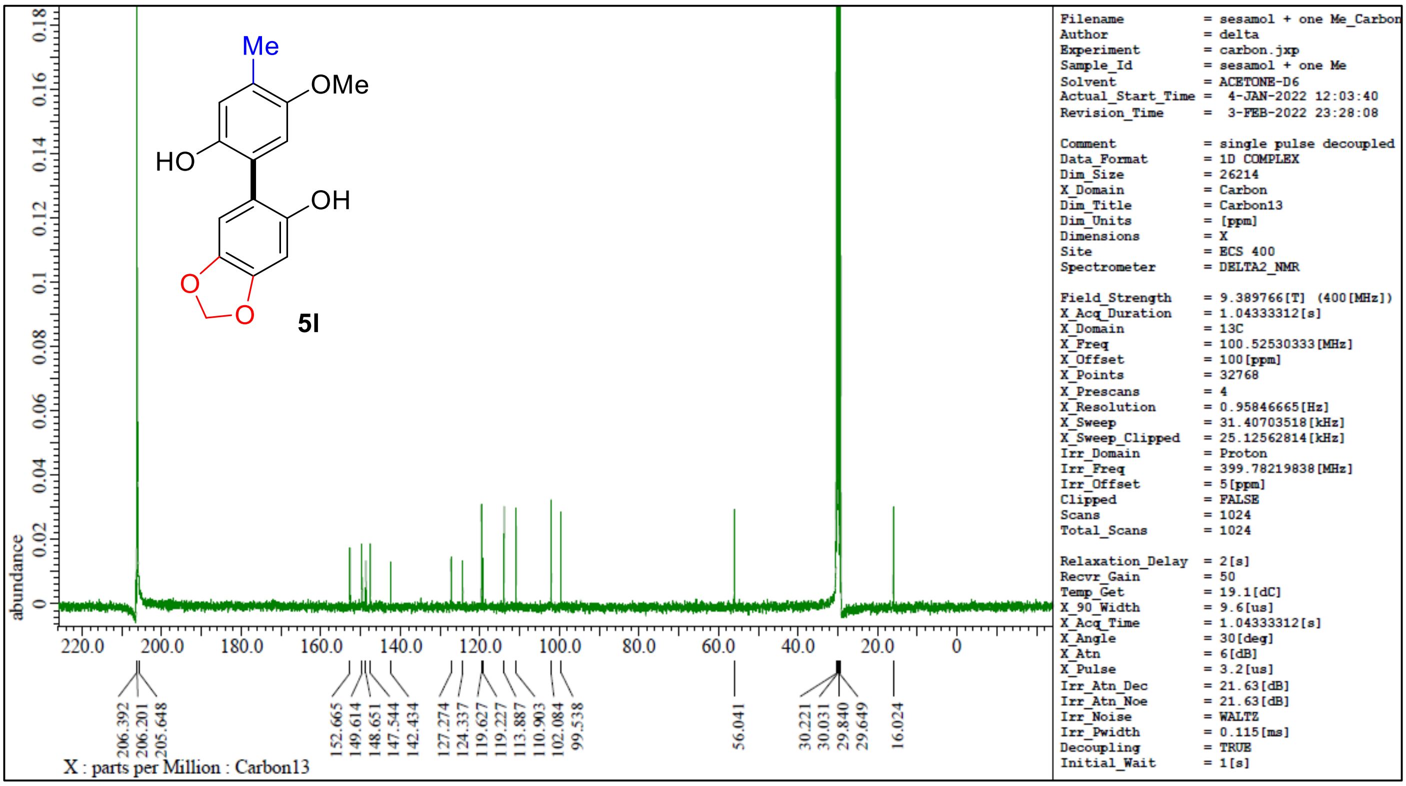

Compound **5l** (^{13}C NMR, 100 MHz, $(CD_3)_2CO$).

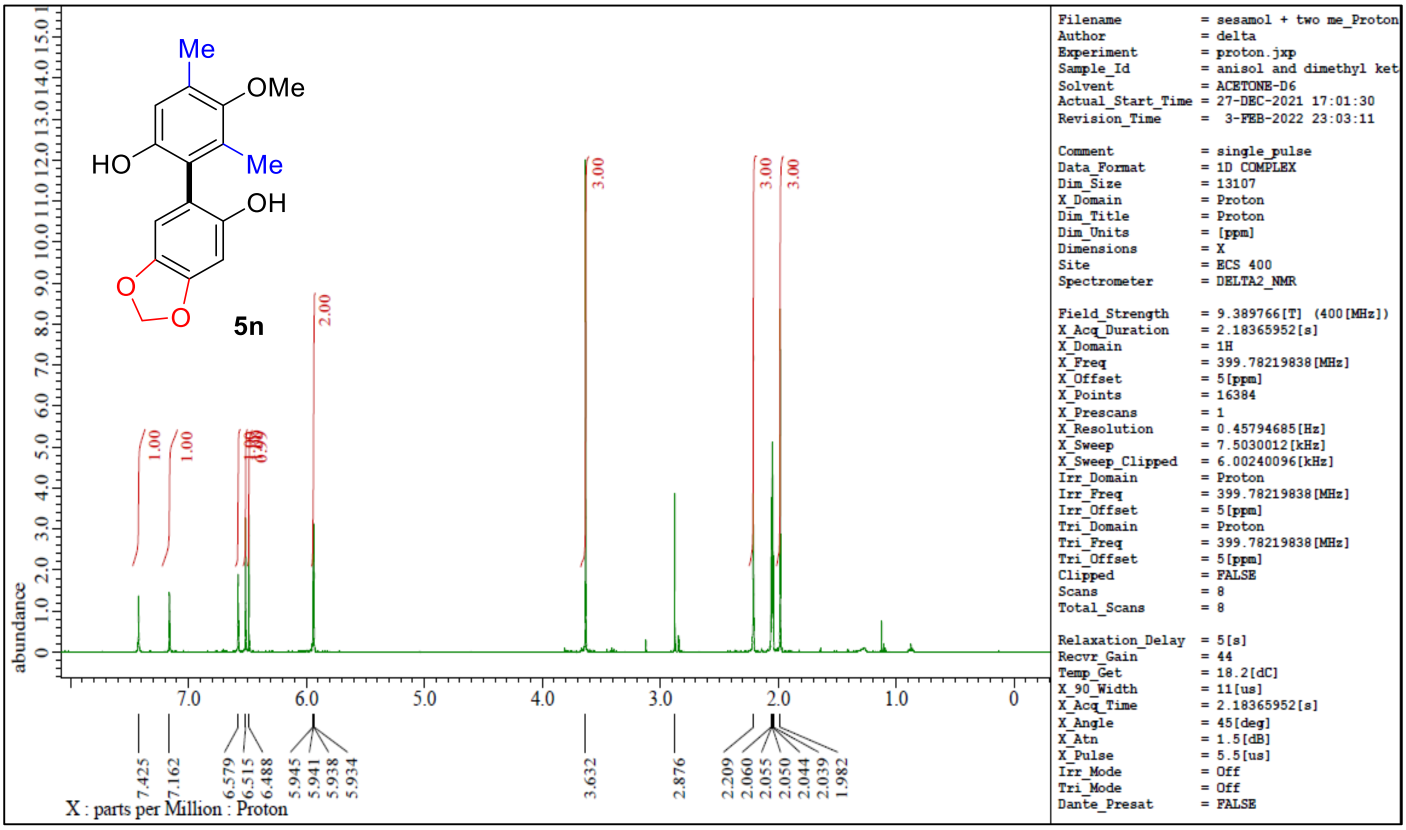

Compound **5n** (^{1}H NMR, 400 MHz, $(CD_3)_2CO$).

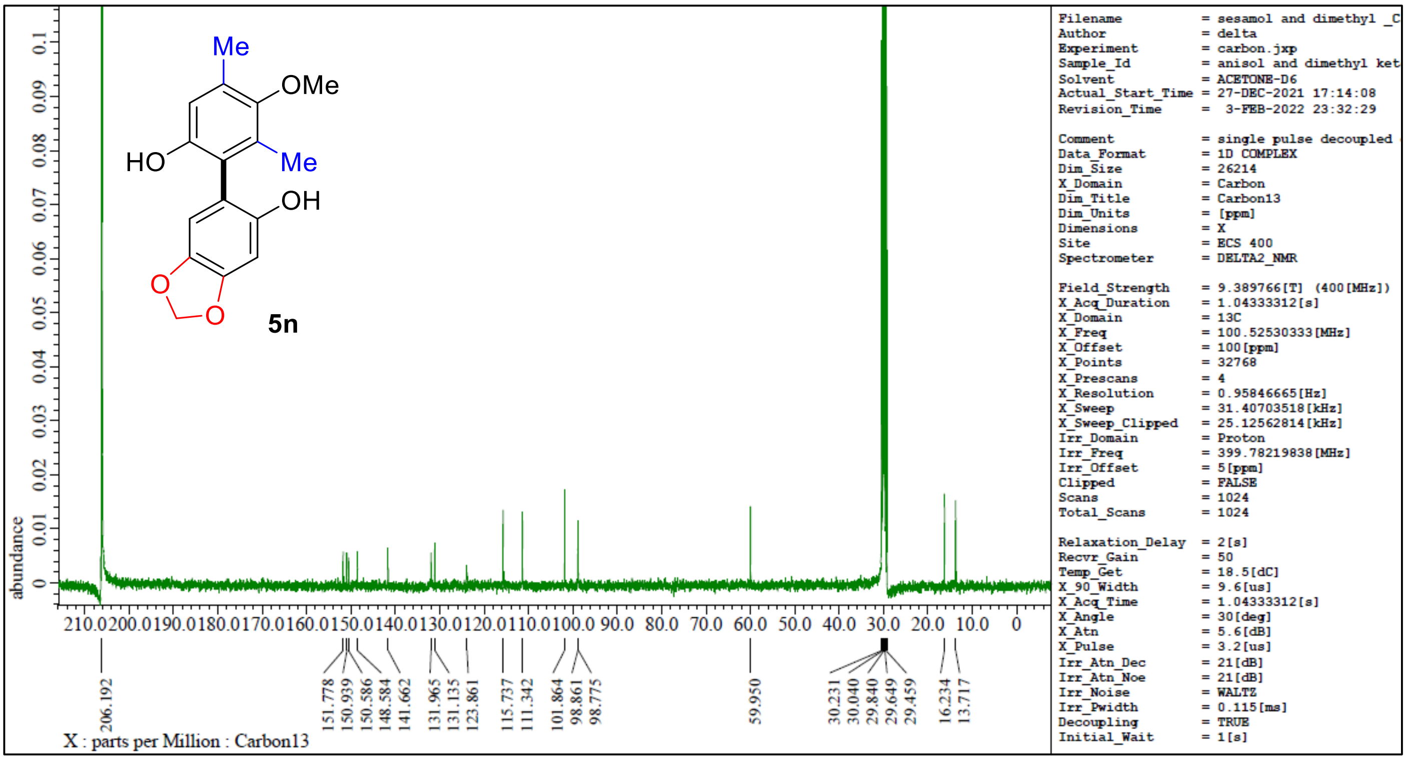

Compound **5n** (^{13}C NMR, 100 MHz, $(CD_3)_2CO$).

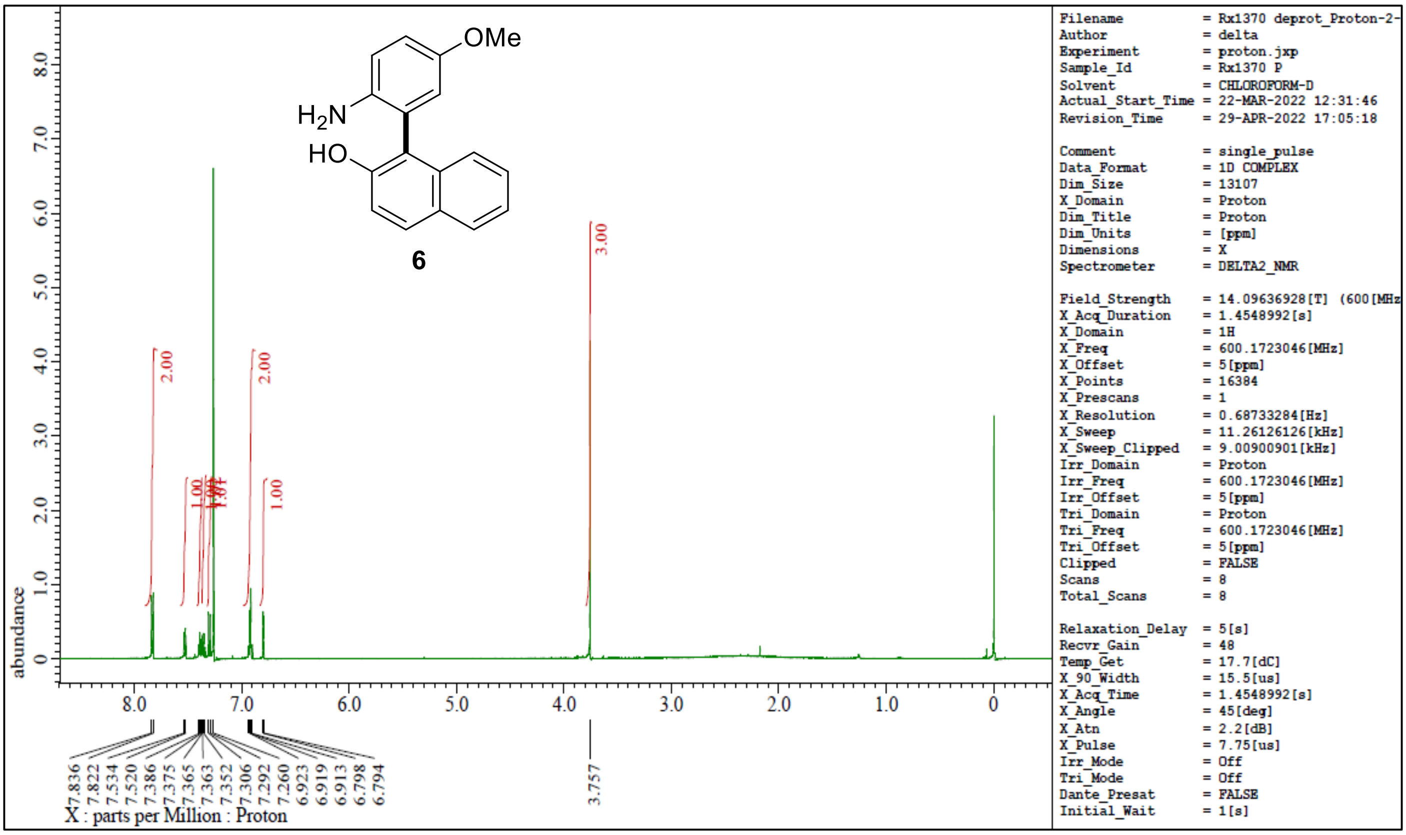

Compound **6** (^{1}H NMR, 600 MHz, $CDCl_3$).

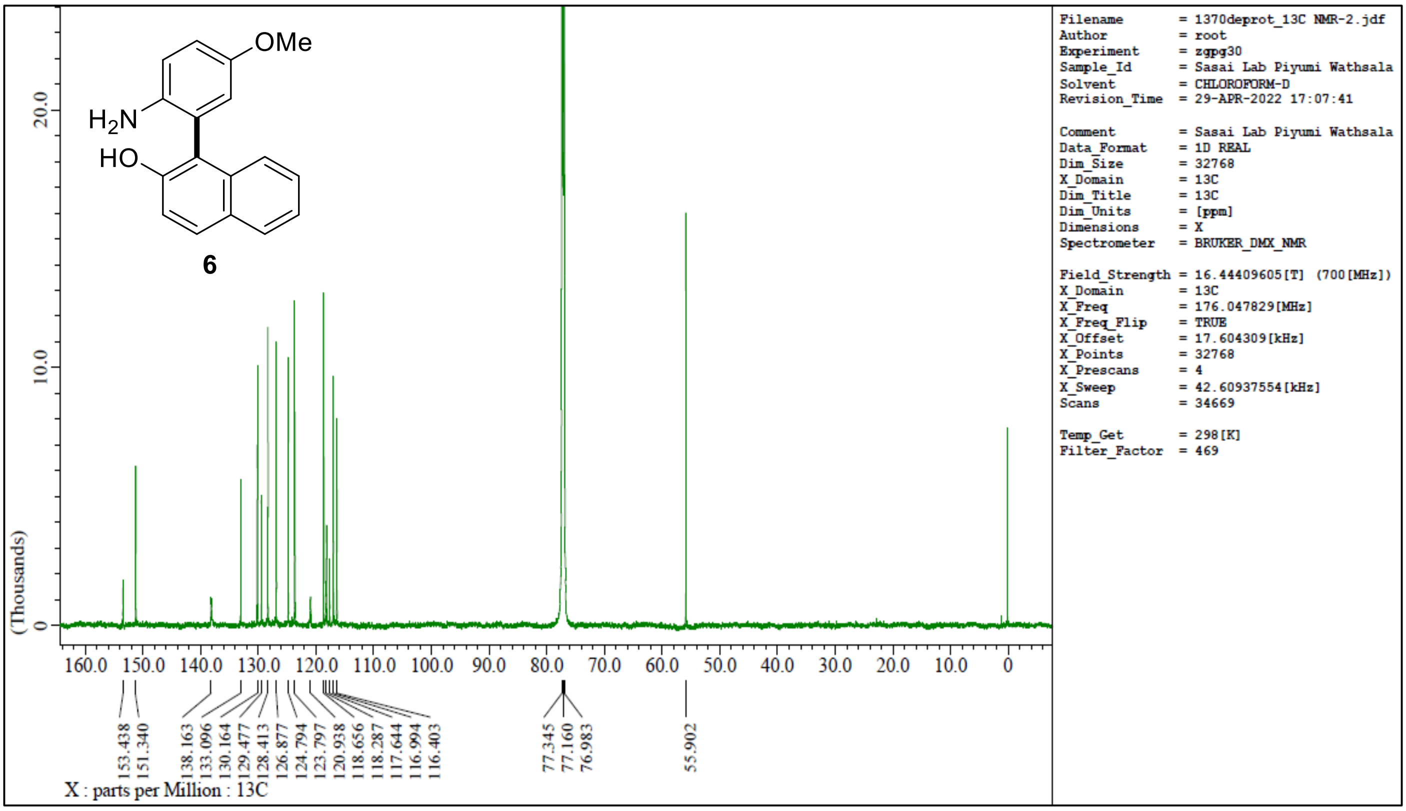

Compound **6** (^{13}C NMR, 175 MHz, $CDCl_3$).

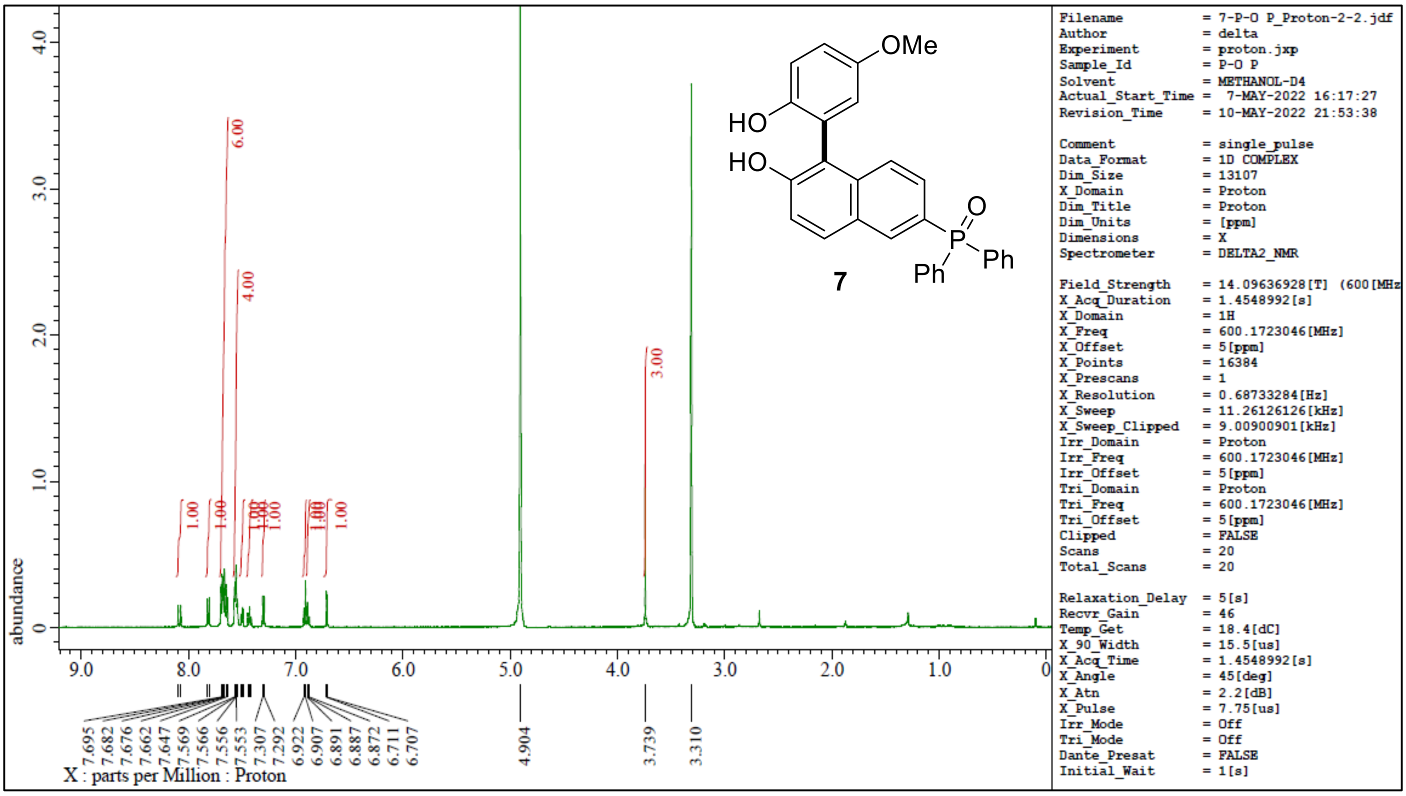

Compound **7** (^{1}H NMR, 600 MHz, CD_3OD).

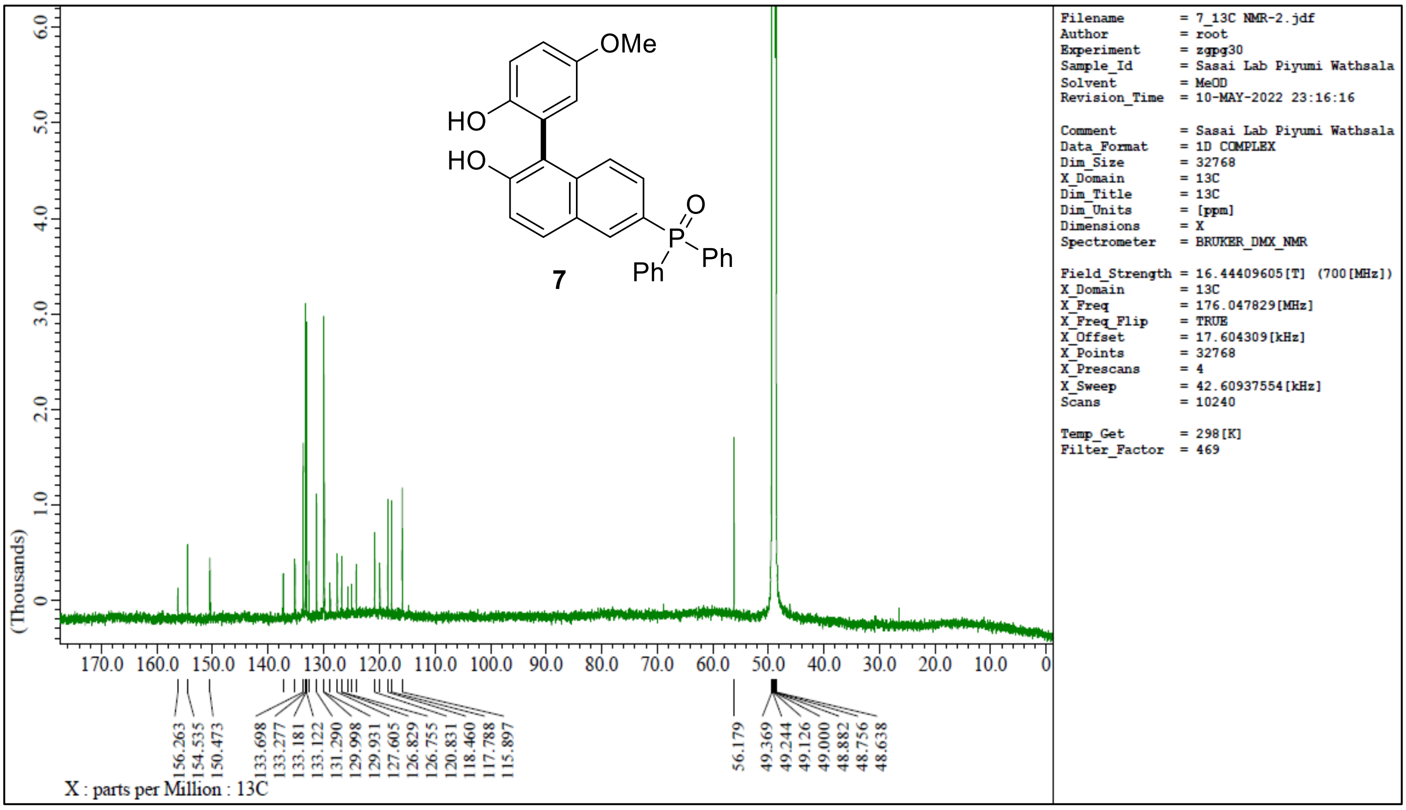

Compound **7** (^{13}C NMR, 175 MHz, CD_3OD).

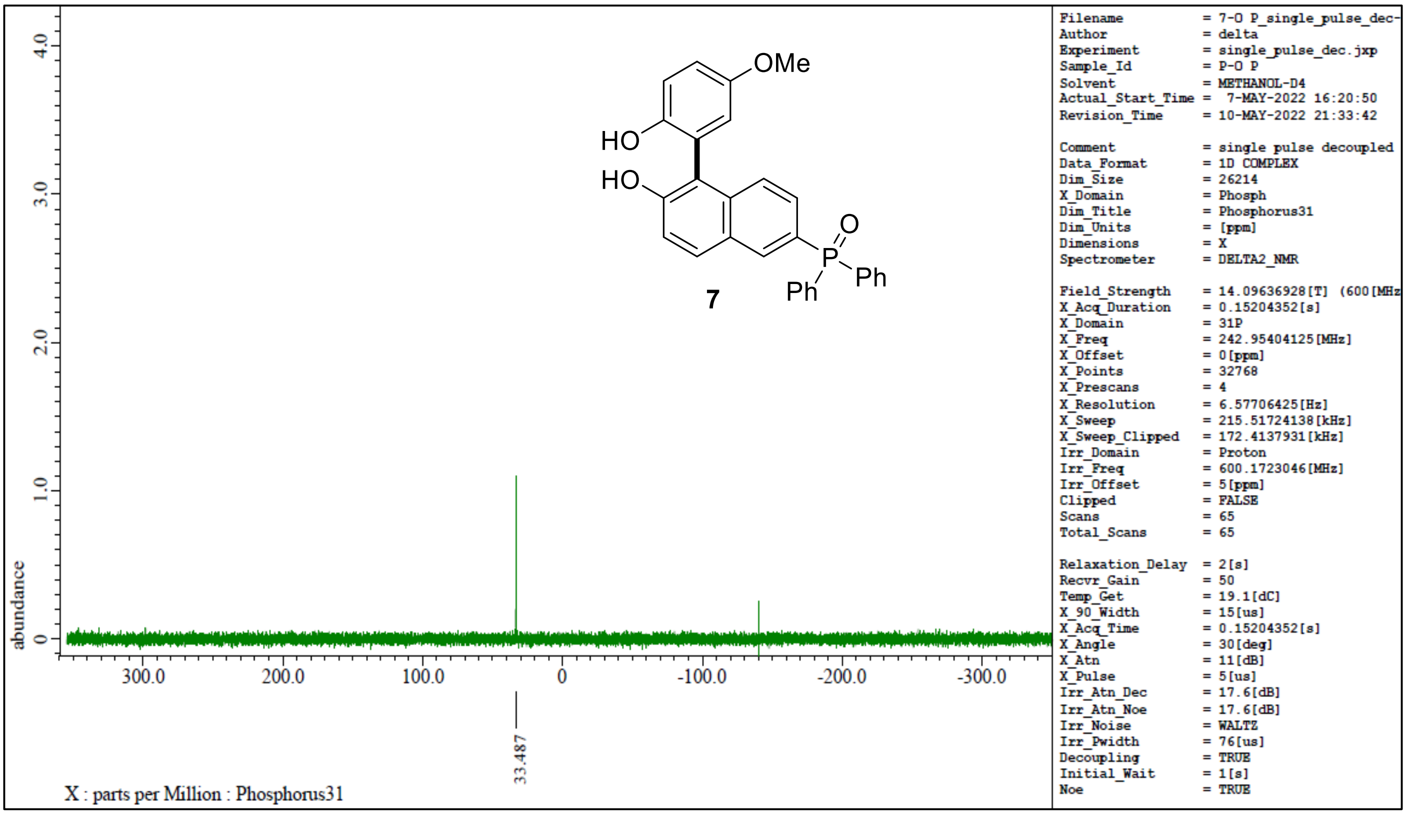

Compound **7** (^{31}P NMR, 243 MHz, CD_3OD).
