# Supplementary material for: Bayesian optimization-driven parallel-screening of multiple parameters for the flow synthesis of biaryl compounds
Source: Commun Chem. 2022 Nov 10;5:148. doi: 10.1038/s42004-022-00764-7 (PMC9814103; doi:10.1038/s42004-022-00764-7)
Supplement: Supplementary file 5 — Supplementary Data 2 [file 42004_2022_764_MOESM5_ESM.pdf]

Scripts for categorical BO

Bayesian Optimization using GPyOpt for e Table 1

```
from copy import deepcopy
import numpy as np
from numpy.random import seed
import GPy, GPyOpt

class GPdata_onehot():
    def __init__(self, X, Y=None, bounds=None, maximize=False):
        self.X = np.copy(X)
        self.bounds = deepcopy(bounds)
        if maximize:
            self.Y = np.copy(-Y)
        else:
            self.Y = np.copy(Y)

        self.mean_ = np.mean(X, axis=0)
        self.sd_ = np.sqrt(np.var(X, axis=0))

        if bounds is None:
            pass
        else:
            param_type = np.array([2 if bounds[i]['type']=='categorical' else 1 if bounds[i]['type'
            self.mean_[np.where(param_type==2)] = 0
            self.sd_[np.where(param_type==2)] = 1

        self.X_std =  (X - self.mean_) / self.sd_

        if bounds is None:
            pass
        else:
            self.bounds_std = deepcopy(bounds)
            for i in range(len(bounds)):
                self.bounds_std[i]['domain'] = tuple(
                    (np.array(self.bounds[i]['domain']) - self.mean_[i]) / self.sd_[i] )



    def de_std(self, x, index=None):

        if index is None:
            return x * self.sd_ + self.mean_
        else:
            return x * self.sd_[index] + self.mean_[index]

    def en_std(self, x, index=None):#

        if index is None:
            return (x - self.mean_) / self.sd_
        else:
            return (x - self.mean_[index]) / self.sd_[index]


    def append(self, x, y=None):
        self.X = np.append(self.X, x, axis=0)
        if y is None:
            pass
        else:
            self.Y = np.append(self.Y, y, axis=0)

        self.mean_ = np.mean(X, axis=0)
```

```python
        self.sd_ = np.sqrt(np.var(X, axis=0))
        self.std =  (X - self.mean_) / self.sd_
        return self

    def delete(self, i):
        self.X = np.delete(self.X, obj=i, axis=0)
        if self.Y is None:
            pass
        else:
            self.Y = np.delete(self.Y, obj=i, axis=0)

        self.mean_ = np.mean(X, axis=0)
        self.sd_ = np.sqrt(np.var(X, axis=0))
        self.std =  (X - self.mean_) / self.sd_
        return self
```

```python
X = np.array([[0, 2, 1, 60., 0.05, 0.05],
              [0, 3, 0.5, 40., 0.01, 0.2],
              [1, 1, 2, 60., 0.01, 0.1],
              [1, 3, 0.5, 20., 0.1, 0.1],
              [2, 1, 2, 40., 0.05, 0.05],
              [2, 2, 1, 20., 0.1, 0.2],
              [2, 2, 1, 20, 0.1, 0.15],
              [0, 2.3, 1, 55, 0.039, 0.04],
              [1, 1.1, 2.4, 85, 0.15, 0.1],
              [2, 1.3, 1.2, 15, 0.11, 0.15],
              [2, 2.1, 1.1, 30, 0.061, 0.15],
              [0, 2.8, 1, 50, 0.014, 0.11],
              [0, 3.4, 1.3, 55, 0.01, 0.032],
              [0, 2.2, 1.7, 15, 0.1, 0.014],
              [0, 3.0, 1.5, 25, 0.015, 0.08]])

Y = np.array([68, 73, 42, 28, 55, 75, 81, 77, 40, 78, 76, 79, 88, 44, 96


             ])[:, np.newaxis]


bounds = [{'name': 'mixer_type', 'type': 'categorical', 'domain': (0, 1, 2)},
          {'name': 'X_eq', 'type': 'continuous', 'domain': (1,3.5)},
          {'name': 'TfOH', 'type': 'continuous', 'domain': (0.25,2.5)},
          {'name': 'temp', 'type': 'continuous', 'domain': (15.,90.)},
          {'name': 'conc.', 'type': 'continuous', 'domain': (0.01,0.15)},
          {'name': 'flow_rate', 'type': 'continuous', 'domain': (0.01,0.15)}]
```

```python
def one_hot_dic(klass):

    one_hot_vec = np.eye(len(klass), dtype='uint8').tolist()
    return {klass[i]: one_hot_vec[i]  for i in range(len(klass))}

def flatten(l):

    for el in l:
        if isinstance(el, collections.abc.Iterable) and not isinstance(el, (str, bytes)):
            yield from flatten(el)
        else:
            yield el

def decode_one_hot(X, bounds):

    X_deonehot = []
    X_temp = np.copy(X)
    j = 0 #working index
    for i, row in enumerate(bounds):
        if row['type'] == 'categorical':
            X_deonehot.append(
                [row['domain'][k] for k in np.argmax(X_temp[:, j:j+len(row['domain'])], axis=1)]
            )
            j += len(row['domain'])
        else:
            X_deonehot.append(
                X_temp[:, j].tolist()
            )
            j += 1

    return np.array(X_deonehot).T
```

```
X_onehot = np.copy(X).tolist()

for i, row_i in enumerate(X_onehot):
    for j, row_bound in enumerate(bounds):
        if row_bound['type'] == 'categorical':
            one_hot_vec = one_hot_dic(row_bound['domain'])
            row_i[j] = one_hot_vec[X[i][j]]
        else:
            pass
    X_onehot[i] = list(flatten(X_onehot[i]))

X_onehot = np.array(X_onehot)

bounds_onehot = []
param_types_onehot = []

for i, row in enumerate(bounds):
    if row['type'] == 'categorical':
        for j in range(len(row['domain'])):
            bounds_onehot.append(
                {'name': row['name'] + '_cat' + str(row['domain'][j]), 'type': 'continuous', 'domair
            )
        param_types_onehot.append([3] * len(row['domain']))
    else:
        bounds_onehot.append(row)
        param_types_onehot.append([0 if row['type'] == 'continuous' else 1])

mean_ = np.mean(X_onehot, axis=0)
sd_ = np.sqrt(np.var(X_onehot, axis=0))

param_types_flat = np.array(list(flatten(param_types_onehot)))
mean_[np.where(param_types_flat==3)] = 0
sd_[np.where(param_types_flat==3)] = 1

X_onehot_std =  (X_onehot - mean_) / sd_
```

```
bounds_onehot_std = deepcopy(bounds_onehot)
for i in range(len(bounds_onehot)):
                bounds_onehot_std[i]['domain'] = tuple(
                    (np.array(bounds_onehot[i]['domain']) - mean_[i]) / sd_[i] )

print('X_onehot_std¥n', X_onehot_std)
print('bounds_onehot_std')
bounds_onehot_std
```

```
initial_x = X_onehot_std
initial_y = -Y
domain = bounds_onehot_std
```

```
batch_size = 3
num_cores = 3

myBopt = GPyOpt.methods.BayesianOptimization(f=None,
                                              domain=domain,
                                              X = initial_x,
                                              Y = initial_y,
                                              acquisition_type='LCB',
                                              exploration_weight=4,
                                              normalize_Y=True,
                                              exact_feval=False,
                                              evaluator_type ='local_penalization',
                                              batch_size = batch_size,
                                              num_cores = num_cores,
                                              kernel  = GPy.kern.RBF(len(domain))
                                             )


next_x = myBopt.suggest_next_locations()
next_x_destd = next_x * sd_ + mean_
print(decode_one_hot(next_x_destd, bounds))
```

```
from copy import deepcopy
import numpy as np
from numpy.random import seed
import GPy, GPyOpt

class GPdata_onehot():
    def __init__(self, X, Y=None, bounds=None, maximize=False):
        self.X = np.copy(X)
        self.bounds = deepcopy(bounds)
        if maximize:
            self.Y = np.copy(-Y)
        else:
            self.Y = np.copy(Y)

        self.mean_ = np.mean(X, axis=0)
        self.sd_ = np.sqrt(np.var(X, axis=0))

        if bounds is None:
            pass
        else:
            param_type = np.array([2 if bounds[i]['type']=='categorical' else 1 if bounds[i]['type'
            self.mean_[np.where(param_type==2)] = 0
            self.sd_[np.where(param_type==2)] = 1

        self.X_std =  (X - self.mean_) / self.sd_

        if bounds is None:
            pass
        else:
            self.bounds_std = deepcopy(bounds)
            for i in range(len(bounds)):
                self.bounds_std[i]['domain'] = tuple(
                    (np.array(self.bounds[i]['domain']) - self.mean_[i]) / self.sd_[i] )



    def de_std(self, x, index=None):

        if index is None:
            return x * self.sd_ + self.mean_
        else:
            return x * self.sd_[index] + self.mean_[index]

    def en_std(self, x, index=None):#

        if index is None:
            return (x - self.mean_) / self.sd_
        else:
            return (x - self.mean_[index]) / self.sd_[index]


    def append(self, x, y=None):
        self.X = np.append(self.X, x, axis=0)
        if y is None:
            pass
        else:
            self.Y = np.append(self.Y, y, axis=0)

        self.mean_ = np.mean(X, axis=0)
```

```python
        self.sd_ = np.sqrt(np.var(X, axis=0))
        self.std =  (X - self.mean_) / self.sd_
        return self

    def delete(self, i):
        self.X = np.delete(self.X, obj=i, axis=0)
        if self.Y is None:
            pass
        else:
            self.Y = np.delete(self.Y, obj=i, axis=0)

        self.mean_ = np.mean(X, axis=0)
        self.sd_ = np.sqrt(np.var(X, axis=0))
        self.std =  (X - self.mean_) / self.sd_
        return self
```

```python
X = np.array([[0, 2., 60., 0.05, 0.05, 1],
              [0, 3., 40., 0.01, 0.2, 0.5],
              [1, 1., 60., 0.01, 0.1, 2],
              [1, 3., 20., 0.1, 0.1, 0.5],
              [2, 1., 40., 0.05, 0.05, 2],
              [2, 2., 20., 0.1, 0.2, 1],
              [0, 3., 5, 0.14, 0.048, 0.29],
              [1, 3.5, 15, 0.035, 0.087, 0.25],
              [2, 3.5, 30, 0.15, 0.022, 0.98],
              [0, 3.4, 35, 0.056, 0.13, 0.37],
              [1, 2.4, 15, 0.019, 0.041, 0.34],
              [2, 2.4, 15, 0.011, 0.06, 0.73],
              [1, 3.2, 15, 0.068, 0.062, 0.25],
              [1, 3.5, 15, 0.067, 0.097, 0.53],
              [1, 3.2, 30, 0.044, 0.068, 0.35]])

Y = np.array([14, 36, 11, 52, 4, 29, 20, 58, 19, 10, 40, 19, 20, 43, 69


             ])[:, np.newaxis]


bounds = [{'name': 'mixer_type', 'type': 'categorical', 'domain': (0, 1, 2)},
          {'name': 'X_eq', 'type': 'continuous', 'domain': (1,3.5)},
          {'name': 'temp', 'type': 'continuous', 'domain': (15.,90.)},
          {'name': 'conc.', 'type': 'continuous', 'domain': (0.01,0.15)},
          {'name': 'flow_rate', 'type': 'continuous', 'domain': (0.01,0.15)},
         {'name': 'TfOH', 'type': 'continuous', 'domain': (0.25,2.5)}]
```

```python
def one_hot_dic(klass):

    one_hot_vec = np.eye(len(klass), dtype='uint8').tolist()
    return {klass[i]: one_hot_vec[i]  for i in range(len(klass))}

def flatten(l):

    for el in l:
        if isinstance(el, collections.abc.Iterable) and not isinstance(el, (str, bytes)):
            yield from flatten(el)
        else:
            yield el

def decode_one_hot(X, bounds):

    X_deonehot = []
    X_temp = np.copy(X)
    j = 0 #working index
    for i, row in enumerate(bounds):
        if row['type'] == 'categorical':
            X_deonehot.append(
                [row['domain'][k] for k in np.argmax(X_temp[:, j:j+len(row['domain'])], axis=1)]
            )
            j += len(row['domain'])
        else:
            X_deonehot.append(
                X_temp[:, j].tolist()
            )
            j += 1

    return np.array(X_deonehot).T
```

```
X_onehot = np.copy(X).tolist()

for i, row_i  in enumerate(X_onehot):
    for j, row_bound in enumerate(bounds):
        if row_bound['type'] == 'categorical':
            one_hot_vec = one_hot_dic(row_bound['domain'])
            row_i[j] = one_hot_vec[X[i][j]]
        else:
            pass
    X_onehot[i] = list(flatten(X_onehot[i]))

X_onehot = np.array(X_onehot)

bounds_onehot = []
param_types_onehot = []

for i, row in enumerate(bounds):
    if row['type'] == 'categorical':
        for j in range(len(row['domain'])):
            bounds_onehot.append(
                {'name': row['name'] + '_cat' + str(row['domain'][j]), 'type': 'continuous', 'domair
            )
        param_types_onehot.append([3] * len(row['domain']))
    else:
        bounds_onehot.append(row)
        param_types_onehot.append([0 if row['type'] == 'continuous' else 1])

mean_ = np.mean(X_onehot, axis=0)
sd_ = np.sqrt(np.var(X_onehot, axis=0))

param_types_flat = np.array(list(flatten(param_types_onehot)))
mean_[np.where(param_types_flat==3)] = 0
sd_[np.where(param_types_flat==3)] = 1

X_onehot_std =  (X_onehot - mean_) / sd_
```

```
bounds_onehot_std = deepcopy(bounds_onehot)
for i in range(len(bounds_onehot)):
                bounds_onehot_std[i]['domain'] = tuple(
                    (np.array(bounds_onehot[i]['domain']) - mean_[i]) / sd_[i] )

print('X_onehot_std¥n', X_onehot_std)
print('bounds_onehot_std')
bounds_onehot_std
```

```
initial_x = X_onehot_std
initial_y = -Y
domain = bounds_onehot_std
```

```
batch_size = 3
num_cores = 3

myBopt = GPyOpt.methods.BayesianOptimization(f=None,
                                             domain=domain,
                                             X = initial_x,
                                             Y = initial_y,
                                             acquisition_type='LCB',
                                             exploration_weight=4,
                                             normalize_Y=True,
                                             exact_feval=False,
                                             evaluator_type ='local_penalization',
                                             batch_size = batch_size,
                                             num_cores = num_cores,
                                             kernel = GPy.kern.RBF(len(domain))
                                            )


next_x = myBopt.suggest_next_locations()
next_x_destd = next_x * sd_ + mean_
print(decode_one_hot(next_x_destd, bounds))
```
