# Supplementary material for: Bayesian optimization-driven parallel-screening of multiple parameters for the flow synthesis of biaryl compounds
Source: Commun Chem. 2022 Nov 10;5:148. doi: 10.1038/s42004-022-00764-7 (PMC9814103; doi:10.1038/s42004-022-00764-7)
Supplement: Supplementary file 1 — Takizawa_PR File [file 42004_2022_764_MOESM1_ESM.pdf]

nature portfolio

Peer Review File

Bayesian optimization-driven parallel-screening of multiple parameters for the flow synthesis of biaryl compounds

Open Access This file is licensed under a Creative Commons Attribution 4.0 International License, which permits use, sharing, adaptation, distribution and reproduction in any medium or format, as long as you give appropriate credit to the original author(s) and the source, provide a link to the Creative Commons license, and indicate if changes were made. In the cases where the authors are anonymous, such as is the case for the reports of anonymous peer reviewers, author attribution should be to 'Anonymous Referee' followed by a clear attribution to the source work. The images or other third party material in this file are included in the article's Creative Commons license, unless indicated otherwise in a credit line to the material. If material is not included in the article's Creative Commons license and your intended use is not permitted by statutory regulation or exceeds the permitted use, you will need to obtain permission directly from the copyright holder. To view a copy of this license, visit http://creativecommons.org/licenses/by/4.0/.

Reviewers' comments:

Reviewer #1 (Remarks to the Author):

I recommend major revisions to the submitted manuscript, prior to publication. Please see the attached pdf for detailed comments.

Reviewer #2 (Remarks to the Author):

The manuscript titled "Bayesian optimization-driven parallel-screening on multi-parameters of micromixer-type and organocatalytic conditions in the flow biaryl synthesis" written by S. Takizawa and coworkers reported optimization of reaction conditions for Brønsted acid catalyzed biaryl couplings using machine-learning, Bayesian optimization. The authors treated a categorical variable (mixer type) via one-hot encoding in this study. This is a simple and rather traditional approach. The authors identified flow conditions with shorter reaction times and lower catalyst loadings, although larger amounts of substrate 2 was required compared with the previously reported conditions. Gram scale syntheses was demonstrated via the developed flow conditions. Overall, it is somewhat difficult for this reviewer to support the submitted manuscript for publication in Communication Chemistry at the present stage. The reasons were shown below.

page 3, introduction section
The really pioneering work for multi-parameter screening and optimization of a chemical reaction reported by Lapkin, Bourne, and coworkers (Chem. Eng. J. 352, (2018) 277-282) should be cited in this manuscript. This Chem. Eng. J. manuscript reported the use of more advanced "multi-objective" Bayesian optimization in optimizing conditions of automated continuous-flow synthesis.

page 6
The authors indicated search range for temperature (20-60 oC) in line 3, however, the search range was changed during the optimization (15-90 oC) as shown in S54. It should be consistent during the optimization.

page 6
The authors described "the desired product 3a was obtained in 93% isolated yield within 15 min..." Readers might think the reaction and purification were finished within 15 min. This sentence should be revised.

page 8
Interestingly, the optimal mixer was different between the first reaction and the second reaction. The authors have better to show the mixing efficiency and heat transfer efficiency of the used mixers. This information should be valuable for readers to speculate the reason why the optimal mixer was different.

page 10
The optimal catalyst loading and concentration of 4 were significantly different between the first and the second reaction. This was also interesting point of this study. The authors should discuss the reason based on the plausible reaction mechanisms.

S2, SI
Although brief explanation for T-shaped and beta-mixers was shown in ref. 50, the information should be also shown in general information in SI.

S4, SI
When flow rate was changed, how was reaction time? Was reaction time maintained by changing the

length of tubes? It should be mentioned in SI.

Takizawa, Washio and coworkers present a Bayesian optimization approach to synthesizing amino hydroxybiaryls and dihidroxybiaryls. The optimization algorithm considers five numerical (continuous) optimization variables and one categorical (discrete) optimization variable. The optimization is carried out in an "offline" fashion, without closed-loop experimentation, in batches of three experiments, quantified by NMR analysis. The optimization algorithm is performed first for iminoquinone monoacetal substrates, then later for quinone monoacetal substrates, finding different optima.

The manuscript is, at face value, an interesting advance in the field, with synthetic utility. The research would be far more impactful if a closed-loop approach could be incorporated, but the present approach still appears to expedite the optimization process in an effective manner. However, there are numerous questionable statements, assumptions and practices within the manuscript and supporting information. As a researcher in the field of flow chemistry, these issues appear relatively major and require additional explanation and clarification before the manuscript can be published. Following successful incorporation of such major revisions, I would recommend publication in *Communications Chemistry*.

Major issues, which must be addressed prior to publication:

1. The description of the experimental setup and procedure is significantly lacking. At present, there is far from enough detail for a reader to be able to reproduce this chemistry. The main problems are as follows:
    a. No volume of the microreactor setup is provided. **This is vital information to calculate the reaction time, without which the manuscript is of little value**.
    b. The discussed mixers (Coment-01-X and beta-type mixer) have no details**. It isn't acceptable for chemistry to be described using secret/proprietary equipment, without any detailed description. This will make the work fundamentally irreproducible by others in the field.** The Comet mixer is stated to be manufactured by "Techno Application", yet I cannot find any details of such a product from this company.

2. Details around the experimental procedure are also lacking. Please address the following:
    a. How much of the reactor output was collected for workup and yield calculation? If the entire 2.2 mL solution was passed through the reactor (of currently unknown volume), there will always be 1 reactor volume left inside the reactor at the end.
    b. For each substrate, the isolated mass should be added, rather than just a % yield.
    c. Was the reaction mixture directly quenched into $NaHCO_3$, or was the reaction mixture first collected, then quenched? If the latter, this doesn't represent a sensible flow process! At 0.08 mL/min, the 2.2 mL syringe will take 27.5 min to empty, meaning that the reactor output has a significant amount of additional reaction time in the collection flask.
    d. In the experimental, it is stated "After the continuous-flow was kept within residence time" – what does this mean? Please clarify.

3. Overall, I am somewhat skeptical of the results reported. There is no evidence to suggest that the reaction is one that should benefit from continuous flow processed (i.e. mixing limited or exothermic).

a. What is the justification for this improvement compared with the present literature (reference 46)? Flow processing cannot be stated to magically improve the reaction rate and yield without justification. Fundamental reaction kinetics do not change in flow!
b. It is difficult to see how the three mixers can be differentiated, particularly since this reaction does not appear to be mixing limited. The use of BO in this case doesn't entirely make sense – the selection of an "optimal mixer" for the two different substrate classes is likely only achieved by chance, due to the selected experiments. The authors would have to perform a head-to-head comparison of the mixers (**using the same conditions**) to claim that there is any real difference.

Additional minor points for consideration:

1. The referenced literature background on the (relatively advanced) field of BO in flow is somewhat lacking. As a minimum, the following significant contributions from the groups of Bourne, Lapkin, Kappe and Jensen should be added (around the position of reference 15):
    a. 10.1016/j.cej.2018.07.031
    b. 10.1002/cmtd.202000051
    c. 10.1002/advs.202105547
    d. 10.1021/acscentsci.2c00207

2. Use of "et al." in many references makes it difficult to recognize which papers are being cited at a glance. For reviewing purposes, it would be preferable for the authors not to do this.

3. In the abstract and introduction, the phrase "machine-engineering" is used. I haven't heard this term before - perhaps "reactor design" or simply "engineering" would be more suitable.

4. The formatting of the introduction makes it quite difficult to read (first paragraph is 2 pages long). Please split this up into shorter, more focused paragraphs.

5. The references selected to support the first introductory sentence (1-6) are somewhat strange. Why focus on the specific areas of electrochemical and microwave reactions? Surely it's more sensible to provide references to reviews on the areas of data-rich experimentation in general?

6. In the description of one-hot encoding, the use of "via" is not really appropriate. Perhaps "represented by" would be more suitable.

7. As stated above, the flow rates aren't really the variable being examined – the important result of this is varying the reaction time (residence time). It would probably be more suitable to discuss residence times, rather than flow rates (or at least have the reactor volume to hand, so the calculation can be made quickly by the reader).

8. "With a batch size of three" is stated, yet there are 6 results in the first set. It is assumed that this represents an initial exploratory data set before the batches of 3 experiments, however, this is not explicitly mentioned. Some words should be added, to this effect.

9. "Finally, we acquired three different experimental **datasets** (entries 13–15)." These aren't datasets, but single data**points**.

10. The experiment performed in Fig. 5 is almost the exact same control experiment from reference 46. This doesn’t add any value to the work, since (unsurprisingly) the same result is observed.

11. Reference 51 states that methods other than parallel LCB were unsuccessful, however there is no discussion of what parallel LCB actually entails. I also could not readily find this information online. Could the authors add a reference to a paper explaining this? Or add further explanation in the citation here?

12. The representation of the “micro mixer” is somewhat inaccurate. There is a T-piece combining the two streams, followed by a mixing element. However, it is assumed that the “T-shaped” experiments are performed in the absence of a micro mixer? In this case, it would be more representative to write “no mixer” instead of “T-shaped”. Similarly, how does this affect the total reactor volume? Do the “T-shaped” experiments have a smaller volume?

13. The experiments are quantified as follows: “1,3,5-Trimethoxybenzene was used as an internal standard.” The authors should add an experimental procedure for these optimization experiments, explicitly stating when the “internal” standard was added. For a true internal standard, this should be added to one of the reaction streams – however 1,3,5-trimethoxybenzene can be reactive in some cases, so it is assumed that this was added after the reaction (often called an “external standard”).

Professor Ph.D Takashi Washio
Associate Professor Ph.D Shinobu Takizawa
The Institute of Scientific and Industrial Research,
Osaka University
Mihogaoka, Ibaraki, Osaka 567-0047, JAPAN
Phone: (+81) 6-6879-8467
FAX: (+81) 6-6879-8469
e-mail: washio@ar.sanken.osaka-u.ac.jp
taki@sanken.osaka-u.ac.jp

Response to referees

Reviewer 1 comments

Takizawa, Washio and coworkers present a Bayesian optimization approach to synthesizing amino hydroxybiaryls and dihidroxybiaryls. The optimization algorithm considers five numerical (continuous) optimization variables and one categorical (discrete) optimization variable. The optimization is carried out in an "offline" fashion, without closed-loop experimentation, in batches of three experiments, quantified by NMR analysis. The optimization algorithm is performed first for iminoquinone monoacetal substrates, then later for quinone monoacetal substrates, finding different optima. The manuscript is, at face value, an interesting advance in the field, with synthetic utility. The research would be far more impactful if a closed-loop approach could be incorporated, but the present approach still appears to expedite the optimization process in an effective manner. However, there are numerous questionable statements, assumptions and practices within the manuscript and supporting information. As a researcher in the field of flow chemistry, these issues appear relatively major and require additional explanation and clarification before the manuscript can be published. Following successful incorporation of such major revisions, I would recommend publication in Communications Chemistry.

Major issues, which must be addressed prior to publication:
Reviewer 1-1: The description of the experimental setup and procedure is significantly lacking. At present, there is far from enough detail for a reader to be able to reproduce this chemistry. The main problems are as follows:
a. No volume of the microreactor setup is provided. This is vital information to calculate the reaction time, without which the manuscript is of little value.
b. The discussed mixers (Coment-01-X and beta-type mixer) have no details. It isn't acceptable for chemistry to be described using secret/proprietary equipment, without any detailed description. This will make the work fundamentally irreproducible by others in the field. The Comet mixer is stated to be manufactured by "Techno Application", yet I cannot find any details of such a product from this company.
Response: According to the reviewer 1 comments, we provided volume of microreactor (see also the footnote of Tables 1 and 2).
Volume of Comet X microreactor = 2.4 mL. Volume of β-type microreactor = 2.7 mL. Volume of T-shaped microreactor = 1.6 mL.
In addition, we described the discussed mixers as following (see also Fig. S2 in ESI).

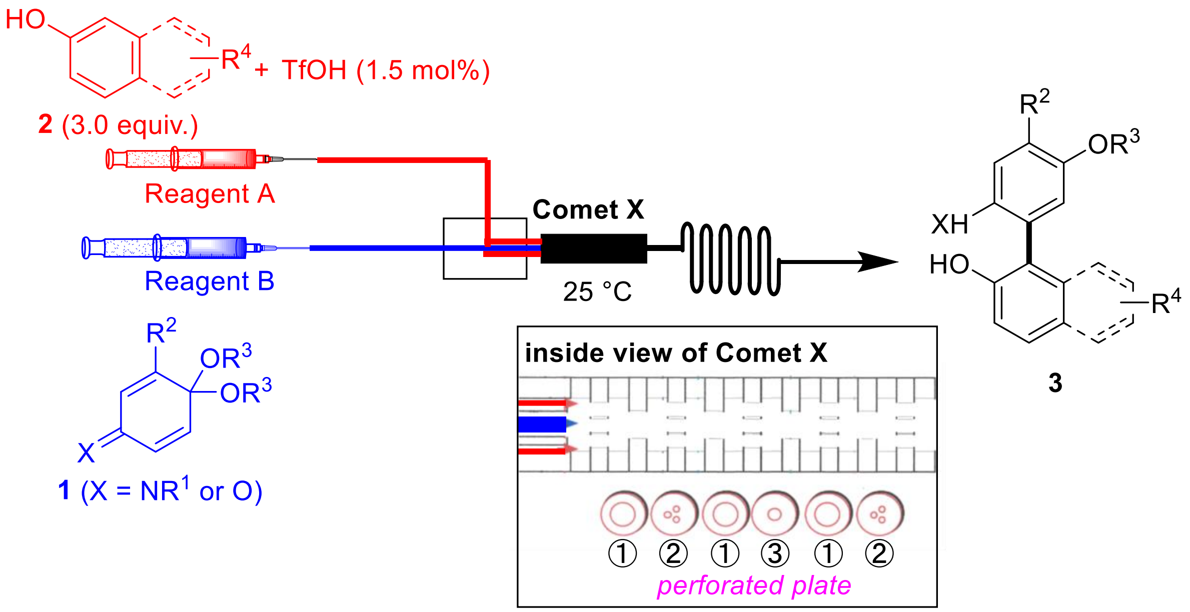

Comet X (Total solution holding capacity: 29.4 μL) equips 19 mixing spaces made by three different types of perforated-plates ①②③ in the stainless steel cylinder. Reagent B (substrate **1** in toluene) introduced to the start of the micro flow reactor from a 0.96 mm inner tube is surrounded by Reagent A (substrate **2** and TfOH in toluene), which has moved through a gap of 2.0 mm outer tube inner diameter and 1.56 mm outer tube diameter, over the entire surface area of the cylindrical release. After the first mixing of Reagent A/Reagent B, the mixture is pushed into three of ϕ0.5 hole on the plate immediately. In the micro spaces, Reagent B enters from the circumference side along the centre of the entire micro flow reactor, and the mixture enters from the circumference outside the centre. In the three-hole spaces, Reagent B enters from the circumference side along the centre of the entire microflow reactor, and Reagent A enters from the circumference outside the centre. The mass transfer is extremely accelerated due to concentration gradient. And then the reaction takes place at the hall. The mixture divided into 3 parts are again integrated into one channel of ϕ 1.0 from the hall of ϕ 2.0. These results in a more concentrated mixing of the reagents and further reaction. Then, it passes through the ϕ 2.0 hall again and is dispersed into three ϕ 0.5 micro spaces.
The Micro Flow Reactor [Comet X] repeats the above process totally five times.

References on the flow reaction with Comet X mixer:

1) H. Koo, H. Y. Kim, K. Oh, (*E*)-Selective Friedel–Crafts acylation of alkynes to β-chlorovinyl ketones: Defying isomerizations in batch reactions by flow chemistry approaches. *Org. Chem. Front.* **2019**, *6*, 1868-1872.
2) T. Doi, H. Otaka, K. Umeda, M. Yoshida, Study for diastereoselective aldol reaction in flow: Synthesis of (*E*)-(*S*)-3-hydroxy-7-tritylthio-4-heptenoic acid, a key component of cyclodepsipeptide HDAC inhibitors. *Tetrahedron*, **2015**, *71*, 6463-6470.
3) A. R. Pradipta, A. Tsutsui, A. Ogura, S. Hanashima, Y. Yamaguchi, A. Kurbangalieva, K. Tanaka, Microfluidic mixing of polyamine with acrolein enables the detection of the [4+4] polymerization of intermediary unsaturated imines: The properties of a cytotoxic 1,5-diazacyclooctane hydrogel. *Synlett*, **2014**, *25*, 2442-2446.
4) Y. Uchinashi, K. Tanaka, Y. Manabe, Y. Fujimoto, K. Fukase, Practical and efficient method for α-sialylation with an azide sialyl donor using a microreactor. *J. Carbohydr. Chem.* **2014**, *33*, 55-67.

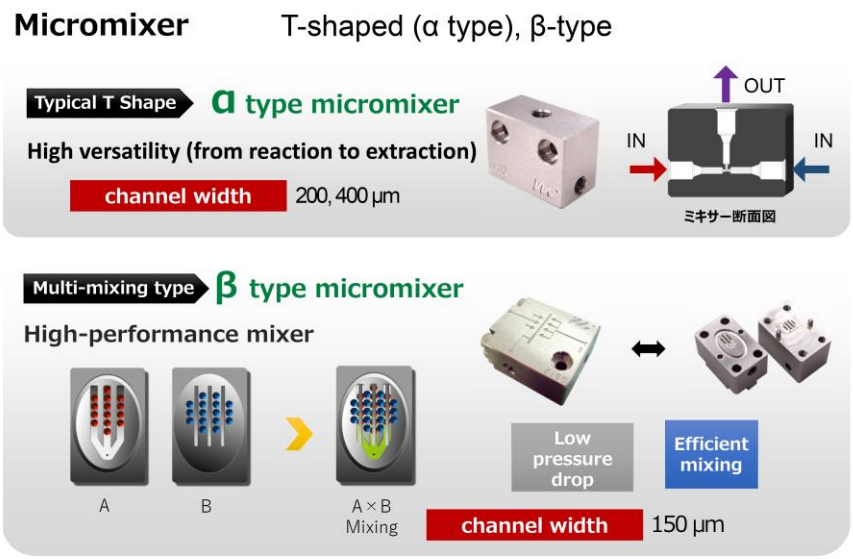

Mixing evaluation by Villermaux/Dushman reaction

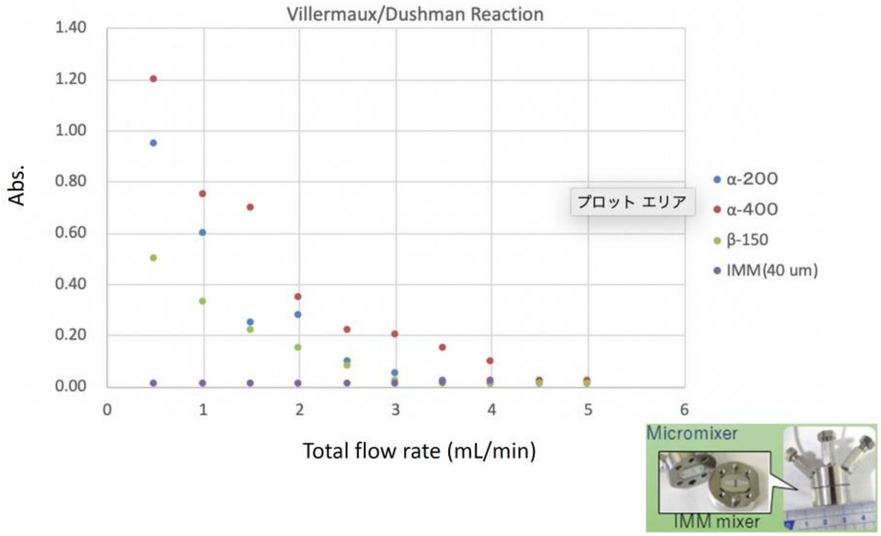

Pressure Drop

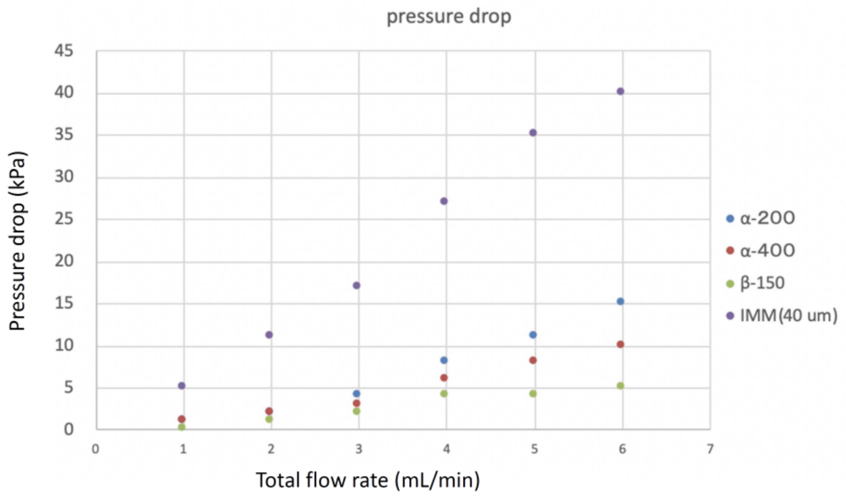

*T-shaped (MiChS CO., LTD.), Comet X-01-SS (Techno applications CO., LTD.), and β-type (MiChS CO., LTD.) mixers are made of stainless steel.

Reviewer 1-2: Details around the experimental procedure are also lacking. Please address the following:
a. How much of the reactor output was collected for workup and yield calculation? If the entire 2.2 mL solution was passed through the reactor (of currently unknown volume), there will always be 1 reactor volume left inside the reactor at the end.
b. For each substrate, the isolated mass should be added, rather than just a % yield.

c. Was the reaction mixture directly quenched into NaHCO3, or was the reaction mixture first collected, then quenched? If the latter, this doesn't represent a sensible flow process! At 0.08 mL/min, the 2.2 mL syringe will take 27.5 min to empty, meaning that the reactor output has a significant amount of additional reaction time in the collection flask.
d. In the experimental, it is stated "After the continuous-flow was kept within residence time" – what does this mean? Please clarify.
Response: We clarified these issues in the following modified general procedures. And the isolated mass was also added for each substrate (see ESI)

General procedure
As shown in Fig. 6, a flow microreactor system was dipped in oil bath to heat at 25 ˚C. A solution of **1a** (0.065 mmol, 0.015 M) in toluene (2.2 mL, syringe 1), and a solution of **2a** (0.195 mmol, 0.045 M) and TfOH (1.5 mol%) in toluene (2.2 mL, syringe 2) were introduced to the flow microreactor system by syringe pumps at a flow rate: 0.08 mL/min. The resulting solution was passed through Comet X mixer (total volume = 2.4 mL, residence time = 15 min) and directly forwarded to the quenching saturated aq. $NaHCO_3$ solution. After all the amount of toluene solutions were pumped, we pumped a fresh air to the flow microreactor at the same flow rate (0.08 mL/min) to avoid losing 1 reactor volume inside. Finally, the organic layer was extracted with EtOAc (15 mL × 3), dried over Na_2SO_4, concentrated in vacuo. The residue was purified by silica column chromatography (n-hexane/EtOAc) to afford **3a** (93%, 25.4 mg).

Reviewer 1-3: Overall, I am somewhat skeptical of the results reported. There is no evidence to suggest that the reaction is one that should benefit from continuous flow processed (i.e. mixing limited or exothermic).
a. What is the justification for this improvement compared with the present literature (reference 46)? Flow processing cannot be stated to magically improve the reaction rate and yield without justification. Fundamental reaction kinetics do not change in flow!
b. It is difficult to see how the three mixers can be differentiated, particularly since this reaction does not appear to be mixing limited. The use of BO in this case doesn't entirely make sense – the selection of an "optimal mixer" for the two different substrate classes is likely only achieved by chance, due to the selected experiments. The authors would have to perform a head-to-head comparison of the mixers (using the same conditions) to claim that there is any real difference.
Response: We assume the usage of TfOH in flow system is critical for this transformation. Using very strong Brønsted acids (e.g. TfOH), poor results were obtained in the previous work (*Angew. Chem., Int. Ed.* 55, 566–571 (2016)). Probably, rapid mixing of substrates and quenching of TfOH would improve yield by suppressing side reactions and decomposition of the substrates (see also ref 52). To show the superior performance of Comet X mixer, we performed a head-to-head comparison of the three mixers (shown in the next figure, see Scheme S1 in ESI).

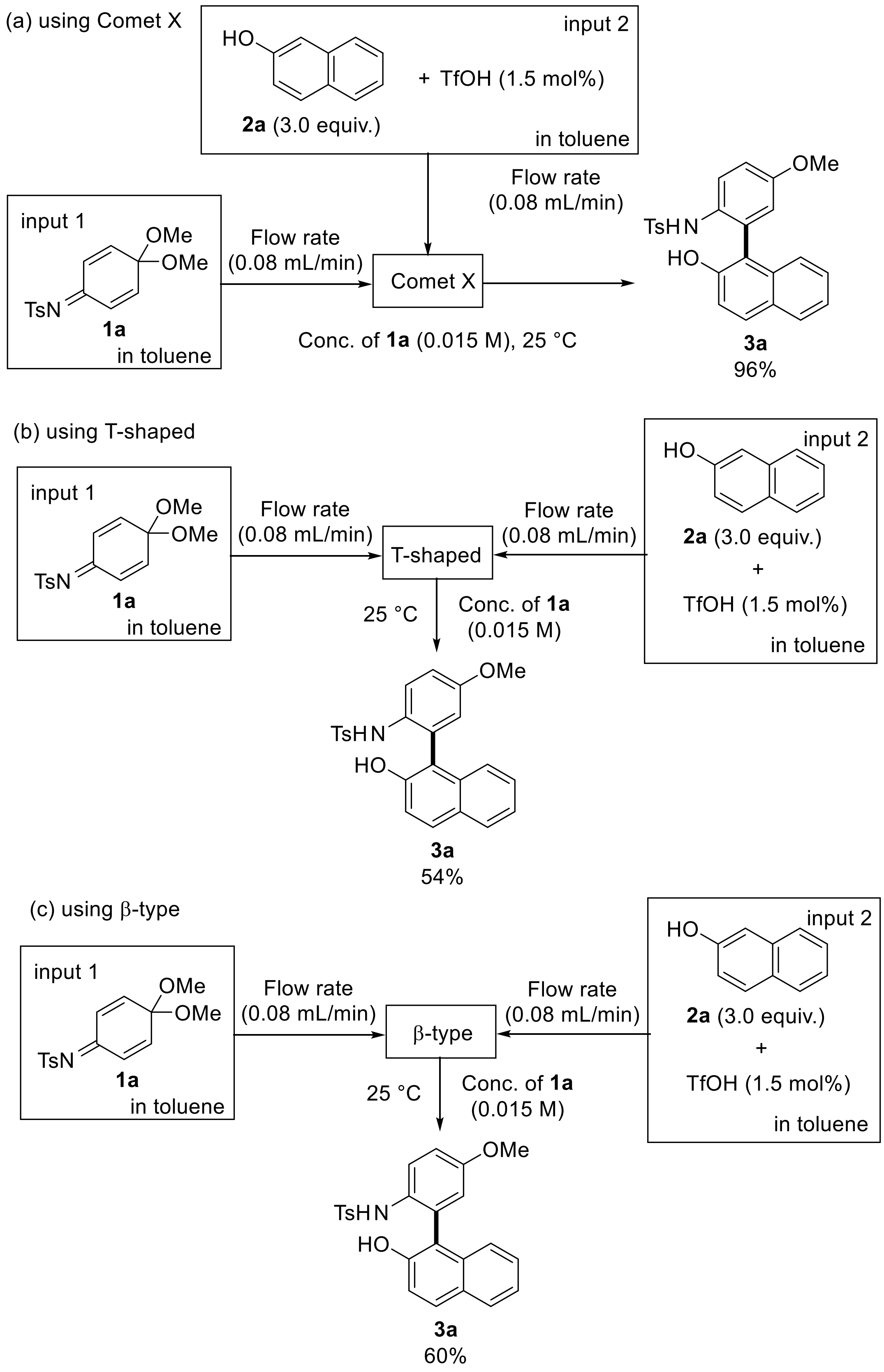

Additional minor points for consideration:

Reviewer 1-4: The referenced literature background on the (relatively advanced) field of BO in flow is somewhat lacking. As a minimum, the following significant contributions from the groups of Bourne, Lapkin, Kappe and Jensen should be added (around the position of reference 15):

a. 10.1016/j.cej.2018.07.031
b. 10.1002/cmtd.202000051
c. 10.1002/advs.202105547
d. 10.1021/acscentsci.2c00207

Response: These four reports were added to the reference section (see ref 16-19).

Reviewer 1-5: Use of "et al." in many references makes it difficult to recognize which papers are being cited at a glance. For reviewing purposes, it would be preferable for the authors not to do this.
Response: We added all authors to each reference.

Reviewer 1-6: In the abstract and introduction, the phrase "machine-engineering" is used. I haven't heard this term before - perhaps "reactor design" or simply "engineering" would be more suitable.
Response: "machine-engineering" was replaced to "engineering"

Reviewer 1-7: The formatting of the introduction makes it quite difficult to read (first paragraph is 2 pages long). Please split this up into shorter, more focused paragraphs.
Response: We split the introduction (1st: flow and BO and 2nd: categorical variables)

Reviewer 1-8: The references selected to support the first introductory sentence (1-6) are somewhat strange. Why focus on the specific areas of electrochemical and microwave reactions? Surely it's more sensible to provide references to reviews on the areas of data-rich experimentation in general?
Response: We modified the first introductory sentence and added more appropriate references (see ref 1-4).
Previous sentence: Data-driven methodology enables the rapid identification of appropriate conditions for eco-friendly and sustainable processes such as electrochemical and microwave reactions
Revised sentence: Data-driven methodology enables the rapid identification of appropriate conditions for eco-friendly and sustainable chemical processes
Reviewer 1-9: In the description of one-hot encoding, the use of "via" is not really appropriate. Perhaps "represented by" would be more suitable.
Response: "via" was replaced to "represented by".

Reviewer 1-10: As stated above, the flow rates aren't really the variable being examined – the important result of this is varying the reaction time (residence time). It would probably be more suitable to discuss residence times, rather than flow rates (or at least have the reactor volume to hand, so the calculation can be made quickly by the reader).
Response: Residence time was inserted into Tables 1 and 2.

Table 1. Screening of reaction conditions for cross-coupling IQMA **1a** and 2-naphthol **2a**[a]

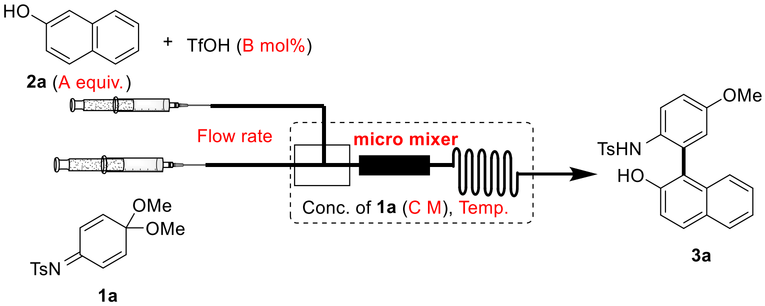

Entry	micro mixer	**2a** (equiv.)	TfOH (mol%)	Temp. (˚C)	Conc. of **1a** (M)	Flow rate (mL/min)	Residence time (min)	NMR yield[b] (%)
1	Comet X[c]	2.0	1.0	60	0.05	0.05	24	68
2	Comet X	3.0	0.5	40	0.01	0.2	6	73
3	β-type[d]	1.0	2.0	60	0.01	0.1	13.5	42
4	β-type	3.0	0.5	20	0.1	0.1	13.5	28
5	T-shaped[e]	1.0	2.0	40	0.05	0.05	16	55
6	T-shaped	2.0	1.0	20	0.1	0.2	4	75
7	T-shaped	2.0	1.0	20	0.1	0.15	5.5	81
8	Comet X	2.3	1.0	55	0.039	0.04	30	77
9	β-type	1.1	2.4	85	0.15	0.1	13.5	40
10	T-shaped	1.3	1.2	15	0.11	0.15	5.5	78
11	T-shaped	2.1	1.1	30	0.061	0.15	5.5	76
12	Comet X	2.8	1.0	50	0.014	0.11	11	79
13	Comet X	3.4	1.3	55	0.01	0.032	37.5	88
14	Comet X	2.2	1.7	15	0.1	0.014	85.5	44

15	Comet X	3.0	1.5	25	0.015	0.08	15	96 (93)[f]

[a]Reaction conditions: **1a** (0.065 mmol), **2a**, and TfOH, in degassed dry toluene. [b]1,3,5-Trimethoxybenzene was used as a standard. [c]Volume of Comet X microreactor = 2.4 mL. [d]Volume of β-type microreactor = 2.7 mL. [e]Volume of T-shaped microreactor = 1.6 mL. [f]Isolated yield.

Table 2. Screening of reaction conditions for cross-coupling QMA **4a** and 5-bromoresorcinol **2j**[a]

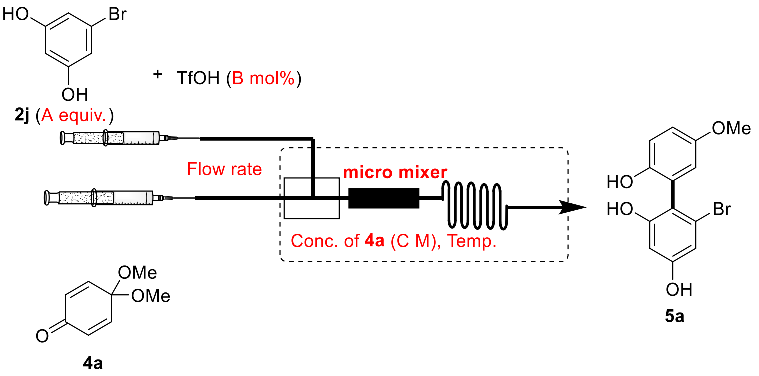

Entry	micro mixer	**2j** (equiv.)	TfOH (mol%)	Temp. (˚C)	Conc. of **4a** (M)	Flow rate (mL/min)	Residence time (min)	NMR yield[b] (%)
1	Comet X[c]	2.0	1.0	60	0.05	0.05	24	14
2	Comet X	3.0	0.5	40	0.01	0.2	6	35
3	β-type[d]	1.0	2.0	60	0.01	0.1	13.5	11
4	β-type	3.0	0.5	20	0.1	0.1	13.5	52
5	T-shaped[e]	1.0	2.0	40	0.05	0.05	16	4
6	T-shaped	2.0	1.0	20	0.1	0.2	4	29
7	Comet X	3.0	0.29	25	0.14	0.048	25	20
8	β-type	3.5	0.25	15	0.035	0.087	15.5	58
9	T-shaped	3.5	0.98	30	0.15	0.022	36	19
10	Comet X	3.4	0.37	35	0.056	0.13	9	10
11	β-type	2.4	0.34	15	0.019	0.041	33	40
12	T-shaped	2.4	0.73	15	0.011	0.060	13	19
13	β-type	3.2	0.25	15	0.068	0.062	22	20
14	β-type	3.5	0.53	15	0.067	0.097	14	43
15	β-type	3.2	0.35	30	0.044	0.068	20	69 (66)[c]

[a]Reaction conditions: **4a** (0.065 mmol), **2j**, and TfOH, in degassed dry toluene/EtOAc (10/1). [b]1,3,5-Trimethoxybenzene was used as a standard. [c]Volume of Comet X microreactor = 2.4 mL. [d]Volume of β-type microreactor = 2.7 mL. [e]Volume of T-shaped microreactor = 1.6 mL.[f]Isolated yield.

Reviewer 1-11: "With a batch size of three" is stated, yet there are 6 results in the first set. It is assumed that this represents an initial exploratory data set before the batches of 3 experiments, however, this is not explicitly mentioned. Some words should be added, to this effect.
Response: Six datapoints were collected to lower experimental number. Three datapoints as an initial dataset were inadequate to reach suitable conditions (see ref 53).

Reviewer 1-12: "Finally, we acquired three different experimental datasets (entries 13–15)." These aren't datasets, but single datapoints.
Response: "datasets" was replaced to "datapoints".

Reviewer 1-13: The experiment performed in Fig. 5 is almost the exact same control experiment from reference 46. This doesn't add any value to the work, since (unsurprisingly) the same result is observed.
Response: We moved Fig.5b from the manuscript to Fig S3 in ESI.

Reviewer 1-14: Reference 51 states that methods other than parallel LCB were unsuccessful, however there is no discussion of what parallel LCB actually entails. I also could not readily find this information online. Could the authors add a reference to a paper explaining this? Or add further explanation in the citation here?

Response: For further information on parallel BO, see: Gonzalez, J., Dai, P., Hennig P. & Lawrence, N. Batch Bayesian optimization via local penalization, *In proceedings of the 19th international conference on artificial intelligence and statistics*, **51**, 648-657 (2016) (see also ref 56).

Reviewer 1-15: The representation of the "micro mixer" is somewhat inaccurate. There is a T-piece combining the two streams, followed by a mixing element. However, it is assumed that the "T-shaped" experiments are performed in the absence of a micro mixer? In this case, it would be more representative to write "no mixer" instead of "T-shaped". Similarly, how does this affect the total reactor volume? Do the "T-shaped" experiments have a smaller volume?
Response: Sorry for our misunderstanding explanation. "T-shaped" is also micro mixer (see also the response for reviewer 1-1).

Reviewer 1-16: The experiments are quantified as follows: "1,3,5-Trimethoxybenzene was used as an internal standard." The authors should add an experimental procedure for these optimization experiments, explicitly stating when the "internal" standard was added. For a true internal standard, this should be added to one of the reaction streams – however 1,3,5-trimethoxybenzene can be reactive in some cases, so it is assumed that this was added after the reaction (often called an "external standard").
Response: We added 1,3,5-Trimethoxybenzene after the reaction. Many chemists traditionally call this type of standard "internal standard". Then, "internal standard" was replaced to "standard"

Reviewer #2 (Remarks to the Author):

The manuscript titled "Bayesian optimization-driven parallel-screening on multi-parameters of micromixer-type and organocatalytic conditions in the flow biaryl synthesis" written by S. Takizawa and coworkers reported optimization of reaction conditions for Brønsted acid catalyzed biaryl couplings using machine-learning, Bayesian optimization. The authors treated a categorical variable (mixer type) via one-hot encoding in this study. This is a simple and rather traditional approach. The authors identified flow conditions with shorter reaction times and lower catalyst loadings, although larger amounts of substrate 2 was required compared with the previously reported conditions. Gram scale syntheses was demonstrated via the developed flow conditions. Overall, it is somewhat difficult for this reviewer to support the submitted manuscript for publication in Communication Chemistry at the present stage. The reasons were shown below.

Reviewer 2-1
page 3, introduction section
The really pioneering work for multi-parameter screening and optimization of a chemical reaction reported by Lapkin, Bourne, and coworkers (Chem. Eng. J. 352, (2018) 277-282) should be cited in this manuscript. This Chem. Eng. J. manuscript reported the use of more advanced "multi-objective" Bayesian optimization in optimizing conditions of automated continuous-flow synthesis.
Response: According to the reviewer 2's comment, we cited the literature in ref 16

Reviewer 2-2
page 6
The authors indicated search range for temperature (20-60 ºC) in line 3, however, the search range was changed during the optimization (15-90 ºC) as shown in S54. It should be consistent during the optimization.
Response: To cover temperature reported by Kürti (= 100 ºC), we searched the indicated range (15-90 ºC) (see also ref 54).

Reviewer 2-3
page 6
The authors described "the desired product 3a was obtained in 93% isolated yield within 15 min..." Readers might think the reaction and purification were finished within 15 min. This sentence should be revised.
Response: Sorry for our misunderstanding description. "15 min" was residence time. We corrected sentence.
Previous sentence: Gratifyingly, the desired product 3a was obtained in 93% isolated yield within 15 min under the reaction conditions in entry 15.
Revised sentence: After correction: Gratifyingly, the desired product 3a was obtained in 93% isolated yield using a microflow system (Comet X micro mixer, flow rate = 0.08 mL/min, and residence time 15 minutes) as shown in entry 15.

Reviewer 2-4
page 8
Interestingly, the optimal mixer was different between the first reaction and the second reaction. The authors have better to show the mixing efficiency and heat transfer efficiency of the used mixers. This information should be valuable for readers to speculate the reason why the optimal mixer was different.
Response: In case of low yield for the first reaction using IQMAs **1**, side products were observed. In contrast, for the second

reaction using QMAs **4**, conversion of **1** was low. Comet-X was better than other mixers for the first reaction owing to suppression of the side reactions by its high efficiency of turbulent mixing. β-type was more appropriate than other mixers for the second reaction because it could decrease the pressure drop, which affects high velocity flow.

Reviewer 2-5
page 10
The optimal catalyst loading and concentration of 4 were significantly different between the first and the second reaction. This was also interesting point of this study. The authors should discuss the reason based on the plausible reaction mechanisms.
Response: For the first reaction and the second reaction, catalyst loading was different, but concentration of catalyst was similar (0.23 M and 0.15 M, respectively). Lower concentration of IQMAs **1** was critical to suppress decomposition of **1** for the first reaction. In contrast, when we tested the same concentration for the second reaction, low conversion of QMAs **4** was observed. Therefore, higher concentration was suitable for the second reaction.

We prepared "Comparison of the suitable reaction conditions for the two reactions" as a new section in the manuscript.

Reviewer 2-6
S2, SI
Although brief explanation for T-shaped and beta-mixers was shown in ref. 50, the information should be also shown in general information in SI.
Response: We added the detailed information on T-shaped, beta-type, and comet-X as Fig. S2 in ESI .

Reviewer 2-7
S4, SI
When flow rate was changed, how was reaction time? Was reaction time maintained by changing the length of tubes? It should be mentioned in SI.

Response: We did not change the length of tubes to maintain the residence time. To clarify how the residence time changed upon varying the flow rate, we have added the residence time in each entry in Tables 1 and 2.

Reviewers' comments:

Reviewer #1 (Remarks to the Author):

Following the quite significant changes to the manuscript, I would now support publication.

One change should still be made: An additional "Villermaux-Dushman" graph has been added to the supporting information (Figure S2). There is no information around how these experiments were carried out (or may have been taken from another publication?), and two of the mixers represented are not mentioned in this manuscript. Therefore, this should be simply removed from the submission.

Reviewer #2 (Remarks to the Author):

This reviewer 2 read response to the reviewers' comments and still have concerns about the revised manuscript.

Against reviewer comment 2-2
The authors answered "To cover temperature reported by Kürti (= 100 oC), we searched the indicated range (15-90 oC) (see also ref 54)."
However, it is still unclear why the authors did not select initial 6 points from this range (15-90 oC). I also noticed that the authors changed search ranges for amount of 2a, concentration of 1a, flow rate, and catalyst loading between initial examination and BO. This reviewer would like to know reason for altering the search ranges.

Against reviewer comment 2-4
The authors answered "In case of low yield for the first reaction using IQMAs 1, side products were observed. In contrast, for the second reaction using QMAs 4, conversion of 1 was low. Comet-X was better than other mixers for the first reaction owing to suppression of the side reactions by its high efficiency of turbulent mixing. β-type was more appropriate than other mixers for the second reaction because it could decrease the pressure drop, which affects high velocity flow."
However, it does not seem to make sense. As the authors described, turbulent flow improve mixing efficiency and the high velocity flow thanks to low pressure drop usually improve mixing efficiency. This reviewer speculates that the mixing efficiency of Comet X mixer is better than that of T-shaped mixer and beta-type mixer at examined low flow rate (0.08 mL/min, revised Scheme S1). The authors should examine the use of T-shape (alpha type) mixer at high flow rates (i.e. 1.5 mL/min) to improve mixing efficiency with long reaction tube to maintain residence time. If the authors observe improved yield, it is possible that the mixing efficiency of the mixer is important for reaction outcome.

Professor Ph.D Takashi Washio
Associate Professor Ph.D Shinobu Takizawa
The Institute of Scientific and Industrial Research,
Osaka University
Mihogaoka, Ibaraki, Osaka 567-0047, JAPAN
Phone: (+81) 6-6879-8467
FAX: (+81) 6-6879-8469
e-mail: washio@ar.sanken.osaka-u.ac.jp
taki@sanken.osaka-u.ac.jp

Response to referees

Reviewer #1 (Remarks to the Author): Following the quite significant changes to the manuscript, I would now support publication.

Reviewer #1-1: One change should still be made: An additional "Villermaux-Dushman" graph has been added to the supporting information (Figure S2). There is no information around how these experiments were carried out (or may have been taken from another publication?), and two of the mixers represented are not mentioned in this manuscript. Therefore, this should be simply removed from the submission.
Response: According to the reviewer's comment, we removed "Villermaux-Dushman" graph and "Pressure drop" graph from Figure S2.

Reviewer #2 (Remarks to the Author): This reviewer 2 read response to the reviewers' comments and still have concerns about the revised manuscript.

Reviewer #2-1: The authors answered "To cover temperature reported by Kürti (= 100 oC), we searched the indicated range (15-90 oC) (see also ref 54)." However, it is still unclear why the authors did not select initial 6 points from this range (15-90 oC). I also noticed that the authors changed search ranges for amount of 2a, concentration of 1a, flow rate, and catalyst loading between initial examination and BO. This reviewer would like to know reason for altering the search ranges.
Response: We appreciate your comments. We performed experiment for initial points at lower temperature (<60 ˚C) because a decomposition of starting material **1a** was observed at 60 ˚C or higher during the pre-screening of catalyst. However, the broader searching ranges than examination prevent missing suitable conditions. According to the conditions reported by Kürti, we set broader searching ranges by BO.

Reviewer #2-2: The authors answered "In case of low yield for the first reaction using IQMAs 1, side products were observed. In contrast, for the second reaction using QMAs 4, conversion of 1 was low. Comet-X was better than other mixers for the first reaction owing to suppression of the side reactions by its high efficiency of turbulent mixing. β-type was more appropriate than other mixers for the second reaction because it could decrease the pressure drop, which affects high velocity flow." However, it does not seem to make sense. As the authors described, turbulent flow improve mixing efficiency and the high velocity flow thanks to low pressure drop usually improve mixing efficiency. This reviewer speculates that the mixing efficiency of Comet X mixer is better than that of T-shaped mixer and beta-type mixer at examined low flow rate (0.08 mL/min, revised Scheme S1). The authors should examine the use of T-shape (alpha type) mixer at high flow rates (i.e. 1.5 mL/min) to improve mixing efficiency with long reaction tube to maintain residence time. If the authors observe improved yield, it is possible that the mixing efficiency of the mixer is important for reaction outcome.
Response: We are grateful to the reviewer for the valuable suggestion. We performed the reaction using T-shaped mixer at high flow rate with longer reaction tube. However, both results were almost same (50% and 54% yields, see Figure R1). Therefore, the difference in the mixer suitable for each reaction is considered to be due to the difference in the respective stirring methods (see Figure S2). We added mixing mechanism of β-type mixer (see Figure S2 in ESI) and the reference for flow reaction using β-type mixer (see ref 2 in ESI).
Additionally, the following comments were inserted in Ref 59: "The reason of low yield on the reaction using IQMAs **1** (Table 1) are attributed to formation of side products. In case of the reaction using QMAs **4** (Table 2), the reason of low yield of desired product was low conversion of **4**. The difference in the mixer suitable for each reaction is considered to be due to the difference in the respective stirring methods".

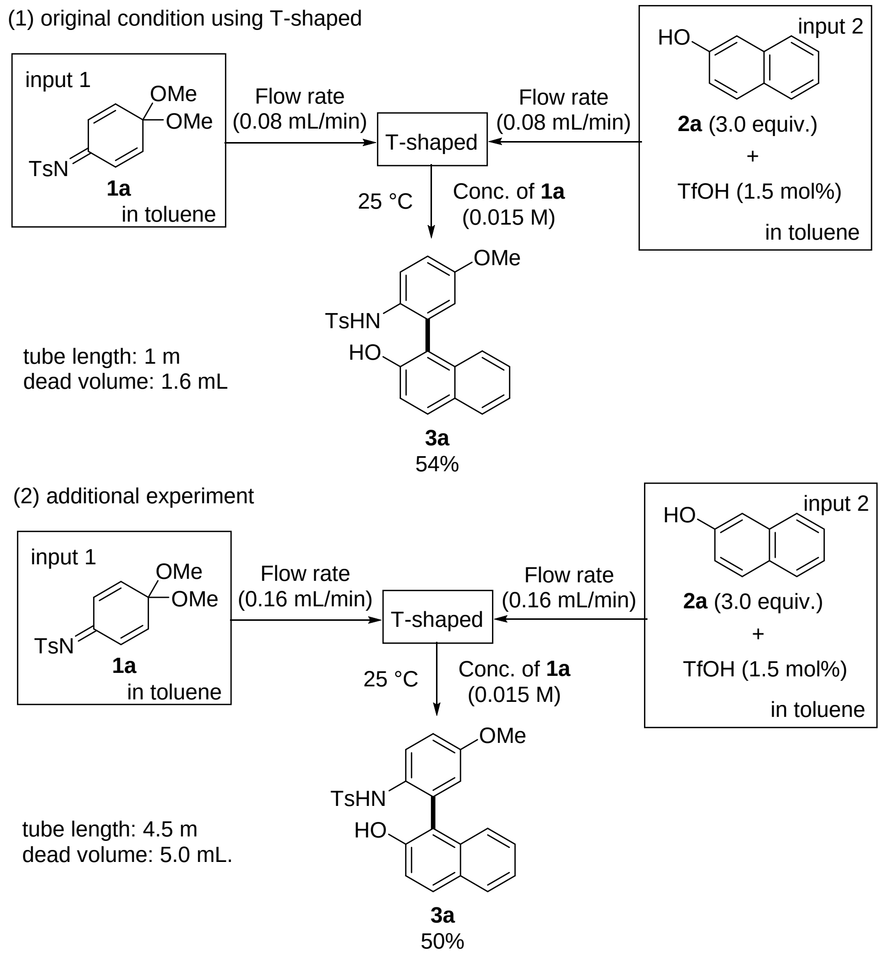

Figure R1. the reaction using T-shaped mixer: (1) original condition; (2) at higher flow rate with longer tube

REVIEWERS' COMMENTS:

Reviewer #2 (Remarks to the Author):

The reviewer 2 read response to the reviewers comments and now was satisfied with the revision.

Although the authors need not perform additional experiments or revise their manuscript, let this reviewer make one comment.

The authors performed the additional experiment at flow rate of 0.16 mL/min, as shown in Figure R1(2). However, the slight increase of flow rate from 0.08 to 0.16 should not significantly improve mixing efficiency. Because mixing efficiency does not linearly increase with the increase of flow rate (M. Engler, N. Kockmann, T. Kiefer, P. Woias, Chem. Eng. J. 101, 315, (2004)). In order to generate engulfment flow, sufficiently high flow rate (>1.5 mL/min) conditions are required. The authors have better to keep this in mind for the future work.
